# Supplementary material for: Experience of and access to maternity care in the UK by immigrant women: a narrative synthesis systematic review
Source: BMJ Open. 2019 Dec 31;9(12):e029478. doi: 10.1136/bmjopen-2019-029478 (PMC6955508; doi:10.1136/bmjopen-2019-029478)
Supplement: Supplementary data [file bmjopen-2019-029478supp001.pdf]

SUPPLEMENTARY FILES

File 1: Search Strategy
File 2: Bibliographic databases searched
File 3: Inclusion/Exclusion critiera
File 4: Excluded papers and rationale
File 5: Master table of included studies
File 6: Characteristics of study participants

File 1: Search Strategy

:Review search strategy - Medline

1	Maternal Health Services/ or Postnatal Care/ or Preconception Care/ or Prenatal Care/ or Perinatal Care/ or Infant Care/ or Midwifery/ or Obstetrics/ or General Practitioners/ or Primary Health Care/ or Family Health/	162335	
2	((maternal or child* or baby or babies or fetus* or fetal* or	119288	Field modified from .mp. to .ti,ab.
3	((birth* or matern* or mother* or pregnan* or childbearing or child-bearing or prenatal or pre-natal or postnatal or post-natal or perinatal or peri-natal or **preconception** or pre-conception or antenatal or ante-natal or postpartum or puerperium) adj3 (health* or nurs* or care or service*)).ti,ab.	65240	Field modified from .mp. to .ti,ab.
4	exp Midwifery/ or exp Obstetric Nursing/ or exp	47851	Field modified from .mp. to .ti,ab.
5	exp Health Services Accessibility/ or exp	1829149	
6	5 and (matern* or child* or baby or babies or fetus* or fetal* or embryo* or obstetric* or birth* or mother* or pregnan* or childbearing or child-bearing or prenatal or pre-natal or postnatal or post-natal or perinatal or peri-natal or preconception or pre-conception or antenatal or ante-natal or postpartum or puerperium).ti,ab.	253992	Limit 5 to female did not sufficiently focus previous search strategy so text terms used instead; Field modified from .mp. to .ti,ab.
7	1 or 2 or 3 or 4 or 6	490776	
8	("use" or access* or utili* or consum* or block* or hurdl* or barrier* or hindr* or hinder* or obstacle* or exclu* or discrimin* or disparit* or disproportion* or inequal* or unequal* or inadequat* or insuffic* or stratif* or limit* or lack* or unreliab* or poor* or poverty* or depriv* or disadvantag* or insecur* or insensit* or status* or entitl* or uninform* or ill-inform* or benefit* or interven* or deliver* or effective* or cost effective*).ti,ab.	8190143	

9	5 and 8	761677	use of/access to health services
10	"Emigrants and Immigrants"/ or Refugees/ or "Transients and Migrants"/ or "Emigration and Immigration"/	42486	
11	(((established or "first generation*" or new* or recent* or current*) adj3 (migrant* or migrat* or immigrant* or immigrat* or emigrant* or emigrat* or emigre* or expat* or (ex adj pat*) or transient* or alien*)) or newcomer* or (new adj comer*) or incomer*	14965	Revised to focus on established or new immigrant groups; Field modified from .mp. to .ti,ab.
12	(refugee* or (asylum adj seek*) or asylee* or (refused adj3 (asylum* or refugee*)) or (displaced adj person*) or exile* or (new adj arrival) or (country adj2 (birth or origin)) or **transnational*).ti,ab.**	13603	Field modified from .mp. to .ti,ab.
13	(foreigner* or (foreign adj (born or citizen* or national* or origin*)) or (non adj (citizen* or native*)) or ((adoptive or naturali#ed) adj (citizen* or resident*)) or overstay* or trafficked or "spousal migrant*").ti,ab.	10542	Additional migrants terminology; Field modified from .mp. to .ti,ab.
14	("non-UK-born" or "born outside the UK" or "length of residence in the UK" or (("not lawful*" or "not legal*" or unlawful* or illegal* or unauthori#ed* or "not authori#ed" or uncertain or insecure or illegal or legal or irregular* or refused or **undocumented)** adj3 (residen* or immigrant* or imigrat* or migrant* or migrat*))).ti,ab.	1375	Additional migrants terminology; Field modified from .mp. to .ti,ab.
15	exp Ethnic Groups/ or (ethnic* or ethno* or race or racial*).ti,ab.	282908	Expanded ethnic terminology; Field modified
16	exp african continental ancestry group/ or exp asian continental ancestry group/ or exp Caribbean Region/	152457	Additional ethnic terminology to specify South Asian and African Caribbean groups;
17	exp Vulnerable Populations/ or ((vulnerab* or disadvantag* or minorit*) adj3 (individ* or	35822	Expanded vulnerable populations terminology; Field

18	("Black and Minority Ethnic" or "Black & Minority ethnic" or BME or african caribbean* or afro caribbean* or black african* or (west adj (indies or indian*))).ti,ab.	7587	Expanded ethnic terminology; Field modified from .mp. to .ti,ab.
19	(south asia* or afghan* or bangladesh*	163384	
20	10 or 11 or 12 or 13 or 14 or 15 or 16 or 17 or 18 or 19	589408	All ethnic/migrant groups
21	7 and 9 and 20	20754	Maternity health services AND [illegible]
22	limit 21 to yr="1990 -Current"	18783	Time range expanded
23	higginbottom*.au.	214	
24	22 and 23	9	Check of strategy retrieval of known relevant records

File 2: Bibliographic databases searched

Databases searched.

- Ovid MEDLINE 1948– and MEDLINE In-Process and Other Non-Indexed Citations to daily update
- Ovid EMBASE 1980–2017 Week 11
- Ovid PsycINFO 1972–March Week 3 2017
- CINAHL Plus with Full Text/EBSCOHost to 2017
- MIDIRS on Ovid 1971 to April 2017
- Thomson Reuters Web of Science* 1900–2017
- ASSIA on ProQuest 1987–current
- HMIC on Ovid 1979–January 2017
- POPline (via http:// www.popline.org/) 1970 to the present

* Thomson Reuters Web of Science 1900-2017 includes the following:

- Science Citation Index Expanded (SCI-EXPANDED) 1900–2017
- Social Sciences Citation Index (SSCI) 1956–2017
- Conference Proceedings Citation Index - Science (CPCI-S) 1990–2017
- Conference Proceedings Citation Index - Social Science and Humanities (CPCI-SSH) 1990–2017
- Book Citation Index - Science (BKCI-S) 2008–2017
- Book Citation Index - Social Science and Humanities (BKCI-SSH) 2008–2017
- Emerging Sources Citation Index (ESCI) - 2015–2017

List of databases for searching grey literature

- **Cochrane Database of Systematic Reviews**
  http://www.thecochranelibrary.com/

Theses

- **Nottingham eDissertations**
  http://edissertations.nottingham.ac.uk/
- selected dissertations from UoN

- **Nottingham eTheses**
  http://etheses.nottingham.ac.uk/
- research degree theses awarded by UoN
- pilot project so not compulsory to submit, therefore not all these included

- **Index to Theses**
  http://www.theses.com/
  theses (incl. abstracts) accepted for higher degrees by universities in GB and Ireland
  now part of ProQuest Dissertations & Theses – UK & Ireland

- **Networked Digital Library of Theses & Dissertations**
  http://www.ndltd.org/
- includes theses and dissertations submitted to over 200 universities worldwide

- **EThOS – British Library Electronic Theses Online**
  http://ethos.bl.uk/Home.do

- **DEEP – DART Europe**
  http://www.dart-europe.eu

- **ProQuest Dissertations & Theses A&I** (worldwide coverage)
  http://search.proquest.com/pqdt/index?accountid=8018

Research Funders

- **Wellcome Trust**
  http://www.wellcome.ac.uk/
- Global charitable foundation supporting biomedical research and the medical humanities
- Provides support with funding, managing grants, education resources, application of research

- **Research Councils UK**
  http://www.rcuk.ac.uk/
- Support research across all academic disciplines
- Offer funding opportunities, international collaborations and training

- **Medical Research Council**
  http://www.mrc.ac.uk/index.htm
- Publicly funded organisation dedicated to improving human health
- Supports research across medical sciences in universities, hospitals and MRC Councils

- **Science and Technology Facilities Council**
  http://www.stfc.ac.uk/
- Independent public body of the Department of Business, Innovations and Skills
- Supports researchers across the sciences with the academic and industrial communities

- **National Institute for Health and Care Excellence**
  http://www.nice.org.uk/

- **Institute for Public Policy and Research**
  http://www.ippr.org/

- **ESRC**
  http://www.esrc.ac.uk/
  REGARD database

- **Clinical Research Network** (part of the NHS National Institute for Health Research)
  http://www.crncc.nihr.ac.uk
  From the NIHR portal
  (https://portal.nihr.ac.uk/Pages/NIHRResearchInfoStatement.aspx):
  - The repository for this information is the Portfolio Database, which currently contains approximately 2,000 studies, and can be accessed for public searching. Detailed instructions on how to search the Portfolio Database are available at http://www.ukcrn.org.uk/index/clinical/portfolio_new/P_search.html, and the Portfolio database is available at http://public.ukcrn.org.uk/search.
  - The National Research Register has been archived as a public resource and to support historical analysis. The archive is available via the National Institute for Health Research Portal http://portal.nihr.ac.uk.

Statistics

- **Department of Health**
  https://www.gov.uk/government/publications?publication_filter_option=statistics

- **UK Data Archive**
  http://www.data-archive.ac.uk

- **UK National Statistics**
  http://www.statistics.gov.uk

- **OECD Statistics Portal**
  http://www.oecd.org/statistics

- **World Health Organisation**

http://www.euro.who.int/en

- **NICE Evidence Services** (formerly NHS Evidence)
- Evidence search
- Clinical Knowledge Summaries
- NICE guidelines
- Journals and databases
- A-Z of topics – e.g. Diabetes
- Medicines information
- Public health information

https://www.evidence.nhs.uk

- **HMIC** (Health Management Information Consortium) – on Ovid
- combined database of the Department of Health, plus the King's Fund Information & Library Service
- official publications, journal articles, grey literature
- health service policy, management & admin, quality of hospitals, nursing, primary care and public health; occupational health; control/regulation of medicines

- **PAIS International**

http://search.proquest.com/pais?accountid=8018 (via ProQuest)

- includes e.g.: gov docs, statistical directories, grey lit, research reports – mostly in social sciences

- **Open Grey**

http://www.opengrey.eu/

- open access to grey literature published in Europe, including reports, dissertations, conference proceedings, official publications

- **Mednar**

http://mednar.com/mednar/desktop/en/green/search.html
one-stop federated search engine designed for professional medical researchers

- **WorldwideScience**

http://worldwidescience.org/

- **OAIster**

http://www.oclc.org/oaister.en.html
catalog of millions of records from open access collections worldwide using the Open Archives Initiative Protocol for Metadata Harvesting (OAI-PMH)

- **Internet Archive Wayback Machine**

http://archive.org/web/
aims to provide permanent access to historical collections that exist in digital format

File 3: Inclusion/Exclusion criteria

Inclusion Criteria

Population	Immigrant women from any country other than England, Scotland, Northern Ireland or Wales
Phenomena of Interest	Maternity care
Context Setting	United Kingdom
Study designs	Qualitative, quantitative and mixed methods studies
Language	English
Date limitations	Jan 1990 - Jan 2018
**Exclusion Criteria**	
Context	Studies located in any country other than England, Scotland, Northern Ireland or Wales
Participants	Black and minority ethnic women born in the United Kingdom
Study Design	Non-empirical research, opinion pieces or editorial

File 4: Excluded papers and rationale

Excluded studies with reasons for exclusion.

Exclusion number	Reference	Reasons for exclusion
**1**	Bowler I. 'They're not the same as us': midwives' stereotypes of South Asian descent maternity patients. *Sociol Health Illn*. 1993 Mar 1;15(2):157-78.	Presented professionals' perspectives: focused on midwife interviews and observational data on midwives.
**2**	Straus L, McEwen A, Hussein FM. Somali women's experience of childbirth in the UK: perspectives from Somali health workers. *Midwifery*. 2 09 Apr 1;25(2):181-6.	Presented professionals' perspectives: interviewed Somali health workers and not the immigrant women.
**3**	Bowler IM. Stereotypes of women of Asian descent in midwifery: some evidence. *Midwifery*. 1993 Mar 1;9(1):7-16.	Presented professionals' perspectives: interviewed midwives.
**4**	Haith-Cooper M, Bradshaw G. Meeting the health and social needs of pregnant asylum seekers: midwifery students' perspectives. Part 2: Dominant discourses and approaches to care. *Nurse Educ Today*. 2013 Aug 1;33(8):772-7.	Presented professionals' perspectives: focused on midwifery students' perceptions.
**5**	Haith-Cooper M, Bradshaw G. Meeting the health and social care needs of pregnant asylum seekers; midwifery students' perspectives: Part 3; The pregnant woman within the global context; an inclusive model for midwifery education to address the needs of asylum-seeking women in the UK. *Nurse Educ Today*. 2013 Sep 1;33(9):1045-50.	Presented professionals' perspectives: interviewed midwives.
**6**	Balaam MC, Kingdon C, Thomson G, Finlayson K, Downe S. 'We make them feel special': the experiences of voluntary sector workers supporting asylum-seeking and refugee women during pregnancy and early motherhood. *Midwifery*. 2016 Mar 1;34:133-40.	Presented professionals' perspectives.
**7**	Richards J, Kliner M, Brierley S, Stroud L. Maternal and infant health of Eastern Europeans in Bradford, UK: a qualitative study. *Community Practitioner*. 2014 Sep 1;87(9):33.	Presented professionals' perspectives.
**8**	Redshaw M, Heikkilä K. Ethnic differences in women's worries about labour and birth. *Ethn Health*. 2011 Jun 1;16(3):213-23.	Mixed sample of UK-born BME and immigrant women with no separate findings reported for immigrant women.

**9**	Darwin Z, Green J, McLeish J, Willmot H, Spiby H. Evaluation of trained volunteer doula services for disadvantaged women in five areas in England: women's experiences. *Health Social Care Community*. 2017 Mar 1;25(2):466-77.	Mixed sample of UK-born BME and immigrant women with no separate findings reported for immigrant women.
**10**	Dunne FP, Brydon PA, Proffitt M, Smith T, Gee H, Holder RL. Fetal and maternal outcomes in Indo-Asian compared to Caucasian women with diabetes in pregnancy. *QJM*. 2000 Dec 1;93(12):813-8.	Mixed sample of Indo-Asian women born inside and outside the UK with no separate findings for immigrant women.
**11**	Ball HL, Moya E, Fairley L, Westman J, Oddie S, Wright J. Infant care practices related to sudden infant death syndrome in South Asian and White British families in the UK. *Paediatr Perinat Epidemiol*. 2012 Jan 1;26(1):3-12.	Focused on care of infants aged 2-4 months, but our chosen limit of maternity care was only up to 6 weeks after birth.<br>Mixed sample of UK-born and non-UK-born women with no separate findings for immigrant women.
**12**	McCarthy R, Haith-Cooper M. Evaluating the impact of befriending for pregnant asylum-seeking and refugee women. *Br J Midwifery*. 2013 Jun;21(6):404-9.	Not an empirical: the study does not report its methodology, sampling, or data analysis.
**13**	Streetly A, Grant C, Bickler G, Eldridge P, Bird S, Griffiths W. Variation in coverage by ethnic group of neonatal (Guthrie) screening programme in south London. *BMJ*. 1994 Aug 6;309(6951):372-4.	Mixed sample of ethnic groups with no separate findings for immigrant women.
**14**	Burchill J, Pevalin DJ. Demonstrating cultural competence within health-visiting practice: working with refugee and asylum-seeking families. *Divers Equal Health Care*. 2014;11(2).	Weak focus on maternity care: just two quotes on the influence of cultural sensitivity training and on addressing female genital mutilation (FGM) in maternity.
**15**	Dormandy E, Michie S, Hooper R, Marteau TM. Low uptake of prenatal screening for Down syndrome in minority ethnic groups and socially deprived groups: a reflection of women's attitudes or a failure to facilitate informed choices? *Int J Epidemiol*. 2005 Feb 28;34(2):346-52.	Not clear if sample was composed of immigrant women: no separate findings for immigrant women.
**16**	Henderson J, Gao H, Redshaw M. Experiencing maternity care: the care received and perceptions of women from different ethnic groups. *BMC Pregnancy Childbirth*. 2013 Dec;13(1):196.	Not clear if sample was composed of immigrant women: no separate findings for immigrant women.
**17**	Ingram J, Cann K, Peacock J, Potter B. Exploring the barriers to exclusive breastfeeding in Black and minority ethnic groups and young mothers in the UK. *Matern Child Nutr*. 2008 Jul 1;4(3):171-80.	Mixed sample of UK-born and immigrant women with no separate findings for immigrant women.

**18**	Parsons L, Day S. Improving obstetric outcomes in ethnic minorities: an evaluation of health advocacy in Hackney. *J Public Health*. 1992 Jun 1;14(2):183-91.	Not clear if sample was composed of immigrant women.
**19**	Knight M, Kurinczuk JJ, Spark P, Brocklehurst P. Inequalities in maternal health: national cohort study of ethnic variation in severe maternal morbidities. *BMJ*. 2009 Mar 4;338:b542.	Mixed sample of both UK- and foreign-born BME with no separate findings for immigrant women.
**20**	Almond P, Lathlean J. Inequity in provision of and access to health-visiting postnatal depression services. *J Adv Nurs*. 2011 Nov 1;67(11):2350-62.	Focused on professionals' perspective. Eight of the nine participants were immigrant women, but just three brief quotes were reported from immigrant Bangladeshi women. Authors did not reply to our request for clarification of the immigrant status of the sample.
**21**	Row MA, Nevill AM, Young DB, Adamson-Macedo EN. (2013) Promoting positive postpartum mental health through exercise in ethnically diverse priority groups. *Divers Equal Health Care*. 2013;10(3)185-195.	Mixed sample of minority ethnicity women born in and outside the UK with no separate findings for immigrant women.
**22**	Hemingway H, Saunders D, Parsons L. Social class, spoken language and pattern of care as determinants of continuity of carer in maternity services in east London. *J Public Health*. 1997 Jun 1;19(2):156-61.	Mixed sample of women with and without English as a first language. We used lack of English as a proxy for immigrant, but only one finding was reported for a non-English sample (i.e., the presence of an advocate who could translate for women visiting midwives or doctors). Did not receive a reply from the authors regarding the immigrant status of the sample.
**23**	Ingram J, Johnson D, Hamid N. South Asian grandmothers' influence on breast feeding in Bristol. *Midwifery*. 2003 Dec 1;19(4):318-27.	No clarity on the immigrant status of the sample and no separate findings for immigrant women. Did not receive a reply from the authors on the immigrant status of the sample.
**24**	Gardner PL, Bunton P, Edge D, Wittkowski A. The experience of postnatal depression in West African mothers living in the United Kingdom: A qualitative study. *Midwifery*. 2014 Jun 1;30(6):756-63.	No clarity on the immigrant status of the sample and no separate findings for immigrant women.
**25**	Kelly Y, Panico L, Bartley M, Marmot M, Nazroo J, Sacker A. Why does birthweight vary among ethnic groups in the UK? Findings from the Millennium Cohort Study. *J Public Health*. 2008 Jul 21;31(1):131-7.	Not clear if participants included immigrant women. Did not receive a reply from the authors on the immigrant status of the sample.

**26**	Beake S, McCourt C, Bick D. Women's views of hospital and community-based postnatal care: the good, the bad and the indifferent. *Evid Based Midwifery*. 2005 Dec 1;3(2):80-7.	Not clear if participants included immigrant women. Did not receive a reply from the authors on the immigrant status of the sample.
**27**	Ahmed S, Green J, Hewison J. Antenatal thalassaemia carrier testing: women's perceptions of information and consent. *J Med Screen*. 2005 Jun 1;12(2):69-77.	Weak focus on maternity care: main focus was on an ancestry issue.
**28**	Dyson SM, Cochran F, Culley L, Dyson SE, Kennefick A, Kirkham M, Morris P, Sutton F, Squire P. Ethnicity questions and antenatal screening for sickle cell/thalassaemia (EQUANS) in England: observation and interview study. *Crit Public Health*. 2007 Mar 1;17(1):31-43.	Not clear if participants included immigrant women.
**29**	Baker D, Garrow A, Shiels C. Inequalities in immunisation and breast feeding in an ethnically diverse urban area: cross-sectional study in Manchester, UK. *J Epidemiol Community Health*. 2011 Apr 1;65(4):346-52.	Not clear if participants included immigrant women and not focused on maternity care. Did not receive a reply from the authors on the immigrant status of the sample.
**30**	Dyson SM, Chambers K, Gawler S, Hubbard S, Jivanji V, Sutton F, Squire P. Lessons for intermediate- and low-prevalence areas in England from the Ethnicity Questions and Antenatal Screening for sickle cell/thalassaemia (EQUANS) study. *Divers Health Social Care*. 2007 Jun 1;4(2).	Not clear if participants included immigrant women and not focused on maternity care.
**31**	Sim JA, Ulanika AA, Katikireddi SV, Gorman D. 'Out of two bad choices, I took the slightly better one': Vaccination dilemmas for Scottish and Polish migrant women during the H1N1 influenza pandemic. *Public Health*. 2011 Aug 1;125(8):505-11.	Not focused on maternity care.
**32**	Wittkowski A, Zumla A, Glendenning S, Fox JR. The experience of postnatal depression in South Asian mothers living in Great Britain: a qualitative study. *J Reprod Infant Psychol*. 2011 Nov 1;29(5):480-92.	Mixed sample with only two quotes related to immigrant women.
**33**	McFadden A, Atkin K, Renfrew MJ. The impact of transnational migration on intergenerational transmission of knowledge and practice related to breast feeding. *Midwifery*. 2014 Apr 1;30(4):439-46.	Not focused on maternity care.
**34**	Datta S, Alfaham M, Davies DP, Dunstan F, Woodhead S, Evans J, Richards B. Vitamin D deficiency in pregnant women from a non-European ethnic minority population – an interventional study. *BJOG*. 2002 Aug 1;109(8):905-8.	Not clear if participants included immigrant women. Did not receive a reply from the authors on the immigrant status of the sample.

**35**	McLeish J, Redshaw M. ‘I didn’t think we’d be dealing with stuff like this’: a qualitative study of volunteer support for very disadvantaged pregnant women and new mothers. *Midwifery*. 2017 Feb 1;45:36-43.	Mixed sample with no separate findings for immigrant women.

File 5: Master table of included studies

Summary of included studies

Reference	Study aim	Region	Methodology	Theory or Framework	Setting	Data analysis	Sample and mode of recruitment
109	To establish efficacy of linkworker services (an intervention) introduced for non-English-speaking Asian women in multi-racial health districts	Not specified	Quantitative survey: 21-item questionnaire	Not specified.		Qualitative: content analysis	Questionnaire to the Heads of Midwifery Services in 30 multi-racial district health authorities. 20 responded. Sample is not immigrant women, however this is an evaluation of an intervention
115	To develop a reliable and valid questionnaire to evaluate satisfaction with maternity care in Sylheti-speaking Bangladeshi women.	London.	Mixed methods: two-stage psychometric study. Firstly, a Sylheti-language questionnaire regarding Bangladeshi women's experiences of maternity services was translated and culturally adapted from an English-language questionnaire using focus groups, in-depth interviews, and iterative methods. Secondly, quantitative psychometric methods were used to field test and evaluate the acceptability, reliability, and validity of this questionnaire.	Not specified.	Not specified.	Qualitative: thematical analysis. Quantitative: validity of an instrument.	Located at four hospitals providing maternity services in London, UK. Study participants included 242 women from the London Bangladeshi communities who were in the antenatal (at least 4 months pregnant) or postnatal phase (up to 6 months after delivery). The women spoke Sylheti, a language with no accepted written form. In stage one purposive samples of 40 women in the antenatal or postnatal phase participated, along with one convenience sample of six women in the antenatal phase and three consecutive samples of 60 women in the postnatal phase. In stage two, 135 women (main sample) completed the questionnaire 2 months after delivery (82% response rate), and 50 women (retest sample) from the main sample completed a second questionnaire 2 weeks later (96% response rate).

88	To study the maternity care experiences of Somali refugee women in an area of West London. This article focused particularly on findings relating to the language barrier, which to a large degree underpinned or at least aggravated other problems the women experienced.	West London.	Qualitative: case study. Six semi-structured interviews and two focus groups (with six participants each).	Not specified.	Not specified.	Qualitative: thematical analysis.	Snowball sampling: 12 Somali women were selected from a larger survey involving 1400 women.
89	To undertake a qualitative study of the maternity experiences of 33 asylum seekers.	London, Plymouth, Hastings, Brighton, Oxford, Manchester, and King's Lynn.	Qualitative.	Not specified.	Home or a neutral location.	Qualitative: content analysis.	Convenience and snowball sampling of recent asylum seekers. Based on semi-structured interviews carried out in seven English cities.
116	A Sure Start local programme had funded a Bangladeshi support worker to provide bilingual breastfeeding support to childbearing Bangladeshi women, many of whom were not fluent in English. This study aimed to conduct a short evaluation of the impact of this work on the uptake and duration of breastfeeding among these women.	Tower Hamlets.	Mixed methods: the survey questionnaire included some open and closed questions about the women's intention to feed; their current feeding methods; the breastfeeding support and information they received antenatally, during the hospital stay, and postnatally; overall views on the information and support received; and some demographic details. Eleven interviews were conducted by telephone in Sylheti (a dialect that has no written format), three in English and one in Urdu (using a female family member to translate). Interviews took between 15 and 30	Not specified.	Not specified (survey conducted by telephone).	Qualitative: content analysis of a questionnaire (open and closed questions).	The two midwives and the support worker had provided breastfeeding support to 194 women during a one-year period (September 2001 to August 2002). Of these, 80 women received help from the support worker alone. The majority of these 80 women were Bangladeshi. For the evaluation, 15 women were randomly selected from these 80 women.

			minutes to complete.				
**{Rowe, 2008 #18 3}**	To identify any social or ethnic differences in access to antenatal care and to quantify the effects of any such differences using data collected in a survey of women's experiences of antenatal screening.	England.	Quantitative: a cross-sectional survey using a postal questionnaire.	Not specified.	Not specified.	Quantitative: cross-sectional analysis.	A stratified clustered random sampling strategy was used. Hospitals in England were stratified according to ethnic mix. To ensure inclusion of an adequate number of women from black and minority ethnicity (BME) backgrounds, hospitals with ≥ 15% of women of BME origin were oversampled. Pregnant women aged ≥16 years and receiving care in 15 participating hospitals were sent a postal questionnaire at 27–31 weeks of gestation.

110	To compare the health behaviours both antenatally (smoking and alcohol consumption) and postnatally (initiation and duration of breast feeding) of mothers who have white British or Irish heritage with those of mothers from ethnic minority groups and to examine in mothers from ethnic minority groups whether indicators of acculturation (generational status, language spoken at home, and length of residency in the UK) were associated with these health behaviours.	England.	Quantitative: a prospective nationally representative cohort study.	Not specified.	Not specified.	Quantitative: cohort study.	Stratified clustered sampling framework to over-represent mothers from ethnic minority groups and disadvantaged areas produced 6478 white British or Irish mothers and 2110 mothers from ethnic minority groups. Of those from ethnic minority groups, 681 (33%) were first generation and 55 (4%) second generation.
90	To explore and synthesise the maternity care experiences of female asylum seekers and refugees.	UK.	Qualitative: multiple exploratory longitudinal case studies that used a series of interviews, photographs taken by the women, field notes, and observational methods to contextualise data obtained during 2002 and 2003.	Theory of interactions and transformational educational theory.	Hospital settings or women's homes.	Qualitative: thematical analysis.	Women were approached if the status of 'asylum seeker' or 'refugee' was written in the hospital notes taken at their booking appointment. Fourteen women were approached, but nine women declined to participate. Five women consented, but one woman was dispersed before 20 weeks gestation and therefore was not included in the study. Of the remaining four participating women, three were asylum seekers and one was a refugee. The sampling technique was not clearly reported.

91	To identify key features of communication across antenatal care and whether they are evaluated positively or negatively by service users.	Central London.	Qualitative: used six focus groups of 15 participants each and conducted 15 semi-structured interviews. Non-English-speaking focus groups and interviews were conducted in standard Bengali, Sylheti, or Somali.	Not specified.	Focus groups: hospitals and university meeting rooms. Semi-structured interviews: various locations to suit the needs of the women.	Qualitative: thematical analysis.	The sampling technique was not clearly reported, but they recruited 30 pregnant women from diverse social and ethnic backgrounds affiliated with one NHS Trust (i.e., hospital) in central London. Participants were recruited within this hospital, in eight community antenatal clinics situated in socially and ethnically diverse areas, via a community parenting group for Somali women, and via a Bengali Women's Health Project. Within the hospital, participants were recruited from the antenatal waiting room (which services low- and high-risk pregnancies), the ultrasound clinic, and the glucose tolerance testing clinic.
111	To determine the pregnancy outcomes of women of similar parity and ethnic background who received antenatal care ('booked') compared those who did not ('unbooked') over a period of 18 months.	North Middlesex University Hospital (NMUH), London.	Quantitative: a retrospective cohort study from September 2006 to March 2008 comparing the socio-demographics and the foetal and maternal outcomes of pregnancies of unbooked versus booked women.	Not specified.	Not specified.	Quantitative: a retrospective cohort study.	Women who received no antenatal care or who delivered within 3 days of their initial booking visit were categorised as 'unbooked'. In each case, the woman who had delivered next on the labour ward register (matched for ethnicity and parity) and who had received antenatal care prior to the second trimester served as a comparison.
117	To explore the perspectives of first- and second-generation women of Pakistani origin on maternity care and to make recommendations for culturally appropriate support and care from maternity services.	West Midlands.	Mixed methods: a retrospective Q methodology study of Pakistani women following childbirth.	Retrospective Q method study.	Not specified.	Qualitative: Q methodology.	A purposive sampling strategy was used. Postnatal first- and second-generation Pakistani women were self-identified by their responses to information leaflets disseminated at local Children's Centres across an inner city in the West Midlands.

92	To study the relationships between Somali women and their Western obstetric care providers. The attitudes, perceptions, beliefs, and experiences of both groups were explored in relation to caesarean sections, particularly to identify factors that might lead to adverse obstetric outcomes.	Greater London.	Qualitative: in-depth individual and focus group interviews.	Framework of naturalistic enquiry, emic/etic model	Not specified.	Qualitative: emic/etic analysis.	Selected 39 Somali women by snowball sampling, 36 from the community and three purposively from a hospital.
93	To address the postulates that immigrant women experience sensitive care through the use of an ethnically congruent interpreter and that such women prefer to meet health providers of the same ethnic and gender profile when in a multi-ethnic obstetrics care setting.	Greater London.	Qualitative: in-depth individual and focus group interviews. Open-ended questions were presented by an obstetrician and an anthropologist.	Framework of naturalistic enquiry.	Not specified.	Qualitative: naturalistic inquiry.	Participants were recruited throughout Greater London between 2005 and 2006. Snowball sampling was used to recruit 36 immigrant Somali women, and another three were selected by a by purposive technique for a total of 39. A purposive technique was used to select further 11 Ghanaian women who had delivered at least one child within the British healthcare system and who were living within the study area at the time of data collection.

118	To evaluate a pilot mental health service for asylum-seeking mothers and babies.	UK (not clear).	Mixed methods: evaluation within a participatory action research framework.	Participatory action research framework.	Not specified.	Qualitative: thematical analysis. Quantitative: the CARE-Index.	An active outreach recruitment strategy was adopted by psychologists, who embedded themselves in a drop-in community group, the Merseyside Refugee & Asylum Seekers & Asylum Seekers Pre & Postnatal Support Group. Participants were West African women who were asylum seekers or refugee and who were either pregnant or had a young baby. They originated from The Gambia, Sierra Leone, Ivory Coast, and Nigeria. All spoke English. Their ages ranged from 17 to 32 years, and all babies were under 6 months of age at the point of initial contact, with three babies not yet born. Attendance at the 21 therapeutic group sessions ranged between 4 and 12 mothers (with their babies). Seven mothers attended a significant proportion or all group sessions. An additional six mothers attended 1-4 group sessions.
94	To apply the 'three delays' framework (developed for low-income African contexts) to a high-income Western scenario to identify delay-causing influences in the pathway to optimal facility treatment.	Greater London.	Qualitative: individual and focus group interviews.	'Three delays' framework.	Not specified.	Constructivist hermeneutic naturalistic study.	Purposive and snowball sampling was used to recruit 54 immigrant women originally from sub-Saharan regions in Africa (Somalia, Ghana, Nigeria, Senegal, and Eritrea) living in London and to recruit 32 maternal providers.

112	To identify predictors of late initiation of antenatal care within an ethnically diverse cohort.	Newham, East London.	Quantitative: a cross-sectional analysis of routinely collected electronic patient records from Newham University Hospital NHS Trust (NUHT).	Not specified.	Not specified.	Quantitative: cross-sectional analysis.	All women who attended their antenatal booking appointment within NUHT between 1st January 2008 and 24th January 2011 were included in this study. The main outcome measure was late antenatal booking, defined as attendance at the antenatal booking appointment after 12 weeks (+6 days) gestation. The sample included women from Somalia, Eastern Europe, Africa, the Caribbean, and South Asia.
87	To explore BME women's experiences of contemporary maternity care in England.	All over England.	Qualitative data collected from a large cross-sectional survey using three open-ended questions that encouraged participants to articulate their experience of maternity care in their own words.	Not specified.	Not specified.	Qualitative: Thematic analysis.	A random sample of 4800 women was selected using Office for National Statistics (ONS) birth registration records. The overall response rate was 63% but was only 3% from BME groups. A total of 368 women self-identified as coming from BME groups. Of those, 219 (60%) responded with open text and 132 (60%) were born outside the UK.
95	To investigate women's experiences of dispersal in pregnancy and to explore the effects of dispersal on the health and maternity care of women asylum seekers who were dispersed during pregnancy in the light of NICE guidelines on antenatal, intrapartum, and postnatal care.	London, South of England, Midlands and East of England, North West, North East, and Wales.	Qualitative: interviews were conducted with 19 women who had been dispersed during their pregnancies and with one woman kept in an Initial Accommodation Centre under a new Home Office pregnancy and dispersal guidance issued in 2012.	Not specified.	Not specified.	Qualitative (not clear).	The sampling technique was not mentioned clearly. The women interviewed came from 14 different countries and had been dispersed or relocated to or within six regions of the UK. At the time of dispersal, 14 had been awaiting a decision on their asylum claim and six had been refused asylum.

[113]	To compare the maternal and birth outcomes of Polish and Scottish women having babies in Scotland and to describe any differences in clinical profiles and service use associated with migration from Poland.	All over Scotland.	Quantitative: a population-based epidemiological study of linked maternal country of birth, maternity, and birth outcomes. Scottish maternity and neonatal records linked to birth registrations were analysed for differences in modes of delivery and pregnancy outcomes between Polish migrants and Scots. These outcomes were also compared with Polish Health Fund and survey data.	Not specified.	Not specified.	Quantitative: statistical analysis.	The study analysed 119,698 Scottish and 3105 Polish births to primiparous women in Scotland in 2004-09 using routinely collected administrative data on maternal country of birth and birth outcome.
[96]	To understand the multiple influences on behaviour and hence the risks to metabolic health of South Asian mothers and their unborn children, to theorise how these influences interact and build over time, and to inform the design of culturally congruent, multi-level interventions.	London boroughs, Tower Hamlets, and Newham.	Qualitative: group story-sharing sessions and individual biographical life-narrative interviews.	Multi-level ecological models.	All but four interviews were in the participants' homes.	Qualitative: phenomenology.	The study recruited from diabetes and antenatal services in two deprived London boroughs 45 women of Bangladeshi, Indian, Sri Lankan, or Pakistani origin aged 21–45 years with histories of diabetes in pregnancy. Overall, 17 women shared their experiences of diabetes, pregnancy, and health services in group discussions, and 28 women gave individual narrative interviews (facilitated by multilingual researchers). All were audiotaped, translated, and transcribed.

9	To understand the nature of need in superdiverse areas and to examine the emergent challenges for effective maternity service delivery in an era of superdiversity.	West Midlands.	Mixed methods: the study used a semi-structured questionnaire and held narrative interviews of newcomer women. The findings were then triangulated with interviews of professionals who regularly worked with such women.	Not specified.	Not specified.	Qualitative: systematic thematic analysis. Quantitative: triangulation of findings.	Sampling was not described clearly. However, the study used a semi-structured questionnaire that was designed in collaboration with maternity professionals and community researchers to explore the views and maternity experiences of newcomer women. Experienced multilingual female community researchers completed 82 of these questionnaires with interviewees in a range of different languages. Narrative interviews were also held with 13 women to further explore issues. The findings were triangulated with 18 interviews of professionals who regularly worked with migrant women.
98	To explore how Somali women with FGM experienced and perceived antenatal and intrapartum care in England.	Birmingham.	Qualitative: a descriptive, exploratory study using face-to-face semi-structured interviews that were audio-recorded.	Not specified.	Private room.	Qualitative: thematical analysis.	The study used convenience and snowball sampling of ten Somali women in Birmingham who had received antenatal care in England in the past 5 years.
100	To explore differences in infant thermal care beliefs between mothers of South Asian and white British origin in Bradford, UK.	Bradford District, West Yorkshire.	Mixed methods: mothers were interviewed using a questionnaire with structured and unstructured questions.	Not specified.	The women chose the location of the interview.	Qualitative: thematical analysis.	A total of 102 mothers (51 South Asian and 51 white British) were recruited in Bradford District, West Yorkshire, UK. The inclusion criteria specified infants aged 13 months or less with a parent of South Asian or white British cultural origin who lived in the Bradford District. South Asia was defined as including the countries of Pakistan, India, Afghanistan, Sri Lanka and Nepal. Recruitment was aided by local community organisations, children's centres, and community contacts. Urdu- and Punjabi-speaking interpreters were requested and provided for 69 per cent of the first-generation South Asian mothers (n = 26) in the sample.

97	To gain an understanding of infant feeding practices among a group of UK-based refugee mothers.	Liverpool and Manchester.	Qualitative: two focus group discussions and 15 semi-structured interviews.	Not specified.	HCPs: private offices or clinics Refugee women: private rooms or discrete areas at the support venue (community centre or church hall).	Qualitative: thematical analysis.	The study purposively selected 30 refugee mothers from 19 countries who now resided in Liverpool or Manchester and were at least 6 months pregnant or had a child who had been born in the UK in the last 4 years. Of these 30, 19 were HIV-negative and 11 were HIV-positive.
119	To provide insights into possible causes of poor maternity outcomes for new migrants in the West Midlands region of the UK and to develop recommendations that could help improve maternity services for these migrants.	West Midlands.	Mixed methods: a semi-structured questionnaire and in-depth interviews.	Not specified.	Not specified.	Qualitative: systematic thematical approach. Quantitative: triangulation of the findings.	A non-probability purposive sample was generated by selecting 82 women who had moved to the UK within the past 5 years and had subsequently utilised maternity services. Of these, 13 underwent in-depth interviews as well.
99	To explore the maternity care experiences of pregnant asylum-seeking women in West Yorkshire to inform service development.	West Yorkshire.	Qualitative: interpretative approach within the tradition of hermeneutic phenomenology.	Not specified.	Not specified.	Qualitative: interpretive approach with hermeneutic phenomenology analysis.	Purposive sampling was performed through the voluntary sector and a children's centre. In addition, word-of mouth led to an element of snowball sampling. Six women were recruited.
120	To provide locally applicable data on the needs of Black and minority ethnic women in relation to their uptake of maternity and neonatal care provision by primary healthcare teams in Leeds.	Leeds.	Mixed methods: questionnaires and focus groups. Interpreters were used when necessary for data collection. A questionnaire was translated into Urdu for some women.	Not specified.	Local community centres and in the participants' homes.	Qualitative: content analysis. Quantitative: survey (not clear).	A total of 97 questionnaires were completed, of which 50 were completed through informal links at community centres, schools, and in women homes. The remaining 47 were completed whilst the researcher attended various antenatal clinics in the community.

101	To study the effectiveness of three linkworker and advocacy schemes that were designed to empower minority ethnic community users of maternity services.	Birmingham.	Qualitative: focus group discussions, semi-structured interviews, and non-directive interviews.	Not specified.	Antenatal clinics in hospitals and health centres, community group settings, and participants' homes.	Qualitative: not clear, thematical analysis?	Individual interviews were conducted with 66 Asian women who had received support from linkworker and advocacy services during their pregnancy and postnatally. Of these, 28 were from Birmingham, 13 from Leeds, and 25 from Wandsworth-London. A semi-structured interview guide was translated into five Asian languages: Hindi, Punjabi, Gujarati, Urdu and Tamil. The study also included ten focus groups made up of 60 women who had not used linkworker or advocacy services. All participants were recruited with the help of various minority ethnic women's groups and community organisations. Interpreters assisted 11 personal interviews with non-users from Vietnamese and Chinese backgrounds.
62	To determine the nature of the barriers confronting women when they used antenatal and postnatal services.	Pollokshields, Glasgow.	Qualitative: semi-structured questionnaire.	Not specified.	Not specified.	Qualitative: thematic analysis.	Twenty women were interviewed in depth by a Centre's Health Development Worker. Of these, 17 were born outside the UK.
102	To study the maternity services experiences of Muslim parents in England.	UK: not specified.	Qualitative: focus groups with Muslim mothers to explore their experiences of and views about maternity services; questionnaires with Muslim fathers; and interviews with health professionals	Not specified.	Not specified.	Qualitative: content analysis.	A mixed sample of 43 immigrants and non-immigrants were recruited via their project advisory groups. The focus groups were conducted in various locations around the UK, with two focus group discussions in a language other than English. A total of eight health professionals were interviewed: six midwives (two of whom worked for Sure Start programmes), a health visitor, and a consultant obstetrician.

[114]		To determine the current clinical practice of maternity care in England, including the service provision and organisations that underpin care, from the perspective of women needing the care; to identify the key areas of concern for women receiving maternity care in England; and to determine whether and in what ways women's experiences and perceptions of care have changed over the last 10 years.	England: not specified.	Quantitative: survey.	Not specified.	Survey: not specified.	Quantitative: cross-sectional design.	Random samples of women selected for the pilot and main studies were identified by staff at the ONS using live birth registrations for births within 2 specific weeks: 2–8 January (pilot) and 4–10 March 2006 (main study). The same method of sampling was used as had been employed in 1995 to enable direct comparisons. Random samples of 400 women for the pilot survey and 4800 women for the main survey who were aged 16 years and over and who had delivered their baby in a one week period in England were selected. The sampling was stratified on the basis of births in different geographical areas (Government Office Regions (GORs)). No subgroups were oversampled. The usable response rate was 60% for the pilot survey and 63% for the main survey. The samples included 229 women of BME born outside the UK.

103	To explore the perceptions of pregnant asylum seekers in relation to the provision of their maternity care while in emergency accommodation in the UK.	South East of England.	Qualitative: an exploratory approach using unstructured interviews with five healthcare professionals and semi-structured interviews with ten pregnant asylum seekers.	Not specified.	Participants' emergency accommodatio ns.	Qualitative: thematical analysis.	Purposive sampling of those providing maternity care for asylum seekers produced a sample comprising two midwives (M1 and M2), one GP (GP), one hospital consultant (C), and one nurse (N), all based in south coast health centres and hospitals. A total of 15 pregnant asylum seekers were approached to participate in the study. These women entered the UK through a south coast port over a three-month period. Their countries of origin were Algeria, Congo, Angola, Nigeria, Somalia, and Iraq, and they spoke French, Portuguese, Yoruba, Arabic, and Kurdish. Translated information letters and consent forms were distributed to pregnant asylum seekers via the Refugee Help Line, which also returned signed consent forms. This constitutes non-probability, purposive sampling.
104	To explore the meanings attributed by migrant Arab Muslim women to their experiences of childbirth in the UK. In particular, to explore migrant Arab Muslim women's experiences of maternity services in the UK; to examine the traditional childbearing beliefs and practices of Arab Muslim society; and to suggest ways to provide culturally sensitive care for this group of women.	UK: not specified.	Qualitative: an interpretive ontological-phenomenological perspective informed by the philosophical tenets of Heidegger (1927/1962).	Heideggerian hermeneutic phenomenology.	All interviews were in the participants' homes except for one, which took place in a restaurant after 10 pm.	Qualitative: thematical analysis.	Purposive sampling produced eight Arab Muslim women who had migrated to one multicultural city in the Midlands.

105	To examine the health-seeking behaviours of Korean migrant women living in the UK.	London.	Qualitative: 21 semi-structured interviews.	Foucauldian approach.	Not clear.	Qualitative: not clear.	Women were recruited from New Malden via Korean community contacts.
121	To explore perinatal clinical indicators and experiences of postnatal care among European and Middle Eastern migrant women and to compare them with those of British women at a tertiary hospital in the North East of Scotland.	North East of Scotland.	Mixed methods. Phase 1 of the research was a secondary analysis of routine data for 15,030 consecutive deliveries at Aberdeen Maternity Hospital. Phase 2 was a retrospective study of 26 European, Middle Eastern, and British mothers in this hospital. After the women had given birth, verbal data was collected using face-to-face semi-structured interviews.	Not clear.	Phase Two: 24 interviews were conducted in the homes of participants and two interviews at the University department.	Qualitative: thematical analysis. Quantitative: Phase One was a secondary analysis of routine data for 15,030 consecutive deliveries at Aberdeen Maternity Hospital. Phase 2 was a retrospective study of women.	Phase 1: The 15,030 deliveries included all births at Aberdeen Maternity Hospital over the financial years 2004–2008 in which maternal nationalities were identified and gestation was ≥ 24 weeks. Both singleton and multiple births were included. The clinical data was harvested from the Patient Administration System and the PROTOS maternity information system. In the case of women with multiple order births during the study, all births were included. Phase 2 of the research was a retrospective study of a few of the mothers who had given birth at this hospital. Eight European and five Middle Eastern women were semi-matched with 13 British women.

122	To assess the mechanisms of support available to EM (ethnic minority) communities from community and voluntary sector organisations in relation to maternal and infant nutrition (a mapping exercise); to explore the experiences of the targeted client groups in seeking and receiving such support; and to identify gaps and opportunities to enhance support mechanisms and engagement with diverse EM communities.	Glasgow, Edinburgh, Aberdeen, Stirling, Fife, Dundee, and Inverness.	Mixed methods: an online questionnaire survey of organisations working with EM communities, focus groups, and telephone interviews with EM women.	Not specified.	Not specified.	Qualitative: thematical analysis. Quantitative:	The study identified 65 community organisations that potentially provided food and health services across EM communities in Scotland. In total, 37 organisations replied to the survey. Of those organisations, 15 indicated that they are providing services in the area of maternal and infant nutrition. A further 12 indicated that despite working with EM communities, they do not provide services in maternal and infant nutrition or healthy eating in general. An additional ten organisations confirmed by telephone that they were or had been working with EM women, but were unable to undertake the survey. The majority of interviewees for the focus groups and interviews were selected in response to a request sent by Black and Ethnic Minorities Infrastructure in Scotland (BEMIS) to community organisations. Snowball sampling was used to provide further contacts. In total, four focus groups were conducted with Polish, Roma, Czech, and African mothers. In addition, six telephone interviews were conducted with Polish mothers. We focused on Polish mothers because they were the largest new ethnic group in Scotland since 2004.

106	To explore the experiences of obstetric care in Scotland among women who have undergone FGM.	Glasgow and Edinburgh.	Qualitative: personal experiences of FGM and interviews.	Interpretivism paradigm and feminist perspective.	The Dignity Alert & Research Forum (DARF) office or in the participant's home.	Qualitative: thematical analysis.	Convenience and purposive sampling resulted in a total number of seven women taking part in this study. All women were of African origin living in Scotland (three in Glasgow and four in Edinburgh). The inclusion criteria for the study were women who have undergone FGM and had experienced childbirth in Scotland. Three women were originally from Somalia, two from The Gambia, one from Ghana, and one from Sudan. Six of them were Muslims and one was Christian. All women had undergone FGM in their countries of origin. Four women had been infibulated and the remaining three could not tell if they have had FGM type 2 or 3.
107	To gain a rich understanding of migrant Pakistani Muslim women's experiences of postnatal depression within motherhood; to inform clinical practice; and to suggest ways of improving supportive services for this group.	East London.	Qualitative: interpretative phenomenology.	Interpretative phenomenological analysis (IPA) theory.	Not specified.	Qualitative: interpretative phenomenology.	Purposive sampling resulted in the recruitment of four migrant Pakistani Muslim women from London aged from 27 to 39.
41	To explore the healthcare experience of vulnerable pregnant migrant women.	London.	Mixed methods: participants were contacted by phone (using a three-way interpreter call if appropriate) and interviewed using a pro forma questionnaire designed to determine their access to antenatal care; barriers to that access; and their experiences during pregnancy, labour, and the immediate postnatal period. Further data was extracted from their records at the Doctors of the	Not specified.	Phone survey.	Qualitative: thematical analysis. Quantitative: not clear.	Pregnant women who presented to the drop-in clinic of the DOTW in London were approached between January 2013 and June 2014.

			World (DOTW) clinic to see how they had accessed the clinic.				
108	To explore relationships between first-generation migrant Pakistani women and midwives in the South Wales region, focusing on the factors that contribute to these relationships and the ways that these factors might affect the women's experiences of care.	South Wales.	Qualitative: a focused ethnography.	Symbolic interactionism.	Midwives: at lunch break or between clinics. Pakistani women: not clear.	Qualitative: thematical analysis.	Purposive sampling, through midwife gatekeepers, was selected for the initial recruitment of pregnant migrant Pakistani women: emails were sent to all midwives working with migrant women in South Wales. Snowballing was then used to recruit other midwives eligible for participation. Focused, non-participant observations of antenatal booking appointments took place in antenatal clinics across the local health board region over a period of 3-6 months. A total of seven midwives and 15 women were observed during these appointments, which lasted 20-60 minutes each.

File 6: Characteristics of study participants

Participants country of origins

Studies	Country of origins
Duff, L. A., Ahmed, L. B., & Lamping, D. L. (2002).	Bangladesh
Ahmed, S., Macfarlane, A., Naylor, J., & Hastings, J. (2006).	Bangladesh
Cross-Sudworth, F., Williams, A., & Herron-Marx, S. (2011).	Pakistan
Greenhalgh,T., Clinch, M., Afsar, N., Choudhury, Y., Sudra, R., Campbell-Richards, D., & Finer, S. (2015).	Bangladesh, Indian, Sri Lanka, Pakistan
de Chavez, A. C., Ball, H. L., & Ward-Platt, M. (2016).	South Asia including Pakistan, India, Afghanistan, Sri Lanka, Nepal
Hicks, C., & Hayes, L. (1991).	Asian Sub-Continent
Harper Bulman, K., & McCourt, C. (2002).	Somalia
Essen et al. (2011).	Somalia
Moxey, J. M. &Jones, L.L. (2016).	Somalia
Raine, R., Cartwright, M., Richens, Y., Mahamed, Z., & Smith, D. (2010).	Somalia and Bengal
Binder, P., Borne, Y., Johnsdotter, S., & Essen, B. (2012).	Somalia and Ghana
Cresswell, J. A., Yu, G., Hatherall, B., Morris, J., Jamal, F., Harden, A., & Renton, A. (2013).	Somalia, Eastern European, African, Caribeean, South Asia
Binder, P., Johnsdotter, S., & Essen, B. (2012).	Somalia, Ghana, Nigeria, Eritrea and Senegal
O'Shaughnessy, R., Nelki, J., Chiumento, A., Hassan, A., & Rahman, A. (2012).	Gambia, Sierre Leone, Ivory Coast and Nigeria
McLeish, J. (2005).	Black African origin, other not specified

Gorman, D. R., Katikireddi, S. V., Morris, C., Chalmers, J. W. T., Sim, J., Szamotulska, K., . . . Hughes, R. G. (2014).	Poland
Rowe, R. E., Magee, H., Quigley, M. A., Heron, P., Askham, J., & Brocklehurst, P. (2008).	Not specified
Hawkins, S. S., Lamb, K., Cole, T. J., & Law, C. (2008).	Not specified
Briscoe, L., & Lavender, T. (2009).	Not specified
Tucker, A., Ogutu, D., Yoong, W., Nauta, M., & Fakokunde, A. (2010).	Not specified
Jomeen, J., & Redshaw, M. (2013).	Not specified
Feldman, R. (2014).	Not specified
Phillimore, J. (2015).	Not specified
Hufton, E., & Raven, J. (2016).	Not specified
Phillimore, J. (2016).	Not specified
Lephard, E., & Haith-Cooper, M. (2016).	Not specified
**Grey literature**	
Goodwin, L. (2016).	Pakistan
Lamba, R. (2015).	Pakistan-muslim
Bawadi, H. (2009).	Arab muslims
Lee, Jeung Yeon (2010).	Korean
BEMIS SCOTLAND in partnership with Community Food and Health (Scotland). (2013).	Poland, Roma, Czech and African
Almalik, M. (2011).	Europe and Middle East
Baldeh, F. (2013).	Somalia, Gambia, Ghana, Sudan
Nabb, J. (2006).	Algeria, Congo, Angola, Nigeria, Somalia and Iraq
Leeds Family Health. (1992).	Not specified

Warrier, S. (1996).	Asian
Pershad, P., Tyrrell, H. (1995).	Not specified
Ali, N. (2014).	Not specified
Redshaw et al. (2006).	Not specified
Shortall, C., et al. (2015).	Not specified

Antenatal & postnatal

**PEER REVIEWED**	
**Antenatal**	
Rowe, R. E., Magee, H., Quigley, M. A., Heron, P., Askham, J., & Brocklehurst, P. (2008).	Antenatal
Raine, R., Cartwright, M., Richens, Y., Mahamed, Z., & Smith, D. (2010).	Antenatal
Binder, P., Johnsdotter, S., & Essen, B. (2012).	Antenatal
Cresswell, J. A., Yu, G., Hatherall, B., Morris, J., Jamal, F., Harden, A., & Renton, A. (2013).	Antenatal
Feldman, R. (2014).	Antenatal
Greenhalgh,T., Clinch, M., Afsar, N., Choudhury,Y., Sudra, R., Campbell-Richards, D. & Finer, S. (2015).	Antenatal
Moxey, J. M. & Jones, L.L. (2016).	Antenatal
**Ante, intrapartum & postnatal**	
Cross-Sudworth, F., Williams, A., & Herron-Marx, S. (2011).	Ante, intrapartum & postnatal
Duff, L. A., Ahmed, L. B., & Lamping, D. L. (2002).	Ante, intrapartum & postnatal
McLeish, J. (2005).	Ante, intrapartum & postnatal
Lephard, E., & Haith-Cooper, M. (2016).	Ante, intrapartum & postnatal

Harper Bulman, K., & McCourt, C. (2002).	Ante, intrapartum & postnatal
Briscoe, L., & Lavender, T. (2009).	Ante, intrapartum & postnatal
Phillimore, J. (2015).	Ante, intrapartum & postnatal
Phillimore, J. (2016).	Ante, intrapartum & postnatal
Essen & al. (2011).	Ante, intrapartum & postnatal
**Postnatal**	
O'Shaughnessy, R., Nelki, J., Chiumento, A., Hassan, A., & Rahman, A. (2012).	Postnatal
Gorman, D. R., Katikireddi, S. V., Morris, C., Chalmers, J. W. T., Sim, J., Szamotulska, K., . . . Hughes, R. G. (2014).	Postnatal
de Chavez, A. C., Ball, H. L., & Ward-Platt, M. (2016).	Postnatal
Ahmed, S., Macfarlane, A., Naylor, J., & Hastings, J. (2006).	Postnatal
Hufton, E., & Raven, J. (2016).	postnatal
**Intrapartum & postnatal**	
Jomeen, J., & Redshaw, M. (2013).	Intrapartum & postnatal
**Antenatal & postnatal**	
Tucker, A., Ogutu, D., Yoong, W., Nauta, M., & Fakokunde, A. (2010).	Antenatal & postnatal
**Not clear**	
Hicks, C., & Hayes, L. (1991).	Not clear

GREY LITERATURE	
**Antenatal**	
Leeds Family Health. (1992).	Antenatal
Goodwin, L. (2016).	Antenatal
**Ante, intrapartum & postnatal**	
Ali, N. (2004).	Ante, intrapartum & postnatal
Bawadi, H. (2009)	Ante, intrapartum & postnatal
Lee, Jeung Yeon (2010).	Ante, intrapartum & postnatal
Baldeh, F. (2013).	Ante, intrapartum & postnatal
Shortall, C., et al (2015).	Ante, intrapartum & postnatal
BEMIS SCOTLAND in partnership with Community Food and Health (Scotland). (2013).	Ante, intrapartum & postnatal
Warrier, S. (1996)	Ante, intrapartum & postnatal
Redshaw et al. (2006).	Ante, intrapartum & postnatal
**Ante & postnatal**	
Almalik, M. (2011).	Ante & postnatal
**Postnatal**	
Lamba, R. (2015)	postnatal

Immigrant category

PEER REVIEWED	
**Refugees**	
Harper Bulman, K., & McCourt, C. (2002).	refugees
Binder, P., Borne, Y., Johnsdotter, S., & Essen, B. (2012).	refugees

Moxey, J. M. & Jones, L.L. (2016).	refugees
Hufton, E., & Raven, J. (2016).	refugees
**Asylum seekers**	
Feldman, R. (2014).	Asylum seekers
Lephard, E., & Haith-Cooper, M. (2016).	Asylum seekers
O'Shaughnessy, R., Nelki, J., Chiumento, A., Hassan, A., & Rahman, A. (2012).	Asylum seekers
McLeish, J. (2005).	Asylum seekers
**Immigrant category not clear**	
Duff, L. A., Ahmed, L. B., & Lamping, D. L. (2002).	Immigrant category not clear
Ahmed, S., Macfarlane, A., Naylor, J., & Hastings, J. (2006).	Immigrant category not clear
Rowe, R. E., Magee, H., Quigley, M. A., Heron, P., Askham, J., & Brocklehurst, P. (2008).	Immigrant category not clear
Hawkins, S. S., Lamb, K., Cole, T. J., & Law, C. (2008).	Immigrant category not clear
Tucker, A., Ogutu, D., Yoong, W., Nauta, M., & Fakokunde, A. (2010).	Immigrant category not clear
Cross-Sudworth, F., Williams, A., & Herron-Marx, S. (2011).	Immigrant category not clear
Raine, R., Cartwright, M., Richens, Y., Mahamed, Z., & Smith, D. (2010).	Immigrant category not clear
Jomeen, J., & Redshaw, M. (2013).	Immigrant category not clear
Gorman, D. R., Katikireddi, S. V., Morris, C., Chalmers, J. W. T., Sim, J., Szamotulska, K., . . . Hughes, R. G. (2014).	Immigrant category not clear
Greenhalgh,T.,Clinch, M., Afsar, N., Choudhury,Y., Sudra, R., Campbell-Richards, D., & Finer, S. (2015).	Immigrant category not clear
de Chavez, A. C., Ball, H. L., & Ward-Platt, M. (2016)	Immigrant category not clear

Hicks, C., & Hayes, L. (1991).	Immigrant category not clear
Binder, P., Johnsdotter, S., & Essen, B. (2012).	Immigrant category not clear
**Asylum seekers and refugees**	
Briscoe, L., & Lavender, T. (2009).	Asylum seekers and refugees
**Mixed migrant categories**	
Cresswell, J. A., Yu, G., Hatherall, B., Morris, J., Jamal, F., Harden, A., & Renton, A. (2013)	Mixed migrants categories
Phillimore, J. (2016).	Mixed migrant categories
Phillimore, J. (2015).	Mixed migrant categories
Essen, & al. (2011).	Mixed migrant categories
