# Supplementary material for: Experience of and access to maternity care in the UK by immigrant women: a narrative synthesis systematic review
Source: BMJ Open. 2019 Dec 31;9(12):e029478. doi: 10.1136/bmjopen-2019-029478 (PMC6955508; doi:10.1136/bmjopen-2019-029478)
Supplement: Author's manuscript [file bmjopen-2019-029478.draft_revisions.pdf]

BMJ Open

BMJ Open is committed to open peer review. As part of this commitment we make the peer review history of every article we publish publicly available.

When an article is published we post the peer reviewers' comments and the authors' responses online. We also post the versions of the paper that were used during peer review. These are the versions that the peer review comments apply to.

The versions of the paper that follow are the versions that were submitted during the peer review process. They are not the versions of record or the final published versions. They should not be cited or distributed as the published version of this manuscript.

BMJ Open is an open access journal and the full, final, typeset and author-corrected version of record of the manuscript is available on our site with no access controls, subscription charges or pay-per-view fees (http://bmjopen.bmj.com).

If you have any questions on BMJ Open's open peer review process please email info.bmjopen@bmj.com

BMJ Open

Immigrant women's experience of and access to maternity care in the United Kingdom (UK): a narrative synthesis systematic review

Journal:	*BMJ Open*
Manuscript ID	bmjopen-2019-029478
Article Type:	Research
Date Submitted by the Author:	30-Jan-2019

Complete List of Authors:	Higginbottom, Gina; University of Nottingham School of Health Sciences, School of Health Sciences<br>Evans, Catrin; University of Nottingham School of Health Sciences, School of Health Sciences<br>Morgan, Myfanwy; King's College London, Institute of Pharmaceutical Science<br>Bharj , Kuldip; University of Leeds, UK, Faculty of Medicine and Health<br>Eldridge, Jeanette; University of Nottingham School of Health Sciences, Research and Learning Services,<br>Hussain, Basharat; University of Nottingham School of Health Sciences,

Keywords:	systematic review, narrative synthesis, immigrant women, maternity care, navigation and access

SCHOLARONE™
Manuscripts

Final

Immigrant women's experience and access to maternity care in the United Kingdom (UK): a narrative synthesis systematic review

Gina Marie Awoko Higginbottom,[1]* Catrin Evans,[1] Myfanwy Morgan,[2] Kuldip Kaur Bharj,[3] Jeanette Eldridge,[1] Basharat Hussain[1]

[1] Faculty of Medicine and Health Sciences, Queen's Medical Centre, University of Nottingham, Lenton Boulevard, Nottingham, NG7 2HA, UK

[2] Institute of Pharmaceutical Science, Faculty of Life Sciences and Medicine, King's College London, UK

[3] Division of Midwifery, University of Leeds, Leeds, UK

For peer review only

ABSTRACT [300 words]
One in four births in the UK is to foreign-born women. In 2016 the figure was 28.2% the highest figure on record and maternal and perinatal mortality are disproportionately higher. Our objective was to use a narrative synthesis approach to a systematic review of empirical research that focused on access and interventions to improve maternity care for immigrant women.

Review methods
A research librarian designed the literature database search strategies (retrieving literature published from 1990 to end June 2017). We retrieved citations (45,954) and independently screened using a screening tool. We also searched for grey literature reported in related databases and websites. We contacted stakeholders with expertise.

Results
We identified 40 studies for inclusion. Immigrant women tended to book and access antenatal care later than the recommended first 10 weeks. Primary factors included limited English language proficiency, lack of awareness of availability of the services, lack of understanding of the purpose of antenatal appointments, immigration status, and income barriers. Those with positive perceptions said healthcare professionals were caring, confidential, and openly communicative in meeting their medical, emotional, psychological, and social needs. Those with negative views perceived that health professionals had been rude, discriminatory, insensitive to their cultural and social needs. These women therefore avoided accessing or continuously utilising maternity care.
We found few interventions that had focused on improving maternity care for these women and the effectiveness of existing interventions have not been rigorously evaluated

Conclusions
The experiences of immigrant women in accessing and using maternity care services in the UK are mixed, but these women largely had poor experiences. Factors contributing to poor experiences included lack of language support, cultural insensitivity, discrimination, poor relationships between immigrant women and healthcare professionals, and a lack of legal entitlements and guidelines on the provision of welfare support and maternity care to immigrants.

Word Count:300

For peer review only - http://bmjopen.bmj.com/site/about/guidelines.xhtml

Final

STRENGTHS AND LIMITATIONS OF THIS STUDY

Immigration is an international phenomenon and this review increases understanding of how immigrant women navigate maternity services in the UK

The review systematically maps our positive and negative aspects of maternity care provision as experienced by immigrant

The review provides strategic direction for enhancement of maternity care services

The review does not address the experiences of maternity care for second-generation women (e.g. women of black and minority origin born in the UK)

SUPPLEMENTARY FILES

- Original protocol

FUNDING STATEMENT

The NIHR HS&DR Programmed funded this project: The views expressed in this report are those of the authors and not necessarily those of the NHS, the NIHR or the Department of Health and Social Care (15/55/03).

PROSPERO 2015: CRD42015023605 Available from http://www.crd.york.ac.uk/PROSPERO/display_record.asp?ID=CRD420150

Professor Gina Higginbottom, Faculty of Medicine and Health Sciences, Queen's Medical Centre, Nottingham, NG7 2UH, UK
Tel: 0115 82 30991
Email: gawoko@hotmail.com

Declared competing interests of authors: none

Keywords: maternity, immigration, interventions, access to services, systematic review, narrative

For peer review only - http://bmjopen.bmj.com/site/about/guidelines.xhtml

Final

INTRODUCTION

The UK is in a period of superdiversity presenting challenges for the delivery and configuration of health services. That is a The UK is in a period of superdiversity defined as a "distinguished by a dynamic interplay of variables among an increased number of new, small and scattered, multiple-origin, transnationally connected, socio-economically differentiated and legally stratified immigrants"' (Vertovec, 2007, p1024)[1] Equality is a key aim of the NHS in the UK.[2] One in four births in the UK is to foreign-born women [3]. Significantly, immigrant women appear disproportionately in confidential inquiries into maternal and perinatal mortality, indicating possible deficits in the delivery of care, access and utilisation.[4] In order to address these shortcomings a coherent evidence base is required in order to inform services. Our review contributes to this evidence base synthesising knowledge related to maternity care access and interventions. Synthesised evidence is required for knowledge users (policy and practice users) to appropriately configure interventions as per the NHS Midwifery 2020 vision,[5] to guide professional development of healthcare professionals (HCPs), and to reshape care to ensure culturally congruent maternity care. It seems likely that consequentially, enhancements to maternity care for immigrant women will not only benefit these women but also improve the health of future generations in the UK.[3, 4, 6]

Facilitating the provision of appropriate healthcare for immigrant populations in the UK will be crucial for maximising their well-being and their health potential. Without the delivery of culturally appropriate and culturally safe maternal care, negative event trajectories may occur ranging from simple miscommunications to life-threatening incidents, [7-9] risking increased maternal and perinatal mortality. Indeed, immigrant women are overrepresented in mortality statistics. [4] While recent reviews have focused on specific aspects of maternity care, [10, 11] they have not considered a comprehensive conceptualisation of access [12] or the current super diversity. [1, 2] Reconfiguration and redesign of NHS maternal services to meet the needs of immigrant women requires integration of all these aspects.

Considering the global context, some commonality exists between high income nations in the maternity care experiences of immigrant women: studies in the United States, [13] Canada, [11]Australia, [14, 1 5]Sweden,[16, 17] and Germany [8, 18] all provided evidence of this in earlier international reviews led by Higginbottom [7, 19]and Gagnon. [11] However, the international comparative reviews by Gagnon focused on specific populations (South Asian and Somali) women in the UK[11], thus focusing on established immigrant groups are not the more recent super diverse patterns of migration. We have addressed this deficit in our current review.

For peer review only - http://bmjopen.bmj.com/site/about/guidelines.xhtml

Final

CONCEPTUAL DEFINITIONS

There is no consensus definition in the UK regarding the definition of the term 'immigrant' in different data sources and datasets relating to migration. The terms immigrant and migrant are frequently used interchangeably whilst conveying the same meaning. **Country of birth** is used by The Annual Population Survey (APS) of workers and Labour Force Survey (LFS) as a precursor for defining a 'migrant'. This survey therefore declares a person born outside the UK is classified as a 'migrant'. Noteworthy is the fact that workers born outside the UK may become British citizens with increasing residence in the UK.

A second source of data on migrants is applications made for obtaining a National Insurance Number, this defers from the former in definition in that the term migrant is conferred on the on the basis of **nationality**. Meaning all applicants that hold nationality other than the UK are considered migrants. However, the situation is dynamic in that the nationality of a person is may also to change over time and in some individuals may acquire dual-citizenship involving several nation states.

A third and significant source of data on migrants is the Office for National Statistics (ONS). ONS utilise a different strategy of classification focusing on the notion of short-term international migrant and long-term international migrant. In this definition the term 'long-term' refers to holding, the intention of residing longer than a year, whereas short-term is intention of residing less than a year. The implication of this is that the ONS considers **length of stay** of a person in the UK critical in determining migrant status. The United Nations (UN) recommend the classification of migrant into short and long term. Additionally, ONS utilises the UN definition of long- term international migrant. Accordingly, *"a migrant is someone who changes his or her country of usual residence for a period of at least a year, so that the country of destination effectively becomes the country of usual residence.*[20] In long –term international migration data, students and asylum seekers are also included which is not the case in the US.

Immigrants and the UK National Health Service (NHS)

In respect of service provision, the NHS adhere to the mandates set by central government that determines immigrant's entitlement to free NHS care. These mandates are concerned with the immigrant status of and the type of service provision.[21] Within these mandates, an asylum seeker woman may not be entitled to full maternity care because of immigration status.[22] Moreover, data collection with the NHS on this topic is not well established nor comprehensive. Currently the NHS usually collects data on ethnicity and nationality and not on the migration related variables such as length of stay, country of origin etc.

National Institute of Clinical Excellence (NICE) which provides clinical guidelines for healthcare practice in the UK is worth noting here informs healthcare delivery in the UK. NICE (2010).[23] in its guidelines on *Pregnancy and complex social factors: a model for service provision for pregnant women with complex social factors* identified recent migrant women having complex social needs. Within the NICE definition, a recent migrant woman is a woman has who moved to the UK within the previous 12 months. The term migrant is used

For peer review only - http://bmjopen.bmj.com/site/about/guidelines.xhtml

Final

generically conflating migrant women of all classifications e.g. economic migrants, asylum seekers, refugees, and those lacking English language proficiency. This suggests that there is implicit acceptance of the term migrant women in healthcare in respect of being born outside the UK, being subject to immigration regulations, with possible challenges in English language proficiency.

The operational definition of an immigrant women used in this review

The preceding paragraphs suggests that the term 'immigrant' is defined in various ways in different countries and by different authors. However, two features are frequently referred to in these definitions. i.e. 'country of birth' and 'length of stay'. These factors are also noted by NICE guidelines on provision of maternity care. [23] cited above. These two characteristics are also important in the entitlement, access and ability to use healthcare in the UK. For example, if you are born outside the UK, it is likely that you are knowledgeable about the UK healthcare provision.

We adopted the following definition of an immigrant woman for the purposes of our review. moreover, to inform our inclusion and exclusion criteria. A woman is an immigrant if she is:

- Born outside the UK, and;
- Is living in the UK for more than 12 months or had the intention to live in the UK for 12 (or more) months when first entered.

Therefore, we included studies on immigrant women where the population studied fulfils the above two characteristics. According to this definition studies on population groups of foreign students, asylum seekers, recent legal refugees and immigrants, illegal immigrants will also be eligible for inclusion. In many cases, the study populations/sample may not be accurately and fully described. We therefore used linguistic ability e.g. the need for an interpreter as a proxy.

Cy for immigrant status. Notwithstanding all of these perspectives, we acknowledge that the term 'immigrant women' is generic and refers to a highly heterogeneous group of individuals with a complex and vast array of ethno-cultural

Aim and rationale

Review question/s: What interventions exist that are focused specifically on improving maternity care for immigrant women in the UK?

(a) How do these interventions address inequality?

(b) How do accessibility and acceptability manifest, as important dimensions of access to maternity care services as perceived and experienced by immigrant women?

Our review draw upon two broad theoretical concepts first Gulliford theory of access and secondly the concept of cultural safety.

A theory of access to services developed by Gulliford *et al.*[12] map out four dimensions *(Figure 1)*:

1. Service availability

For peer review only - http://bmjopen.bmj.com/site/about/guidelines.xhtml

Final

2. Utilisation of services and barriers to access (which includes personal, financial, and
organisational barriers)
3. Relevance, effectiveness, and access
4. Equity and access

INSERT FIGURE 1

We used this theoretical model in our systematic review, which was based on a synthesis project funded by the National Institute for Health Research (NIHR). Unlike most access models in the United States, this framework reflects the philosophy of the NHS in that its key principles are to provide horizontal access (ensuring equality of access in the population) and vertical access (meeting the needs of particular groups in the population, such as minority ethnic groups). The application of these principles is influenced by availability, accessibility, and acceptability. The Gulliford model has been widely used in empirical research, with the main paper having been cited at least 386 times. With its emphasis on accessibility, acceptability, relevance, and effectiveness, this model is entirely appropriate for assessing the provision of maternity services to minority ethnic groups. In this study, the Theory of Access to assist in the theme development. Following theme development, we established how theory intersected with our evidence.

Secondly, concepts of cultural safety which provided a theoretical lense for the production of recommendations.t Cultural safety is a theory that aims to assist underderstandings of deficits in care by considering the historical and social processes that impact power relationships within and beyond healthcare.[24] Cultural safety is achieved when programmes, instruments, procedures, methods, and actions are implemented in ways that do not harm any members of the culture or ethnocultural group who are the recipients of care. Those within the culture are best placed to know what is or is not safe for their culture, which suggests the need for increased dialogue about immigrant and partner approaches.[25-29] Our protocol is published in BMJ *Open* see https://bmjopen.bmj.com/content/bmjopen/7/7/e016988.full.pdf.

Methods

We used Popay's approach to NS[30], which consists of 4 elements

Element 1: Developing a theory of why and for whom.
Element 2: Developing a preliminary synthesis of the findings of the included studies, following implementation of the search strategy.
Element 3: Exploring relationships in the data.
Element 4: Assessing the robustness of the synthesis.

For peer review only - http://bmjopen.bmj.com/site/about/guidelines.xhtml

Final

There are a number of evolving genres of systematic reviews especially in the of qualitative evidence synthesis from aggregative to interpretive approaches,[31 30] the purpose and selection of a NS approach was to produce highly relevant policy and practice evidence.
The NS approach relies primarily a narrative synthesis of the key findings of studies using text to summarise the findings of the synthesis, which is a result of a synthesis of the *narrative findings of included papers*. GH and

MM have successfully employed this review genre previously and have vast expertise in its usage. [7, 32] NS maybe used with all paradigms of research quantitative, qualitative studies, and mixed methods research studies, as the emphasis is on an interpretive synthesis of the narrative findings of research rather than on a metadata analysis.[30]

Our review had no predefined hypotheses. Our concise review focus of immigrant women's access to maternity care and on inventions in maternity care, this was developed and refined by mapping the available knowledge but still ensuring the relevance of the knowledge synthesis (to policy makers, other potential knowledge users, and immigrant communities). Our focus, included antenatal, labour, and postnatal care; maternal risk factors; health promotion; access to and availability and competency of maternity services; the role of culture and tradition; maternity outcomes in relation to perceived maternal risk factors; and responses to care interventions. We engaged in additional consultation with our Project Advisory Group (PAG) to further refine the review questions to increase their relevancy.

Search strategy refinement and implementation

The comprehensive, exhaustive search strategy was evolved developed in close collaboration with our experienced information scientist. The strategy generated high rates of retrieval of records pertinent to the research question of this project.

The search strategy used key terms used in consistently formulated text-based queries and search statements. These terms were based on subject headings, thesaurus terms, or related indexing and categorisation terms appropriate for each literature database. An example of a detailed final search strategy is given in *Supplementary File 1*. First, we searched 10 electronic databases using the aforementioned strategies. Following this, we searched for appropriate grey literature in SI Web of Knowledge Conference Proceedings Citation Index (Science 1990–), ISI Web of Knowledge Conference Proceedings Citation Index (Social Science and Humanities 1990–), ProQuest Dissertations and Theses, and the Cochrane Methodology Register. We also searched using Google and Google Scholar and consulted with the study expert advisory group. In conclusion we hand searched the reference list of all included studies and relevant systematic reviews. Citations were downloaded into an ENDNOTE library and following this all duplicates removed. The bibliographic databases that we searched are listed in *Supplementary File 2*.

We adopted the PICO approach to implement the search strategy as follows: -
P = immigrant women

For peer review only - http://bmjopen.bmj.com/site/about/guidelines.xhtml

Final

I = maternity care
[C = non-immigrant women – implicit comparator emerging in the results]
O = experience of care

Therefore, our search strategy development was based on:

Search concept 1 = pregnancy, childbirth [implicitly females requiring maternity care], explicit terms covering women/females requiring all types of maternity care [antenatal, perinatal, postnatal, etc.]
Search concept 2 = immigrant populations [which would not fully distinguish between "new" and "second-generation" immigrants – this would be done at the selection stage]
Search concept 3 = terms used to identify access to, use of, deficiencies in, etc., service provision [to help identify groups with poorer health outcomes or vulnerabilities]

Our final answer set of citations included concepts 1 and 2 and 3.

Screening and selection

We included studies that focused on immigrant women and adopted the following definition of an immigrant woman for the purposes of our review. A woman is an immigrant if she is:

- Born outside the UK, and;
- Is living in the UK for more than 12 months or had the intention to live in the UK for 12 (or more) months when she first entered the UK.

We therefore included studies on immigrant women where the population studied fulfils the above two characteristics. According to this definition studies that focused on the following population groups, foreign students, asylum seekers, recent legal refugees and immigrants, illegal immigrants were also eligible for inclusion. In many cases, the study populations/sample was not accurately nor fully described. We therefore used linguistic ability e.g. the need for an interpreter as a *proxy for immigrant status*. Our focus was on first generation immigrant women regardless of their phenotype meaning women of all ethnic groups are included women of *white ethnicities*, although we encountered few studies that focused on the latter and our review was constrained by the lack of various studies in the scientific literature. Study screening was undertaken by two team members independently (GH & BH) assessing the relevance of titles and abstracts in respect of our screening tool. The entire team reviewed ambiguous papers in order to achieve a consensus agreement. Full text papers of potentially included studies were retrieved and appraised. The exclusion and inclusion criteria can be found in *Supplementary File 3*. Excluded papers with the rationale for exclusion can be found in *Supplementary File 4*

Quality assessment

All included studies were critically appraised by two reviewers using tools from the Center for Evidence-Based Management (CEBMa).[33] We used GRAMM [34] for the mixed-methods studies. Differences were resolved in our reflective team meetings. We also used high, medium, and low as appraisal categories. This is approach is congruent with recent

For peer review only - http://bmjopen.bmj.com/site/about/guidelines.xhtml

Final

publications from the Cochrane Qualitative Research Group's CERQUAL publications, as they use this type of evaluation was previously used by Higginbottom et al in published studies.[7, 19] Studies are classified in three domains, high, medium and low to enable a 'macro' evaluation..

- *High* was assigned to studies that used a rigorous and robust scientific approach that largely met all CEBMa benchmarks, perhaps equal to or exceeding 7 out of 10 for qualitative studies, 9 out of 12 for cross-sectional surveys, or 5 out of 6 for mixed-methods research.

- *Medium* was assigned to studies that had some flaws but that did not seriously undermine the quality and scientific value of the research conducted, perhaps scoring 5 or 6 out of 10 for qualitative studies, 6 to 8 out of 12 for cross-sectional surveys, or 4 out of 6 for mixed-methods research.

- *Low* was assigned to studies that had serious or fatal flaws and poor scientific value and scored below the numbers of benchmarks listed above for medium-level appraisals in each type of research

The past decade as witness a growth in the development of new approaches to systematic reviews especially in the domain of qualitative evidence synthesis (QES). Concurrently innovative approaches to assessing quality have evolved. Popay [30] suggests that we do evaluate the 'richness' of studies. Furthermore this research team have utilised this approach in previous funded studies and publications.[7, 32] Popay defined richness as "*the extent to which study findings provide in-depth explanatory insights that are transferable to other settings"*p 230.[30] We used the criterion established in previous studies appraised all the studies in this review using this evaluative tool. Thick' papers create or draw upon theory to provide in-depth explanatory insights that can potentially be transferable to other contexts. By contrast, 'thin' papers provide limited or superficial description and offer little opportunity for generalizing. Each paper was assessed against the criteria as set out in Higginbottom et al, p.5 and categorised as either 'thick' or 'thin' see table 1.

INSERT Table 1:

Data Extraction and assessment of relevance

We conducted the following foundational activities in order to extract data (discussed in detail later).

1) Textual description. A systematic textual narrative was written for each study. We used headings adapted from Popay *et al.*[30] Setting, Participants, Aim, Sampling and Recruitment, Method, Analysis, Results.

For peer review only - http://bmjopen.bmj.com/site/about/guidelines.xhtml

Final

(2) Tabulation and summarisation of all studies to be included. These tables described the attributes of the studies and the results. Information was extracted from the textual description using the same headings as above and additional headings as necessary. Papers in the PDF format were imported into ATLAS.ti qualitative data analysis software (ATLAS.ti Scientific Software Development GmbH, Berlin) using the 'Attributes' option to allow the tabulation of relevant data.

Analysis and synthesis

We produced a *Textual description* of each included studies to facilitate consistency in the comparative processes. We used headings adapted from Popay *et al.*:[30] Setting, Participants, Aim, Sampling and Recruitment, Method, Analysis, Results.

We then proceeded to *Tabulation and collation of all studies to be included.* Papers in the PDF format were imported into ATLAS.ti qualitative data analysis software (ATLAS.ti Scientific Software Development GmbH, Berlin) using the 'Attributes' option to allow the tabulation of relevant data. The analysis created over 250 codes. The two perspectives (tabulation and coding) were then merged to create a cohesive interpretation.

In Element 2 we also applied the critical appraisals tools, made an appraisal and included this in the one-page textual summary we created.

Following construction of the preliminary themes, we produced code/narrative theme tables to demonstrate how the basic meaning units related to the theme. Utilising the codes produced in ATLAS.ti and aligning these to the manually extracted key findings

We used the following concept suggested by Roper & Shapira p.95 [35] in order to interrogate date during the analytical processes.

- ***Setting:*** *the environment or context*
- ***Activities****: patterns of behaviour that occur often*
- ***Events****: rare and infrequent activities*
- ***Relationships and social structures****: kinship, friendship, bonds, enemies, hierarchal*
- ***General perspectives****: the group's shared understandings*
- ***Specific perspectives on the research topic/s*** *how people understand the phenomena*
- ***Strategies:*** *ways of achieving goals*
- ***Process:*** *flow of events how things change over time*
- ***Meaning:*** *significance and understanding of behaviour*
- ***Repeated phases:*** *depictions of thought processes*

We reviewed all these processes in our reflective team meetings to ensure the rigour and robustness of our analytical steps. This iterative process similar to qualitative research involved deconstructing the narrative findings into meaning units and social processes as they

For peer review only - http://bmjopen.bmj.com/site/about/guidelines.xhtml

Final

manifested in maternity care experiences of immigrant women. Individual team members engaged in independent theming of tabular and coded data. We subsequently merged these individual perspectives to form the final harmonised themes representing a '*meta-inference'* in respect of the narrative findings of the included studies. Meta-inference is a term used in mixed methods research to describe merging of findings from the positivistic and the interpretative paradigms as is the case in this NS. Tashakorri and Teddlie p.101 [36] describe meta-inference as "*an overall conclusion, explanation of understanding developed from the integration of inferences obtained from the qualitative and quantitative strands".*

We have constructed the themes in a directive fashion (*meaning containing implicit indications)* in order to provide tangible guidance for policy and practice that might be developed into transformational policy and practice relevant strategies that benefits immigrant women and the NHS.

Rigour, reflexivity and the quality of the synthesis

Reflexivity in the review process requires a self-conscious and explicit acknowledgement of the impact of the researcher on the research processes, interpretations and research products. Reflexivity demands acknowledgement of inherent power dimensions, hierarchies and prevailing ideologies that might shape and determine interpretations and the consequent knowledge production and research products. Gender, sexuality, professional socialisation, ethno-cultural orientation and political lenses as these impact upon social identities further coalescing to provide a specific perspective on any given phenomena. The review team members are imbued with a strong personal and professional commitment to the eradication of inequalities and allegiance to contemporary equality and diversity agendas. From a reflexive perspective, this is important given that immigration is global phenomena and the inherent vulnerability of some immigrant women.
Reflexive analysis alerts us as researchers to emergent themes and informs the formal and systematic process of analysis. Murphy *et al.* p.188[37] define reflexivity as:

> *" we mean sensitivity to the ways in which the researcher's presence in the research setting has contributed to the data collected and their own a priori assumptions have shaped the data analysis"*

The review members hold a deep knowledge of the semantic challenges associated with describing ethno-cultural groups globally recognising that in some socio-political context these terms may be regarded as pejorative in nature. Two team members are immigrants to the UK and two team members are UK citizens who have been immigrants to other countries. We believe our reflexive team meetings, consensus decision making, and incisive debates mitigated these tensions.

For peer review only - http://bmjopen.bmj.com/site/about/guidelines.xhtml

Final

During in reflective team meetings we facilitated dialogue and challenges to primary selection of included papers, emerging interpretations and the extent to which individual subjective lenses had influenced these decisions. The development of themes included an exercise undertaken independently by each team (5 and one project advisory group member). Our collaborative decisions required constant review and reading and, in some cases, reviewing the theme allocation and evidence. Therefore, we believe we achieved a nuanced and comprehensive approach, reaching consensus.

Within the published NS reviews, we have not noted great attention to the issue of publication bias however we strived to eradicate any potential bias by undertaking a comprehensive and exhaustive literature review that included grey literature and follow up emails with authors seeking greater clarity and explanation of opaque issues. A number of the included research studies were identified via *ProQuest* and *E-theses* and do not appear as publications in peer reviewed scientific journals.

We held a national stakeholder event during which we presented our preliminary findings to a wide range of health professions (obstetrician, general practitioner and midwives), academics, voluntary and community workers. Possibly this approach may be considered contentious in the respect of systematic review, as attendees had no previous knowledge of the original included papers, however they held deep topic knowledge. Notwithstanding this, we found broad support for our findings and facilitated groups work activities in order to challenge our initial interpretations. These challenges resulted in the construction of *Theme 5: Discrimination, racism, stereotyping, cultural sensitivity, inaction, and cultural clash in maternity care for immigrant women.* These focused activities collectively contribute to the confidence in the review findings, providing verification and validation of the themes.

Results

The search outcomes are comprehensively detailed in figure 2 the PRISMA flow chart [38]

INSERT FIGURE 2

Studies included in the review, findings, and evidence

Our systematic review using narrative synthesis identified 40 empirical research studies in the scientific and grey literature. A broad range of ethnocultural groups and methodological genres are included in this review (see *Supplementary file 5* for master table of included studies). The distribution of the studies across the themes can be found in figure 3 and publication dates in figure 4.

INSERT FIGURE 3

Methodological genres

For peer review only - http://bmjopen.bmj.com/site/about/guidelines.xhtml

Final

We identified eight quantitative studies that used a questionnaire as a tool for data collection.[39-46] These population-based studies and cohort surveys were all cross-sectional: none were longitudinal.

Mixed-methods studies

We identified ten mixed-methods studies using a range of designs; that is, these studies employed both qualitative and quantitative dimensions.[1, 2, 47-54] For example, Duff *et al.*[48] reported a two-stage psychometric study in which focus groups and interviews were used in the first stage to develop a questionnaire for an ethnocultural group (Sylheti) In the second stage, quantitative methods were used to test and evaluate the acceptability, reliability, and validity of the questionnaire. Other mixed-methods designs included (a) interviewing a small sample of the participants after collecting data from a large-scale survey; (b) conducting semi-structured interviews with a small sample of participants based on quantitative data routinely collected from a large group of participants; and (c) using face-to-face, postal, and online questionnaires to collect data. One of the studies used Q methodology, which uses questionnaires with structured and unstructured questions.

Studies focusing on specific ethnocultural groups

The chosen studies included participants from a wide range of ethnocultural groups that originated in diverse countries in different continents, including Asia (e.g., Bangladesh and Pakistan), Africa (e.g., Somalia and Ghana), and Europe (e.g., Poland). In some cases, the sample was drawn from a single ethnocultural group, such as Bangladeshi.[48] However, most of the studies were undertaken on mixed samples of immigrant women originating from different countries (e.g., Somalia, Bangladesh, and Eastern Europe).

Studies focusing on immigrant women without a clearly specified ethnocultural group

We identified 16 studies that used the term immigrant women generically and not clearly specify an ethnocultural group. In deciding to include these studies, we believed that legitimate proxies for immigrant status could be the specified use of an interpreter or the participants having countries of origin or birth outside the UK. Some studies reported immigrant women arriving from 14 different countries but did not specify the country of birth. These studies could still be included.

Studies sorted by immigrant category of the participants

More than half of the included studies (25 in total) did not clearly specify the immigrant category of the population they studied, such as economic migrant, asylum seeker or refugee.[17, 39-42, 44-50, 52-64]

Other studies specified the immigrant category as asylum seekers only;[51, 65-68] refugees only;[17, 69-71] or a mix of immigrant categories that included spousal immigrants, economic migrants, asylum seekers, and refugees.[1, 2, 43, 72-74] Although a range of identifiers were used, we noted a pattern of reducing immigrant women and their complex cultures, ethnicities, and

For peer review only - http://bmjopen.bmj.com/site/about/guidelines.xhtml

Final

lives to simplified contexts or situations (e.g., ‘asylum seekers’) and using ‘immigrant’ as a generic label for a non-British and potentially non-white woman.

Studies sorted by phase of maternity care

For peer review only

For peer review only - http://bmjopen.bmj.com/site/about/guidelines.xhtml

Final

Of the included studies, 17 were undertaken across all phases of maternity care: antenatal, intrapartum, and postnatal.[1, 2, 46-48, 50, 54, 59-63, 65, 67, 69, 72, 75] Nine studies reported on antenatal care alone,[39, 43, 52, 56, 57, 66, 71, 73, 74] and six studies are focused on postnatal care.[44, 49, 51, 58, 64, 70] Two studies included focused on ante- and postnatal care but did not include the intrapartum phase.[42, 53] One study focused on just the intrapartum and postnatal phases of maternity care,[55] and one study did not specify the maternity care setting.[40]

INSERT FIGURE 4

We identified 40 research studies that met our inclusion criteria, and we extracted and synthesised key findings into five themes.

Theme 1: Access and utilisation of maternity care services by immigrant women

Late booking emerged as an important dimension in this theme that is that immigrant women study participants tended to book and access antenatal care later than the recommended timeframe (during the first 10 weeks of pregnancy). This issue was found to be multi-factorial in nature including issues such as limited English language proficiency, immigration status, lack of awareness of the services, lack of understanding of the purpose of the services, income barriers, the presence of female genital mutilation, differences between the maternity care systems of their countries of origin and the UK, arrival in the UK late in the pregnancy, frequent relocations after arrival, the poor reputations of antenatal services in specific communities and perceptions of regarding antenatal care as a facet of medicalisation of childbirth. The factors affecting the access and utilisation of postnatal services were similar to those reported for antenatal services.

Theme 2: Maternity care relationships between immigrant women and healthcare professionals.

Our included studies evidenced the perception of service users in this group and their interactions and therapeutic encounters with healthcare professionals. These interactions were significant in understanding access, utilisation, outcomes, and the quality of their maternity care experience.

Included studies in this theme demonstrated that the perceptions of study participants regarding the ways healthcare professionals delivered maternity care services were both positive and negative. A number of studies illustrated positive relationships between healthcare professionals and immigrant women. Study participants asserted that the healthcare professionals were caring, respected confidentiality, and communicated openly in meeting their medical as well as emotional, psychological, and social needs. Conversely,

For peer review only - http://bmjopen.bmj.com/site/about/guidelines.xhtml

Final

studies evidenced negative relationships between participants and healthcare professionals. Studies evidenced healthcare professionals from the perspective of immigrant as being rude, discriminatory, or insensitive to the cultural and social needs of the women. The end result of these negative encounters being that these women tended to avoid accessing utilising maternity care services consistently.

In our included studies, participants expressed a desire for the healthcare professionals to be empathetic, respectful, culturally congruent, and professional when providing maternity care services. Some women also suggested employing healthcare professionals from the immigrant population.

Theme 3: Communication challenges experienced by immigrant women in maternity care.

It is axiomatic that limited English language fluency presents verbal communication challenges between health care professional and their patients, families and carers. Moreover, this is compounded when healthcare professionals use complex medical or professional language that is difficult to comprehend. Nonverbal communication is culturally defined and challenges can occur through misunderstandings of facial expressions, gestures, or pictorial representations. Poor communications result as illustrated in our included studies in limited awareness of available services in addition to miscommunication with healthcare professionals. Study participants often expressed challenges accessing services, failed to understand procedures and their outcomes, were constrained in their ability to articulate their health or maternity needs to health care providers, were disempowered in respect of their involvement indecision making, often gave consent for clinical procedures without fully comprehending the risks and benefits, and did not receive understandable advice on baby care. Studies identified that communication was not reciprocal and healthcare professionals often misunderstood participants. These created feelings of isolation, fear and a perception of being ignored. Interventions to address the language challenges included the provision of formal and informal services, bilingual support workers, and written maternity care information in the necessary languages.

Theme 4: Organisation and legal entitlements and their impacts on the maternity care experiences of immigrant women.

The study participants in our included studies had mixed experiences with the maternity care services in the UK. Positive and commendable experiences included feeling safe in giving birth at hospital rather than at home, being able to register a complaint if poor healthcare was received, being close to a hospital facility, not being denied access to a maternity service, and having good experiences with postnatal care. Conversely, negative experiences included lack of continuity e.g. not being able to see same maternity care providers each time and being unaware of the configuration of maternity services work, limiting navigation. Participants in our included studies found services bureaucratic and perceived within the UK maternity care model a propensity towards medical/obstetric intervention and lower segment caesarean section births.

For peer review only - http://bmjopen.bmj.com/site/about/guidelines.xhtml

For peer review only

Final

The legal status of an immigrant women in the UK has a profound influence on their on their access to maternity care. Women without entitlement to free maternity care services in the UK were deterred from accessing timely antenatal care by the costs and by the confidentiality of their legal status. Moreover, some women arrived in the UK during the final phase of their pregnancies that resulted in fractures in the care process, loss of their social networks, reduced control over their lives, increased mental stress, and increased vulnerability to domestic violence.

Positive experiences included receiving information from their midwives on the benefits of breastfeeding together with demonstrations on how to position the baby. Negative experiences included poor support from hospital staff on how to breastfeed their babies consequently these reported experiences are mixed.

Theme 5: Discrimination, racism, stereotyping, cultural sensitivity, inaction, and cultural clash in maternity care for immigrant women.

Inequalities in access, navigation, utilisation and the subsequent maternity care outcomes are influenced by discrimination and cultural insensitivity in maternity care services according to the perspectives established in our included studies. Discrimination was often subtle and difficult to identify, but direct and overt discrimination was reported in some studies.

Specifically, study participants of Muslim faiths challenged assumptions held by healthcare professionals, including those held regarding Muslim food practices and that their partners or husbands should help the women during labour. In addition, evidence from our included studies suggested that they also felt that they were regarded

as different and dangerous people. Moreover, healthcare professionals were reported in some studies to lack cultural sensitivity and cultural understanding. For example, these women did not optimally benefit from antenatal classes facilitated by a non-Muslim educator who had no knowledge of the relationships of Muslim culture to maternity.

Furthermore, studies reported participant dissatisfaction of antenatal class with a gender mix, which contravened religious edicts. Studies illustrated that some women of Muslim faith felt their cultural and religious needs for breastfeeding were not met on the postnatal wards, and they felt that the staff lacked insight, knowledge and understanding of female genital mutilation (FGM).

Evidence from our included studies suggests immigrant women perceived that the staff did not treat them with respect or full attention. They felt devalued, unsupported, and fearful while receiving maternity care. In a few cases, however, midwives were happy to meet the cultural and religious needs of the study participants in our included studies in both antenatal and postnatal settings and this is a, positive finding.

For peer review only - http://bmjopen.bmj.com/site/about/guidelines.xhtml

Final

Our findings also identified instances of cultural clash and conflicting advice during pregnancy and maternity care, mostly resulting from differences between the home countries of the immigrants and the UK in their cultural practices and medical systems. We conceptualise the findings graphically in figure 5.

INSERT FIGURE

Discussion and conclusions

The experiences of immigrant women in accessing, navigating and utilising maternity care services in the UK are both positive and negative. However, evidence in our review suggests that immigrant women largely had poor experiences. The experience of maternity care services in multi-factorial in nature and a number of issues coalesce to determine this poorer experience. Issues evidenced in the review included lack of language support, cultural insensitivity, discrimination, poor relationships between immigrant women and healthcare professionals, and a lack of legal entitlements and guidelines on the provision of welfare support and maternity care to immigrants.

Implications of findings and recommendations for maternity care policy, practice, and service delivery

It would seem imperative to adopt a universal of aim of achieving optimal maternity care for all and not just for immigrant women. However, maternity care services should strive to give more information and knowledge to immigrant women about their rights to care, the availability and configuration of maternity services, and how to navigate maternity care systems. The child in utero of an immigrant is a future UK citizen and optimising maternity care is a dimension of securing the future health of the nation. In a period of super diversity is incumbent upon health professional to have an awareness of immigrant women's legal rights and perhaps education on this topic should be mandated for maternity care professionals. Continuity in maternity caregivers and compulsory provision of interpreters would also help to improve the experiences of these women.

Inequitable access appeared to be an artefact of the immigration and legal status of asylum-seeking women, and has a profound impact on health care experiences and consequently health. Paramount is the consideration of language fluency, as this appears to be key determinant of optimal access and utilisation of maternity care services. We concluded that addressing language barriers and ensuring culturally sensitive care are essential elements of providing optimal maternal care for immigrant women. The issue of confidentiality may be compromised by having known interpreters in small communities: lack of confidentiality can make finding ways to communicate with women with low levels of English more problematic. Setting up a national-level website offering standard information on maternity care with the option of translation in a wide range of languages may be a solution.

For peer review only - http://bmjopen.bmj.com/site/about/guidelines.xhtml

Final

Additionally, the identification of best language practices should be identified with regard to improve the current language service model.

The knowledge, understandings and attitudes of maternity care health care providers is a critical determinant of care. Ethno-culturally based stereotypes, racism, judgmental views, and direct and indirect discrimination require eradication. Challenging discrimination and racism are needed at all levels: individual, institutional, clinical, and societal is an urgent imperative. Interventions to improve maternity care for immigrant women are scant, and economic evaluations of these interventions were very absent. In addition, the interventions needed to be more focused and implemented at the organisational, service, and staff levels. Increasing the social capital, health literacy, and advocacy resources for immigrant women would empower them to access and utilise maternity care services appropriately.

Maternity care staff require a greater level of mandated education to have better cultural awareness of needs of diverse client groups including newcomers to the UK. Our findings highlight the importance of demonstrating compassion, empathy, and warmth in their relationships with these women to reinforce positive attitudes among the immigrant women.

It is contingent on maternity care providers to value diversity among service users and to offer individualised and culturally congruent care. One way to achieve this goal would be through birth plans that can be jointly agreed and discussed in advance by the maternity care staff and recently arrived newcomers and immigrant women. Maternity care staff should seek to empower immigrant women by providing comprehensible information and better education concerning the configuration of the maternity system in UK, conveying accurate information about care delivery. Central to these suggestions may be to enable volunteer and third-sector organisations to work as links between the statutory maternity services and immigrant women.

Representatives of immigration control agencies may feel obligated to adhere to immigrant rules and consider the maternity care needs of immigrant women's and baby's health as a secondary issue. The policy context regarding data protection and sharing information with the Home Office about the immigrant status of women was at issue as well, especially since variabilities have been seen in the policies for sharing this information. The results suggest that the legal and policy context is important in addressing the maternity care needs of immigrant women.

Gaps in the evidence

We found that very few interventions in the published literature that had been implemented to address inequalities in access and quality in maternity care for immigrant women, and the effectiveness of these few had not been evaluated robustly. Studies evaluating the effectiveness of these interventions were almost absent from the literature, and no single study existed on the economic evaluation of the interventions. Studies of the usual 6 weeks postnatal checks by a general practitioner were not identified.

For peer review only - http://bmjopen.bmj.com/site/about/guidelines.xhtml

Final

Strengths and limitations

- We were challenged and constrained by the lack of consistency in describing immigrant population sin the published literature. There is exists a great deal of variation and no unified approach within the UK literature.
- Immigration is an international phenomenon and this review increases understanding of how immigrant women navigate maternity services in the UK
- The review systematically maps our positive and negative aspects of maternity care provision as experienced by immigrant
- The review provides strategic direction for enhancement of maternity care services
- The review does not address the experiences of maternity care for second generation women (e.g. women of black and minority origin born in the UK)

Implications for future research

More research is required into the term 'immigrant', how this term is used, and the changes in its use over time that may affect immigrant women's care. At present, the term is used very broadly and simplistically, which masks its inherent heterogeneity. Furthermore, more research is required to understand how the intersections of particular characteristics – such as gender, education status, time in the UK, immigration status, wealth, and country of origin – may influence or alter the experiences of these women in their maternity. Research is required that focuses on specific interventions to improve maternity care for immigrant women.

COMPETING INTEREST
None

AUTHOR CONTRIBUTIONS

Dr Gina Higginbottom (Professor, School of |Health Sciences) was principal investigator. Initiated the project and oversaw all stages. Led the interpretation/synthesis phases and drafted the manuscript.

Dr. Basharat Hussain (Senior Research Fellow) contributed to all stages of the review. Led the data extraction, coding, and quality appraisal and contributed to the manuscript.

Dr. Catrin Evans (Associate Professor of Nursing, Director of the Centre for Evidence Based Health Care) contributed to all stages of the review, provided expert methodological advice, acted as second reviewer for quality appraisal, and development of the synthesis. She contributed to the review of the final version of the manuscript.

For peer review only - http://bmjopen.bmj.com/site/about/guidelines.xhtml

For peer review only

Final

Dr Myfanwy Morgan (Professor Emerita Kings College) contributed to all stages of the review, provided expert methodological advice, acted as second reviewer for quality appraisal, and development of the synthesis. She contributed to the review of the manuscript.

Dr Kuldip Bharj (Retired Director of Midwifery, University of Leeds) contributed to all stages of the review, provided clinical and policy perspectives, contributed to formulation of the implications and recommendation in the manuscript.

Jeanette Eldridge (Information Specialist) designed the literature search strategy, advised the team on all aspects of information retrieval, undertook the main database searches, and contributed to the development of the manuscript.

Disclaimer

The views expressed in this report are those of the authors and not necessarily those of the NHS, NIHR or the Department of Health.

Patient and Public Involvement

The systematic review questions were developed in consultation with our project advisory group (PAG) including service users' priorities experience and preferences. This systematic review did not include empirical research therefore there were no human participants.

Acknowledgements

This project was funded by the UK NIHR Health Services and Delivery Research Programme (Grant No. HS&DR-15/55/03). Along with this funding, NIHR also contributed by peer reviewing the funding proposal.
We gratefully acknowledge valuable input from the members of our Project Advisory Group. Their input has been very helpful in making possible the successful completion and the high quality of this review. The following people kindly consented to be members:

- Jim Thornton, Professor of Obstetrics and Gynaecology, Faculty of Medicine and Health Sciences, University of Nottingham
- Dr Caroline Mitchell, General Practitioner/Senior Clinical Lecturer, Clinical Academic Training Programme Lead, Academic Unit of Primary Medical Care (AUPMC), University of Sheffield
- Dr Jane Mischenko, Commissioning Lead: Children and Maternity Services, NHS Leeds
- Carol McCormack, Specialist Midwife, NUH Trust

We also thank following immigrant women for their input in the conceptualisation of this review:

For peer review only - http://bmjopen.bmj.com/site/about/guidelines.xhtml

Final

- Ms Valentine Nkoyo, Director of Mojatu, Nottingham
- Kinsi Clarke, Nottingham Refugee Forum

IN TEXT TABLES

Table 1: Thick and Thin Criteria *Higginbottom et al* [19]

Richness	Operational Definition
Thick papers	• Offer greater explanatory insights into the outcome of interest<br>• Provide a clear account of the process by which the findings were produced—including the sample, its selection and its size, with any limitations or bias noted—along with clear methods of analysis<br>• Present a developed and plausible interpretation of the analysis based on the data presented.
Thin papers	• Offer only limited insights<br>• Lack a clear account of the process by which the findings were produced<br>• Present an underdeveloped and weak interpretation of the analysis based on the data presented

References

1. Phillimore J. Delivering maternity services in an era of superdiversity: The challenges of novelty and newness. *Ethnic and Racial Studies* 2015; 38: 568-582. Health & Mental Health Services 3370. DOI: 10.1080/01419870.2015.980288 http://ovidsp.ovid.com/ovidweb.cgi?T=JS&PAGE=reference&D=psyc12&NEWS=N&AN=2015-02651-003; http://dx.doi.org/10.1080/01419870.2015.980288.

2. Phillimore J. Migrant maternity in an era of superdiversity: New migrants' access to, and experience of, antenatal care in the West Midlands, UK. *Social Science and Medicine* 2016; 148: 152-159. DOI: 10.1016/j.socscimed.2015.11.030. http://ovidsp.ovid.com/ovidweb.cgi?T=JS&PAGE=reference&D=medl&NEWS=N&AN=26705910; https://www.sciencedirect.com/science/article/pii/S0277953615302380?via%3Dihub.

3. Office of National Statistics. *Birth in England and Wales by Parents Country of Birth (2013)*. 2014. Available from https://www.ons.gov.uk/peoplepopulationandcommunity/birthsdeathsandmarriages/livebirths/bulletins/parentscountryofbirthenglandandwales/2014-08-28).

4. Manktelow B, Smith L, Seaton S, Hyman-Taylor P, Kurinczuk J, Field D, et al. *MBRRACE-UK Perinatal Mortality Surveillance Report, UK Perinatal Deaths for Births from January to December 2014*. 2016. Leicester: The Infant Mortality and Morbidity Studies, Department of Health Sciences, University of Leicester. Available from https://www.npeu.ox.ac.uk/downloads/files/mbrrace-uk/reports/MBRRACE-UK-PMS-Report-2014.pdf).

For peer review only - http://bmjopen.bmj.com/site/about/guidelines.xhtml

Final

5. Department of Health. *Midwifery 2020: Delivering expectations*. 2010. Available from https://assets.publishing.service.gov.uk/government/uploads/system/uploads/attachment_data/file/216029/dh_119470.pdf).
6. Garcia R, Ali N, Papadopoulos C and Randhawa G. Specific antenatal interventions for Black, Asian and Minority Ethnic (BAME) pregnant women at high risk of poor birth outcomes in the United Kingdom: a scoping review. *BMC Pregnancy and Childbirth* 2015; 15: 226. DOI: 10.1186/s12884-015-0657-2.
7. Higginbottom G, Morgan M, Alexandre M, Chiu Y, Forgeron J, Kocay D, et al. Immigrant women's experiences of maternity-care services in Canada: a systematic review using a narrative synthesis. *Systematic Reviews* 2015; 4: 13. DOI: 10.1186/2046-4053-4-13.
8. Salway SM, Higginbottom G, Reime B, Bharj KK, Chowbey P, Foster C, et al. Contributions and challenges of cross-national comparative research in migration, ethnicity and health: insights from a preliminary study of maternal health in Germany, Canada and the UK. *BMC Public Health* 2011; 11: 514. DOI: 10.1186/1471-2458-11-514.
9. Higginbottom GM, Safipour J, Yohani S, O'Brien B, Mumtaz Z and Paton P. An ethnographic study of communication challenges in maternity care for immigrant women in rural Alberta. *Midwifery* 2015; 31: 297-304. DOI: 10.1016/j.midw.2014.09.009.
10. Tobin CL, Di Napoli P and Beck CT. Refugee and Immigrant Women's Experience of Postpartum Depression: A Meta-Synthesis. *Journal of Transcultural Nursing* 2017: 84-100. DOI: 10.1177/1043659616686167.
11. Gagnon A ZM, Zeitlin J, Roam Collaboration. Migration to western industrialised countries and perinatal health: a systematic review. *Social Science and Medicine* 2009; 69: 934-946. DOI: 10.1016/j.socscimed.2009.06.027.
12. Gulliford M, Figueroa-Munoz J, Morgan M, Hughes D, Gibson B, Beech R, et al. What does 'access to health care' mean? *Journal of Health Services Research and Policy* 2002; 7: 186-188. DOI: 10.1258/135581902760082517.
13. Guendelman S, Buekens P, Blondel B, Kaminski M, Notzon FC and Masuy-Stroobant G. Birth outcomes of immigrant women in the United States, France, and Belgium. *Maternal and Child Health Journal* 1999; 3: 177-187. DOI: 10.1023/A:102232802.
14. Carolan M and Cassar L. Antenatal care perceptions of pregnant African women attending maternity services in Melbourne, Australia. *Midwifery* 2010; 26: 189-201. DOI: 10.1016/j.midw.2008.03.005.
15. Small R, Yelland J, Lumley J, Brown S and Liamputtong P. Immigrant women's views about care during labor and birth: an Australian study of Vietnamese, Turkish, and Filipino women. *Birth* 2002; 29: 266-277. DOI: 10.1046/j.1523-536X.2002.00201.x
16. Essén B, Johnsdotter S, Hovelius B, Gudmundsson S, Sjöberg NO, Friedman J, et al. Qualitative study of pregnancy and childbirth experiences in Somalian women resident in Sweden. *BJOG: An International Journal of Obstetrics and Gynaecology* 2000; 107: 1507-1512. DOI: 10.1111/j.1471-0528.2000.tb11676.x.
17. Binder P, Johnsdotter S and Essen B. Conceptualising the prevention of adverse obstetric outcomes among immigrants using the 'three delays' framework in a high-income context. *Social Science and Medicine* 2012; 75: 2028-2036. DOI: 10.1016/j.socscimed.2012.08.010. http://ovidsp.ovid.com/ovidweb.cgi?T=JS&PAGE=reference&D=med7&NEWS=N&AN=22940012.
18. Drewniak D, Krones T and Wild V. Do attitudes and behavior of health care professionals exacerbate health care disparities among immigrant and ethnic minority groups? An integrative literature review. *International Journal of Nursing Studies* 2017; 70: 89-98. DOI: 10.1016/j.ijnurstu.2017.02.015.

For peer review only - http://bmjopen.bmj.com/site/about/guidelines.xhtml

Final

19. Mumtaz Z, O'Brien B and Higginbottom G. Navigating maternity health care: a survey of the Canadian prairie newcomer experience. *BMC Pregnancy and Childbirth* 2014; 14: 4. DOI: 10.1186/1471-2393-14-4.
20. Mullins T. Definition of 'immigrant'. In: Hussain B (ed). London: Migration Statistics Unit, Office for National Statistics, 2017.
21. The King's Fund. What do we know about the impact of immigration on the NHS? Available from https://www.kingsfund.org.uk/projects/verdict/what-do-we-know-about-impact-immigration-nhs (2015, accessed 08/08/2018).
22. Jayaweera H. *Health of migrants in the UK: What do we know?* 2011. The Migration Observatory, University of Oxford. Available from https://migrationobservatory.ox.ac.uk/resources/briefings/health-of-migrants-in-the-uk-what-do-we-know/ (accessed 08/08/2018).
23. National Institute for Health and Clinical Excellence (NICE). *Pregnancy and complex social factors: A model for service provision for pregnant women with complex social factors*. 2010. Available from https://www.nice.org.uk/guidance/cg110).
24. Ramsden I. From facts to practice: cultural safety and nursing education in Aotearoa and Te Waipounamu. In: *Binan Goonj: bridging cultures in Aboriginal health*. 2nd ed. Sydney: Elsevier, 2009. https://www.elsevier.com/books/binan-goonj/eckermann/978-0-7295-3936-4
25. Ramsden I. *Cultural safety and nursing education in Aotearoa and Te Waipounamu*. PhD, Victoria University of Wellington New Zealand, 2002.
26. Papps E and Ramsden I. Cultural safety in nursing: The New Zealand experience. *International Journal for Quality in Health Care* 1996; 8: 491-497. DOI: 10.1093/intqhc/8.5.491.
27. Nursing Council of New Zealand. Guidelines for Cultural Safety, the Treaty of Waitangi, and Maori Health in Nursing and Midwifery Education and Practice. Available from http://www.nursingcouncil.org.nz/culturalsafety.pdf. (2002, accessed 26 February).
28. Brascoupé S and Catherine Waters BA M. Cultural safety: Exploring the applicability of the concept of cultural safety to Aboriginal health and community wellness. *International Journal of Indigenous Health* 2009; 5: 6. DOI: 10.18357/ijih52200912332.
29. Aboriginal Nurses Association of Canada. Cultural Competence and Cultural Safety in Nursing Education: A Framework for First Nations, Inuit and Métis Nursing. Available from https://www.cna-aiic.ca/~/media/cna/page-content/pdf-en/first_nations_framework_e.pdf (2009, accessed 26 February).
30. Popay J, Roberts H, Sowden A, Petticrew M, Arai L, Rodgers M, et al. *Guidance on the conduct of narrative synthesis in systematic reviews*. 2006. Available from http://citeseerx.ist.psu.edu/viewdoc/download?doi=10.1.1.178.3100&rep=rep1&type=pdf).
31. Booth A. *Supplementary Guidance for Inclusion of Qualitative Research in Cochrane Systematic Reviews of Interventions*. Version 1 (updated August 2011) ed.: Cochrane Collaboration Qualitative Methods Group, 2016.
32. Higginbottom GM, Morgan M, O'Mahony J, Chiu Y, Kocay D, Alexandre M, et al. Immigrant women's experiences of postpartum depression in Canada: a protocol for systematic review using a narrative synthesis. *Systematic Reviews* 2013; 2: 65. DOI: 10.1186/2046-4053-2-65.
33. Center for Evidence-Based Management. Critical Appraisal Tools & Questionnaires. Available from https://www.cebma.org/ (2017, accessed 15 February).
34. O'Cathain A. Reporting Mixed Methods Projects. In: *Mixed Methods Research for Nursing and the Health Sciences*. Blackwell, 2009, pp.135-158.
35. Roper JM SJ. *Ethnography in Nursing Research*. Sage Publications, 2000.

For peer review only - http://bmjopen.bmj.com/site/about/guidelines.xhtml

Final

36. Tashakorri C TA. *Foundations in mixed methods research; Integrating Quantitative and Qualitative Approaches in the Social and Behavioral Sciences*. Thousand Oaks, Ca: Sage Publications, 2008.
37. Murphy E DR, Greatbatch D, Parker S, Watson P Qualitative research methods in health technology assessment: a review of the literature Health Technology Assessment. *Health Technology Assessment Reports* 1998; 2.
38. Moher D, Liberati A, Tetzlaff J, Altman DG and Group P. Preferred reporting items for systematic reviews and meta-analyses: the PRISMA statement. *PLoS Medicine* 2009; 6: e1000097. DOI: 10.1371/journal.pmed.1000097.
39. Rowe RE, Magee H, Quigley MA, Heron P, Askham J and Brocklehurst P. Social and ethnic differences in attendance for antenatal care in England. *Public Health* 2008; 122: 1363-1372. Health & Mental Health Services 3370. DOI: 10.1016/j.puhe.2008.05.011. http://ovidsp.ovid.com/ovidweb.cgi?T=JS&PAGE=reference&D=psyc6&NEWS=N&AN=2009-02349-007; http://dx.doi.org/10.1016/j.puhe.2008.05.011.
40. Hicks C and Hayes L. Linkworkers in antenatal care: facilitators of equal opportunities in health provision or salves for the management conscience? *Health Services Management Research* 1991; 4: 89-93. DOI: 10.1177/095148489100400202.
41. Hawkins SS, Lamb K, Cole TJ and Law C. Influence of moving to the UK on maternal health behaviours: Prospective cohort study. *BMJ* 2008; 336: 1052-1055. DOI: 10.1136/bmj.39532.688877.25. http://ovidsp.ovid.com/ovidweb.cgi?T=JS&PAGE=reference&D=emed11&NEWS=N&AN=351692247.
42. Tucker A, Ogutu D, Yoong W, Nauta M and Fakokunde A. The unbooked mother: a cohort study of maternal and foetal outcomes in a North London Hospital. *Archives of Gynecology and Obstetrics* 2010; 281: 613-616. DOI: 10.1007/s00404-009-1152-7. http://ovidsp.ovid.com/ovidweb.cgi?T=JS&PAGE=reference&D=med6&NEWS=N&AN=19551396.
43. Cresswell JA, Yu G, Hatherall B, Morris J, Jamal F, Harden A, et al. Predictors of the timing of initiation of antenatal care in an ethnically diverse urban cohort in the UK. *BMC Pregnancy and Childbirth* 2013; 13: 103. DOI: 10.1186/1471-2393-13-103. http://ovidsp.ovid.com/ovidweb.cgi?T=JS&PAGE=reference&D=medl&NEWS=N&AN=23642084.
44. Gorman DR, Katikireddi SV, Morris C, Chalmers JWT, Sim J, Szamotulska K, et al. Ethnic variation in maternity care: a comparison of Polish and Scottish women delivering in Scotland 2004-2009. *Public Health* 2014; 128: 262-267. DOI: 10.1016/j.puhe.2013.11.008. http://ovidsp.ovid.com/ovidweb.cgi?T=JS&PAGE=reference&D=medl&NEWS=N&AN=24613449.
45. Pershad P and Tyrrell H. *Access to antenatal and postnatal services for Asian women living in East Pollokshields, Glasgow*. 1995. Save the Children. Available from).
46. Redshaw M, Rowe R, Hockley C and Brocklehurst P. *Recorded delivery: a national survey of women's experience of maternity care 2006*. 2007. Oxford: National Perinatal Epidemiology Unit (NPEU). Available from https://www.npeu.ox.ac.uk/recorded-delivery).
47. Shortall C, McMorran J, Taylor K, Traianou A, Garcia de Frutos M, Jones L, et al. *Experiences of Pregnant Migrant Women receiving Ante/Peri and Postnatal Care in the UK: A Doctors of the World Report on the Experiences of attendees at their London Drop-In Clinic*. 2015. Available from https://b.3cdn.net/droftheworld/08303864eb97b2d304_lam6brw4c.pdf).
48. Duff LA, Ahmed LB and Lamping DL. Evaluating satisfaction with maternity care in women from minority ethnic communities : development and validation of a Sylheti

For peer review only - http://bmjopen.bmj.com/site/about/guidelines.xhtml

Final

questionnaire. *International Journal for Quality in Health Care* 2002; 13: 215-230. DOI: 10.1093/intqhc/13.3.215.
49. Ahmed S, Macfarlane A, Naylor J and Hastings J. Evaluating bilingual peer support for breastfeeding in a local sure start. *British Journal of Midwifery* 2006; 14: 467-470. DOI: 10.12968/bjom.2006.14.8.21640. http://search.ebscohost.com/login.aspx?direct=true&db=rzh&AN=106354580&site=ehost-live.
50. Cross-Sudworth F, Williams A and Herron-Marx S. Maternity services in multi-cultural Britain: Using Q methodology to explore the views of first- and second-generation women of Pakistani origin. *Midwifery* 2011; 27: 458-468. DOI: 10.1016/j.midw.2010.03.001. http://search.ebscohost.com/login.aspx?direct=true&db=rzh&AN=104661096&site=ehost-live.
51. O'Shaughnessy R, Nelki J, Chiumento A, Hassan A and Rahman A. Sweet Mother: evaluation of a pilot mental health service for asylum-seeking mothers and babies. *Journal of Public Mental Health* 2012; 11: 214-228. DOI: 10.1108/17465721211289392.
52. Leeds Family Health. *Research into the uptake of maternity services as provided by primary health care teams to women from black and minorities*. 1992. London: Leeds FHSA. Available from).
53. Almalik M. *A comparative evaluation of postnatal care for migrant and UK-born women*. PhD, University of Aberdeen, 2011.
54. BEMIS Scotland and Community Food and Health (Scotland). *Strengthening food work across ethnic minority communities: A focus on maternal and infant nutrition*. 2013. Available from https://www.communityfoodandhealth.org.uk/publications/strengthening-food-work-ethnic-minority-communities-focus-maternal-infant-nutrition/).
55. Jomeen J and Redshaw M. Ethnic minority women's experience of maternity services in England. *Ethnicity and Health* 2013; 18: 280-296. DOI: 10.1080/13557858.2012.730608. http://ovidsp.ovid.com/ovidweb.cgi?T=JS&PAGE=reference&D=medl&NEWS=N&AN=23039872.
56. Raine R, Cartwright M, Richens Y, Mahamed Z and Smith D. A qualitative study of women's experiences of communication in antenatal care: identifying areas for action. *Maternal and Child Health Journal* 2010; 14: 590-599. DOI: 10.1007/s10995-009-0489-7. http://ovidsp.ovid.com/ovidweb.cgi?T=JS&PAGE=reference&D=med6&NEWS=N&AN=19554436.
57. Greenhalgh T, Clinch M, Afsar N, Choudhury Y, Sudra R, Campbell-Richards D, et al. Socio-cultural influences on the behaviour of South Asian women with diabetes in pregnancy: Qualitative study using a multi-level theoretical approach. *BMC Medicine* 2015; 13: 120. DOI: 10.1186/s12916-015-0360-1. http://ovidsp.ovid.com/ovidweb.cgi?T=JS&PAGE=reference&D=emed17&NEWS=N&AN=604640620.
58. de Chavez AC, Ball HL and Ward-Platt M. Bi-ethnic infant thermal care beliefs in Bradford, UK. *International Journal of Human Rights in Healthcare* 2016; 9: 120-134. DOI: 10.1108/IJHRH-08-2015-0024. http://ovidsp.ovid.com/ovidweb.cgi?T=JS&PAGE=reference&D=psyc13&NEWS=N&AN=2016-25577-005.
59. Warrier S. *Consumer empowerment: a qualitative study of linkworker and advocacy services for non-English speaking users of maternity services*. Report no. 9780946741205, 1996. London: The Maternity Alliance. Available from).
60. Ali N. *Experiences of maternity services: Muslim women's perspectives*. 2004. London: The Maternity Alliance. Available from https://www.maternityaction.org.uk/wp-content/uploads/2013/09/muslimwomensexperiencesofmaternityservices.pdf).

For peer review only - http://bmjopen.bmj.com/site/about/guidelines.xhtml

Final

61. Bawadi HA. *Migrant Arab Muslim women's experiences of childbirth in the UK*. PhD, De Montfort University, 2009.
62. Lee JY. *'My body is Korean, but not my child's...': a Foucauldian approach to Korean migrant women's health-seeking behaviours in the UK*. PhD, University of Nottingham, 2010.
63. Baldeh F. *Obstetric Care in Scotland: the experience of women who have undergone Female Genital Mutilation (FGM)*. MSc (Masters), Queen Margaret University, 2013.
64. Lamba R. *A Qualitative Study Exploring Migrant Pakistani-Muslim Women's Lived Experiences and Understanding of Postnatal Depression*. PhD, University of East London, 2015.
65. McLeish J. Maternity experiences of asylum seekers in England. *British Journal of Midwifery* 2005; 13: 782-785. DOI: 10.12968/bjom.2005.13.12.20125. http://search.ebscohost.com/login.aspx?direct=true&db=rzh&AN=106416342&site=ehost-live.
66. Feldman R. When maternity doesn't matter: Dispersing pregnant women seeking asylum. *British Journal of Midwifery* 2014; 22: 23-28. DOI: 10.12968/bjom.2014.22.1.23. http://search.ebscohost.com/login.aspx?direct=true&db=rzh&AN=104006997&site=ehost-live.
67. Lephard E and Haith-Cooper M. Pregnant and seeking asylum: Exploring women's experiences' from booking to baby'. *British Journal of Midwifery* 2016; 24: 130-136. DOI: 10.12968/bjom.2016.24.2.130. https://www.magonlinelibrary.com/doi/10.12968/bjom.2016.24.2.130.
68. Nabb J. Pregnant asylum-seekers: Perceptions of maternity service provision. *Evidence-Based Midwifery* 2006; 4: 89-96. http://create.canterbury.ac.uk/5890/.
69. Harper Bulman K and McCourt C. Somali refugee women's experiences of maternity care in west London: a case study. *Critical Public Health* 2002; 12: 365-380. DOI: 10.1080/0958159021000029568. http://ovidsp.ovid.com/ovidweb.cgi?T=JS&PAGE=reference&D=mwic&NEWS=N&AN=2003020613.
70. Hufton E and Raven J. Exploring the infant feeding practices of immigrant women in the North West of England: A case study of asylum seekers and refugees in Liverpool and Manchester. *Maternal and Child Nutrition* 2016; 12: 299-313. DOI: 10.1111/mcn.12145. http://onlinelibrary.wiley.com/journal/10.1111/(ISSN)1740-8709 http://ovidsp.ovid.com/ovidweb.cgi?T=JS&PAGE=reference&D=emed18a&NEWS=N&AN=609162428.
71. Moxey JM and Jones LL. A qualitative study exploring how Somali women exposed to female genital mutilation experience and perceive antenatal and intrapartum care in England. *BMJ Open* 2016; 6: e009846. DOI: 10.1136/bmjopen-2015-009846.
72. Essén B, Binder P and Johnsdotter S. An anthropological analysis of the perspectives of Somali women in the West and their obstetric care providers on caesarean birth. *Journal of Psychosomatic Obstetrics and Gynaecology* 2011; 32: 10-18. DOI: 10.3109/0167482X.2010.547966.
73. Binder P, Borne Y, Johnsdotter S and Essen B. Shared language is essential: communication in a multiethnic obstetric care setting. *Journal of Health Communication* 2012; 17: 1171-1186. DOI: 10.1080/10810730.2012.665421. http://ovidsp.ovid.com/ovidweb.cgi?T=JS&PAGE=reference&D=emed14&NEWS=N&AN=366382910.
74. Goodwin L. *The midwife-woman relationship in a South Wales community: a focused ethnography of the experiences of midwives and migrant Pakistani women in early pregnancy*. PhD, Cardiff University, 2016.

For peer review only - http://bmjopen.bmj.com/site/about/guidelines.xhtml

Final

75. Briscoe L and Lavender T. Exploring maternity care for asylum seekers and refugees. *British Journal of Midwifery* 2009; 17: 17-24. DOI: 10.12968/bjom.2009.17.1.37649. http://search.ebscohost.com/login.aspx?direct=true&db=rzh&AN=105631311&site=ehost-live.

For peer review only

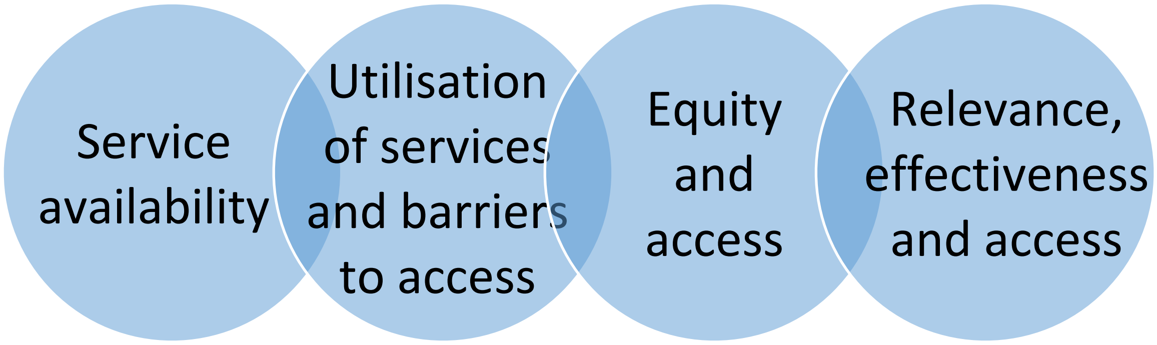

Figure 1: "Gulliford theory of access"[12]

For peer review only

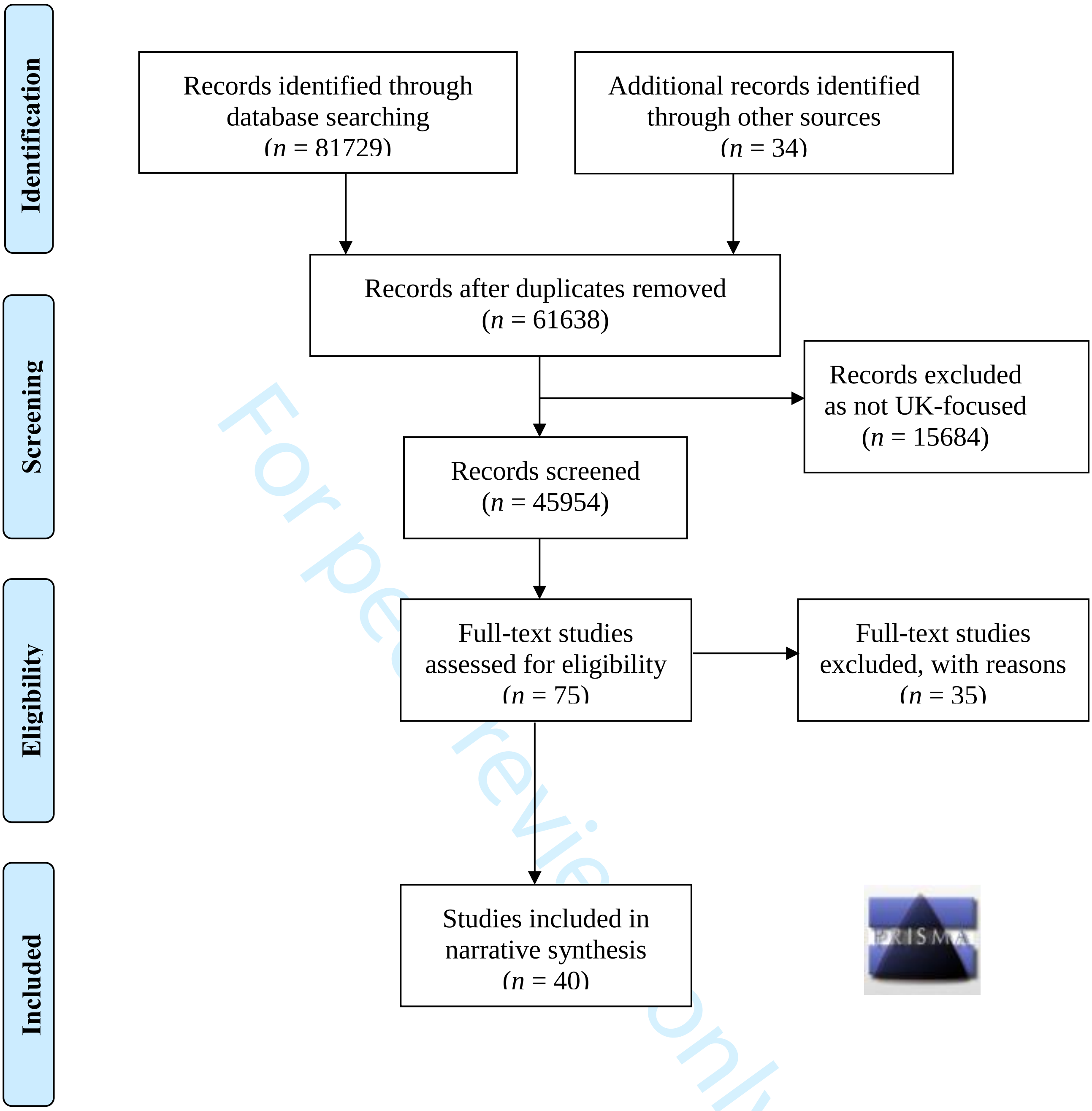

Figure 2: PRISMA flow diagram of the final selection process.

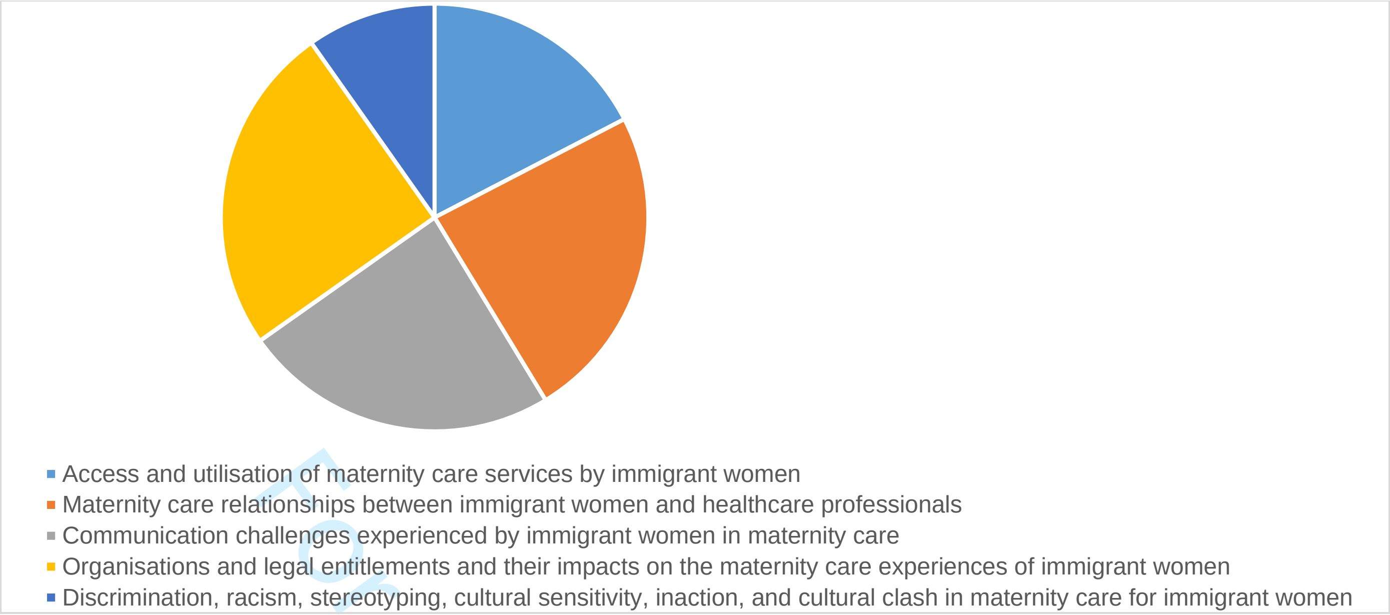

.***Figure 3:*** *The* total numbers of studies involved in each theme

For peer review only

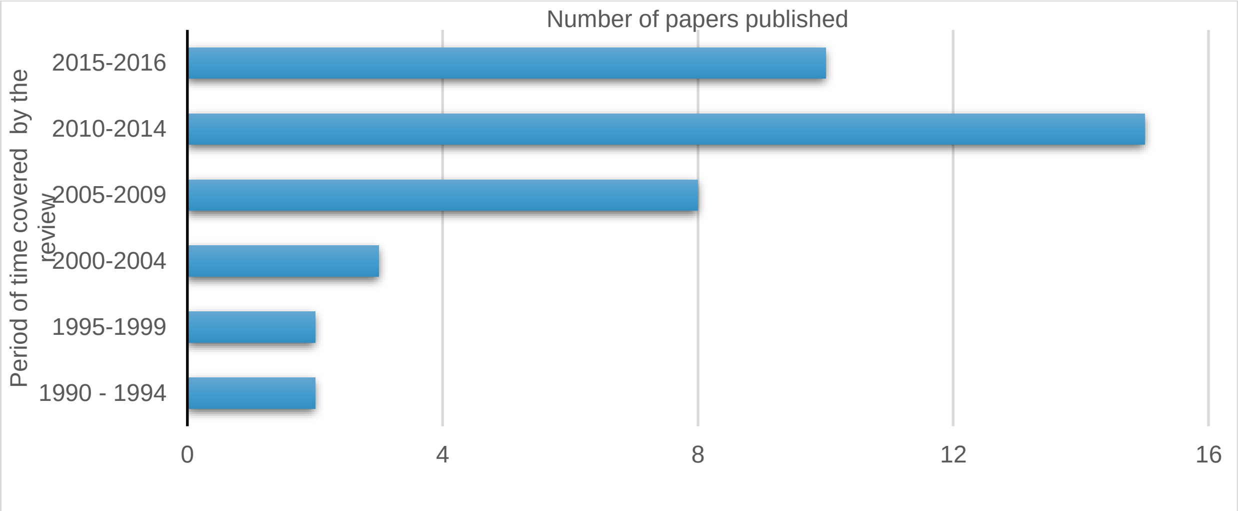

Figure 4: The range of publication dates for the included studies (1990–2016).

For peer review only

Figure 5: Immigrant women's experiences of maternity care in the UK

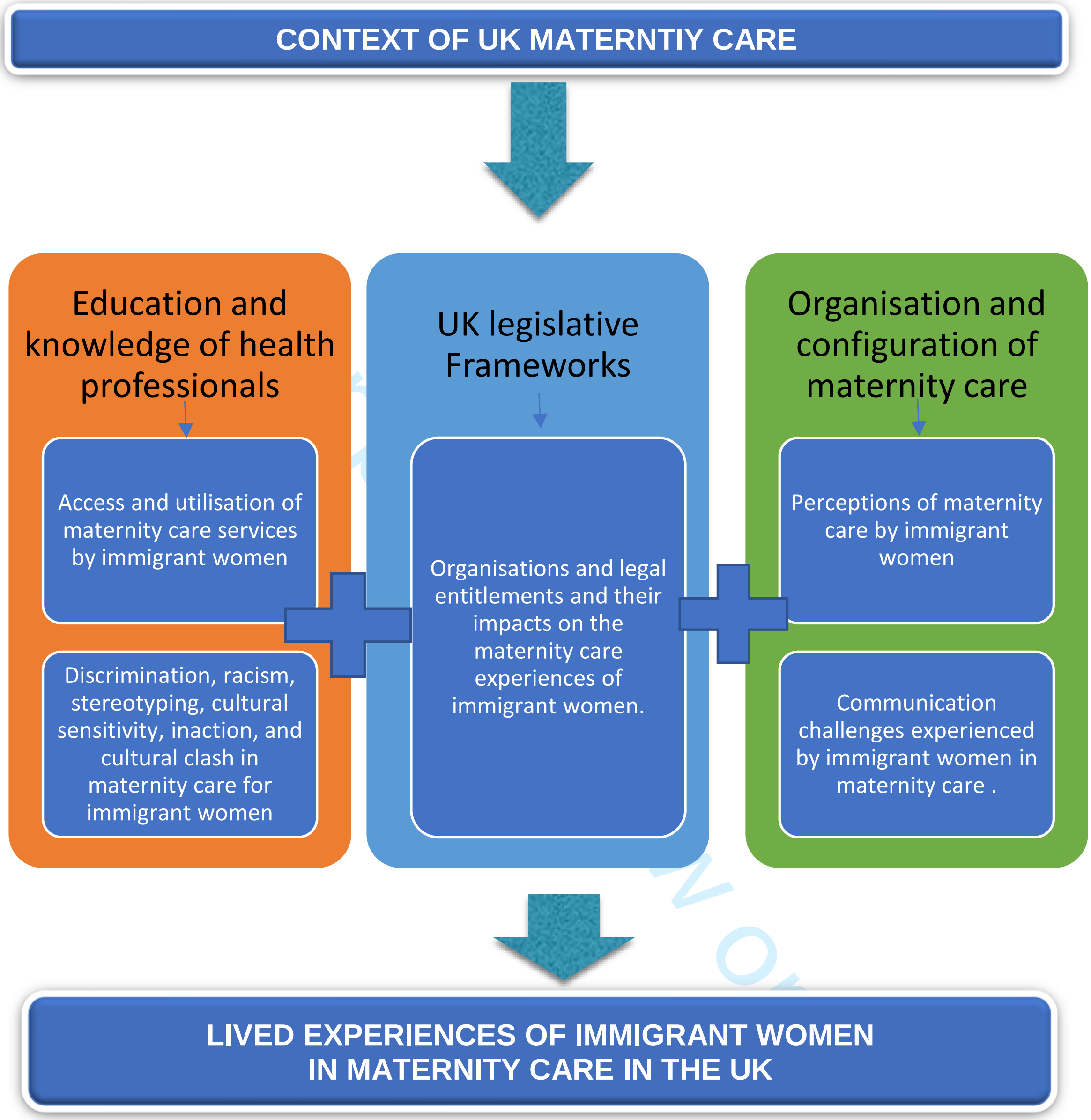

SUPPLEMENTARY FILES
File 1: Search Strategy
File 2: Bibliographic databases searched
File 3: Inclusion/Exclusion critiera
File 4: Excluded papers and rationale
File 5: Master table of included studies
File 6: Characteristics of study participants

For peer review only

File 1: Search Strategy

:Review search strategy - Medline

1	Maternal Health Services/ or Postnatal Care/ or Preconception Care/ or Prenatal Care/ or Perinatal Care/ or Infant Care/ or Midwifery/ or Obstetrics/ or General Practitioners/ or Primary Health Care/ or Family Health/	162335	
2	((maternal or child* or baby or babies or fetus* or fetal* or	119288	Field modified from .mp. to .ti,ab.
3	((birth* or matern* or mother* or pregnan* or childbearing or child-bearing or prenatal or pre-natal or postnatal or post-natal or perinatal or peri-natal or **preconception** or pre-conception or antenatal or ante-natal or postpartum or puerperium) adj3 (health* or nurs* or care or service*)).ti,ab.	65240	Field modified from .mp. to .ti,ab.
4	exp Midwifery/ or exp Obstetric Nursing/ or exp	47851	Field modified from .mp. to .ti,ab.
5	exp Health Services Accessibility/ or exp	1829149	
6	5 and (matern* or child* or baby or babies or fetus* or fetal* or embryo* or obstetric* or birth* or mother* or pregnan* or childbearing or child-bearing or prenatal or pre-natal or postnatal or post-natal or perinatal or peri-natal or preconception or pre-conception or antenatal or ante-natal or postpartum or puerperium).ti,ab.	253992	Limit 5 to female did not sufficiently focus previous search strategy so text terms used instead; Field modified from .mp. to .ti,ab.
7	1 or 2 or 3 or 4 or 6	490776	
8	("use" or access* or utili* or consum* or block* or hurdl* or barrier* or hindr* or hinder* or obstacle* or exclu* or discrimin* or disparit* or disproportion* or inequal* or unequal* or inadequat* or insuffic* or stratif* or limit* or lack* or unreliab* or poor* or poverty* or depriv* or disadvantag* or insecur* or insensit* or status* or entitl* or uninform* or ill-inform* or benefit* or interven* or deliver* or effective* or cost effective*).ti,ab.	8190143	

For peer review only

9	5 and 8	761677	use of/access to health services
10	"Emigrants and Immigrants"/ or Refugees/ or "Transients and Migrants"/ or "Emigration and Immigration"/	42486	
11	(((established or "first generation*" or new* or recent* or current*) adj3 (migrant* or migrat* or immigrant* or immigrat* or emigrant* or emigrat* or emigre* or expat* or (ex adj pat*) or transient* or alien*)) or newcomer* or (new adj comer*) or incomer*	14965	Revised to focus on established or new immigrant groups; Field modified from .mp. to .ti,ab.
12	(refugee* or (asylum adj seek*) or asylee* or (refused adj3 (asylum* or refugee*)) or (displaced adj person*) or exile* or (new adj arrival) or (country adj2 (birth or origin)) or **transnational*).ti,ab.**	13603	Field modified from .mp. to .ti,ab.
13	(foreigner* or (foreign adj (born or citizen* or national* or origin*)) or (non adj (citizen* or native*)) or ((adoptive or naturali#ed) adj (citizen* or resident*)) or overstay* or trafficked or "spousal migrant*").ti,ab.	10542	Additional migrants terminology; Field modified from .mp. to .ti,ab.
14	("non-UK-born" or "born outside the UK" or "length of residence in the UK" or (("not lawful*" or "not legal*" or unlawful* or illegal* or unauthori#ed* or "not authori#ed" or uncertain or insecure or illegal or legal or irregular* or refused or **undocumented)** adj3 (residen* or immigrant* or imigrat* or migrant* or migrat*))).ti,ab.	1375	Additional migrants terminology; Field modified from .mp. to .ti,ab.
15	exp Ethnic Groups/ or (ethnic* or ethno* or race or racial*).ti,ab.	282908	Expanded ethnic terminology; Field modified
16	exp african continental ancestry group/ or exp asian continental ancestry group/ or exp Caribbean Region/	152457	Additional ethnic terminology to specify South Asian and African Caribbean groups;
17	exp Vulnerable Populations/ or ((vulnerab* or disadvantag* or minorit*) adj3 (individ* or	35822	Expanded vulnerable populations terminology; Field

18	("Black and Minority Ethnic" or "Black & Minority ethnic" or BME or african caribbean* or afro caribbean* or black african* or (west adj (indies or indian*))).ti,ab.	7587	Expanded ethnic terminology; Field modified from .mp. to .ti,ab.
19	(south asia* or afghan* or bangladesh*	163384	
20	10 or 11 or 12 or 13 or 14 or 15 or 16 or 17 or 18 or 19	589408	All ethnic/migrant groups
21	7 and 9 and 20	20754	Maternity health services AND [illegible]
22	limit 21 to yr="1990 -Current"	18783	Time range expanded
23	higginbottom*.au.	214	
24	22 and 23	9	Check of strategy retrieval of known relevant records

For peer review only

File 2: Bibliographic databases searched

Databases searched.

- Ovid MEDLINE 1948– and MEDLINE In-Process and Other Non-Indexed Citations to daily update
- Ovid EMBASE 1980–2017 Week 11
- Ovid PsycINFO 1972–March Week 3 2017
- CINAHL Plus with Full Text/EBSCOHost to 2017
- MIDIRS on Ovid 1971 to April 2017
- Thomson Reuters Web of Science* 1900–2017
- ASSIA on ProQuest 1987–current
- HMIC on Ovid 1979–January 2017
- POPline (via http:// www.popline.org/) 1970 to the present

* Thomson Reuters Web of Science 1900-2017 includes the following:

- Science Citation Index Expanded (SCI-EXPANDED) 1900–2017
- Social Sciences Citation Index (SSCI) 1956–2017
- Conference Proceedings Citation Index - Science (CPCI-S) 1990–2017
- Conference Proceedings Citation Index - Social Science and Humanities (CPCI-SSH) 1990–2017
- Book Citation Index - Science (BKCI-S) 2008–2017
- Book Citation Index - Social Science and Humanities (BKCI-SSH) 2008–2017
- Emerging Sources Citation Index (ESCI) - 2015–2017

For peer review only

List of databases for searching grey literature

- **Cochrane Database of Systematic Reviews**
  http://www.thecochranelibrary.com/

Theses

- **Nottingham eDissertations**
  http://edissertations.nottingham.ac.uk/
- selected dissertations from UoN

- **Nottingham eTheses**
  http://etheses.nottingham.ac.uk/
- research degree theses awarded by UoN
- pilot project so not compulsory to submit, therefore not all these included

- **Index to Theses**
  http://www.theses.com/
  theses (incl. abstracts) accepted for higher degrees by universities in GB and Ireland
  now part of ProQuest Dissertations & Theses – UK & Ireland

- **Networked Digital Library of Theses & Dissertations**
  http://www.ndltd.org/
- includes theses and dissertations submitted to over 200 universities worldwide

- **EThOS – British Library Electronic Theses Online**
  http://ethos.bl.uk/Home.do

- **DEEP – DART Europe**
  http://www.dart-europe.eu

- **ProQuest Dissertations & Theses A&I** (worldwide coverage)
  http://search.proquest.com/pqdt/index?accountid=8018

Research Funders

- **Wellcome Trust**
  http://www.wellcome.ac.uk/
- Global charitable foundation supporting biomedical research and the medical humanities
- Provides support with funding, managing grants, education resources, application of research

- **Research Councils UK**
  http://www.rcuk.ac.uk/
- Support research across all academic disciplines
- Offer funding opportunities, international collaborations and training

For peer review only

- **Medical Research Council**
  http://www.mrc.ac.uk/index.htm
- Publicly funded organisation dedicated to improving human health
- Supports research across medical sciences in universities, hospitals and MRC Councils

- **Science and Technology Facilities Council**
  http://www.stfc.ac.uk/
- Independent public body of the Department of Business, Innovations and Skills
- Supports researchers across the sciences with the academic and industrial communities

- **National Institute for Health and Care Excellence**
  http://www.nice.org.uk/

- **Institute for Public Policy and Research**
  http://www.ippr.org/

- **ESRC**
  http://www.esrc.ac.uk/
  REGARD database

- **Clinical Research Network** (part of the NHS National Institute for Health Research)
  http://www.crncc.nihr.ac.uk
  From the NIHR portal
  (https://portal.nihr.ac.uk/Pages/NIHRResearchInfoStatement.aspx):
  - The repository for this information is the Portfolio Database, which currently contains approximately 2,000 studies, and can be accessed for public searching. Detailed instructions on how to search the Portfolio Database are available at http://www.ukcrn.org.uk/index/clinical/portfolio_new/P_search.html, and the Portfolio database is available at http://public.ukcrn.org.uk/search.
  - The National Research Register has been archived as a public resource and to support historical analysis. The archive is available via the National Institute for Health Research Portal http://portal.nihr.ac.uk.

Statistics

- **Department of Health**
  https://www.gov.uk/government/publications?publication_filter_option=statistics

- **UK Data Archive**
  http://www.data-archive.ac.uk

- **UK National Statistics**
  http://www.statistics.gov.uk

- **OECD Statistics Portal**
  http://www.oecd.org/statistics

- **World Health Organisation**

For peer review only

http://www.euro.who.int/en

- **NICE Evidence Services** (formerly NHS Evidence)
- Evidence search
- Clinical Knowledge Summaries
- NICE guidelines
- Journals and databases
- A-Z of topics – e.g. Diabetes
- Medicines information
- Public health information

https://www.evidence.nhs.uk

- **HMIC** (Health Management Information Consortium) – on Ovid
- combined database of the Department of Health, plus the King's Fund Information & Library Service
- official publications, journal articles, grey literature
- health service policy, management & admin, quality of hospitals, nursing, primary care and public health; occupational health; control/regulation of medicines

- **PAIS International**

http://search.proquest.com/pais?accountid=8018 (via ProQuest)

- includes e.g.: gov docs, statistical directories, grey lit, research reports – mostly in social sciences

- **Open Grey**

http://www.opengrey.eu/

- open access to grey literature published in Europe, including reports, dissertations, conference proceedings, official publications

- **Mednar**

http://mednar.com/mednar/desktop/en/green/search.html
one-stop federated search engine designed for professional medical researchers

- **WorldwideScience**

http://worldwidescience.org/

- **OAIster**

http://www.oclc.org/oaister.en.html
catalog of millions of records from open access collections worldwide using the Open Archives Initiative Protocol for Metadata Harvesting (OAI-PMH)

- **Internet Archive Wayback Machine**

http://archive.org/web/
aims to provide permanent access to historical collections that exist in digital format

For peer review only

For peer review only

File 3: Inclusion/Exclusion criteria

Inclusion Criteria

Population	Immigrant women from any country other than England, Scotland, Northern Ireland or Wales
Phenomena of Interest	Maternity care
Context Setting	United Kingdom
Study designs	Qualitative, quantitative and mixed methods studies
Language	English
Date limitations	Jan 1990 - Jan 2018
**Exclusion Criteria**	
Context	Studies located in any country other than England, Scotland, Northern Ireland or Wales
Participants	Black and minority ethnic women born in the United Kingdom
Study Design	Non-empirical research, opinion pieces or editorial

For peer review only

File 4: Excluded papers and rationale

Excluded studies with reasons for exclusion.

Exclusion number	Reference	Reasons for exclusion
**1**	Bowler I. 'They're not the same as us': midwives' stereotypes of South Asian descent maternity patients. *Sociol Health Illn*. 1993 Mar 1;15(2):157-78.	Presented professionals' perspectives: focused on midwife interviews and observational data on midwives.
**2**	Straus L, McEwen A, Hussein FM. Somali women's experience of childbirth in the UK: perspectives from Somali health workers. *Midwifery*. 2 09 Apr 1;25(2):181-6.	Presented professionals' perspectives: interviewed Somali health workers and not the immigrant women.
**3**	Bowler IM. Stereotypes of women of Asian descent in midwifery: some evidence. *Midwifery*. 1993 Mar 1;9(1):7-16.	Presented professionals' perspectives: interviewed midwives.
**4**	Haith-Cooper M, Bradshaw G. Meeting the health and social needs of pregnant asylum seekers: midwifery students' perspectives. Part 2: Dominant discourses and approaches to care. *Nurse Educ Today*. 2013 Aug 1;33(8):772-7.	Presented professionals' perspectives: focused on midwifery students' perceptions.
**5**	Haith-Cooper M, Bradshaw G. Meeting the health and social care needs of pregnant asylum seekers; midwifery students' perspectives: Part 3; The pregnant woman within the global context; an inclusive model for midwifery education to address the needs of asylum-seeking women in the UK. *Nurse Educ Today*. 2013 Sep 1;33(9):1045-50.	Presented professionals' perspectives: interviewed midwives.
**6**	Balaam MC, Kingdon C, Thomson G, Finlayson K, Downe S. 'We make them feel special': the experiences of voluntary sector workers supporting asylum-seeking and refugee women during pregnancy and early motherhood. *Midwifery*. 2016 Mar 1;34:133-40.	Presented professionals' perspectives.
**7**	Richards J, Kliner M, Brierley S, Stroud L. Maternal and infant health of Eastern Europeans in Bradford, UK: a qualitative study. *Community Practitioner*. 2014 Sep 1;87(9):33.	Presented professionals' perspectives.
**8**	Redshaw M, Heikkilä K. Ethnic differences in women's worries about labour and birth. *Ethn Health*. 2011 Jun 1;16(3):213-23.	Mixed sample of UK-born BME and immigrant women with no separate findings reported for immigrant women.

**9**	Darwin Z, Green J, McLeish J, Willmot H, Spiby H. Evaluation of trained volunteer doula services for disadvantaged women in five areas in England: women's experiences. *Health Social Care Community*. 2017 Mar 1;25(2):466-77.	Mixed sample of UK-born BME and immigrant women with no separate findings reported for immigrant women.
**10**	Dunne FP, Brydon PA, Proffitt M, Smith T, Gee H, Holder RL. Fetal and maternal outcomes in Indo-Asian compared to Caucasian women with diabetes in pregnancy. *QJM*. 2000 Dec 1;93(12):813-8.	Mixed sample of Indo-Asian women born inside and outside the UK with no separate findings for immigrant women.
**11**	Ball HL, Moya E, Fairley L, Westman J, Oddie S, Wright J. Infant care practices related to sudden infant death syndrome in South Asian and White British families in the UK. *Paediatr Perinat Epidemiol*. 2012 Jan 1;26(1):3-12.	Focused on care of infants aged 2-4 months, but our chosen limit of maternity care was only up to 6 weeks after birth.<br>Mixed sample of UK-born and non-UK-born women with no separate findings for immigrant women.
**12**	McCarthy R, Haith-Cooper M. Evaluating the impact of befriending for pregnant asylum-seeking and refugee women. *Br J Midwifery*. 2013 Jun;21(6):404-9.	Not an empirical: the study does not report its methodology, sampling, or data analysis.
**13**	Streetly A, Grant C, Bickler G, Eldridge P, Bird S, Griffiths W. Variation in coverage by ethnic group of neonatal (Guthrie) screening programme in south London. *BMJ*. 1994 Aug 6;309(6951):372-4.	Mixed sample of ethnic groups with no separate findings for immigrant women.
**14**	Burchill J, Pevalin DJ. Demonstrating cultural competence within health-visiting practice: working with refugee and asylum-seeking families. *Divers Equal Health Care*. 2014;11(2).	Weak focus on maternity care: just two quotes on the influence of cultural sensitivity training and on addressing female genital mutilation (FGM) in maternity.
**15**	Dormandy E, Michie S, Hooper R, Marteau TM. Low uptake of prenatal screening for Down syndrome in minority ethnic groups and socially deprived groups: a reflection of women's attitudes or a failure to facilitate informed choices? *Int J Epidemiol*. 2005 Feb 28;34(2):346-52.	Not clear if sample was composed of immigrant women: no separate findings for immigrant women.
**16**	Henderson J, Gao H, Redshaw M. Experiencing maternity care: the care received and perceptions of women from different ethnic groups. *BMC Pregnancy Childbirth*. 2013 Dec;13(1):196.	Not clear if sample was composed of immigrant women: no separate findings for immigrant women.
**17**	Ingram J, Cann K, Peacock J, Potter B. Exploring the barriers to exclusive breastfeeding in Black and minority ethnic groups and young mothers in the UK. *Matern Child Nutr*. 2008 Jul 1;4(3):171-80.	Mixed sample of UK-born and immigrant women with no separate findings for immigrant women.

**18**	Parsons L, Day S. Improving obstetric outcomes in ethnic minorities: an evaluation of health advocacy in Hackney. *J Public Health*. 1992 Jun 1;14(2):183-91.	Not clear if sample was composed of immigrant women.
**19**	Knight M, Kurinczuk JJ, Spark P, Brocklehurst P. Inequalities in maternal health: national cohort study of ethnic variation in severe maternal morbidities. *BMJ*. 2009 Mar 4;338:b542.	Mixed sample of both UK- and foreign-born BME with no separate findings for immigrant women.
**20**	Almond P, Lathlean J. Inequity in provision of and access to health-visiting postnatal depression services. *J Adv Nurs*. 2011 Nov 1;67(11):2350-62.	Focused on professionals' perspective. Eight of the nine participants were immigrant women, but just three brief quotes were reported from immigrant Bangladeshi women. Authors did not reply to our request for clarification of the immigrant status of the sample.
**21**	Row MA, Nevill AM, Young DB, Adamson-Macedo EN. (2013) Promoting positive postpartum mental health through exercise in ethnically diverse priority groups. *Divers Equal Health Care*. 2013;10(3)185-195.	Mixed sample of minority ethnicity women born in and outside the UK with no separate findings for immigrant women.
**22**	Hemingway H, Saunders D, Parsons L. Social class, spoken language and pattern of care as determinants of continuity of carer in maternity services in east London. *J Public Health*. 1997 Jun 1;19(2):156-61.	Mixed sample of women with and without English as a first language. We used lack of English as a proxy for immigrant, but only one finding was reported for a non-English sample (i.e., the presence of an advocate who could translate for women visiting midwives or doctors). Did not receive a reply from the authors regarding the immigrant status of the sample.
**23**	Ingram J, Johnson D, Hamid N. South Asian grandmothers' influence on breast feeding in Bristol. *Midwifery*. 2003 Dec 1;19(4):318-27.	No clarity on the immigrant status of the sample and no separate findings for immigrant women. Did not receive a reply from the authors on the immigrant status of the sample.
**24**	Gardner PL, Bunton P, Edge D, Wittkowski A. The experience of postnatal depression in West African mothers living in the United Kingdom: A qualitative study. *Midwifery*. 2014 Jun 1;30(6):756-63.	No clarity on the immigrant status of the sample and no separate findings for immigrant women.
**25**	Kelly Y, Panico L, Bartley M, Marmot M, Nazroo J, Sacker A. Why does birthweight vary among ethnic groups in the UK? Findings from the Millennium Cohort Study. *J Public Health*. 2008 Jul 21;31(1):131-7.	Not clear if participants included immigrant women. Did not receive a reply from the authors on the immigrant status of the sample.

**26**	Beake S, McCourt C, Bick D. Women's views of hospital and community-based postnatal care: the good, the bad and the indifferent. *Evid Based Midwifery*. 2005 Dec 1;3(2):80-7.	Not clear if participants included immigrant women. Did not receive a reply from the authors on the immigrant status of the sample.
**27**	Ahmed S, Green J, Hewison J. Antenatal thalassaemia carrier testing: women's perceptions of information and consent. *J Med Screen*. 2005 Jun 1;12(2):69-77.	Weak focus on maternity care: main focus was on an ancestry issue.
**28**	Dyson SM, Cochran F, Culley L, Dyson SE, Kennefick A, Kirkham M, Morris P, Sutton F, Squire P. Ethnicity questions and antenatal screening for sickle cell/thalassaemia (EQUANS) in England: observation and interview study. *Crit Public Health*. 2007 Mar 1;17(1):31-43.	Not clear if participants included immigrant women.
**29**	Baker D, Garrow A, Shiels C. Inequalities in immunisation and breast feeding in an ethnically diverse urban area: cross-sectional study in Manchester, UK. *J Epidemiol Community Health*. 2011 Apr 1;65(4):346-52.	Not clear if participants included immigrant women and not focused on maternity care. Did not receive a reply from the authors on the immigrant status of the sample.
**30**	Dyson SM, Chambers K, Gawler S, Hubbard S, Jivanji V, Sutton F, Squire P. Lessons for intermediate- and low-prevalence areas in England from the Ethnicity Questions and Antenatal Screening for sickle cell/thalassaemia (EQUANS) study. *Divers Health Social Care*. 2007 Jun 1;4(2).	Not clear if participants included immigrant women and not focused on maternity care.
**31**	Sim JA, Ulanika AA, Katikireddi SV, Gorman D. 'Out of two bad choices, I took the slightly better one': Vaccination dilemmas for Scottish and Polish migrant women during the H1N1 influenza pandemic. *Public Health*. 2011 Aug 1;125(8):505-11.	Not focused on maternity care.
**32**	Wittkowski A, Zumla A, Glendenning S, Fox JR. The experience of postnatal depression in South Asian mothers living in Great Britain: a qualitative study. *J Reprod Infant Psychol*. 2011 Nov 1;29(5):480-92.	Mixed sample with only two quotes related to immigrant women.
**33**	McFadden A, Atkin K, Renfrew MJ. The impact of transnational migration on intergenerational transmission of knowledge and practice related to breast feeding. *Midwifery*. 2014 Apr 1;30(4):439-46.	Not focused on maternity care.
**34**	Datta S, Alfaham M, Davies DP, Dunstan F, Woodhead S, Evans J, Richards B. Vitamin D deficiency in pregnant women from a non-European ethnic minority population – an interventional study. *BJOG*. 2002 Aug 1;109(8):905-8.	Not clear if participants included immigrant women. Did not receive a reply from the authors on the immigrant status of the sample.

**35**	McLeish J, Redshaw M. 'I didn't think we'd be dealing with stuff like this': a qualitative study of volunteer support for very disadvantaged pregnant women and new mothers. *Midwifery*. 2017 Feb 1;45:36-43.	Mixed sample with no separate findings for immigrant women.

For peer review only

File 5: Master table of included studies

Summary of included studies

Reference	Study aim	Region	Methodology	Theory or Framework	Setting	Data analysis	Sample and mode of recruitment
109	To establish efficacy of linkworker services (an intervention) introduced for non-English-speaking Asian women in multi-racial health districts	Not specified	Quantitative survey: 21-item questionnaire	Not specified.		Qualitative: content analysis	Questionnaire to the Heads of Midwifery Services in 30 multi-racial district health authorities. 20 responded. Sample is not immigrant women, however this is an evaluation of an intervention
115	To develop a reliable and valid questionnaire to evaluate satisfaction with maternity care in Sylheti-speaking Bangladeshi women.	London.	Mixed methods: two-stage psychometric study. Firstly, a Sylheti-language questionnaire regarding Bangladeshi women's experiences of maternity services was translated and culturally adapted from an English-language questionnaire using focus groups, in-depth interviews, and iterative methods. Secondly, quantitative psychometric methods were used to field test and evaluate the acceptability, reliability, and validity of this questionnaire.	Not specified.	Not specified.	Qualitative: thematical analysis. Quantitative: validity of an instrument.	Located at four hospitals providing maternity services in London, UK. Study participants included 242 women from the London Bangladeshi communities who were in the antenatal (at least 4 months pregnant) or postnatal phase (up to 6 months after delivery). The women spoke Sylheti, a language with no accepted written form. In stage one purposive samples of 40 women in the antenatal or postnatal phase participated, along with one convenience sample of six women in the antenatal phase and three consecutive samples of 60 women in the postnatal phase. In stage two, 135 women (main sample) completed the questionnaire 2 months after delivery (82% response rate), and 50 women (retest sample) from the main sample completed a second questionnaire 2 weeks later (96% response rate).

For peer review only

88	To study the maternity care experiences of Somali refugee women in an area of West London. This article focused particularly on findings relating to the language barrier, which to a large degree underpinned or at least aggravated other problems the women experienced.	West London.	Qualitative: case study. Six semi-structured interviews and two focus groups (with six participants each).	Not specified.	Not specified.	Qualitative: thematical analysis.	Snowball sampling: 12 Somali women were selected from a larger survey involving 1400 women.
89	To undertake a qualitative study of the maternity experiences of 33 asylum seekers.	London, Plymouth, Hastings, Brighton, Oxford, Manchester, and King's Lynn.	Qualitative.	Not specified.	Home or a neutral location.	Qualitative: content analysis.	Convenience and snowball sampling of recent asylum seekers. Based on semi-structured interviews carried out in seven English cities.
116	A Sure Start local programme had funded a Bangladeshi support worker to provide bilingual breastfeeding support to childbearing Bangladeshi women, many of whom were not fluent in English. This study aimed to conduct a short evaluation of the impact of this work on the uptake and duration of breastfeeding among these women.	Tower Hamlets.	Mixed methods: the survey questionnaire included some open and closed questions about the women's intention to feed; their current feeding methods; the breastfeeding support and information they received antenatally, during the hospital stay, and postnatally; overall views on the information and support received; and some demographic details. Eleven interviews were conducted by telephone in Sylheti (a dialect that has no written format), three in English and one in Urdu (using a female family member to translate). Interviews took between 15 and 30	Not specified.	Not specified (survey conducted by telephone).	Qualitative: content analysis of a questionnaire (open and closed questions).	The two midwives and the support worker had provided breastfeeding support to 194 women during a one-year period (September 2001 to August 2002). Of these, 80 women received help from the support worker alone. The majority of these 80 women were Bangladeshi. For the evaluation, 15 women were randomly selected from these 80 women.

			minutes to complete.				
**{Rowe, 2008 #183}**	To identify any social or ethnic differences in access to antenatal care and to quantify the effects of any such differences using data collected in a survey of women's experiences of antenatal screening.	England.	Quantitative: a cross-sectional survey using a postal questionnaire.	Not specified.	Not specified.	Quantitative: cross-sectional analysis.	A stratified clustered random sampling strategy was used. Hospitals in England were stratified according to ethnic mix. To ensure inclusion of an adequate number of women from black and minority ethnicity (BME) backgrounds, hospitals with ≥ 15% of women of BME origin were oversampled. Pregnant women aged ≥16 years and receiving care in 15 participating hospitals were sent a postal questionnaire at 27–31 weeks of gestation.

110	To compare the health behaviours both antenatally (smoking and alcohol consumption) and postnatally (initiation and duration of breast feeding) of mothers who have white British or Irish heritage with those of mothers from ethnic minority groups and to examine in mothers from ethnic minority groups whether indicators of acculturation (generational status, language spoken at home, and length of residency in the UK) were associated with these health behaviours.	England.	Quantitative: a prospective nationally representative cohort study.	Not specified.	Not specified.	Quantitative: cohort study.	Stratified clustered sampling framework to over-represent mothers from ethnic minority groups and disadvantaged areas produced 6478 white British or Irish mothers and 2110 mothers from ethnic minority groups. Of those from ethnic minority groups, 681 (33%) were first generation and 55 (4%) second generation.
90	To explore and synthesise the maternity care experiences of female asylum seekers and refugees.	UK.	Qualitative: multiple exploratory longitudinal case studies that used a series of interviews, photographs taken by the women, field notes, and observational methods to contextualise data obtained during 2002 and 2003.	Theory of interactions and transformational educational theory.	Hospital settings or women's homes.	Qualitative: thematical analysis.	Women were approached if the status of 'asylum seeker' or 'refugee' was written in the hospital notes taken at their booking appointment. Fourteen women were approached, but nine women declined to participate. Five women consented, but one woman was dispersed before 20 weeks gestation and therefore was not included in the study. Of the remaining four participating women, three were asylum seekers and one was a refugee. The sampling technique was not clearly reported.

For peer review only

91	To identify key features of communication across antenatal care and whether they are evaluated positively or negatively by service users.	Central London.	Qualitative: used six focus groups of 15 participants each and conducted 15 semi-structured interviews. Non-English-speaking focus groups and interviews were conducted in standard Bengali, Sylheti, or Somali.	Not specified.	Focus groups: hospitals and university meeting rooms. Semi-structured interviews: various locations to suit the needs of the women.	Qualitative: thematical analysis.	The sampling technique was not clearly reported, but they recruited 30 pregnant women from diverse social and ethnic backgrounds affiliated with one NHS Trust (i.e., hospital) in central London. Participants were recruited within this hospital, in eight community antenatal clinics situated in socially and ethnically diverse areas, via a community parenting group for Somali women, and via a Bengali Women's Health Project. Within the hospital, participants were recruited from the antenatal waiting room (which services low- and high-risk pregnancies), the ultrasound clinic, and the glucose tolerance testing clinic.
111	To determine the pregnancy outcomes of women of similar parity and ethnic background who received antenatal care ('booked') compared those who did not ('unbooked') over a period of 18 months.	North Middlesex University Hospital (NMUH), London.	Quantitative: a retrospective cohort study from September 2006 to March 2008 comparing the socio-demographics and the foetal and maternal outcomes of pregnancies of unbooked versus booked women.	Not specified.	Not specified.	Quantitative: a retrospective cohort study.	Women who received no antenatal care or who delivered within 3 days of their initial booking visit were categorised as 'unbooked'. In each case, the woman who had delivered next on the labour ward register (matched for ethnicity and parity) and who had received antenatal care prior to the second trimester served as a comparison.
117	To explore the perspectives of first- and second-generation women of Pakistani origin on maternity care and to make recommendations for culturally appropriate support and care from maternity services.	West Midlands.	Mixed methods: a retrospective Q methodology study of Pakistani women following childbirth.	Retrospective Q method study.	Not specified.	Qualitative: Q methodology.	A purposive sampling strategy was used. Postnatal first- and second-generation Pakistani women were self-identified by their responses to information leaflets disseminated at local Children's Centres across an inner city in the West Midlands.

92	To study the relationships between Somali women and their Western obstetric care providers. The attitudes, perceptions, beliefs, and experiences of both groups were explored in relation to caesarean sections, particularly to identify factors that might lead to adverse obstetric outcomes.	Greater London.	Qualitative: in-depth individual and focus group interviews.	Framework of naturalistic enquiry, emic/etic model	Not specified.	Qualitative: emic/etic analysis.	Selected 39 Somali women by snowball sampling, 36 from the community and three purposively from a hospital.
93	To address the postulates that immigrant women experience sensitive care through the use of an ethnically congruent interpreter and that such women prefer to meet health providers of the same ethnic and gender profile when in a multi-ethnic obstetrics care setting.	Greater London.	Qualitative: in-depth individual and focus group interviews. Open-ended questions were presented by an obstetrician and an anthropologist.	Framework of naturalistic enquiry.	Not specified.	Qualitative: naturalistic inquiry.	Participants were recruited throughout Greater London between 2005 and 2006. Snowball sampling was used to recruit 36 immigrant Somali women, and another three were selected by a by purposive technique for a total of 39. A purposive technique was used to select further 11 Ghanaian women who had delivered at least one child within the British healthcare system and who were living within the study area at the time of data collection.

For peer review only

118	To evaluate a pilot mental health service for asylum-seeking mothers and babies.	UK (not clear).	Mixed methods: evaluation within a participatory action research framework.	Participatory action research framework.	Not specified.	Qualitative: thematical analysis. Quantitative: the CARE-Index.	An active outreach recruitment strategy was adopted by psychologists, who embedded themselves in a drop-in community group, the Merseyside Refugee & Asylum Seekers & Asylum Seekers Pre & Postnatal Support Group. Participants were West African women who were asylum seekers or refugee and who were either pregnant or had a young baby. They originated from The Gambia, Sierra Leone, Ivory Coast, and Nigeria. All spoke English. Their ages ranged from 17 to 32 years, and all babies were under 6 months of age at the point of initial contact, with three babies not yet born. Attendance at the 21 therapeutic group sessions ranged between 4 and 12 mothers (with their babies). Seven mothers attended a significant proportion or all group sessions. An additional six mothers attended 1-4 group sessions.
94	To apply the 'three delays' framework (developed for low-income African contexts) to a high-income Western scenario to identify delay-causing influences in the pathway to optimal facility treatment.	Greater London.	Qualitative: individual and focus group interviews.	'Three delays' framework.	Not specified.	Constructivist hermeneutic naturalistic study.	Purposive and snowball sampling was used to recruit 54 immigrant women originally from sub-Saharan regions in Africa (Somalia, Ghana, Nigeria, Senegal, and Eritrea) living in London and to recruit 32 maternal providers.

For peer review only

112	To identify predictors of late initiation of antenatal care within an ethnically diverse cohort.	Newham, East London.	Quantitative: a cross-sectional analysis of routinely collected electronic patient records from Newham University Hospital NHS Trust (NUHT).	Not specified.	Not specified.	Quantitative: cross-sectional analysis.	All women who attended their antenatal booking appointment within NUHT between 1st January 2008 and 24th January 2011 were included in this study. The main outcome measure was late antenatal booking, defined as attendance at the antenatal booking appointment after 12 weeks (+6 days) gestation. The sample included women from Somalia, Eastern Europe, Africa, the Caribbean, and South Asia.
87	To explore BME women's experiences of contemporary maternity care in England.	All over England.	Qualitative data collected from a large cross-sectional survey using three open-ended questions that encouraged participants to articulate their experience of maternity care in their own words.	Not specified.	Not specified.	Qualitative: Thematic analysis.	A random sample of 4800 women was selected using Office for National Statistics (ONS) birth registration records. The overall response rate was 63% but was only 3% from BME groups. A total of 368 women self-identified as coming from BME groups. Of those, 219 (60%) responded with open text and 132 (60%) were born outside the UK.
95	To investigate women's experiences of dispersal in pregnancy and to explore the effects of dispersal on the health and maternity care of women asylum seekers who were dispersed during pregnancy in the light of NICE guidelines on antenatal, intrapartum, and postnatal care.	London, South of England, Midlands and East of England, North West, North East, and Wales.	Qualitative: interviews were conducted with 19 women who had been dispersed during their pregnancies and with one woman kept in an Initial Accommodation Centre under a new Home Office pregnancy and dispersal guidance issued in 2012.	Not specified.	Not specified.	Qualitative (not clear).	The sampling technique was not mentioned clearly. The women interviewed came from 14 different countries and had been dispersed or relocated to or within six regions of the UK. At the time of dispersal, 14 had been awaiting a decision on their asylum claim and six had been refused asylum.

For peer review only

113	To compare the maternal and birth outcomes of Polish and Scottish women having babies in Scotland and to describe any differences in clinical profiles and service use associated with migration from Poland.	All over Scotland.	Quantitative: a population-based epidemiological study of linked maternal country of birth, maternity, and birth outcomes. Scottish maternity and neonatal records linked to birth registrations were analysed for differences in modes of delivery and pregnancy outcomes between Polish migrants and Scots. These outcomes were also compared with Polish Health Fund and survey data.	Not specified.	Not specified.	Quantitative: statistical analysis.	The study analysed 119,698 Scottish and 3105 Polish births to primiparous women in Scotland in 2004-09 using routinely collected administrative data on maternal country of birth and birth outcome.
96	To understand the multiple influences on behaviour and hence the risks to metabolic health of South Asian mothers and their unborn children, to theorise how these influences interact and build over time, and to inform the design of culturally congruent, multi-level interventions.	London boroughs, Tower Hamlets, and Newham.	Qualitative: group story-sharing sessions and individual biographical life-narrative interviews.	Multi-level ecological models.	All but four interviews were in the participants' homes.	Qualitative: phenomenology.	The study recruited from diabetes and antenatal services in two deprived London boroughs 45 women of Bangladeshi, Indian, Sri Lankan, or Pakistani origin aged 21–45 years with histories of diabetes in pregnancy. Overall, 17 women shared their experiences of diabetes, pregnancy, and health services in group discussions, and 28 women gave individual narrative interviews (facilitated by multilingual researchers). All were audiotaped, translated, and transcribed.

For peer review only

9	To understand the nature of need in superdiverse areas and to examine the emergent challenges for effective maternity service delivery in an era of superdiversity.	West Midlands.	Mixed methods: the study used a semi-structured questionnaire and held narrative interviews of newcomer women. The findings were then triangulated with interviews of professionals who regularly worked with such women.	Not specified.	Not specified.	Qualitative: systematic thematic analysis. Quantitative: triangulation of findings.	Sampling was not described clearly. However, the study used a semi-structured questionnaire that was designed in collaboration with maternity professionals and community researchers to explore the views and maternity experiences of newcomer women. Experienced multilingual female community researchers completed 82 of these questionnaires with interviewees in a range of different languages. Narrative interviews were also held with 13 women to further explore issues. The findings were triangulated with 18 interviews of professionals who regularly worked with migrant women.
98	To explore how Somali women with FGM experienced and perceived antenatal and intrapartum care in England.	Birmingham.	Qualitative: a descriptive, exploratory study using face-to-face semi-structured interviews that were audio-recorded.	Not specified.	Private room.	Qualitative: thematical analysis.	The study used convenience and snowball sampling of ten Somali women in Birmingham who had received antenatal care in England in the past 5 years.
100	To explore differences in infant thermal care beliefs between mothers of South Asian and white British origin in Bradford, UK.	Bradford District, West Yorkshire.	Mixed methods: mothers were interviewed using a questionnaire with structured and unstructured questions.	Not specified.	The women chose the location of the interview.	Qualitative: thematical analysis.	A total of 102 mothers (51 South Asian and 51 white British) were recruited in Bradford District, West Yorkshire, UK. The inclusion criteria specified infants aged 13 months or less with a parent of South Asian or white British cultural origin who lived in the Bradford District. South Asia was defined as including the countries of Pakistan, India, Afghanistan, Sri Lanka and Nepal. Recruitment was aided by local community organisations, children's centres, and community contacts. Urdu- and Punjabi-speaking interpreters were requested and provided for 69 per cent of the first-generation South Asian mothers (n = 26) in the sample.

97	To gain an understanding of infant feeding practices among a group of UK-based refugee mothers.	Liverpool and Manchester.	Qualitative: two focus group discussions and 15 semi-structured interviews.	Not specified.	HCPs: private offices or clinics Refugee women: private rooms or discrete areas at the support venue (community centre or church hall).	Qualitative: thematical analysis.	The study purposively selected 30 refugee mothers from 19 countries who now resided in Liverpool or Manchester and were at least 6 months pregnant or had a child who had been born in the UK in the last 4 years. Of these 30, 19 were HIV-negative and 11 were HIV-positive.
119	To provide insights into possible causes of poor maternity outcomes for new migrants in the West Midlands region of the UK and to develop recommendations that could help improve maternity services for these migrants.	West Midlands.	Mixed methods: a semi-structured questionnaire and in-depth interviews.	Not specified.	Not specified.	Qualitative: systematic thematical approach. Quantitative: triangulation of the findings.	A non-probability purposive sample was generated by selecting 82 women who had moved to the UK within the past 5 years and had subsequently utilised maternity services. Of these, 13 underwent in-depth interviews as well.
99	To explore the maternity care experiences of pregnant asylum-seeking women in West Yorkshire to inform service development.	West Yorkshire.	Qualitative: interpretative approach within the tradition of hermeneutic phenomenology.	Not specified.	Not specified.	Qualitative: interpretive approach with hermeneutic phenomenology analysis.	Purposive sampling was performed through the voluntary sector and a children's centre. In addition, word-of mouth led to an element of snowball sampling. Six women were recruited.
120	To provide locally applicable data on the needs of Black and minority ethnic women in relation to their uptake of maternity and neonatal care provision by primary healthcare teams in Leeds.	Leeds.	Mixed methods: questionnaires and focus groups. Interpreters were used when necessary for data collection. A questionnaire was translated into Urdu for some women.	Not specified.	Local community centres and in the participants' homes.	Qualitative: content analysis. Quantitative: survey (not clear).	A total of 97 questionnaires were completed, of which 50 were completed through informal links at community centres, schools, and in women homes. The remaining 47 were completed whilst the researcher attended various antenatal clinics in the community.

101	To study the effectiveness of three linkworker and advocacy schemes that were designed to empower minority ethnic community users of maternity services.	Birmingham.	Qualitative: focus group discussions, semi-structured interviews, and non-directive interviews.	Not specified.	Antenatal clinics in hospitals and health centres, community group settings, and participants' homes.	Qualitative: not clear, thematical analysis?	Individual interviews were conducted with 66 Asian women who had received support from linkworker and advocacy services during their pregnancy and postnatally. Of these, 28 were from Birmingham, 13 from Leeds, and 25 from Wandsworth-London. A semi-structured interview guide was translated into five Asian languages: Hindi, Punjabi, Gujarati, Urdu and Tamil. The study also included ten focus groups made up of 60 women who had not used linkworker or advocacy services. All participants were recruited with the help of various minority ethnic women's groups and community organisations. Interpreters assisted 11 personal interviews with non-users from Vietnamese and Chinese backgrounds.
62	To determine the nature of the barriers confronting women when they used antenatal and postnatal services.	Pollokshields, Glasgow.	Qualitative: semi-structured questionnaire.	Not specified.	Not specified.	Qualitative: thematic analysis.	Twenty women were interviewed in depth by a Centre's Health Development Worker. Of these, 17 were born outside the UK.
102	To study the maternity services experiences of Muslim parents in England.	UK: not specified.	Qualitative: focus groups with Muslim mothers to explore their experiences of and views about maternity services; questionnaires with Muslim fathers; and interviews with health professionals	Not specified.	Not specified.	Qualitative: content analysis.	A mixed sample of 43 immigrants and non-immigrants were recruited via their project advisory groups. The focus groups were conducted in various locations around the UK, with two focus group discussions in a language other than English. A total of eight health professionals were interviewed: six midwives (two of whom worked for Sure Start programmes), a health visitor, and a consultant obstetrician.

For peer review only

114	To determine the current clinical practice of maternity care in England, including the service provision and organisations that underpin care, from the perspective of women needing the care; to identify the key areas of concern for women receiving maternity care in England; and to determine whether and in what ways women's experiences and perceptions of care have changed over the last 10 years.	England: not specified.	Quantitative: survey.	Not specified.	Survey: not specified.	Quantitative: cross-sectional design.	Random samples of women selected for the pilot and main studies were identified by staff at the ONS using live birth registrations for births within 2 specific weeks: 2–8 January (pilot) and 4–10 March 2006 (main study). The same method of sampling was used as had been employed in 1995 to enable direct comparisons. Random samples of 400 women for the pilot survey and 4800 women for the main survey who were aged 16 years and over and who had delivered their baby in a one week period in England were selected. The sampling was stratified on the basis of births in different geographical areas (Government Office Regions (GORs)). No subgroups were oversampled. The usable response rate was 60% for the pilot survey and 63% for the main survey. The samples included 229 women of BME born outside the UK.

For peer review only

103	To explore the perceptions of pregnant asylum seekers in relation to the provision of their maternity care while in emergency accommodation in the UK.	South East of England.	Qualitative: an exploratory approach using unstructured interviews with five healthcare professionals and semi-structured interviews with ten pregnant asylum seekers.	Not specified.	Participants' emergency accommodations.	Qualitative: thematical analysis.	Purposive sampling of those providing maternity care for asylum seekers produced a sample comprising two midwives (M1 and M2), one GP (GP), one hospital consultant (C), and one nurse (N), all based in south coast health centres and hospitals. A total of 15 pregnant asylum seekers were approached to participate in the study. These women entered the UK through a south coast port over a three-month period. Their countries of origin were Algeria, Congo, Angola, Nigeria, Somalia, and Iraq, and they spoke French, Portuguese, Yoruba, Arabic, and Kurdish. Translated information letters and consent forms were distributed to pregnant asylum seekers via the Refugee Help Line, which also returned signed consent forms. This constitutes non-probability, purposive sampling.
104	To explore the meanings attributed by migrant Arab Muslim women to their experiences of childbirth in the UK. In particular, to explore migrant Arab Muslim women's experiences of maternity services in the UK; to examine the traditional childbearing beliefs and practices of Arab Muslim society; and to suggest ways to provide culturally sensitive care for this group of women.	UK: not specified.	Qualitative: an interpretive ontological-phenomenological perspective informed by the philosophical tenets of Heidegger (1927/1962).	Heideggerian hermeneutic phenomenology.	All interviews were in the participants' homes except for one, which took place in a restaurant after 10 pm.	Qualitative: thematical analysis.	Purposive sampling produced eight Arab Muslim women who had migrated to one multicultural city in the Midlands.

For peer review

105	To examine the health-seeking behaviours of Korean migrant women living in the UK.	London.	Qualitative: 21 semi-structured interviews.	Foucauldian approach.	Not clear.	Qualitative: not clear.	Women were recruited from New Malden via Korean community contacts.
121	To explore perinatal clinical indicators and experiences of postnatal care among European and Middle Eastern migrant women and to compare them with those of British women at a tertiary hospital in the North East of Scotland.	North East of Scotland.	Mixed methods. Phase 1 of the research was a secondary analysis of routine data for 15,030 consecutive deliveries at Aberdeen Maternity Hospital. Phase 2 was a retrospective study of 26 European, Middle Eastern, and British mothers in this hospital. After the women had given birth, verbal data was collected using face-to-face semi-structured interviews.	Not clear.	Phase Two: 24 interviews were conducted in the homes of participants and two interviews at the University department.	Qualitative: thematical analysis. Quantitative: Phase One was a secondary analysis of routine data for 15,030 consecutive deliveries at Aberdeen Maternity Hospital. Phase 2 was a retrospective study of women.	Phase 1: The 15,030 deliveries included all births at Aberdeen Maternity Hospital over the financial years 2004–2008 in which maternal nationalities were identified and gestation was ≥ 24 weeks. Both singleton and multiple births were included. The clinical data was harvested from the Patient Administration System and the PROTOS maternity information system. In the case of women with multiple order births during the study, all births were included. Phase 2 of the research was a retrospective study of a few of the mothers who had given birth at this hospital. Eight European and five Middle Eastern women were semi-matched with 13 British women.

For peer review only

122	To assess the mechanisms of support available to EM (ethnic minority) communities from community and voluntary sector organisations in relation to maternal and infant nutrition (a mapping exercise); to explore the experiences of the targeted client groups in seeking and receiving such support; and to identify gaps and opportunities to enhance support mechanisms and engagement with diverse EM communities.	Glasgow, Edinburgh, Aberdeen, Stirling, Fife, Dundee, and Inverness.	Mixed methods: an online questionnaire survey of organisations working with EM communities, focus groups, and telephone interviews with EM women.	Not specified.	Not specified.	Qualitative: thematical analysis. Quantitative:	The study identified 65 community organisations that potentially provided food and health services across EM communities in Scotland. In total, 37 organisations replied to the survey. Of those organisations, 15 indicated that they are providing services in the area of maternal and infant nutrition. A further 12 indicated that despite working with EM communities, they do not provide services in maternal and infant nutrition or healthy eating in general. An additional ten organisations confirmed by telephone that they were or had been working with EM women, but were unable to undertake the survey. The majority of interviewees for the focus groups and interviews were selected in response to a request sent by Black and Ethnic Minorities Infrastructure in Scotland (BEMIS) to community organisations. Snowball sampling was used to provide further contacts. In total, four focus groups were conducted with Polish, Roma, Czech, and African mothers. In addition, six telephone interviews were conducted with Polish mothers. We focused on Polish mothers because they were the largest new ethnic group in Scotland since 2004.

For peer review only

106	To explore the experiences of obstetric care in Scotland among women who have undergone FGM.	Glasgow and Edinburgh.	Qualitative: personal experiences of FGM and interviews.	Interpretivism paradigm and feminist perspective.	The Dignity Alert & Research Forum (DARF) office or in the participant's home.	Qualitative: thematical analysis.	Convenience and purposive sampling resulted in a total number of seven women taking part in this study. All women were of African origin living in Scotland (three in Glasgow and four in Edinburgh). The inclusion criteria for the study were women who have undergone FGM and had experienced childbirth in Scotland. Three women were originally from Somalia, two from The Gambia, one from Ghana, and one from Sudan. Six of them were Muslims and one was Christian. All women had undergone FGM in their countries of origin. Four women had been infibulated and the remaining three could not tell if they have had FGM type 2 or 3.
107	To gain a rich understanding of migrant Pakistani Muslim women's experiences of postnatal depression within motherhood; to inform clinical practice; and to suggest ways of improving supportive services for this group.	East London.	Qualitative: interpretative phenomenology.	Interpretative phenomenological analysis (IPA) theory.	Not specified.	Qualitative: interpretative phenomenology.	Purposive sampling resulted in the recruitment of four migrant Pakistani Muslim women from London aged from 27 to 39.
41	To explore the healthcare experience of vulnerable pregnant migrant women.	London.	Mixed methods: participants were contacted by phone (using a three-way interpreter call if appropriate) and interviewed using a pro forma questionnaire designed to determine their access to antenatal care; barriers to that access; and their experiences during pregnancy, labour, and the immediate postnatal period. Further data was extracted from their records at the Doctors of the	Not specified.	Phone survey.	Qualitative: thematical analysis. Quantitative: not clear.	Pregnant women who presented to the drop-in clinic of the DOTW in London were approached between January 2013 and June 2014.

For peer review only - http://bmjopen.bmj.com/site/about/guidelines.xhtml

			World (DOTW) clinic to see how they had accessed the clinic.				
108	To explore relationships between first-generation migrant Pakistani women and midwives in the South Wales region, focusing on the factors that contribute to these relationships and the ways that these factors might affect the women's experiences of care.	South Wales.	Qualitative: a focused ethnography.	Symbolic interactionism.	Midwives: at lunch break or between clinics. Pakistani women: not clear.	Qualitative: thematical analysis.	Purposive sampling, through midwife gatekeepers, was selected for the initial recruitment of pregnant migrant Pakistani women: emails were sent to all midwives working with migrant women in South Wales. Snowballing was then used to recruit other midwives eligible for participation. Focused, non-participant observations of antenatal booking appointments took place in antenatal clinics across the local health board region over a period of 3-6 months. A total of seven midwives and 15 women were observed during these appointments, which lasted 20-60 minutes each.

eer review only

For peer review only - http://bmjopen.bmj.com/site/about/guidelines.xhtml

For peer review only

File 6: Characteristics of study participants

Participants country of origins

Studies	Country of origins
Duff, L. A., Ahmed, L. B., & Lamping, D. L. (2002).	Bangladesh
Ahmed, S., Macfarlane, A., Naylor, J., & Hastings, J. (2006).	Bangladesh
Cross-Sudworth, F., Williams, A., & Herron-Marx, S. (2011).	Pakistan
Greenhalgh,T., Clinch, M., Afsar, N., Choudhury, Y., Sudra, R., Campbell-Richards, D., & Finer, S. (2015).	Bangladesh, Indian, Sri Lanka, Pakistan
de Chavez, A. C., Ball, H. L., & Ward-Platt, M. (2016).	South Asia including Pakistan, India, Afghanistan, Sri Lanka, Nepal
Hicks, C., & Hayes, L. (1991).	Asian Sub-Continent
Harper Bulman, K., & McCourt, C. (2002).	Somalia
Essen et al. (2011).	Somalia
Moxey, J. M. &Jones, L.L. (2016).	Somalia
Raine, R., Cartwright, M., Richens, Y., Mahamed, Z., & Smith, D. (2010).	Somalia and Bengal
Binder, P., Borne, Y., Johnsdotter, S., & Essen, B. (2012).	Somalia and Ghana
Cresswell, J. A., Yu, G., Hatherall, B., Morris, J., Jamal, F., Harden, A., & Renton, A. (2013).	Somalia, Eastern European, African, Caribeean, South Asia
Binder, P., Johnsdotter, S., & Essen, B. (2012).	Somalia, Ghana, Nigeria, Eritrea and Senegal
O'Shaughnessy, R., Nelki, J., Chiumento, A., Hassan, A., & Rahman, A. (2012).	Gambia, Sierre Leone, Ivory Coast and Nigeria
McLeish, J. (2005).	Black African origin, other not specified

For peer review only

Gorman, D. R., Katikireddi, S. V., Morris, C., Chalmers, J. W. T., Sim, J., Szamotulska, K., . . . Hughes, R. G. (2014).	Poland
Rowe, R. E., Magee, H., Quigley, M. A., Heron, P., Askham, J., & Brocklehurst, P. (2008).	Not specified
Hawkins, S. S., Lamb, K., Cole, T. J., & Law, C. (2008).	Not specified
Briscoe, L., & Lavender, T. (2009).	Not specified
Tucker, A., Ogutu, D., Yoong, W., Nauta, M., & Fakokunde, A. (2010).	Not specified
Jomeen, J., & Redshaw, M. (2013).	Not specified
Feldman, R. (2014).	Not specified
Phillimore, J. (2015).	Not specified
Hufton, E., & Raven, J. (2016).	Not specified
Phillimore, J. (2016).	Not specified
Lephard, E., & Haith-Cooper, M. (2016).	Not specified
**Grey literature**	
Goodwin, L. (2016).	Pakistan
Lamba, R. (2015).	Pakistan-muslim
Bawadi, H. (2009).	Arab muslims
Lee, Jeung Yeon (2010).	Korean
BEMIS SCOTLAND in partnership with Community Food and Health (Scotland). (2013).	Poland, Roma, Czech and African
Almalik, M. (2011).	Europe and Middle East
Baldeh, F. (2013).	Somalia, Gambia, Ghana, Sudan
Nabb, J. (2006).	Algeria, Congo, Angola, Nigeria, Somalia and Iraq
Leeds Family Health. (1992).	Not specified

For peer review only

Warrier, S. (1996).	Asian
Pershad, P., Tyrrell, H. (1995).	Not specified
Ali, N. (2014).	Not specified
Redshaw et al. (2006).	Not specified
Shortall, C., et al. (2015).	Not specified

Antenatal & postnatal

**PEER REVIEWED**	
**Antenatal**	
Rowe, R. E., Magee, H., Quigley, M. A., Heron, P., Askham, J., & Brocklehurst, P. (2008).	Antenatal
Raine, R., Cartwright, M., Richens, Y., Mahamed, Z., & Smith, D. (2010).	Antenatal
Binder, P., Johnsdotter, S., & Essen, B. (2012).	Antenatal
Cresswell, J. A., Yu, G., Hatherall, B., Morris, J., Jamal, F., Harden, A., & Renton, A. (2013).	Antenatal
Feldman, R. (2014).	Antenatal
Greenhalgh,T., Clinch, M., Afsar, N., Choudhury,Y., Sudra, R., Campbell-Richards, D. & Finer, S. (2015).	Antenatal
Moxey, J. M. & Jones, L.L. (2016).	Antenatal
**Ante, intrapartum & postnatal**	
Cross-Sudworth, F., Williams, A., & Herron-Marx, S. (2011).	Ante, intrapartum & postnatal
Duff, L. A., Ahmed, L. B., & Lamping, D. L. (2002).	Ante, intrapartum & postnatal
McLeish, J. (2005).	Ante, intrapartum & postnatal
Lephard, E., & Haith-Cooper, M. (2016).	Ante, intrapartum & postnatal

For peer review only - http://bmjopen.bmj.com/site/about/guidelines.xhtml

Harper Bulman, K., & McCourt, C. (2002).	Ante, intrapartum & postnatal
Briscoe, L., & Lavender, T. (2009).	Ante, intrapartum & postnatal
Phillimore, J. (2015).	Ante, intrapartum & postnatal
Phillimore, J. (2016).	Ante, intrapartum & postnatal
Essen & al. (2011).	Ante, intrapartum & postnatal
**Postnatal**	
O'Shaughnessy, R., Nelki, J., Chiumento, A., Hassan, A., & Rahman, A. (2012).	Postnatal
Gorman, D. R., Katikireddi, S. V., Morris, C., Chalmers, J. W. T., Sim, J., Szamotulska, K., . . . Hughes, R. G. (2014).	Postnatal
de Chavez, A. C., Ball, H. L., & Ward-Platt, M. (2016).	Postnatal
Ahmed, S., Macfarlane, A., Naylor, J., & Hastings, J. (2006).	Postnatal
Hufton, E., & Raven, J. (2016).	postnatal
**Intrapartum & postnatal**	
Jomeen, J., & Redshaw, M. (2013).	Intrapartum & postnatal
**Antenatal & postnatal**	
Tucker, A., Ogutu, D., Yoong, W., Nauta, M., & Fakokunde, A. (2010).	Antenatal & postnatal
**Not clear**	
Hicks, C., & Hayes, L. (1991).	Not clear

GREY LITERATURE	
**Antenatal**	
Leeds Family Health. (1992).	Antenatal
Goodwin, L. (2016).	Antenatal
**Ante, intrapartum & postnatal**	
Ali, N. (2004).	Ante, intrapartum & postnatal
Bawadi, H. (2009)	Ante, intrapartum & postnatal
Lee, Jeung Yeon (2010).	Ante, intrapartum & postnatal
Baldeh, F. (2013).	Ante, intrapartum & postnatal
Shortall, C., et al (2015).	Ante, intrapartum & postnatal
BEMIS SCOTLAND in partnership with Community Food and Health (Scotland). (2013).	Ante, intrapartum & postnatal
Warrier, S. (1996)	Ante, intrapartum & postnatal
Redshaw et al. (2006).	Ante, intrapartum & postnatal
**Ante & postnatal**	
Almalik, M. (2011).	Ante & postnatal
**Postnatal**	
Lamba, R. (2015)	postnatal

Immigrant category

PEER REVIEWED	
**Refugees**	
Harper Bulman, K., & McCourt, C. (2002).	refugees
Binder, P., Borne, Y., Johnsdotter, S., & Essen, B. (2012).	refugees

For peer review only

Moxey, J. M. & Jones, L.L. (2016).	refugees
Hufton, E., & Raven, J. (2016).	refugees
**Asylum seekers**	
Feldman, R. (2014).	Asylum seekers
Lephard, E., & Haith-Cooper, M. (2016).	Asylum seekers
O'Shaughnessy, R., Nelki, J., Chiumento, A., Hassan, A., & Rahman, A. (2012).	Asylum seekers
McLeish, J. (2005).	Asylum seekers
**Immigrant category not clear**	
Duff, L. A., Ahmed, L. B., & Lamping, D. L. (2002).	Immigrant category not clear
Ahmed, S., Macfarlane, A., Naylor, J., & Hastings, J. (2006).	Immigrant category not clear
Rowe, R. E., Magee, H., Quigley, M. A., Heron, P., Askham, J., & Brocklehurst, P. (2008).	Immigrant category not clear
Hawkins, S. S., Lamb, K., Cole, T. J., & Law, C. (2008).	Immigrant category not clear
Tucker, A., Ogutu, D., Yoong, W., Nauta, M., & Fakokunde, A. (2010).	Immigrant category not clear
Cross-Sudworth, F., Williams, A., & Herron-Marx, S. (2011).	Immigrant category not clear
Raine, R., Cartwright, M., Richens, Y., Mahamed, Z., & Smith, D. (2010).	Immigrant category not clear
Jomeen, J., & Redshaw, M. (2013).	Immigrant category not clear
Gorman, D. R., Katikireddi, S. V., Morris, C., Chalmers, J. W. T., Sim, J., Szamotulska, K., . . . Hughes, R. G. (2014).	Immigrant category not clear
Greenhalgh,T.,Clinch, M., Afsar, N., Choudhury,Y., Sudra, R., Campbell-Richards, D., & Finer, S. (2015).	Immigrant category not clear
de Chavez, A. C., Ball, H. L., & Ward-Platt, M. (2016)	Immigrant category not clear

For peer review only

Hicks, C., & Hayes, L. (1991).	Immigrant category not clear
Binder, P., Johnsdotter, S., & Essen, B. (2012).	Immigrant category not clear
**Asylum seekers and refugees**	
Briscoe, L., & Lavender, T. (2009).	Asylum seekers and refugees
**Mixed migrant categories**	
Cresswell, J. A., Yu, G., Hatherall, B., Morris, J., Jamal, F., Harden, A., & Renton, A. (2013)	Mixed migrants categories
Phillimore, J. (2016).	Mixed migrant categories
Phillimore, J. (2015).	Mixed migrant categories
Essen, & al. (2011).	Mixed migrant categories

For peer review only

PRISMA 2009 Checklist

Section/topic	#	Checklist item	Reported on page #
**TITLE**			
Title	1	Identify the report as a systematic review, meta-analysis, or both.	1
**ABSTRACT**			
Structured summary	2	Provide a structured summary including, as applicable: background; objectives; data sources; study eligibility criteria, participants, and interventions; study appraisal and synthesis methods; results; limitations; conclusions and implications of key findings; systematic review registration number.	0
**INTRODUCTION**			
Rationale	3	Describe the rationale for the review in the context of what is already known.	2
Objectives	4	Provide an explicit statement of questions being addressed with reference to participants, interventions, comparisons, outcomes, and study design (PICOS).	4 & 6
**METHODS**			
Protocol and registration	5	Indicate if a review protocol exists, if and where it can be accessed (e.g., Web address), and, if available, provide registration information including registration number.	Yes https://bmjopen.bmj.com/content/bmjopen/7/7/e016988.full.pdf
Eligibility criteria	6	Specify study characteristics (e.g., PICOS, length of follow-up) and report characteristics (e.g., years considered, language, publication status) used as criteria for eligibility, giving rationale.	4 & 6
Information sources	7	Describe all information sources (e.g., databases with dates of coverage, contact with study authors to identify additional studies) in the search and date last searched.	Supplementary files
Search	8	Present full electronic search strategy for at least one database, including any limits used, such that it could be repeated.	Supplementary file
Study selection	9	State the process for selecting studies (i.e., screening, eligibility, included in systematic review, and, if applicable, included in the meta-analysis).	7 and Supplementary files

For peer review only

PRISMA 2009 Checklist

Data collection process	10	Describe method of data extraction from reports (e.g., piloted forms, independently, in duplicate) and any processes for obtaining and confirming data from investigators.	8
Data items	11	List and define all variables for which data were sought (e.g., PICOS, funding sources) and any assumptions and simplifications made.	6
Risk of bias in individual studies	12	Describe methods used for assessing risk of bias of individual studies (including specification of whether this was done at the study or outcome level), and how this information is to be used in any data synthesis.	9,10,11

n

Section/topic	#	Checklist item	Reported on page #
Risk of bias across studies	15	Specify any assessment of risk of bias that may affect the cumulative evidence (e.g., publication bias, selective reporting within studies).	9,10,11
Additional analyses	16	Describe methods of additional analyses (e.g., sensitivity or subgroup analyses, meta-regression), if done, indicating which were pre-specified.	11
**RESULTS**			
Study selection	17	Give numbers of studies screened, assessed for eligibility, and included in the review, with reasons for exclusions at each stage, ideally with a flow diagram.	Supplementary files and Prisma diagram
Study characteristics	18	For each study, present characteristics for which data were extracted (e.g., study size, PICOS, follow-up period) and provide the citations.	Supplementary file – Table of included studies
Risk of bias within studies	19	Present data on risk of bias of each study and, if available, any outcome level assessment (see item 12).	Supplementary files
Results of individual studies	20	For all outcomes considered (benefits or harms), present, for each study: (a) simple summary data for each intervention group (b) effect estimates and confidence intervals, ideally with a forest plot.	n/a

For peer review only

For peer review only - http://bmjopen.bmj.com/site/about/guidelines.xhtml

PRISMA 2009 Checklist

Synthesis of results	21	Present results of each meta-analysis done, including confidence intervals and measures of consistency.	n.a
Risk of bias across studies	22	Present results of any assessment of risk of bias across studies (see Item 15).	11
Additional analysis	23	Give results of additional analyses, if done (e.g., sensitivity or subgroup analyses, meta-regression [see Item 16]).	11
**DISCUSSION**			
Summary of evidence	24	Summarize the main findings including the strength of evidence for each main outcome; consider their relevance to key groups (e.g., healthcare providers, users, and policy makers).	14
Limitations	25	Discuss limitations at study and outcome level (e.g., risk of bias), and at review-level (e.g., incomplete retrieval of identified research, reporting bias).	19
Conclusions	26	Provide a general interpretation of the results in the context of other evidence, and implications for future research.	19
**FUNDING**			
Funding	27	Describe sources of funding for the systematic review and other support (e.g., supply of data); role of funders for the systematic review.	1

From: Moher D, Liberati A, Tetzlaff J, Altman DG, The PRISMA Group (2009). Preferred Reporting Items for Systematic Reviews and Meta-Analyses: The PRISMA Statement. PLoS Med 6(7): e1000097. doi:10.1371/journal.pmed1000097

For more information, visit: **www.prisma-statement.org**.

Page 2 of 2

For peer review only

PRISMA 2009 Checklist

For peer review only

PRISMA 2009 Checklist

For peer review only

BMJ Open

Experience of and access to maternity care in the United Kingdom (UK) by immigrant women: a narrative synthesis systematic review

Journal:	*BMJ Open*
Manuscript ID	bmjopen-2019-029478.R1
Article Type:	Original research
Date Submitted by the Author:	12-Jul-2019

Complete List of Authors:	Higginbottom, Gina; University of Nottingham School of Health Sciences, School of Health Sciences<br>Evans, Catrin; University of Nottingham School of Health Sciences, School of Health Sciences<br>Morgan, Myfanwy; King's College London, Institute of Pharmaceutical Science<br>Bharj , Kuldip; University of Leeds, UK, Faculty of Medicine and Health<br>Eldridge, Jeanette; University of Nottingham School of Health Sciences, Research and Learning Services,<br>Hussain, Basharat; University of Nottingham School of Health Sciences,

**Primary Subject Heading**:	Obstetrics and gynaecology
Secondary Subject Heading:	Health services research, Health policy, Communication
Keywords:	systematic review, narrative synthesis, immigrant women, maternity care, navigation and access

SCHOLARONE™
Manuscripts

Final

Experience of and access to maternity care in the United Kingdom (UK) by immigrant women: a narrative synthesis systematic review

Gina Marie Awoko Higginbottom,[1]* Catrin Evans,[1] Myfanwy Morgan,[2] Kuldip Kaur Bharj,[3] Jeanette Eldridge,[1] Basharat Hussain[1]

[1] Faculty of Medicine and Health Sciences, Queen's Medical Centre, University of Nottingham, Lenton Boulevard, Nottingham, NG7 2HA, UK

[2] Institute of Pharmaceutical Science, Faculty of Life Sciences and Medicine, King's College London, UK

[3] Division of Midwifery, University of Leeds, Leeds, UK

For peer review only

ABSTRACT [300 words]

One in four births in the UK is to foreign-born women. In 2016 the figure was 28.2% the highest figure on record and maternal and perinatal mortality are disproportionately higher. Our objective was to use a narrative synthesis approach to a systematic review of empirical research that focused on access and experience of maternity care by immigrant women.

Review methods

A research librarian designed the search strategies (retrieving literature published from 1990 to end June 2017). We retrieved 45,954 citations and used a screening tool to identify relevance. We searched for grey literature reported in databases and websites. We contacted stakeholders with expertise to identify additional research.

Results

We identified 40 studies (22 qualitative, 8 quantitative, and 10 mixed method)for inclusion. Immigrant women, particularly asylum seekers, often booked and access antenatal care later than the recommended first 10 weeks. Primary factors included limited English language proficiency, lack of awareness of availability of the services, lack of understanding of the purpose of antenatal appointments, immigration status, and income barriers. Maternity care experiences were both positive and negative. Women with positive perceptions described healthcare professionals as caring, confidential, and openly communicative in meeting their medical, emotional, psychological, and social needs. Those with negative views perceived that health professionals had been rude, discriminatory, and insensitive to their cultural and social needs. These women therefore avoided continuously utilising maternity care. We found few interventions that had focused on improving maternity care and the effectiveness of existing interventions have not been scientifically evaluated

Conclusions

The experiences of immigrant women in accessing and using maternity care services were both positive and negative. Further education and training of health professionals in meeting the challenges of a super diverse population may enhance quality of care and perceptions and experiences of maternity care by immigrant women..

Word Count: 299

For peer review only - http://bmjopen.bmj.com/site/about/guidelines.xhtml

Final

STRENGTHS AND LIMITATIONS OF THIS STUDY

Immigration is an international phenomenon and this review increases understanding of how immigrant women navigate maternity services in the UK

The review systematically maps our positive and negative aspects of maternity care provision as experienced by immigrant women

The review provides strategic direction for enhancement of maternity care services

The review does not address the experiences of maternity care for second-generation women (e.g. women of black and minority origin born in the UK)

SUPPLEMENTARY FILES

- Original protocol
- Supplementary files 1 -6

FUNDING STATEMENT

The NIHR HS&DR Programmed funded this project: The views expressed in this report are those of the authors and not necessarily those of the NHS, the NIHR or the Department of Health and Social Care (15/55/03).

PROSPERO 2015: CRD42015023605 Available from http://www.crd.york.ac.uk/PROSPERO/display_record.asp?ID=CRD420150

Professor Gina Higginbottom, Faculty of Medicine and Health Sciences, Queen's Medical Centre, Nottingham, NG7 2UH, UK
Tel: 0115 82 30991
Email: gawoko@hotmail.com

Declared competing interests of authors: none

Keywords: maternity, immigration, interventions, access to services, systematic review, narrative

For peer review only - http://bmjopen.bmj.com/site/about/guidelines.xhtml

Final

INTRODUCTION

The UK is in a period of superdiversity that is characterised by an increased number of new, small and scattered, multiple-origin, transnationally connected, socio-economically differentiated and legally stratified immigrants"' (Vertovec, 2007, p1024)[1] This presents challenges for the delivery and configuration of maternity services in a situation where one in four births in the UK is to foreign-born women [3]and of achieving equality of provision which forms a key aim of the NHS in the UK.[2] . Without the delivery of culturally appropriate and culturally safe maternal care, negative event trajectories may occur ranging from simple miscommunications to life-threatening incidents, [7-9] risking increased maternal and perinatal mortality. Indeed, immigrant women Significantly, immigrant women appear disproportionately in confidential inquiries into maternal and perinatal mortality,[4] perhaps indicating possible deficits in the delivery of care, access and utilisation.. To inform service delivery and address these shortcomings a coherent evidence base is required. Our review contributes to this by synthesising knowledge related to maternity care access and interventions to appropriately configure interventions as per the NHS Midwifery 2020 vision,[5] to guide professional development of healthcare professionals (HCPs), and to reshape care to ensure culturally safe and congruent maternity care that will not only benefit immigrant women but also improve the health of future generations in the UK.[3, 4, 6] While recent reviews have focused on specific aspects of maternity care, [10, 11] they have not considered a comprehensive conceptualisation of access [12] or the current super diversity. [1, 2] Reconfiguration and redesign of NHS maternal services to meet the needs of immigrant women requires integration of all these aspects.

Considering the global context, some commonality exists between high income nations in the maternity care experiences of immigrant women: studies in the United States, [13] Canada, [11]Australia, [14, 1 5]Sweden,[16, 17] and Germany [8, 18] all provided evidence of this in earlier international reviews led by Higginbottom [7, 19] Gagnon and Small. [11] However, the international comparative reviews by Gagnon focused on specific populations (South Asian and Somali) women in the UK[11], thus focusing on established immigrant groups are not the more recent super diverse patterns of migration. We have addressed this deficit in our current review

CONCEPTUAL DEFINITIONS

There is no consensus definition in the UK regarding the definition of the term 'immigrant' in different data sources and datasets relating to migration. The terms immigrant and migrant are frequently used interchangeably whilst conveying the same meaning. **Country of birth** is used by The Annual Population Survey (APS) of workers and Labour Force Survey (LFS) as a precursor for defining a 'migrant'. This survey therefore declares a person born outside the

For peer review only - http://bmjopen.bmj.com/site/about/guidelines.xhtml

Final

UK is classified as a 'migrant'. Noteworthy is the fact that workers born outside the UK may become British citizens with increasing residence in the UK.

A second source of data on migrants is applications made for obtaining a National Insurance Number, this differs from the former in definition in that the term migrant is conferred on the on the basis of **nationality**. Meaning all applicants that hold nationality other than the UK are considered migrants. However, the situation is dynamic in that the nationality of a person is may also to change over time and in some cases individuals may acquire dual-citizenship involving several nation states.

A third and significant source of data on migrants is the Office for National Statistics (ONS). ONS utilise a different strategy of classification focusing on the notion of short-term international migrant and long-term international migrant. In this definition the term 'long-term' refers to holding, the intention of residing longer than a year, whereas short-term is intention of residing less than a year. The implication of this is that the ONS considers **length of stay** of a person in the UK critical in determining migrant status. The United Nations (UN) recommend the classification of migrant into short and long term. Additionally, ONS utilises the UN definition of long-term international migrant. Accordingly, *"a migrant is someone who changes his or her country of usual residence for a period of at least a year, so that the country of destination effectively becomes the country of usual residence.*[20] In long-term international migration data, students and asylum seekers are also included which is not the case in the US.

Immigrants and the UK National Health Service (NHS)

In respect of service provision, the NHS adheres to the mandates set by central government that determines immigrant's entitlement to free NHS care. These mandates are concerned with the immigrant status and the type of service provision.[21] Within these mandates, an asylum seeker woman may not be entitled to full maternity care because of immigration status.[22] Moreover, data collection with the NHS on this topic is not well established nor comprehensive. Currently, the NHS usually collects data on ethnicity and nationality and not on the migration- related variables such as length of stay, and country of origin, etc.

The National Institute of Clinical Excellence (NICE) which provides clinical guidelines for healthcare practice in the UK i. NICE (2010).[23] in its guidelines on *Pregnancy and complex social factors: a model for service provision for pregnant women with complex social factors* identified recent migrant women having complex social needs. Within the NICE definition, a recent migrant woman is a woman has who moved to the UK within the previous 12 months. The term migrant is used generically conflating migrant women of all classifications e.g. economic migrants, asylum seekers, refugees, and those lacking English language proficiency. This suggests that there is implicit acceptance of the term migrant women in healthcare in respect of being born outside the UK, being subject to immigration regulations, with possible challenges in English language proficiency.

The operational definition of an immigrant women used in this review

For peer review only - http://bmjopen.bmj.com/site/about/guidelines.xhtml

Final

The preceding paragraphs suggests that the term 'immigrant' is defined in various ways in different countries and by different authors. However, two features are frequently referred to in these definitions. I.e. 'country of birth' and 'length of stay'. These factors are also noted by NICE guidelines on provision of maternity care. [23] cited above. These two characteristics are also important in the entitlement, access and ability to use healthcare in the UK. For example, if you are born outside the UK, it is unlikely that you are knowledgeable about the UK healthcare provision.

We adopted the following definition of an immigrant woman for the purposes of our review, and most importantly, to inform our inclusion and exclusion criteria. We defined a woman as an immigrant if she was:

- Born outside the UK, and;
- Was living in the UK for more than 12 months or had the intention to live in the UK for 12 (or more) months when first entered.

Therefore, we included studies on immigrant women where the population studied fulfills the above two characteristics. According to this definition studies on population groups of foreign students, asylum seekers, recent legal refugees, and immigrants, illegal immigrants, will also be eligible for inclusion. In many cases, the study populations/sample may not be accurately and fully described. We therefore used linguistic ability e.g. the need for an interpreter as a proxy for immigrant status. Notwithstanding all of these perspectives, we acknowledge that the term 'immigrant women' is generic and refers to a highly heterogeneous group of individuals with a complex and vast array of ethnocultural groups.

Aim and rationale

We consider in this paper how accessibility and acceptability manifest, as important dimensions of access to maternity care services and women's perception about availability of services and their experiences of accessing these services and whether interventions exist that challenge inequalities in maternity health care provision.

Our review utilised two theoretical frameworks first Gulliford and colleagues theory of access and secondly the concept of cultural safety.

A theory of access to services developed by Gulliford *et al.*[12] map out four dimensions *(Figure 1)*:

1. Service availability
2. Utilisation of services and barriers to access (which includes personal, financial, and organisational barriers)
3. Relevance, effectiveness, and access
4. Equity and access

INSERT FIGURE 1

For peer review only - http://bmjopen.bmj.com/site/about/guidelines.xhtml

Final

We used this theoretical model in our systematic review, which was based on a synthesis project funded by the National Institute for Health Research (NIHR). Unlike most access models in the United States, this framework reflects the philosophy of the NHS in that its key principles are to provide horizontal access (ensuring equality of access in the population) Could refer here to limitations on this re asylum seekers that note above and vertical access (meeting the needs of particular groups in the population, such as minority ethnic groups)recognised to have complex needs by NICE. The application of these principles is influenced by availability, accessibility, and acceptability. The Gulliford model has been widely used in empirical research, with the main paper having been cited at least 386 times. With its emphasis on accessibility, acceptability, relevance, and effectiveness, this model is entirely appropriate for assessing the provision of maternity services to minority ethnic groups. In this study we used the Theory of Access to assist in the theme development. Following theme development, we established how this theory intersected with our evidence.

Secondly, concepts of cultural safety which provided a theoretical lense for the production of recommendations. Cultural safety is a theory that aims to assist the underderstandings of deficits in care by considering the historical and social processes that impact power relationships within and beyond healthcare.[24] Cultural safety is achieved when programmes, instruments, procedures, methods, and actions are implemented in ways that do not harm any members of the culture or ethnocultural group who are the recipients of care. Those within the culture are best placed to know what is or is not safe for their culture, which suggests the need for increased dialogue about immigrant and partner approaches.[25-29] Our protocol is published in BMJ *Open* see https://bmjopen.bmj.com/content/bmjopen/7/7/e016988.full.pdf.

Methods

We used Popay's approach to NS[30], which consists of 4 elements (for a comprehensive explanation please see our published protocol (https://bmjopen.bmj.com/content/bmjopen/7/7/e016988.full.pdf). The unique feature of this approach is that it provides highly specified steps.

Element 1: Developing a theory of why and for whom.
Element 2: Developing a preliminary synthesis of the findings of the included studies, following implementation of the search strategy.
Element 3: Exploring relationships in the data.
Element 4: Assessing the robustness of the synthesis.

The NS approach relies primarily a narrative synthesis of the key findings of studies using text to summarise the findings of the synthesis, which is a result of a synthesis of the *narrative findings of included papers*. Team members have successfully employed NS previously and have vast expertise in its usage. [7, 32] NS maybe used with all paradigms of

For peer review only - http://bmjopen.bmj.com/site/about/guidelines.xhtml

Final

research quantitative, qualitative studies, and mixed methods research studies, as the emphasis is on an interpretive synthesis of the narrative findings of research rather than on a metadata analysis.[30]

Search strategy refinement and implementation

The search strategy used key terms used in consistently formulated text-based queries and search statements. These terms were based on subject headings, thesaurus terms, or related indexing and categorisation terms appropriate for each literature database. An example of a detailed final search strategy is given in *Supplementary File 1*. First, we searched 10 electronic databases using the aforementioned strategies see *Supplementary File 2.* Following this, we searched for appropriate grey literature in SI Web of Knowledge Conference Proceedings Citation Index (Science 1990–), ISI Web of Knowledge Conference Proceedings Citation Index (Social Science and Humanities 1990–), ProQuest Dissertations and Theses, and the Cochrane Methodology Register. We also searched using Google and Google Scholar and consulted with the study expert advisory group. In conclusion we hand searched the reference list of all included studies and relevant systematic reviews. Citations were downloaded into an ENDNOTE library and following this all duplicates removed. The bibliographic databases that we searched are listed in Table 1.

INSERT TABLE 1

We adopted the PICO approach to implement the search strategy as follows:-
P = immigrant women
I = maternity care
[C = non-immigrant women - implicit comparator emerging in the results]
O = experience of care

Therefore, our search strategy development was based on:

Search concept 1 = pregnancy, childbirth [implicitly females requiring maternity care], explicit terms covering women/females requiring all types of maternity care [antenatal, perinatal, postnatal, etc.]
Search concept 2 = immigrant populations [which would not fully distinguish between "new" and "second-generation" immigrants – this would be done at the selection stage]
Search concept 3 = terms used to identify access to, use of, deficiencies in, etc., service provision [to help identify groups with poorer health outcomes or vulnerabilities]
This comprehensive search strategy generated high rates of retrieval of records, however many were not pertinent.

Our final answer set of citations included concepts 1 and 2 and 3.

Screening for relevance
I

For peer review only - http://bmjopen.bmj.com/site/about/guidelines.xhtml

Final

-

In many cases, the study populations/sample was not fully described. When this dimension was opaque we contacted the authors for further clarification. We therefore in some cases used linguistic ability e.g. the need for an interpreter as a *proxy for immigrant status*. Our focus was on first generation immigrant women regardless of their phenotype meaning was associated with inclusion of women of *white ethnicities*, although we encountered few studies that focused on these groups. Study screening was undertaken by two team members independently (GH & BH) assessing the relevance of titles and abstracts in respect of our screening tool. The entire team reviewed ambiguous papers in order to achieve a consensus agreement and where necessary full text papers of potentially included studies were retrieved and appraised. The exclusion and inclusion criteria can be found in *Supplementary File 3*. When we retrieved full text papers which were later rejected we have documented these excluded papers and presented a rationale for exclusion. These can be found in *Supplementary File 4* and the included studies in *Supplementary File 5*.

Results

The search outcomes are comprehensively detailed in figure 2 the PRISMA flow chart [38]
INSERT FIGURE 2 PRISMA FLOW CHART

Studies included in the review, findings, and evidence

Our systematic review using narrative synthesis identified 40 empirical research studies in the scientific and grey literature. A broad range of ethnocultural groups and methodological genres are included in this review (see table 2 for of included studies). The distribution of the studies across the themes can be found in figure 3 and publication dates in figure 4.

INSERT TABLE 2

INSERT FIGURE 3 AND 4

Data Extraction and assessment of relevance

We conducted the following foundational activities in order to extract data (discussed in detail later).

1) Textual description. A systematic textual narrative was written for each study. We used headings adapted from Popay *et al.*[30] Setting, Participants, Aim, Sampling and Recruitment, Method, Analysis, Results.

(2) Tabulation and summarisation of all studies to be included. These tables described the attributes of the studies and the results. Information was extracted from the textual

For peer review only - http://bmjopen.bmj.com/site/about/guidelines.xhtml

Final

description using the same headings as above and additional headings as necessary. Papers in the PDF format were imported into ATLAS.ti qualitative data analysis software (ATLAS.ti Scientific Software Development GmbH, Berlin) using the 'Attributes' option to allow the tabulation of relevant data.

Quality assessment

In element w e conducted the quality appraisal (see tables 3 & 4). All included studies were critically appraised by two reviewers using tools from the Center for Evidence-Based Management (CEBMa).[33] We used GRAMM [34] for the mixed-methods studies. Differences were resolved in our reflective team meetings. We also used high, medium, and low as appraisal categories, discussed in detail below. This is approach is congruent with recent publications from the Cochrane Qualitative Research Group's CERQUAL publications, as they use this type of evaluation was previously used by Higginbottom et al. published studies.[7, 19] Studies are classified in three into domains, high, medium and low to enable a 'macro' evaluation.

- *High* was assigned to studies that used a rigorous and robust scientific approach that largely met all CEBMa benchmarks, perhaps equal to or exceeding 7 out of 10 for qualitative studies, 9 out of 12 for cross-sectional surveys, or 5 out of 6 for mixed-methods research.

- *Medium* was assigned to studies that had some flaws but that did not seriously undermine the quality and scientific value of the research conducted, perhaps scoring 5 or 6 out of 10 for qualitative studies, 6 to 8 out of 12 for cross-sectional surveys, or 4 out of 6 for mixed-methods research.

- *Low* was assigned to studies that had serious or fatal flaws and poor scientific value and scored below the numbers of benchmarks listed above for medium-level appraisals in each type of research

---

INSERT TABLES 3 & 4

The past decade as witness a growth approaches to assessing quality. Popay [30] suggests evaluating not only the scientific quality but also the 'richness' of studies. Popay defined richness as "*the extent to which study findings provide in-depth explanatory insights that are transferable to other settings*"p 230.[30] 'Thick' papers create or draw upon theory to provide in-depth explanatory insights that can potentially be transferable to other contexts. By contrast, 'thin' papers provide a limited or superficial description and offer little opportunity for generalising. Each paper was assessed against the criteria as set out in Higginbottom et al.

For peer review only - http://bmjopen.bmj.com/site/about/guidelines.xhtml

Final

p.5 and categorised as either ‘thick’ or ‘thin’ see table 3.What is outcome of all this quality assessment – decide to retain but quality indicator that perhaps at least refer to in Discussion?

Methodological genres

We identified eight quantitative studies that all used a questionnaire for data collection.[39-46] These population-based studies and cohort surveys were all cross-sectional: none were longitudinal.

Mixed-methods studies

We identified ten mixed-methods studies using a range of designs; that is, these studies employed both qualitative and quantitative dimensions.[1, 2, 47-54] For example, Duff *et al.*[48] reported a two-stage psychometric study in which focus groups and interviews were used in the first stage to develop a questionnaire for an ethnocultural group (Sylheti) In the second stage, quantitative methods were used to test and evaluate the acceptability, reliability, and validity of the questionnaire. Other mixed-methods designs included (a) interviewing a small sample of the participants after collecting data from a large-scale survey; (b) conducting semi-structured interviews with a small sample of participants based on quantitative data routinely collected from a large group of participants; and (c) using face-to-face, postal, and online questionnaires to collect data. One of the studies used Q methodology, which uses questionnaires with structured and unstructured questions.

Qualitative studies

Of the 40 studies included in this review , we identified 22 as qualitative research studies employing a range of qualitative methodologies and approaches.[4, 105-125] However, many of these studies did not specify a qualitative methodological genre but instead employed a more generic qualitative approach and described only the data collection tools used. For example, some presented multiple longitudinal case studies of participants (asylum seekers and refugees) about their maternity care experiences that included photographs taken by the participants, field notes, and observations in addition to researcher interviews. Another example was a case study of an ethnocultural group, immigrant women of Somali origin, [106] that used semi-structured interviews and focus groups. Some studies used focus groups and interviews conducted in the language of the population group; for example, Bengali, Sylheti, Urdu, and Arabic. Others used in-depth interviews, open-ended questions, group story-sharing sessions, and individual biographical life-narrative interviews. In contrast, a few studies specified a qualitative interpretive approach that used hermeneutic phenomenology and focused ethnography.

Studies focusing on specific ethnocultural groups

The chosen studies included participants from a wide range of ethnocultural groups that originated in diverse countries in different continents, including Asia (e.g., Bangladesh and

For peer review only - http://bmjopen.bmj.com/site/about/guidelines.xhtml

Final

Pakistan), Africa (e.g., Somalia and Ghana), and Europe (e.g., Poland). In some cases, the sample was drawn from a single ethnocultural group, such as Bangladeshi.[48] However, most of the studies were undertaken on mixed samples of immigrant women originating from different countries (e.g., Somalia, Bangladesh, and Eastern Europe).

Studies focusing on immigrant women without a clearly specified ethnocultural group
We identified 16 studies that used the term immigrant women generically and not clearly specify an ethnocultural group. In deciding to include these studies, we believed that legitimate proxies for immigrant status could be the specified use of an interpreter or the participants having countries of origin or birth outside the UK. Some studies reported immigrant women arriving from 14 different countries but did not specify the country of birth. These studies could still be included.

Studies sorted by immigrant category of the participants
More than half of the included studies (25 in total) did not clearly specify the immigrant category of the population they studied, such as economic migrant, asylum seeker or refugee.[17, 39-42, 44-50, 52-64]

Other studies specified the immigrant category as asylum seekers only;[51, 65-68] refugees only;[17, 69-71] or a mix of immigrant categories that included spousal immigrants, economic migrants, asylum seekers, and refugees.[1, 2, 43, 72-74] Although a range of identifiers were used, we noted a pattern of reducing immigrant women and their complex cultures, ethnicities, and lives to simplified contexts or situations (e.g., 'asylum seekers') and using 'immigrant' as a generic label for a non-British and potentially non-white woman.

Studies sorted by phase of maternity care

Of the included studies, 17 were undertaken across all phases of maternity care: antenatal, intrapartum, and postnatal.[1, 2, 46-48, 50, 54, 59-63, 65, 67, 69, 72, 75] Nine studies reported on antenatal care alone,[39, 43, 52, 56, 57, 66, 71, 73, 74] and six studies are focused on postnatal care.[44, 49, 51, 58, 64, 70] Two studies included focused on ante- and postnatal care but did not include the intrapartum phase.[42, 53] One study focused on just the intrapartum and postnatal phases of maternity care,[55] and one study did not specify the maternity care setting.

The characteristics of participants in all the included studies can be found in *Supplementary File 6.*

Analysis and synthesis

Following construction of the preliminary themes, we produced code/narrative theme tables to demonstrate how the basic meaning units related to the theme. Utilising the codes produced in ATLAS.ti and aligning these to the manually extracted key findings see Figure 5 We reviewed all these processes in our reflective team meetings to ensure the rigour and robustness of our analytical steps. This iterative process similar to qualitative research

For peer review only - http://bmjopen.bmj.com/site/about/guidelines.xhtml

Final

involved deconstructing the narrative findings into meaning units and social processes as they manifested in maternity care experiences of immigrant women. Individual team members engaged in independent theming of tabular and coded data. We subsequently merged these individual perspectives to form the final harmonised themes representing a '*meta-inference'* with respect to the narrative findings of the included studies. Meta-inference is a term used in mixed methods research to describe merging of findings from the positivistic and the interpretative paradigms as is the case in this NS. Tashakorri and Teddlie p.101 [36] describe meta-inference as "*an overall conclusion, explanation of understanding developed from the integration of inferences obtained from the qualitative and quantitative strands".*

We have constructed the themes in a directive fashion (*meaning containing implicit indications)* in order to provide tangible guidance for policy and practice that might be developed into transformational policy and practice relevant strategies that benefits immigrant women and the NHS.

Following construction of the preliminary themes, we produced code/narrative theme tables to demonstrate how the basic meaning units related to the theme. Utilising the codes produced in ATLAS.ti and aligning these to the manually extracted key findings **see figure 5**

INSERT FIGURE 5

Rigour, reflexivity and the quality of the synthesis

Reflexivity in the review process requires a self-conscious and explicit acknowledgement of the impact of the researcher on the research processes, interpretations and research products. Reflexivity demands acknowledgement of inherent power dimensions, hierarchies and prevailing ideologies that might shape and determine interpretations and the consequent knowledge production and research products. Gender, sexuality, professional socialisation, ethnocultural orientation and political lenses as these impact upon social identities further coalescing to provide a specific perspective on any given phenomena. The review team members are imbued with a strong personal and professional commitment to the eradication of inequalities and allegiance to contemporary equality and diversity agendas. From a reflexive perspective, this is important given that immigration is global phenomena and the inherent vulnerability of some immigrant women.
Reflexive analysis alerts us as researchers to emergent themes and informs the formal and systematic process of analysis. Murphy *et al.* p.188[37] define reflexivity as:

> *" we mean sensitivity to the ways in which the researcher's presence in the research setting has contributed to the data collected and their own a priori assumptions have shaped the data analysis"*

Our collaborative decisions required constant review and reading and, in some cases, reviewing the theme allocation and evidence. Therefore, we believe we achieved a nuanced and comprehensive approach, reaching consensus.

For peer review only - http://bmjopen.bmj.com/site/about/guidelines.xhtml

Final

Within the published NS reviews, we have not noted great attention to the issue of publication bias however we strived to eradicate any potential bias by undertaking a comprehensive and exhaustive literature review that included grey literature and follow up emails with authors seeking greater clarity and explanation of opaque issues. A number of the included research studies were identified via *ProQuest* and *E-theses* and do not appear as publications in peer reviewed scientific journals.

We held a national stakeholder event during which we presented our preliminary findings to a wide range of health professions (obstetrician, general practitioner and midwives), academics, voluntary and community workers. Possibly this approach may be considered contentious in the respect of systematic review, as attendees had no previous knowledge of the original included papers, however they held deep topic knowledge. Notwithstanding this, we found broad support for our findings and facilitated groups work activities in order to challenge our initial interpretations. These challenges resulted in the construction of *Theme 5: Discrimination, racism, stereotyping, cultural sensitivity, inaction, and cultural clash in maternity care for immigrant women.* These focused activities collectively contribute to the confidence in the review findings, providing verification and validation of the themes.

We identified 40 research studies that met our inclusion criteria, and we extracted and synthesised key findings into five themes. see Table 5 for the publications informing each theme.

INSERT TABLE 5

Theme 1: Access and utilisation of maternity care services by immigrant women

Late booking emerged as an important dimension in this theme with immigrant women study participants often booking and accessing antenatal care later than the recommended timeframe (during the first 10 weeks of pregnancy). This issue was found to be multi-factorial in nature, including the effects of limited English language proficiency, immigration status, lack of awareness of the services, lack of understanding of the purpose of the services, income barriers, the presence of female genital mutilation, differences between the maternity care systems of their countries of origin and the UK, arrival in the UK late in the pregnancy, frequent relocations after arrival, the poor reputations of antenatal services in specific communities and perceptions of regarding antenatal care as a facet of medicalisation of childbirth. The factors affecting the access and utilisation of postnatal services were similar to those reported for antenatal services.

Theme 2: Maternity care relationships between immigrant women and healthcare professionals.

For peer review only - http://bmjopen.bmj.com/site/about/guidelines.xhtml

Final

Our included studies evidenced the perception of service users in this group and their interactions and therapeutic encounters with healthcare professionals. These interactions were significant in understanding access, utilisation, outcomes, and the quality of their maternity care experience.

Included studies in this theme demonstrated that the perceptions of study participants regarding the ways healthcare professionals delivered maternity care services were both positive and negative. A number of studies illustrated positive relationships between healthcare professionals and immigrant women. Study participants asserted that the healthcare professionals were caring, respected confidentiality, and communicated openly in meeting their medical as well as emotional, psychological, and social needs. Conversely, some studies evidenced negative relationships between participants and healthcare professionals. Studies evidenced healthcare professionals from the perspective of immigrant women as being rude, discriminatory, or insensitive to the cultural and social needs of the women. The end result of these negative encounters being that these women tended to avoid accessing utilising maternity care services consistently.

Theme 3: Communication challenges experienced by immigrant women in maternity care.

It is axiomatic that limited English language fluency presents verbal communication challenges between health care professional and their patients, families and carers. Moreover, this is compounded when healthcare professionals use complex medical or professional language that is difficult to comprehend. Nonverbal communication is culturally defined and challenges can occur through misunderstandings of facial expressions, gestures, or pictorial representations. Poor communications result as illustrated in our included studies in limited awareness of available services in addition to miscommunication with healthcare professionals. Study participants often expressed challenges accessing services, failed to understand procedures and their outcomes, were constrained in their ability to articulate their health or maternity needs to health care providers, were disempowered in respect of their involvement indecision making, often/sometimes gave consent for clinical procedures without fully comprehending the risks and benefits, and did not receive understandable advice on baby care. Studies identified that communication was not reciprocal and healthcare professionals often misunderstood participants. These created feelings of isolation, fear and a perception of being ignored. Interventions to address the language challenges included the provision of formal and informal services, bilingual support workers, and written maternity care information in the necessary languages.

Theme 4: Organisation and legal entitlements and their impacts on the maternity care experiences of immigrant women.

The study participants in our included studies had mixed experiences with the maternity care services in the UK. Positive and commendable experiences included feeling safe in giving birth at hospital rather than at home, being able to register a complaint if poor healthcare was received, being close to a hospital facility, not being denied access to a maternity service, and

For peer review only - http://bmjopen.bmj.com/site/about/guidelines.xhtml

Final

having good experiences with postnatal care. Conversely, negative experiences included lack of continuity e.g. not being able to see same maternity care providers each time and being unaware of the configuration of maternity services work, limiting navigation. Participants in our included studies found services bureaucratic and perceived within the UK maternity care model a propensity towards medical/obstetric intervention and lower segment caesarean section births.

The legal status of an immigrant women in the UK has a profound influence on their on their access to maternity care. Women without entitlement to free maternity care services in the UK were deterred from accessing timely antenatal care by the costs and by the confidentiality of their legal status. Moreover, some women arrived in the UK during the final phase of their pregnancies that resulted in fractures in the care process, loss of their social networks, reduced control over their lives, increased mental stress, and increased vulnerability to domestic violence.

Positive experiences included receiving information from their midwives on the benefits of breastfeeding together with demonstrations on how to position the baby. Negative experiences included poor support from hospital staff on how to breastfeed their babies consequently these reported experiences are mixed.

Theme 5: Cultural sensitivity, inaction, and cultural clash in maternity care for immigrant women.

Inequalities in access, navigation, utilisation and the subsequent maternity care outcomes are influenced by discrimination and cultural insensitivity in maternity care services according to the perspectives of women in several included studies. Although discrimination iss often subtle and difficult to identify, direct and overt discrimination was reported in some studies.

Specifically, study participants of Muslim faiths challenged assumptions held by healthcare professionals, including those held regarding Muslim food practices and that their partners or husbands should help the women during labour. Moreover, healthcare professionals were reported in some studies to lack cultural sensitivity and cultural understanding. For example, these women did not optimally benefit from antenatal classes facilitated by a non-Muslim educator who had no knowledge of the relationships of Muslim culture to maternity. Furthermore, studies reported participant dissatisfaction of antenatal class with a gender mix, which contravened religious edicts. Studies illustrated that some women of Muslim faith felt their cultural and religious needs for breastfeeding were not met on the postnatal wards, and they felt that the staff lacked insight, knowledge and understanding of female genital mutilation (FGM).

Evidence from our included studies suggests some immigrant women perceived that the staff did not treat them with respect or attended fully to their health care needs, and they felt devalued, unsupported, and fearful while receiving maternity care. In a few cases, however,

For peer review only - http://bmjopen.bmj.com/site/about/guidelines.xhtml

Final

midwives were happy to meet the cultural and religious needs of the study participants in our included studies in both antenatal and postnatal settings and this is a, positive finding.

Our findings also identified instances of cultural clash and conflicting advice during pregnancy and maternity care, mostly resulting from differences between the cultural practices and medical systems of the home countries of the immigrant women and those in the UK We conceptualise the findings graphically in figure 6.

INSERT FIGURE 6

Discussion and conclusions

The UK is in a period of superdiversity defined as a "distinguished by a dynamic interplay of variables among an increased number of new, small and scattered, multiple-origin, transnationally connected, socio-economically differentiated and legally stratified immigrants"' Responding to this level of diversity is challenging for UK maternity care health services and may require the development of new and innovative strategies.

The experiences of immigrant women in accessing, navigating and utilising maternity care services in the UK are both positive and negative. In order to enhance services it is essential that strategies are developed to overcome the negative experiences reported. The experience of maternity care services is multi-factorial in nature with a number of issues appearing to coalese to determine the poorer experience reported by some immigrant women .Important factors identified by the review included a lack of language support, cultural insensitivity, discrimination, poor relationships between immigrant women and healthcare professionals, and a lack of legal entitlements and guidelines on the provision of welfare support and maternity care to immigrants.

Implications of findings and recommendations for maternity care policy, practice, and service delivery

Inequitable access appeared to be a consequence of the immigration and legal status of asylum-seeking women, and has a profound impact on health care experiences and consequently health. language fluency, as this appears to be key determinant of optimal access and utilisation of maternity care services. We concluded that addressing language barriers and ensuring culturally sensitive care are essential elements of providing optimal maternal care for immigrant women. The issue of confidentiality may be compromised by having known interpreters in small communities: lack of confidentiality can make finding ways to communicate with women with low levels of English more problematic. Setting up a national-level website offering standard information on maternity care with the option of translation in a wide range of languages may be a solution. Additionally, the identification of best language practices should be identified with regard to improve the current language service model.

For peer review only - http://bmjopen.bmj.com/site/about/guidelines.xhtml

Final

The knowledge, understandings and attitudes of maternity care health care providers is a critical determinant of care. Ethno-culturally based stereotypes, racism, judgmental views, and direct and indirect discrimination require eradication which will require challenging discrimination and racism at all levels: individual, institutional, clinical, and societal Interventions to improve maternity care for immigrant women are scant, and formal evaluations of these interventions were largely absent. In addition, the interventions needed to be more focused and implemented at the organisational, service, and staff levels. Increasing the social capital, health literacy, and advocacy resources for immigrant women may empower women to access and utilise maternity care services appropriately.

Maternity care staff require a greater level of mandated education to have better cultural awareness of needs of diverse client groups including newcomers to the UK. Our findings highlight the importance of demonstrating compassion, empathy, and warmth in their relationships with these women to reinforce positive attitudes among the immigrant women.

It is contingent on maternity care providers to value diversity among service users and to offer individualised and culturally congruent care. One way to achieve this goal would be through birth plans that can be jointly agreed and discussed in advance by the maternity care staff and recently arrived newcomers and immigrant women. Maternity care staff should seek to empower immigrant women by providing comprehensible information and better education concerning the configuration of the maternity system in the UK, conveying accurate information about care delivery. Central to these suggestions may be to enable volunteer and third-sector organisations to work as links between the statutory maternity services and immigrant women. We found evidence (though not scientifically evaluated) of such links in our national networking event.

Representatives of immigration control agencies may feel obligated to adheres to immigrant rules and consider the maternity care needs of immigrant women's and baby's health as a secondary issue. The policy context regarding data protection and sharing information with the Home Office about the immigrant status of women was at issue as well, especially since variabilities have been seen in the policies for sharing this information. The results suggest that the legal and policy context is important in addressing the maternity care needs of immigrant women.

It would seem imperative, as reflected in current policy directives, to adopt a universal of aim of achieving optimal maternity care for all and not just for immigrant women .However, maternity care services should strive to give more information to immigrant women about their rights to care, the availability and configuration of maternity services, and how to navigate maternity care systems. The child in utero of an immigrant is a future UK citizen and optimising maternity care is a dimension of securing the future health of the nation. In a period of super diversity is incumbent upon health professional to have an awareness of immigrant women's legal rights and perhaps education on this topic should be mandated for maternity care professionals. Continuity in maternity caregivers and compulsory provision of

For peer review only - http://bmjopen.bmj.com/site/about/guidelines.xhtml

Final

interpreters would also help to improve the experiences of these women. This is a general para and better to start with more specific issue relating to

Decision makers and health care leaders should address the findings at a strategic level. A focus on diversity, equality and the needs of immigrant women could reasonably be embedded in the role and responsibility of '*Board level Maternity Champion'* and of '*Maternity Clinical Networks*'. Maternity service providers could consider the appointment of one obstetrician and one midwife jointly responsible for championing maternity care provision to immigrant women in their organisation. As these dimensions feature within the '*Bespoke Maternity Safety Improvement Plan.*
Key areas of action include: -

- **Focus on learning and best practice** – issues of equality and diversity should be featured in the Saving Babies' Lives care bundle for use by maternity commissioners and providers.

- **Focus on multi-professional team working** – continuous personal and professional training

- **Focus on data** – greater focus on ethnicity and immigration within the Maternity Services Dataset and other key data sets.

- **Focus on innovation** – create space for accelerated improvement and innovation at local level.

Gaps in the evidence

We identified very few interventions to address inequalities in access and quality in maternity care for immigrant women in the published literature , and the effectiveness of these few had not been evaluated robustly while none included economic evaluation of the intervention. Studies of the usual 6 weeks postnatal checks by a general practitioner were not identified nor studies that focused on the intrapartum period. As mentioned earlier we found few studies that focused on immigrant women with 'white ethnicities' in our review time period e.g. women of Eastern European origin.

Strengths and limitations

- We were challenged and constrained by the lack of consistency in describing immigrant population sin the published literature. There is exists a great deal of variation and no unified approach within the UK literature.

For peer review only - http://bmjopen.bmj.com/site/about/guidelines.xhtml

Final

- Immigration is an international phenomenon and this review increases understanding of how immigrant women navigate maternity services in the UK

- The review systematically maps our positive and negative aspects of maternity care provision as experienced by immigrant

- The review provides strategic policy level direction for enhancement of maternity care services

- The review does not address the experiences of maternity care for second generation women (e.g. women of black and minority origin born in the UK) nor does it consider refugee and asylum seeking women as a separate group

Implications for future research

More research is required into the term 'immigrant', how this term is used, and the changes in its use over time that may affect immigrant women's care. At present, the term is used very broadly and simplistically, which masks its inherent heterogeneity. Furthermore, more research is required to understand how the intersections of particular characteristics – such as gender, education status, time in the UK, immigration status, wealth, and country of origin – may influence or alter the experiences of these women in their maternity. Research is required that focuses on developing and evaluating specific interventions to improve maternity care for immigrant women.

COMPETING INTEREST
None

AUTHOR CONTRIBUTIONS

Dr Gina Higginbottom (Professor, School of |Health Sciences) was principal investigator. Initiated the project and oversaw all stages. Led the interpretation/synthesis phases and drafted the manuscript.

Dr. Basharat Hussain (Senior Research Fellow) contributed to all stages of the review. Led the data extraction, coding, and quality appraisal and contributed to the manuscript.

Dr. Catrin Evans (Associate Professor of Nursing, Director of the Centre for Evidence Based Health Care) contributed to all stages of the review, provided expert methodological advice, acted as second reviewer for quality appraisal, and development of the synthesis. She contributed to the review of the final version of the manuscript.

For peer review only - http://bmjopen.bmj.com/site/about/guidelines.xhtml

Final

Dr Myfanwy Morgan (Professor Emerita King's College London) contributed to all stages of the review, provided expert methodological advice, acted as second reviewer for quality appraisal, and development of the synthesis. She contributed to the review of the manuscript.

Dr Kuldip Bharj (Retired Director of Midwifery, University of Leeds) contributed to all stages of the review, provided clinical and policy perspectives, contributed to formulation of the implications and recommendation in the manuscript.

Jeanette Eldridge (Information Specialist) designed the literature search strategy, advised the team on all aspects of information retrieval, undertook the main database searches, and contributed to the development of the manuscript.

Disclaimer

The views expressed in this report are those of the authors and not necessarily those of the NHS, NIHR or the Department of Health.

Patient and Public Involvement

The systematic review questions were developed in consultation with our project advisory group (PAG) including service users' priorities experience and preferences. This systematic review did not include empirical research therefore there were no human participants.

Acknowledgements

This project was funded by the UK NIHR Health Services and Delivery Research Programme (Grant No. HS&DR-15/55/03). Along with this funding, NIHR also contributed by peer reviewing the funding proposal.
We gratefully acknowledge valuable input from the members of our Project Advisory Group. Their input has been very helpful in making possible the successful completion and the high quality of this review. The following people kindly consented to be members:

- Jim Thornton, Professor of Obstetrics and Gynaecology, Faculty of Medicine and Health Sciences, University of Nottingham
- Dr Caroline Mitchell, General Practitioner/Senior Clinical Lecturer, Clinical Academic Training Programme Lead, Academic Unit of Primary Medical Care (AUPMC), University of Sheffield
- Dr Jane Mischenko, Commissioning Lead: Children and Maternity Services, NHS Leeds
- Carol McCormack, Specialist Midwife, NUH Trust

We also thank following immigrant women for their input in the conceptualisation of this review:

For peer review only

Final

- Ms Valentine Nkoyo, Director of Mojatu, Nottingham
- Kinsi Clarke, Nottingham Refugee Forum

Data Availability

Yes, the data are available as all studies in this review are publicly available.

For peer review only

IN TEXT TABLES

For peer review only - http://bmjopen.bmj.com/site/about/guidelines.xhtml

Final

Ovid MEDLINE 1948– and MEDLINE In-Process and Other Non-Indexed Citations to daily update

- Ovid EMBASE 1980–2017 Week 11
- Ovid PsycINFO 1972–March Week 3 2017
- CINAHL Plus with Full Text/EBSCOHost to 2017
- MIDIRS on Ovid 1971 to April 2017
- Thomson Reuters Web of Science* 1900–2017
- ASSIA on ProQuest 1987–current
- HMIC on Ovid 1979–January 2017
- POPline (via http:// www.popline.org/) 1970 to the present

Thomson Reuters Web of Science 1900-2017 includes the following:

- Science Citation Index Expanded (SCI-EXPANDED) 1900–2017
- Social Sciences Citation Index (SSCI) 1956–2017
- Conference Proceedings Citation Index - Science (CPCI-S) 1990–2017
- Conference Proceedings Citation Index - Social Science and Humanities (CPCI-SSH) 1990–2017
- Book Citation Index - Science (BKCI-S) 2008–2017
- Book Citation Index - Social Science and Humanities (BKCI-SSH) 2008–2017
- Emerging Sources Citation Index (ESCI) - 2015–2017

Table 1: Data bases searched

Table 2: Master table of included studies

For peer review only - http://bmjopen.bmj.com/site/about/guidelines.xhtml

Final

Reference	Study aim	Region	Methodology	Theory or Framework	Setting	Data analysis	Sample and mode of recruitment
125	To establish efficacy of link-worker services (an intervention) introduced for non-English-speaking Asian women in multi-racial health districts	Not specified	Quantitative survey: 21-item questionnaire	Not specified.		Qualitative: content analysis	Questionnaire to the Heads of Midwifery Services in 30 multi-racial district health authorities. 20 responded. Sample is not immigrant women, however this is an evaluation of an intervention
131	To develop a reliable and valid questionnaire to evaluate satisfaction with maternity care in Sylheti-speaking Bangladeshi women.	London.	Mixed methods: two-stage psychometric study. Firstly, a Sylheti-language questionnaire regarding Bangladeshi women's experiences of maternity services was translated and culturally adapted from an English-language questionnaire using focus groups, in-depth interviews, and iterative methods. Secondly, quantitative psychometric methods were used to field test and evaluate the acceptability, reliability, and validity of this questionnaire.	Not specified.	Not specified.	Qualitative: thematic analysis. Quantitative: validity of an instrument.	Located at four hospitals providing maternity services in London, UK. Study participants included 242 women from the London Bangladeshi communities who were in the antenatal (at least 4 months pregnant) or postnatal phase (up to 6 months after delivery). The women spoke Sylheti, a language with no accepted written form. In stage one purposive samples of 40 women in the antenatal or postnatal phase participated, along with one convenience sample of six women in the antenatal phase and three consecutive samples of 60 women in the postnatal phase. In stage two, 135 women (main sample) completed the questionnaire 2 months after delivery (82% response rate), and 50 women (retest sample) from the main sample completed a second questionnaire 2 weeks later (96% response rate).

For peer review only

Final

105	To study the maternity care experiences of Somali refugee women in an area of West London. This article focused particularly on findings relating to the language barrier, which to a large degree underpinned or at least aggravated other problems the women experienced.	West London.	Qualitative: case study. Six semi-structured interviews and two focus groups (with six participants each).	Not specified.	Not specified.	Qualitative: themati analysis.	Snowball sampling: 12 Somali women were selected from a larger survey involving 1400 women.
106	To undertake a qualitative study of the maternity experiences of 33 asylum seekers.	London, Plymouth, Hastings, Brighton, Oxford, Manchester, and King's Lynn.	Qualitative.	Not specified.	Home or a neutral location.	Qualitative: content analysis.	Convenience and snowball sampling of recent asylum seekers. Based on semi-structured interviews carried out in seven English cities.

For peer review only - http://bmjopen.bmj.com/site/about/guidelines.xhtml

Final

132	A Sure Start local programme had funded a Bangladeshi support worker to provide bilingual breastfeeding support to childbearing Bangladeshi women, many of whom were not fluent in English. This study aimed to conduct a short evaluation of the impact of this work on the uptake and duration of breastfeeding among these women.	Tower Hamlets.	Mixed methods: the survey questionnaire included some open and closed questions about the women's intention to feed; their current feeding methods; the breastfeeding support and information they received antenatally, during the hospital stay, and postnatally; overall views on the information and support received; and some demographic details. Eleven interviews were conducted by telephone in Sylheti (a dialect that has no written format), three in English and one in Urdu (using a female family member to translate). Interviews took between 15 and 30 minutes to complete.	Not specified.	Not specified (survey conducted by telephone).	Qualitative: content analysis of a questionnaire (open and closed questions).	The two midwives and the support worker had provided breastfeeding support to 194 women during a one-year period (September 2001 to August 2002). Of these, 80 women received help from the support worker alone. The majority of these 80 women were Bangladeshi. For the evaluation, 15 women were randomly selected from these 80 women.

Final

48	To identify any social or ethnic differences in access to antenatal care and to quantify the effects of any such differences using data collected in a survey of women's experiences of antenatal screening.	England.	Quantitative: a cross-sectional survey using a postal questionnaire.	Not specified.	Not specified.	Quantitative: cross-sectional analysis.	A stratified clustered random sampling strategy was used. Hospitals in England were stratified according to ethnic mix. To ensure inclusion of an adequate number of women from black and minority ethnicity (BME) backgrounds, hospitals with ≥ 15% of women of BME origin were oversampled. Pregnant women aged ≥16 years and receiving care in 15 participating hospitals were sent a postal questionnaire at 27–31 weeks of gestation.
126	To compare the health behaviours both antenatally (smoking and alcohol consumption) and postnatally (initiation and duration of breast feeding) of mothers who have white British or Irish heritage with those of mothers from ethnic minority groups and to examine in mothers from ethnic minority groups whether indicators of acculturation (generational status, language spoken at home, and length of residency in the UK) were associated with these health behaviours.	England.	Quantitative: a prospective nationally representative cohort study.	Not specified.	Not specified.	Quantitative: cohort study.	Stratified clustered sampling framework to over-represent mothers from ethnic minority groups and disadvantaged areas produced 6478 white British or Irish mothers and 2110 mothers from ethnic minority groups. Of those from ethnic minority groups, 681 (33%) were first generation and 55 (4%) second generation.

For peer review only - http://bmjopen.bmj.com/site/about/guidelines.xhtml

Final

[107]	To explore and synthesise the maternity care experiences of female asylum seekers and refugees.	UK.	Qualitative: multiple exploratory longitudinal case studies that used a series of interviews, photographs taken by the women, field notes, and observational methods to contextualise data obtained during 2002 and 2003.	Theory of interactions and transformational educational theory.	Hospital settings or women's homes.	Qualitative: thematic analysis.	Women were approached if the status of 'asylum seeker' or 'refugee' was written in the hospital notes taken at their booking appointment. Fourteen women were approached, but nine women declined to participate. Five women consented, but one woman was dispersed before 20 weeks gestation and therefore was not included in the study. Of the remaining four participating women, three were asylum seekers and one was a refugee. The sampling technique was not clearly reported.
[108]	To identify key features of communication across antenatal care and whether they are evaluated positively or negatively by service users.	Central London.	Qualitative: used six focus groups of 15 participants each and conducted 15 semi-structured interviews. Non-English-speaking focus groups and interviews were conducted in standard Bengali, Sylheti, or Somali.	Not specified.	Focus groups: hospitals and university meeting rooms. Semi-structured interviews: various locations to suit the needs of the women.	Qualitative: thematic analysis.	The sampling technique was not clearly reported, but they recruited 30 pregnant women from diverse social and ethnic backgrounds affiliated with one NHS Trust (i.e., hospital) in central London. Participants were recruited within this hospital, in eight community antenatal clinics situated in socially and ethnically diverse areas, via a community parenting group for Somali women, and via a Bengali Women's Health Project. Within the hospital, participants were recruited from the antenatal waiting room (which services low- and high-risk pregnancies), the ultrasound clinic, and the glucose tolerance testing clinic.

For peer review only - http://bmjopen.bmj.com/site/about/guidelines.xhtml

Final

127	To determine the pregnancy outcomes of women of similar parity and ethnic background who received antenatal care ('booked') compared those who did not ('unbooked') over a period of 18 months.	North Middlesex University Hospital (NMUH), London.	Quantitative: a retrospective cohort study from September 2006 to March 2008 comparing the socio-demographics and the foetal and maternal outcomes of pregnancies of unbooked versus booked women.	Not specified.	Not specified.	Quantitative: a retrospective cohort study.	Women who received no antenatal care or who delivered within 3 days of their initial booking visit were categorised as 'unbooked'. In each case, the woman who had delivered next on the labour ward register (matched for ethnicity and parity) and who had received antenatal care prior to the second trimester served as a comparison.
133	To explore the perspectives of first- and second-generation women of Pakistani origin on maternity care and to make recommendations for culturally appropriate support and care from maternity services.	West Midlands.	Mixed methods: a retrospective Q methodology study of Pakistani women following childbirth.	Retrospective Q method study.	Not specified.	Qualitative: Q methodology.	A purposive sampling strategy was used. Postnatal first- and second-generation Pakistani women were self-identified by their responses to information leaflets disseminated at local Children's Centres across an inner city in the West Midlands.
109	To study the relationships between Somali women and their Western obstetric care providers. The attitudes, perceptions, beliefs, and experiences of both groups were explored in relation to caesarean sections, particularly to identify factors that might lead to adverse obstetric outcomes.	Greater London.	Qualitative: in-depth individual and focus group interviews.	Framework of naturalistic enquiry, emic/etic model	Not specified.	Qualitative: emic/etic analysis.	Selected 39 Somali women by snowball sampling, 36 from the community and three purposively from a hospital.

For peer review only - http://bmjopen.bmj.com/site/about/guidelines.xhtml

Final

110	To address the postulates that immigrant women experience sensitive care through the use of an ethnically congruent interpreter and that such women prefer to meet health providers of the same ethnic and gender profile when in a multi-ethnic obstetrics care setting.	Greater London.	Qualitative: in-depth individual and focus group interviews. Open-ended questions were presented by an obstetrician and an anthropologist.	Framework of naturalistic enquiry.	Not specified.	Qualitative: naturalistic inquiry.	Participants were recruited throughout Greater London between 2005 and 2006. Snowball sampling was used to recruit 36 immigrant Somali women, and another three were selected by a by purposive technique for a total of 39. A purposive technique was used to select further 11 Ghanaian women who had delivered at least one child within the British healthcare system and who were living within the study area at the time of data collection.

For peer review only - http://bmjopen.bmj.com/site/about/guidelines.xhtml

Final

134	To evaluate a pilot mental health service for asylum-seeking mothers and babies.	UK (not clear).	Mixed methods: evaluation within a participatory action research framework.	Participatory action research framework.	Not specified.	Qualitative: thematic analysis. Quantitative: the CARE-Index.	An active outreach recruitment strategy was adopted by psychologists, who embedded themselves in a drop-in community group, the Merseyside Refugee & Asylum Seekers & Asylum Seekers Pre-& Postnatal Support Group. Participants were West African women who were asylum seekers or refugee and who were either pregnant or had a young baby. They originated from The Gambia, Sierra Leone, Ivory Coast, and Nigeria. All spoke English. Their ages ranged from 17 to 32 years, and all babies were under 6 months of age at the point of initial contact, with three babies not yet born. Attendance at the 21 therapeutic group sessions ranged between 4 and 12 mothers (with their babies). Seven mothers attended a significant proportion or all group sessions. An additional six mothers attended 1-4 group sessions.
4	To apply the 'three delays' framework (developed for low-income African contexts) to a high-income Western scenario to identify delay-causing influences in the pathway to optimal facility treatment.	Greater London.	Qualitative: individual and focus group interviews.	'Three delays' framework.	Not specified.	Constructivist hermeneutic naturalistic study.	Purposive and snowball sampling was used to recruit 54 immigrant women originally from sub-Saharan regions in Africa (Somalia, Ghana, Nigeria, Senegal, and Eritrea) living in London and to recruit 32 maternal providers.

For peer review only - http://bmjopen.bmj.com/site/about/guidelines.xhtml

Final

14	To identify predictors of late initiation of antenatal care within an ethnically diverse cohort.	Newham, East London.	Quantitative: a cross-sectional analysis of routinely collected electronic patient records from Newham University Hospital NHS Trust (NUHT).	Not specified.	Not specified.	Quantitative: cross-sectional analysis.	All women who attended their antenatal booking appointment within NUHT between 1st January 2008 and 24th January 2011 were included in this study. The main outcome measure was late antenatal booking, defined as attendance at the antenatal booking appointment after 12 weeks (+6 days) gestation. The sample included women from Somalia, Eastern Europe, Africa, the Caribbean, and South Asia.
104	To explore BME women's experiences of contemporary maternity care in England.	All over England.	Qualitative data collected from a large cross-sectional survey using three open-ended questions that encouraged participants to articulate their experience of maternity care in their own words.	Not specified.	Not specified.	Qualitative: Thematic analysis.	A random sample of 4800 women was selected using Office for National Statistics (ONS) birth registration records. The overall response rate was 63% but was only 3% from BME groups. A total of 368 women self-identified as coming from BME groups. Of those, 219 (60%) responded with open text and 132 (60%) were born outside the UK.
111	To investigate women's experiences of dispersal in pregnancy and to explore the effects of dispersal on the health and maternity care of women asylum seekers who were dispersed during pregnancy in the light of NICE guidelines on antenatal, intrapartum, and postnatal care.	London, South of England, Midlands and East of England, North West, North East, and Wales.	Qualitative: interviews were conducted with 19 women who had been dispersed during their pregnancies and with one woman kept in an Initial Accommodation Centre under a new Home Office pregnancy and dispersal guidance issued in 2012.	Not specified.	Not specified.	Qualitative (not clear).	The sampling technique was not mentioned clearly. The women interviewed came from 14 different countries and had been dispersed or relocated to or within six regions of the UK. At the time of dispersal, 14 had been awaiting a decision on their asylum claim and six had been refused asylum.

For peer review only - http://bmjopen.bmj.com/site/about/guidelines.xhtml

Final

128	To compare the maternal and birth outcomes of Polish and Scottish women having babies in Scotland and to describe any differences in clinical profiles and service use associated with migration from Poland.	All over Scotland.	Quantitative: a population-based epidemiological study of linked maternal country of birth, maternity, and birth outcomes. Scottish maternity and neonatal records linked to birth registrations were analysed for differences in modes of delivery and pregnancy outcomes between Polish migrants and Scots. These outcomes were also compared with Polish Health Fund and survey data.	Not specified.	Not specified.	Quantitative: statistical analysis.	The study analysed 119,698 Scottish and 3105 Polish births to primiparous women in Scotland in 2004-09 using routinely collected administrative data on maternal country of birth and birth outcome.
112	To understand the multiple influences on behaviour and hence the risks to metabolic health of South Asian mothers and their unborn children, to theorise how these influences interact and build over time, and to inform the design of culturally congruent, multi-level interventions.	London boroughs, Tower Hamlets, and Newham.	Qualitative: group story-sharing sessions and individual biographical life-narrative interviews.	Multi-level ecological models.	All but four interviews were in the participants' homes.	Qualitative: phenomenology.	The study recruited from diabetes and antenatal services in two deprived London boroughs 45 women of Bangladeshi, Indian, Sri Lankan, or Pakistani origin aged 21–45 years with histories of diabetes in pregnancy. Overall, 17 women shared their experiences of diabetes, pregnancy, and health services in group discussions, and 28 women gave individual narrative interviews (facilitated by multilingual researchers). All were audiotaped, translated, and transcribed.

view only

Final

[20]	To understand the nature of need in super-diverse areas and to examine the emergent challenges for effective maternity service delivery in an era of superdiversity.	West Midlands.	Mixed methods: the study used a semi-structured questionnaire and held narrative interviews of newcomer women. The findings were then triangulated with interviews of professionals who regularly worked with such women.	Not specified.	Not specified.	Qualitative: systematic thematic analysis. Quantitative: triangulation of findings.	Sampling was not described clearly. However, the study used a semi-structured questionnaire that was designed in collaboration with maternity professionals and community researchers to explore the views and maternity experiences of newcomer women. Experienced multilingual female community researchers completed 82 of these questionnaires with interviewees in a range of different languages. Narrative interviews were also held with 13 women to further explore issues. The findings were triangulated with 18 interviews of professionals who regularly worked with migrant women.
[114]	To explore how Somali women with FGM experienced and perceived antenatal and intrapartum care in England.	Birmingham.	Qualitative: a descriptive, exploratory study using face-to-face semi-structured interviews that were audio-recorded.	Not specified.	Private room.	Qualitative: thematic analysis.	The study used convenience and snowball sampling of ten Somali women in Birmingham who had received antenatal care in England in the past 5 years.

For peer review only - http://bmjopen.bmj.com/site/about/guidelines.xhtml

Final

116	To explore differences in infant thermal care beliefs between mothers of South Asian and white British origin in Bradford, UK.	Bradford District, West Yorkshire.	Mixed methods: mothers were interviewed using a questionnaire with structured and unstructured questions.	Not specified.	The women chose the location of the interview.	Qualitative: thematic analysis.	A total of 102 mothers (51 South Asian and 51 white British) were recruited in Bradford District, West Yorkshire, UK. The inclusion criteria specified infants aged 13 months or less with a parent of South Asian or white British cultural origin who lived in the Bradford District. South Asia was defined as including the countries of Pakistan, India, Afghanistan, Sri Lanka and Nepal. Recruitment was aided by local community organisations, children's centres, and community contacts. Urdu- and Punjabi-speaking interpreters were requested and provided for 69 per cent of the first-generation South Asian mothers (n = 26) in the sample.
113	To gain an understanding of infant feeding practices among a group of UK-based refugee mothers.	Liverpool and Manchester.	Qualitative: two focus group discussions and 15 semi-structured interviews.	Not specified.	HCPs: private offices or clinics Refugee women: private rooms or discrete areas at the support venue (community centre or church hall).	Qualitative: thematic analysis.	The study purposively selected 30 refugee mothers from 19 countries who now resided in Liverpool or Manchester and were at least 6 months pregnant or had a child who had been born in the UK in the last 4 years. Of these 30, 19 were HIV-negative and 11 were HIV-positive.

For peer review only - http://bmjopen.bmj.com/site/about/guidelines.xhtml

Final

3	To provide insights into possible causes of poor maternity outcomes for new migrants in the West Midlands region of the UK and to develop recommendations that could help improve maternity services for these migrants.	West Midlands.	Mixed methods: a semi-structured questionnaire and in-depth interviews.	Not specified.	Not specified.	Qualitative: systematic thematic approach. Quantitative: triangulation of the findings.	A non-probability purposive sample was generated by selecting 82 women who had moved to the UK within the past 5 years and had subsequently utilised maternity services. Of these, 13 underwent in-depth interviews as well.
115	To explore the maternity care experiences of pregnant asylum-seeking women in West Yorkshire to inform service development.	West Yorkshire.	Qualitative: interpretative approach within the tradition of hermeneutic phenomenology.	Not specified.	Not specified.	Qualitative: interpretive approach with hermeneutic phenomenology analysis.	Purposive sampling was performed through the voluntary sector and a children's centre. In addition, word-of mouth led to an element of snowball sampling. Six women were recruited.
135	To provide locally applicable data on the needs of Black and minority ethnic women in relation to their uptake of maternity and neonatal care provision by primary healthcare teams in Leeds.	Leeds.	Mixed methods: questionnaires and focus groups. Interpreters were used when necessary for data collection. A questionnaire was translated into Urdu for some women.	Not specified.	Local community centres and in the participants' homes.	Qualitative: content analysis. Quantitative: survey (not clear).	A total of 97 questionnaires were completed, of which 50 were completed through informal links at community centres, schools, and in women homes. The remaining 47 were completed whilst the researcher attended various antenatal clinics in the community.

For peer review only - http://bmjopen.bmj.com/site/about/guidelines.xhtml

Final

117	To study the effectiveness of three linkworker and advocacy schemes that were designed to empower minority ethnic community users of maternity services.	Birmingham.	Qualitative: focus group discussions, semi-structured interviews, and non-directive interviews.	Not specified.	Antenatal clinics in hospitals and health centres, community group settings, and participants' homes.	Qualitative: not clear, thematic analysis?	Individual interviews were conducted with 66 Asian women who had received support from link-worker and advocacy services during their pregnancy and postnatally. Of these, 28 were from Birmingham, 13 from Leeds, and 25 from Wandsworth-London. A semi-structured interview guide was translated into five Asian languages: Hindi, Punjabi, Gujarati, Urdu and Tamil. The study also included ten focus groups made up of 60 women who had not used linkworker or advocacy services. All participants were recruited with the help of various minority ethnic women's groups and community organisations. Interpreters assisted 11 personal interviews with non-users from Vietnamese and Chinese backgrounds.
129	To determine the nature of the barriers confronting women when they used antenatal and postnatal services.	Pollokshields, Glasgow.	Qualitative: semi-structured questionnaire.	Not specified.	Not specified.	Qualitative: thematic analysis.	Twenty women were interviewed in depth by a Centre's Health Development Worker. Of these, 17 were born outside the UK.

For peer review only - http://bmjopen.bmj.com/site/about/guidelines.xhtml

Final

118	To study the maternity services experiences of Muslim parents in England.	UK: not specified.	Qualitative: focus groups with Muslim mothers to explore their experiences of and views about maternity services; questionnaires with Muslim fathers; and interviews with health professionals	Not specified.	Not specified.	Qualitative: content analysis.	A mixed sample of 43 immigrants and non-immigrants were recruited via their project advisory groups. The focus groups were conducted in various locations around the UK, with two focus group discussions in a language other than English. A total of eight health professionals were interviewed: six midwives (two of whom worked for Sure Start programmes), a health visitor, and a consultant obstetrician.
130	To determine the current clinical practice of maternity care in England, including the service provision and organisations that underpin care, from the perspective of women needing the care; to identify the key areas of concern for women receiving maternity care in England; and to determine whether and in what ways women's experiences and perceptions of care have changed over the last 10 years.	England: not specified.	Quantitative: survey.	Not specified.	Survey: not specified.	Quantitative: cross-sectional design.	Random samples of women selected for the pilot and main studies were identified by staff at the ONS using live birth registrations for births within 2 specific weeks: 2–8 January (pilot) and 4–10 March 2006 (main study). The same method of sampling was used as had been employed in 1995 to enable direct comparisons. Random samples of 400 women for the pilot survey and 4800 women for the main survey who were aged 16 years and over and who had delivered their baby in a one-week period in England were selected. The sampling was stratified on the basis of births in different geographical areas (Government Office Regions (GORs)). No subgroups were oversampled. The usable response rate was 60% for the pilot survey and 63% for the main survey. The samples included 229 women of BME born outside the UK.

For peer review only - http://bmjopen.bmj.com/site/about/guidelines.xhtml

Final

119	To explore the perceptions of pregnant asylum seekers in relation to the provision of their maternity care while in emergency accommodation in the UK.	South East of England.	Qualitative: an exploratory approach using unstructured interviews with five healthcare professionals and semi-structured interviews with ten pregnant asylum seekers.	Not specified.	Participants' emergency accommodat ions.	Qualitative: thematic analysis.	Purposive sampling of those providing maternity care for asylum seekers produced a sample comprising two midwives (M1 and M2), one GP (GP), one hospital consultant (C), and one nurse (N), all based in south coast health centres and hospitals. A total of 15 pregnant asylum seekers were approached to participate in the study. These women entered the UK through a south coast port over a three-month period. Their countries of origin were Algeria, Congo, Angola, Nigeria, Somalia, and Iraq, and they spoke French, Portuguese, Yoruba, Arabic, and Kurdish. Translated information letters and consent forms were distributed to pregnant asylum seekers via the Refugee Help Line, which also returned signed consent forms. This constitutes non-probability, purposive sampling.

For peer review only - http://bmjopen.bmj.com/site/about/guidelines.xhtml

Final

120	To explore the meanings attributed by migrant Arab Muslim women to their experiences of childbirth in the UK. In particular, to explore migrant Arab Muslim women's experiences of maternity services in the UK; to examine the traditional childbearing beliefs and practices of Arab Muslim society; and to suggest ways to provide culturally sensitive care for this group of women.	UK: not specified.	Qualitative: an interpretive ontological-phenomenological perspective informed by the philosophical tenets of Heidegger (1927/1962).	Heideggerian hermeneutic phenomenology.	All interviews were in the participants' homes except for one, which took place in a restaurant after 10 pm.	Qualitative: thematic analysis.	Purposive sampling produced eight Arab Muslim women who had migrated to one multicultural city in the Midlands.
121	To examine the health-seeking behaviours of Korean migrant women living in the UK.	London.	Qualitative: 21 semi-structured interviews.	Foucauldian approach.	Not clear.	Qualitative: not clear.	Women were recruited from New Malden via Korean community contacts.

For peer review only - http://bmjopen.bmj.com/site/about/guidelines.xhtml

Final

136	To explore perinatal clinical indicators and experiences of postnatal care among European and Middle Eastern migrant women and to compare them with those of British women at a tertiary hospital in the North East of Scotland.	North East of Scotland.	Mixed methods. Phase 1 of the research was a secondary analysis of routine data for 15,030 consecutive deliveries at Aberdeen Maternity Hospital. Phase 2 was a retrospective study of 26 European, Middle Eastern, and British mothers in this hospital. After the women had given birth, verbal data was collected using face-to-face semi-structured interviews.	Not clear.	Phase Two: 24 interviews were conducted in the homes of participants and two interviews at the University department.	Qualitative: thematic analysis. Quantitative: Phase One was a secondary analysis of routine data for 15,030 consecutive deliveries at Aberdeen Maternity Hospital. Phase 2 was a retrospective study of women.	Phase 1: The 15,030 deliveries included all births at Aberdeen Maternity Hospital over the financial years 2004–2008 in which maternal nationalities were identified and gestation was ≥ 24 weeks. Both singleton and multiple births were included. The clinical data was harvested from the Patient Administration System and the PROTOS maternity information system. In the case of women with multiple order births during the study, all births were included. Phase 2 of the research was a retrospective study of a few of the mothers who had given birth at this hospital. Eight European and five Middle Eastern women were semi-matched with 13 British women.

For peer review only

For peer review only - http://bmjopen.bmj.com/site/about/guidelines.xhtml

Final

137	To assess the mechanisms of support available to EM (ethnic minority) communities from community and voluntary sector organisations in relation to maternal and infant nutrition (a mapping exercise); to explore the experiences of the targeted client groups in seeking and receiving such support; and to identify gaps and opportunities to enhance support mechanisms and engagement with diverse EM communities.	Glasgow, Edinburgh, Aberdeen, Stirling, Fife, Dundee, and Inverness.	Mixed methods: an online questionnaire survey of organisations working with EM communities, focus groups, and telephone interviews with EM women.	Not specified.	Not specified.	Qualitative: thematic analysis. Quantitative:	The study identified 65 community organisations that potentially provided food and health services across EM communities in Scotland. In total, 37 organisations replied to the survey. Of those organisations, 15 indicated that they are providing services in the area of maternal and infant nutrition. A further 12 indicated that despite working with EM communities, they do not provide services in maternal and infant nutrition or healthy eating in general. An additional ten organisations confirmed by telephone that they were or had been working with EM women, but were unable to undertake the survey. The majority of interviewees for the focus groups and interviews were selected in response to a request sent by Black and Ethnic Minorities Infrastructure in Scotland (BEMIS) to community organisations. Snowball sampling was used to provide further contacts. In total, four focus groups were conducted with Polish, Roma, Czech, and African mothers. In addition, six telephone interviews were conducted with Polish mothers. We focused on Polish mothers because they were the largest new ethnic group in Scotland since 2004.

For peer review only

Final

122	To explore the experiences of obstetric care in Scotland among women who have undergone FGM.	Glasgow and Edinburgh.	Qualitative: personal experiences of FGM and interviews.	Interpretivism paradigm and feminist perspective.	The Dignity Alert & Research Forum (DARF) office or in the participant's home.	Qualitative: thematic analysis.	Convenience and purposive sampling resulted in a total number of seven women taking part in this study. All women were of African origin living in Scotland (three in Glasgow and four in Edinburgh). The inclusion criteria for the study were women who have undergone FGM and had experienced childbirth in Scotland. Three women were originally from Somalia, two from The Gambia, one from Ghana, and one from Sudan. Six of them were Muslims and one was Christian. All women had undergone FGM in their countries of origin. Four women had been infibulated and the remaining three could not tell if they have had FGM type 2 or 3.
123	To gain a rich understanding of migrant Pakistani Muslim women's experiences of postnatal depression within motherhood; to inform clinical practice; and to suggest ways of improving supportive services for this group.	East London.	Qualitative: interpretative phenomenology.	Interpretative phenomenological analysis (IPA) theory.	Not specified.	Qualitative: interpretative phenomenology.	Purposive sampling resulted in the recruitment of four migrant Pakistani Muslim women from London aged from 27 to 39.

For peer review only - http://bmjopen.bmj.com/site/about/guidelines.xhtml

Final

46	To explore the healthcare experience of vulnerable pregnant migrant women.	London.	Mixed methods: participants were contacted by phone (using a three-way interpreter call if appropriate) and interviewed using a pro forma questionnaire designed to determine their access to antenatal care; barriers to that access; and their experiences during pregnancy, labour, and the immediate postnatal period. Further data was extracted from their records at the Doctors of the World (DOTW) clinic to see how they had accessed the clinic.	Not specified.	Phone survey.	Qualitative: thematic analysis. Quantitative: not clear.	Pregnant women who presented to the drop-in clinic of the DOTW in London were approached between January 2013 and June 2014.

For peer review only - http://bmjopen.bmj.com/site/about/guidelines.xhtml

Final

124	To explore relationships between first-generation migrant Pakistani women and midwives in the South Wales region, focusing on the factors that contribute to these relationships and the ways that these factors might affect the women's experiences of care.	South Wales.	Qualitative: a focused ethnography.	Symbolic interactionism.	Midwives: at lunch break or between clinics. Pakistani women: not clear.	Qualitative: thematic analysis.	Purposive sampling, through midwife gatekeepers, was selected for the initial recruitment of pregnant migrant Pakistani women: emails were sent to all midwives working with migrant women in South Wales. Snowballing was then used to recruit other midwives eligible for participation. Focused, non-participant observations of antenatal booking appointments took place in antenatal clinics across the local health board region over a period of 3-6 months. A total of seven midwives and 15 women were observed during these appointments, which lasted 20-60 minutes each.

For peer review only

Table 3: Thick and Thin Criteria *Higginbottom et al* [19]

Richness	Operational Definition
Thick papers	• Offer greater explanatory insights into the outcome of interest<br>• Provide a clear account of the process by which the findings were produced—including the sample, its selection and its size, with any limitations or bias noted—along with clear methods of analysis<br>• Present a developed and plausible interpretation of the analysis based on the data presented.
Thin papers	• Offer only limited insights<br>• Lack a clear account of the process by which the findings were produced<br>• Present an underdeveloped and weak interpretation of the analysis based on the data presented

For peer review only - http://bmjopen.bmj.com/site/about/guidelines.xhtml

Final

Table 4: Quality appraisal of the included studies

Manual reference no.	Quality as per the CEBMa tool	Relevance	Thick/Thin
**1**	low	high	thin
**2**	low	high	thin
**3**	low	high	thin
**4**	low	high	thick
**5**	high	high	thick
**6**	med	high	thick
**7**	low	high	thin
**8**	low	high	thin
**9**	low	high	thin
**10**	med	high	thin
**11**	med	high	thin
**12**	med	high	thin
**13**	med	low	thin
**14**	med	med	thin
**15**	high	high	thin
**16**	high	high	thin
**17**	med	high	thin

For peer review only - http://bmjopen.bmj.com/site/about/guidelines.xhtml

Final

**18**	med	med	thick
**19**	high	high	thin
**20**	med	high	thick
**21**	high/medium	high	thin
**22**	high	high	thick
**23**	med	med	thin
**24**	high	high	thick
**25**	med	high	thin
**26**	high	high	thick
**27**	low	med	thin
**28**	med	high	thin
**29**	low	high	thin
**30**	med	med	thin
**31**	high	high	thick
**32**	low	high	thick
**33**	high	high	thick
**34**	low	high	thin
**35**	high	high	thick
**36**	low	low	thin
**37**	med	med	thin
**38**	med	high	thick
**39**	high	high	thin
**40**	high	high	thin/thick?

For peer review only - http://bmjopen.bmj.com/site/about/guidelines.xhtml

Final

**Table 5: Publications informing the themes**	1	2	3	4	5
	23	23	23	11	12
Bedew, H (2009). *Migrant Arab Muslim women's experiences of childbirth in the UK.*		X	X	X	X
Bazley Goodwin, LK (2016). *The midwife-woman relationship in a South Wales community: a focused ethnography of the experiences of midwives and migrant Pakistani women in early pregnancy.*	X	X	X		
Hicks, C., & Hayes, L. (1991). *Link-workers in antenatal care: facilitators of equal opportunities in health provision or salves for the management conscience?*	X		X	X	
Leeds Family Health (1992). *Research into the uptake of maternity services as provided by primary health care teams to women from black and minorities.*	X	X	X		

For peer review only - http://bmjopen.bmj.com/site/about/guidelines.xhtml

Final

Pershad, P., & Tyrrell, H. (1995). *Access to antenatal and postnatal services for Asian women living in East Pollokshields, Glasgow.*	X	X	X	X	
Warrier, S. (1996). *Consumer empowerment: a qualitative study of link-worker and advocacy services for non-English speaking users of maternity services.*	X	X	X	X	
Duff, L. A., Ahmed, L. B., & Lamping, D. L. (2002). *Evaluating satisfaction with maternity care in women from minority ethnic communities: development and validation of a Sylheti questionnaire.*			X		X
Harper Busman, K., & McCourt, C. (2002). *Somali refugee women's experiences of maternity care in west London: a case study.*	X	X	X	X	X
Ali, N. (2004). *Experiences of maternity services: Muslim women's perspectives.*	X	X	X	X	X
MacLeish, J. (2005). *Maternity experiences of asylum seekers in England.*	X	X	X	X	X
Ahmed, S., Macfarlane, A., Naylor, J., & Hastings, J. (2006). *Evaluating bilingual peer support for breastfeeding in a local sure start.*	X	X	X	X	
Redshaw, *et al.* (2007). *Recorded delivery: a national survey of women's experience of maternity care 2006.*		X	X	X	

For peer review only - http://bmjopen.bmj.com/site/about/guidelines.xhtml

Final

Nabb, J. (2006). *Pregnant asylum-seekers: Perceptions of maternity service provision.*	X	X		X	
Rowe, R. E., Magee, H., Quigley, M. A., Heron, P., Askham, J., & Brocklehurst, P. (2008). *Social and ethnic differences in attendance for antenatal care in England.*	X				
Hawkins, S. S., Lamb, K., Cole, T. J., & Law, C. (2008). *Influence of moving to the UK on maternal health behaviours: Prospective cohort study.*	X		X		
Briscoe, L., & Lavender, T. (2009).<br>*Exploring maternity care for asylum seekers and refugees.*			X	X	
Raine, R., Cartwright, M., Richens, Y., Mahamed, Z., & Smith, D. 2010). *A qualitative study of women's experiences of communication in antenatal care: identifying areas for action.*	X	X	X		
Tucker, A., Ogutu, D., Yoong, W., Nauta, M., & Fakokunde, A. (2010). *The unbooked mother: a cohort study of maternal and foetal outcomes in a North London Hospital.*	X		X		
Lee, J-Y (2010). *'My body is Korean, but not my child's...': a Foucauldian approach to Korean migrant women's health-seeking behaviours in the UK.*	X	X			
Cross-Sudworth, F., Williams, A., & Herron-Marx, S. (2011). *Maternity services in multi-cultural Britain: using Q methodology to explore the views of first- and second-generation women of Pakistani origin.*		X		X	X

For peer review only

Final

Essen, *et al.* (2011). ***An anthropological analysis of the perspectives of Somali women in the West and their obstetric care providers on caesarean birth.***		X			
Almalik, M. (2011). ***A comparative evaulation of postnatal care for migrant and UK-born women.***	X	X	X	X	
Binder, P., Borne, Y., Johnsdotter, S., & Essen, B. (2012a). ***Conceptualising the prevention of adverse obstetric outcomes among immigrants using the 'three delays' framework in a high-income context.***	X	X	X	X	
O'Shaughnessy, R., Nelki, J., Chiumento, A., Hassan, A., & Rahman, A. (2012). ***Sweet Mother: evaluation of a pilot mental health service for asylum-seeking mothers and babies.***				X	
Binder, P., Johnsdotter, S., & Essen, B. (2012b). ***Shared language is essential: communication in a multiethnic obstetric care setting.***		X	X		
Cresswell, J. A., Yu, G., Hatherall, B., Morris, J., Jamal, F., Harden, A., & Renton, A. (2013). ***Predictors of the timing of initiation of antenatal care in an ethnically diverse urban cohort in the UK.***	X		X		
Jomeen, J., & Redshaw, M. (2013). ***Ethnic minority women's experience of maternity services in England.***		X	X		X
BEMIS Scotland (2013). ***A comparative evaluation of postnatal care for migrant and UK-born women.***	X	X	X		

For peer review only

For peer review only - http://bmjopen.bmj.com/site/about/guidelines.xhtml

Final

Baldeh, F. (2013). *Obstetric Care in Scotland: the experience of women who have undergone Female Genital Mutilation (FGM).*		X	X	X	
Feldman, R. (2014). *When maternity doesn't matter: Dispersing pregnant women seeking asylum.*	X			X	
Gorman, D. R., Katikireddi, S. V., Morris, C., Chalmers, J. W. T., Sim, J., Szamotulska, K., . . . & Hughes, R. G. (2014). *Ethnic variation in maternity care: a comparison of Polish and Scottish women delivering in Scotland 2004-2009.*	X				
Greenhalgh, T., Clinch, M., Afsar, N., Choudhury, Y., Sudra, R., Campbell-Richards, D., & Finer, S. (2015). *Socio-cultural influences on the behaviour of South Asian women with diabetes in pregnancy: Qualitative study using a multi-level theoretical approach.*	X				
Phillimore, J. (2015). *Delivering maternity services in an era of superdiversity: The challenges of novelty and newness.*	X	X	X	X	X
Lamba, R. (2015). *A Qualitative Study Exploring Migrant Pakistani-Muslim Women's Lived Experiences and Understanding of Postnatal Depression.*		X	X		
Shortall, C., *et al.* (2015). *Experiences of Pregnant Migrant Women receiving Ante/Peri and Postnatal Care in the UK: A Doctors of the World Report on the Experiences of attendees at their London Drop-In Clinic.*	X		X	X	
Moxey, J. M. & L. L. Jones (2016). *A qualitative study exploring how Somali women exposed to female genital mutilation experience and perceive antenatal and intrapartum care in England.*	X	X	X	X	

For peer review only - http://bmjopen.bmj.com/site/about/guidelines.xhtml

Final

de Chavez, A. C., Ball, H. L., & Ward-Platt, M. (2016). *Bi-ethnic infant thermal care beliefs in Bradford, UK.*			X		
Hufton, E., & Raven, J. (2016). *Exploring the infant feeding practices of immigrant women in the North West of England: A case study of asylum seekers and refugees in Liverpool and Manchester.*		X		X	
Phillimore, J. (2016). *Migrant maternity in an era of superdiversity: New migrants' access to, and experience of, antenatal care in the West Midlands, UK.*	X			X	
Lephard, E., & Hait.h-Cooper, M. (2016).<br>*Pregnant and seeking asylum: Exploring women's experiences from booking to baby'.*	X	X	X	X	X

For peer review only - http://bmjopen.bmj.com/site/about/guidelines.xhtml

Final

References

1. Phillimore J. Delivering maternity services in an era of superdiversity: The challenges of novelty and newness. *Ethnic and Racial Studies* 2015; 38: 568-582. Health & Mental Health Services 3370. DOI: 10.1080/01419870.2015.980288 http://ovidsp.ovid.com/ovidweb.cgi?T=JS&PAGE=reference&D=psyc12&NEWS=N&AN=2015-02651-003; http://dx.doi.org/10.1080/01419870.2015.980288.

2. Phillimore J. Migrant maternity in an era of superdiversity: New migrants' access to, and experience of, antenatal care in the West Midlands, UK. *Social Science and Medicine* 2016; 148: 152-159. DOI: 10.1016/j.socscimed.2015.11.030. http://ovidsp.ovid.com/ovidweb.cgi?T=JS&PAGE=reference&D=medl&NEWS=N&AN=26705910; https://www.sciencedirect.com/science/article/pii/S0277953615302380?via%3Dihub.

3. Office of National Statistics. *Birth in England and Wales by Parents Country of Birth (2013)*. 2014. Available from https://www.ons.gov.uk/peoplepopulationandcommunity/birthsdeathsandmarriages/livebirths/bulletins/parentscountryofbirthenglandandwales/2014-08-28).

4. Manktelow B, Smith L, Seaton S, Hyman-Taylor P, Kurinczuk J, Field D, et al. *MBRRACE-UK Perinatal Mortality Surveillance Report, UK Perinatal Deaths for Births from January to December 2014*. 2016. Leicester: The Infant Mortality and Morbidity Studies, Department of Health Sciences, University of Leicester. Available from https://www.npeu.ox.ac.uk/downloads/files/mbrrace-uk/reports/MBRRACE-UK-PMS-Report-2014.pdf).

5. Department of Health. *Midwifery 2020: Delivering expectations*. 2010. Available from https://assets.publishing.service.gov.uk/government/uploads/system/uploads/attachment_data/file/216029/dh_119470.pdf).

6. Garcia R, Ali N, Papadopoulos C and Randhawa G. Specific antenatal interventions for Black, Asian and Minority Ethnic (BAME) pregnant women at high risk of poor birth outcomes in the United Kingdom: a scoping review. *BMC Pregnancy and Childbirth* 2015; 15: 226. DOI: 10.1186/s12884-015-0657-2.

For peer review only - http://bmjopen.bmj.com/site/about/guidelines.xhtml

Final

7. Higginbottom G, Morgan M, Alexandre M, Chiu Y, Forgeron J, Kocay D, et al. Immigrant women's experiences of maternity-care services in Canada: a systematic review using a narrative synthesis. *Systematic Reviews* 2015; 4: 13. DOI: 10.1186/2046-4053-4-13.
8. Salway SM, Higginbottom G, Reime B, Bharj KK, Chowbey P, Foster C, et al. Contributions and challenges of cross-national comparative research in migration, ethnicity and health: insights from a preliminary study of maternal health in Germany, Canada and the UK. *BMC Public Health* 2011; 11: 514. DOI: 10.1186/1471-2458-11-514.
9. Higginbottom GM, Safipour J, Yohani S, O'Brien B, Mumtaz Z and Paton P. An ethnographic study of communication challenges in maternity care for immigrant women in rural Alberta. *Midwifery* 2015; 31: 297-304. DOI: 10.1016/j.midw.2014.09.009.
10. Tobin CL, Di Napoli P and Beck CT. Refugee and Immigrant Women's Experience of Postpartum Depression: A Meta-Synthesis. *Journal of Transcultural Nursing* 2017: 84-100. DOI: 10.1177/1043659616686167.
11. Gagnon A ZM, Zeitlin J, Roam Collaboration. Migration to western industrialised countries and perinatal health: a systematic review. *Social Science and Medicine* 2009; 69: 934-946. DOI: 10.1016/j.socscimed.2009.06.027.
12. Gulliford M, Figueroa-Munoz J, Morgan M, Hughes D, Gibson B, Beech R, et al. What does 'access to health care' mean? *Journal of Health Services Research and Policy* 2002; 7: 186-188. DOI: 10.1258/135581902760082517.
13. Guendelman S, Buekens P, Blondel B, Kaminski M, Notzon FC and Masuy-Stroobant G. Birth outcomes of immigrant women in the United States, France, and Belgium. *Maternal and Child Health Journal* 1999; 3: 177-187. DOI: 10.1023/A:102232802.
14. Carolan M and Cassar L. Antenatal care perceptions of pregnant African women attending maternity services in Melbourne, Australia. *Midwifery* 2010; 26: 189-201. DOI: 10.1016/j.midw.2008.03.005.
15. Small R, Yelland J, Lumley J, Brown S and Liamputtong P. Immigrant women's views about care during labor and birth: an Australian study of Vietnamese, Turkish, and Filipino women. *Birth* 2002; 29: 266-277. DOI: 10.1046/j.1523-536X.2002.00201.x
16. Essén B, Johnsdotter S, Hovelius B, Gudmundsson S, Sjöberg NO, Friedman J, et al. Qualitative study of pregnancy and childbirth experiences in Somalian women resident in Sweden. *BJOG: An International Journal of Obstetrics and Gynaecology* 2000; 107: 1507-1512. DOI: 10.1111/j.1471-0528.2000.tb11676.x.
17. Binder P, Johnsdotter S and Essen B. Conceptualising the prevention of adverse obstetric outcomes among immigrants using the 'three delays' framework in a high-income context. *Social Science and Medicine* 2012; 75: 2028-2036. DOI: 10.1016/j.socscimed.2012.08.010. http://ovidsp.ovid.com/ovidweb.cgi?T=JS&PAGE=reference&D=med7&NEWS=N&AN=22940012.
18. Drewniak D, Krones T and Wild V. Do attitudes and behavior of health care professionals exacerbate health care disparities among immigrant and ethnic minority groups? An integrative literature review. *International Journal of Nursing Studies* 2017; 70: 89-98. DOI: 10.1016/j.ijnurstu.2017.02.015.
19. Mumtaz Z, O'Brien B and Higginbottom G. Navigating maternity health care: a survey of the Canadian prairie newcomer experience. *BMC Pregnancy and Childbirth* 2014; 14: 4. DOI: 10.1186/1471-2393-14-4.
20. Mullins T. Definition of 'immigrant'. In: Hussain B (ed). London: Migration Statistics Unit, Office for National Statistics, 2017.
21. The King's Fund. What do we know about the impact of immigration on the NHS? Available from https://www.kingsfund.org.uk/projects/verdict/what-do-we-know-about-impact-immigration-nhs (2015, accessed 08/08/2018).

For peer review only - http://bmjopen.bmj.com/site/about/guidelines.xhtml

Final

22. Jayaweera H. *Health of migrants in the UK: What do we know?* 2011. The Migration Observatory, University of Oxford. Available from https://migrationobservatory.ox.ac.uk/resources/briefings/health-of-migrants-in-the-uk-what-do-we-know/ (accessed 08/08/2018).
23. National Institute for Health and Clinical Excellence (NICE). *Pregnancy and complex social factors: A model for service provision for pregnant women with complex social factors*. 2010. Available from https://www.nice.org.uk/guidance/cg110).
24. Ramsden I. From facts to practice: cultural safety and nursing education in Aotearoa and Te Waipounamu. In: *Binan Goonj: bridging cultures in Aboriginal health*. 2nd ed. Sydney: Elsevier, 2009. https://www.elsevier.com/books/binan-goonj/eckermann/978-0-7295-3936-4
25. Ramsden I. *Cultural safety and nursing education in Aotearoa and Te Waipounamu*. PhD, Victoria University of Wellington New Zealand, 2002.
26. Papps E and Ramsden I. Cultural safety in nursing: The New Zealand experience. *International Journal for Quality in Health Care* 1996; 8: 491-497. DOI: 10.1093/intqhc/8.5.491.
27. Nursing Council of New Zealand. Guidelines for Cultural Safety, the Treaty of Waitangi, and Maori Health in Nursing and Midwifery Education and Practice. Available from http://www.nursingcouncil.org.nz/culturalsafety.pdf. (2002, accessed 26 February).
28. Brascoupé S and Catherine Waters BA M. Cultural safety: Exploring the applicability of the concept of cultural safety to Aboriginal health and community wellness. *International Journal of Indigenous Health* 2009; 5: 6. DOI: 10.18357/ijih52200912332.
29. Aboriginal Nurses Association of Canada. Cultural Competence and Cultural Safety in Nursing Education: A Framework for First Nations, Inuit and Métis Nursing. Available from https://www.cna-aiic.ca/~/media/cna/page-content/pdf-en/first_nations_framework_e.pdf (2009, accessed 26 February).
30. Popay J, Roberts H, Sowden A, Petticrew M, Arai L, Rodgers M, et al. *Guidance on the conduct of narrative synthesis in systematic reviews*. 2006. Available from http://citeseerx.ist.psu.edu/viewdoc/download?doi=10.1.1.178.3100&rep=rep1&type=pdf).
31. Booth A. *Supplementary Guidance for Inclusion of Qualitative Research in Cochrane Systematic Reviews of Interventions*. Version 1 (updated August 2011) ed.: Cochrane Collaboration Qualitative Methods Group, 2016.
32. Higginbottom GM, Morgan M, O'Mahony J, Chiu Y, Kocay D, Alexandre M, et al. Immigrant women's experiences of postpartum depression in Canada: a protocol for systematic review using a narrative synthesis. *Systematic Reviews* 2013; 2: 65. DOI: 10.1186/2046-4053-2-65.
33. Center for Evidence-Based Management. Critical Appraisal Tools & Questionnaires. Available from https://www.cebma.org/ (2017, accessed 15 February).
34. O'Cathain A. Reporting Mixed Methods Projects. In: *Mixed Methods Research for Nursing and the Health Sciences*. Blackwell, 2009, pp.135-158.
35. Roper JM SJ. *Ethnography in Nursing Research*. Sage Publications, 2000.
36. Tashakorri C TA. *Foundations in mixed methods research; Integrating Quantitative and Qualitative Approaches in the Social and Behavioral Sciences*. Thousand Oaks, Ca: Sage Publications, 2008.
37. Murphy E DR, Greatbatch D, Parker S, Watson P Qualitative research methods in health technology assessment: a review of the literature Health Technology Assessment. *Health Technology Assessment Reports* 1998; 2.
38. Moher D, Liberati A, Tetzlaff J, Altman DG and Group P. Preferred reporting items for systematic reviews and meta-analyses: the PRISMA statement. *PLoS Medicine* 2009; 6: e1000097. DOI: 10.1371/journal.pmed.1000097.

For peer review only - http://bmjopen.bmj.com/site/about/guidelines.xhtml

Final

39. Rowe RE, Magee H, Quigley MA, Heron P, Askham J and Brocklehurst P. Social and ethnic differences in attendance for antenatal care in England. *Public Health* 2008; 122: 1363-1372. Health & Mental Health Services 3370. DOI: 10.1016/j.puhe.2008.05.011. http://ovidsp.ovid.com/ovidweb.cgi?T=JS&PAGE=reference&D=psyc6&NEWS=N&AN=2009-02349-007;
http://dx.doi.org/10.1016/j.puhe.2008.05.011.
40. Hicks C and Hayes L. Linkworkers in antenatal care: facilitators of equal opportunities in health provision or salves for the management conscience? *Health Services Management Research* 1991; 4: 89-93. DOI: 10.1177/095148489100400202.
41. Hawkins SS, Lamb K, Cole TJ and Law C. Influence of moving to the UK on maternal health behaviours: Prospective cohort study. *BMJ* 2008; 336: 1052-1055. DOI: 10.1136/bmj.39532.688877.25.
http://ovidsp.ovid.com/ovidweb.cgi?T=JS&PAGE=reference&D=emed11&NEWS=N&AN=351692247.
42. Tucker A, Ogutu D, Yoong W, Nauta M and Fakokunde A. The unbooked mother: a cohort study of maternal and foetal outcomes in a North London Hospital. *Archives of Gynecology and Obstetrics* 2010; 281: 613-616. DOI: 10.1007/s00404-009-1152-7.
http://ovidsp.ovid.com/ovidweb.cgi?T=JS&PAGE=reference&D=med6&NEWS=N&AN=19551396.
43. Cresswell JA, Yu G, Hatherall B, Morris J, Jamal F, Harden A, et al. Predictors of the timing of initiation of antenatal care in an ethnically diverse urban cohort in the UK. *BMC Pregnancy and Childbirth* 2013; 13: 103. DOI: 10.1186/1471-2393-13-103.
http://ovidsp.ovid.com/ovidweb.cgi?T=JS&PAGE=reference&D=medl&NEWS=N&AN=23642084.
44. Gorman DR, Katikireddi SV, Morris C, Chalmers JWT, Sim J, Szamotulska K, et al. Ethnic variation in maternity care: a comparison of Polish and Scottish women delivering in Scotland 2004-2009. *Public Health* 2014; 128: 262-267. DOI: 10.1016/j.puhe.2013.11.008.
http://ovidsp.ovid.com/ovidweb.cgi?T=JS&PAGE=reference&D=medl&NEWS=N&AN=24613449.
45. Pershad P and Tyrrell H. *Access to antenatal and postnatal services for Asian women living in East Pollokshields, Glasgow*. 1995. Save the Children. Available from).
46. Redshaw M, Rowe R, Hockley C and Brocklehurst P. *Recorded delivery: a national survey of women's experience of maternity care 2006*. 2007. Oxford: National Perinatal Epidemiology Unit (NPEU). Available from https://www.npeu.ox.ac.uk/recorded-delivery).
47. Shortall C, McMorran J, Taylor K, Traianou A, Garcia de Frutos M, Jones L, et al. *Experiences of Pregnant Migrant Women receiving Ante/Peri and Postnatal Care in the UK: A Doctors of the World Report on the Experiences of attendees at their London Drop-In Clinic*. 2015. Available from
https://b.3cdn.net/droftheworld/08303864eb97b2d304_lam6brw4c.pdf).
48. Duff LA, Ahmed LB and Lamping DL. Evaluating satisfaction with maternity care in women from minority ethnic communities : development and validation of a Sylheti questionnaire. *International Journal for Quality in Health Care* 2002; 13: 215-230. DOI: 10.1093/intqhc/13.3.215.
49. Ahmed S, Macfarlane A, Naylor J and Hastings J. Evaluating bilingual peer support for breastfeeding in a local sure start. *British Journal of Midwifery* 2006; 14: 467-470. DOI: 10.12968/bjom.2006.14.8.21640.
http://search.ebscohost.com/login.aspx?direct=true&db=rzh&AN=106354580&site=ehost-live.
50. Cross-Sudworth F, Williams A and Herron-Marx S. Maternity services in multi-cultural Britain: Using Q methodology to explore the views of first- and second-generation

For peer review only - http://bmjopen.bmj.com/site/about/guidelines.xhtml

Final

women of Pakistani origin. *Midwifery* 2011; 27: 458-468. DOI: 10.1016/j.midw.2010.03.001. http://search.ebscohost.com/login.aspx?direct=true&db=rzh&AN=104661096&site=ehost-live.
51. O'Shaughnessy R, Nelki J, Chiumento A, Hassan A and Rahman A. Sweet Mother: evaluation of a pilot mental health service for asylum-seeking mothers and babies. *Journal of Public Mental Health* 2012; 11: 214-228. DOI: 10.1108/17465721211289392.
52. Leeds Family Health. *Research into the uptake of maternity services as provided by primary health care teams to women from black and minorities*. 1992. London: Leeds FHSA. Available from).
53. Almalik M. *A comparative evaluation of postnatal care for migrant and UK-born women*. PhD, University of Aberdeen, 2011.
54. BEMIS Scotland and Community Food and Health (Scotland). *Strengthening food work across ethnic minority communities: A focus on maternal and infant nutrition*. 2013. Available from https://www.communityfoodandhealth.org.uk/publications/strengthening-food-work-ethnic-minority-communities-focus-maternal-infant-nutrition/).
55. Jomeen J and Redshaw M. Ethnic minority women's experience of maternity services in England. *Ethnicity and Health* 2013; 18: 280-296. DOI: 10.1080/13557858.2012.730608. http://ovidsp.ovid.com/ovidweb.cgi?T=JS&PAGE=reference&D=medl&NEWS=N&AN=23039872.
56. Raine R, Cartwright M, Richens Y, Mahamed Z and Smith D. A qualitative study of women's experiences of communication in antenatal care: identifying areas for action. *Maternal and Child Health Journal* 2010; 14: 590-599. DOI: 10.1007/s10995-009-0489-7. http://ovidsp.ovid.com/ovidweb.cgi?T=JS&PAGE=reference&D=med6&NEWS=N&AN=19554436.
57. Greenhalgh T, Clinch M, Afsar N, Choudhury Y, Sudra R, Campbell-Richards D, et al. Socio-cultural influences on the behaviour of South Asian women with diabetes in pregnancy: Qualitative study using a multi-level theoretical approach. *BMC Medicine* 2015; 13: 120. DOI: 10.1186/s12916-015-0360-1. http://ovidsp.ovid.com/ovidweb.cgi?T=JS&PAGE=reference&D=emed17&NEWS=N&AN=604640620.
58. de Chavez AC, Ball HL and Ward-Platt M. Bi-ethnic infant thermal care beliefs in Bradford, UK. *International Journal of Human Rights in Healthcare* 2016; 9: 120-134. DOI: 10.1108/IJHRH-08-2015-0024. http://ovidsp.ovid.com/ovidweb.cgi?T=JS&PAGE=reference&D=psyc13&NEWS=N&AN=2016-25577-005.
59. Warrier S. *Consumer empowerment: a qualitative study of linkworker and advocacy services for non-English speaking users of maternity services*. Report no. 9780946741205, 1996. London: The Maternity Alliance. Available from).
60. Ali N. *Experiences of maternity services: Muslim women's perspectives*. 2004. London: The Maternity Alliance. Available from https://www.maternityaction.org.uk/wp-content/uploads/2013/09/muslimwomensexperiencesofmaternityservices.pdf).
61. Bawadi HA. *Migrant Arab Muslim women's experiences of childbirth in the UK*. PhD, De Montfort University, 2009.
62. Lee JY. *'My body is Korean, but not my child's...': a Foucauldian approach to Korean migrant women's health-seeking behaviours in the UK*. PhD, University of Nottingham, 2010.
63. Baldeh F. *Obstetric Care in Scotland: the experience of women who have undergone Female Genital Mutilation (FGM)*. MSc (Masters), Queen Margaret University, 2013.

For peer review only - http://bmjopen.bmj.com/site/about/guidelines.xhtml

Final

64. Lamba R. *A Qualitative Study Exploring Migrant Pakistani-Muslim Women's Lived Experiences and Understanding of Postnatal Depression*. PhD, University of East London, 2015.
65. McLeish J. Maternity experiences of asylum seekers in England. *British Journal of Midwifery* 2005; 13: 782-785. DOI: 10.12968/bjom.2005.13.12.20125. http://search.ebscohost.com/login.aspx?direct=true&db=rzh&AN=106416342&site=ehost-live.
66. Feldman R. When maternity doesn't matter: Dispersing pregnant women seeking asylum. *British Journal of Midwifery* 2014; 22: 23-28. DOI: 10.12968/bjom.2014.22.1.23. http://search.ebscohost.com/login.aspx?direct=true&db=rzh&AN=104006997&site=ehost-live.
67. Lephard E and Haith-Cooper M. Pregnant and seeking asylum: Exploring women's experiences' from booking to baby'. *British Journal of Midwifery* 2016; 24: 130-136. DOI: 10.12968/bjom.2016.24.2.130. https://www.magonlinelibrary.com/doi/10.12968/bjom.2016.24.2.130.
68. Nabb J. Pregnant asylum-seekers: Perceptions of maternity service provision. *Evidence-Based Midwifery* 2006; 4: 89-96. http://create.canterbury.ac.uk/5890/.
69. Harper Bulman K and McCourt C. Somali refugee women's experiences of maternity care in west London: a case study. *Critical Public Health* 2002; 12: 365-380. DOI: 10.1080/0958159021000029568. http://ovidsp.ovid.com/ovidweb.cgi?T=JS&PAGE=reference&D=mwic&NEWS=N&AN=2003020613.
70. Hufton E and Raven J. Exploring the infant feeding practices of immigrant women in the North West of England: A case study of asylum seekers and refugees in Liverpool and Manchester. *Maternal and Child Nutrition* 2016; 12: 299-313. DOI: 10.1111/mcn.12145. http://onlinelibrary.wiley.com/journal/10.1111/(ISSN)1740-8709 http://ovidsp.ovid.com/ovidweb.cgi?T=JS&PAGE=reference&D=emed18a&NEWS=N&AN=609162428.
71. Moxey JM and Jones LL. A qualitative study exploring how Somali women exposed to female genital mutilation experience and perceive antenatal and intrapartum care in England. *BMJ Open* 2016; 6: e009846. DOI: 10.1136/bmjopen-2015-009846.
72. Essén B, Binder P and Johnsdotter S. An anthropological analysis of the perspectives of Somali women in the West and their obstetric care providers on caesarean birth. *Journal of Psychosomatic Obstetrics and Gynaecology* 2011; 32: 10-18. DOI: 10.3109/0167482X.2010.547966.
73. Binder P, Borne Y, Johnsdotter S and Essen B. Shared language is essential: communication in a multiethnic obstetric care setting. *Journal of Health Communication* 2012; 17: 1171-1186. DOI: 10.1080/10810730.2012.665421. http://ovidsp.ovid.com/ovidweb.cgi?T=JS&PAGE=reference&D=emed14&NEWS=N&AN=366382910.
74. Goodwin L. *The midwife-woman relationship in a South Wales community: a focused ethnography of the experiences of midwives and migrant Pakistani women in early pregnancy*. PhD, Cardiff University, 2016.
75. Briscoe L and Lavender T. Exploring maternity care for asylum seekers and refugees. *British Journal of Midwifery* 2009; 17: 17-24. DOI: 10.12968/bjom.2009.17.1.37649. http://search.ebscohost.com/login.aspx?direct=true&db=rzh&AN=105631311&site=ehost-live.

For peer review only - http://bmjopen.bmj.com/site/about/guidelines.xhtml

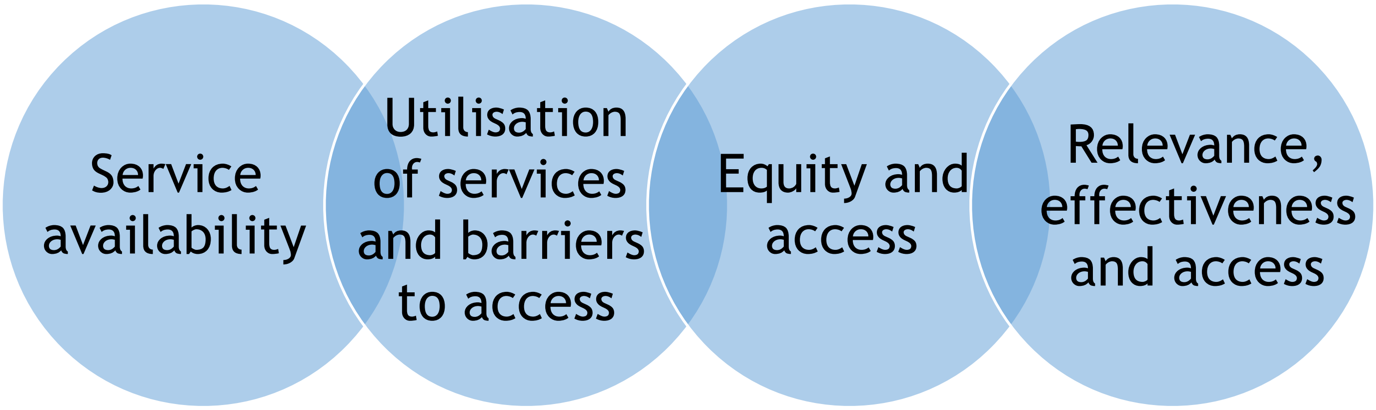

Type to enter text

Type to enter text

Figure1: Gulliford *et al.* Theory of access

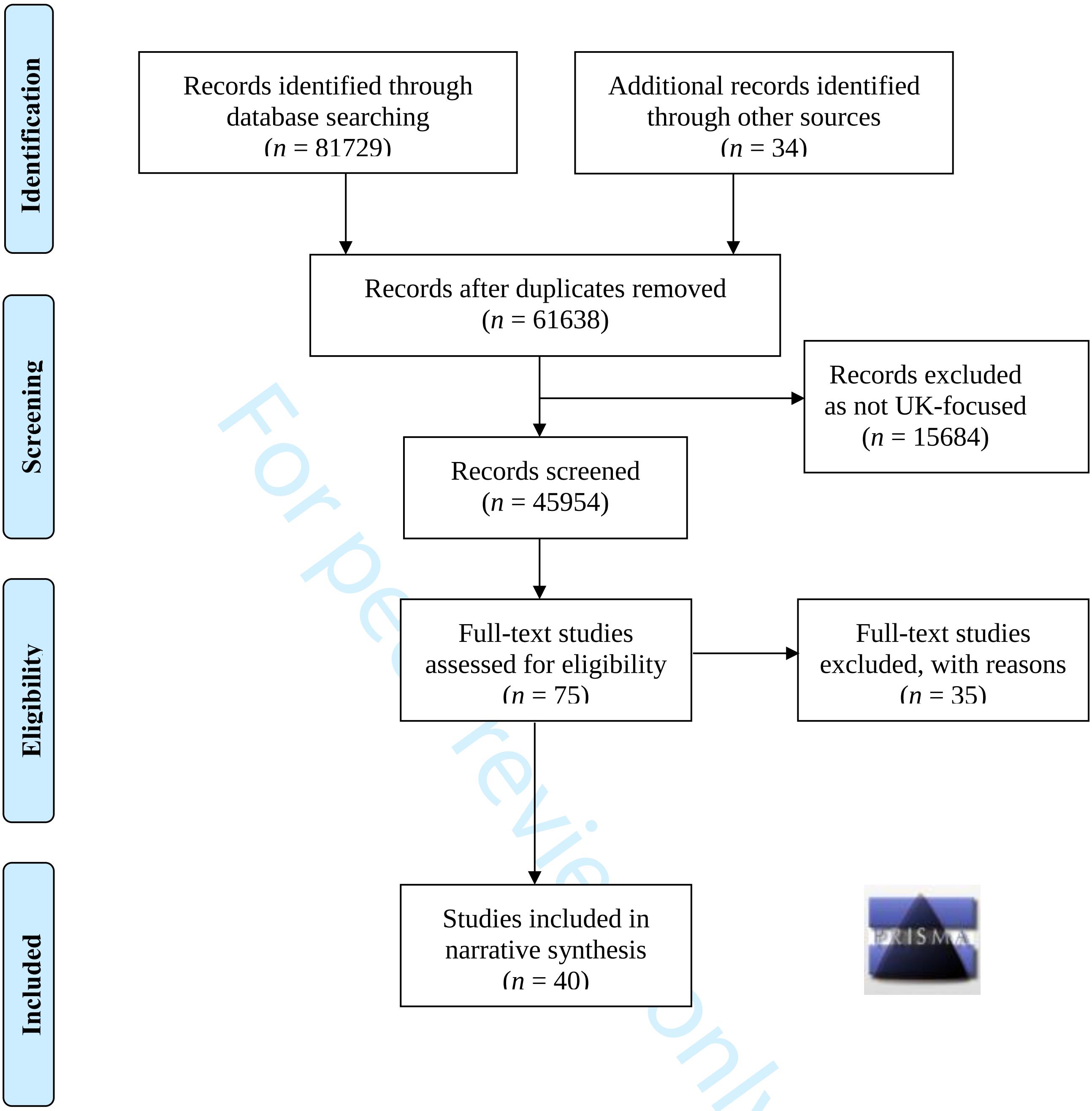

Figure 2: PRISMA flow diagram of the final selection process.

For peer review only

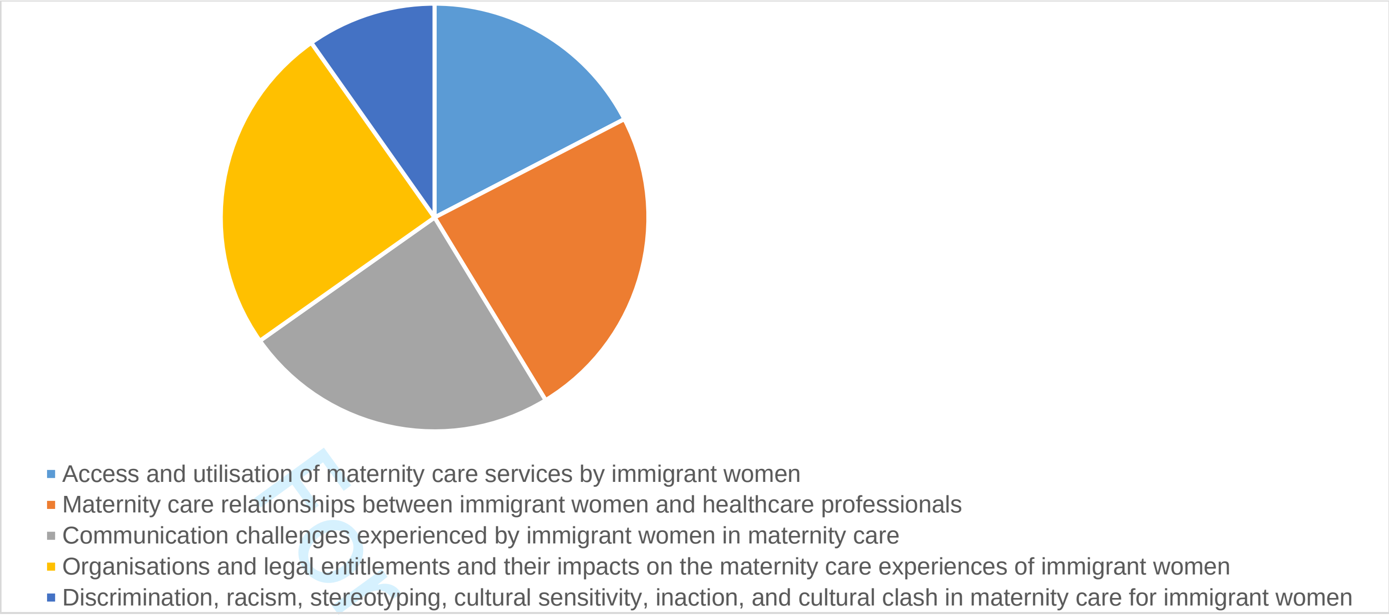

.***Figure 3:*** *The* total numbers of studies involved in each theme

For peer review only

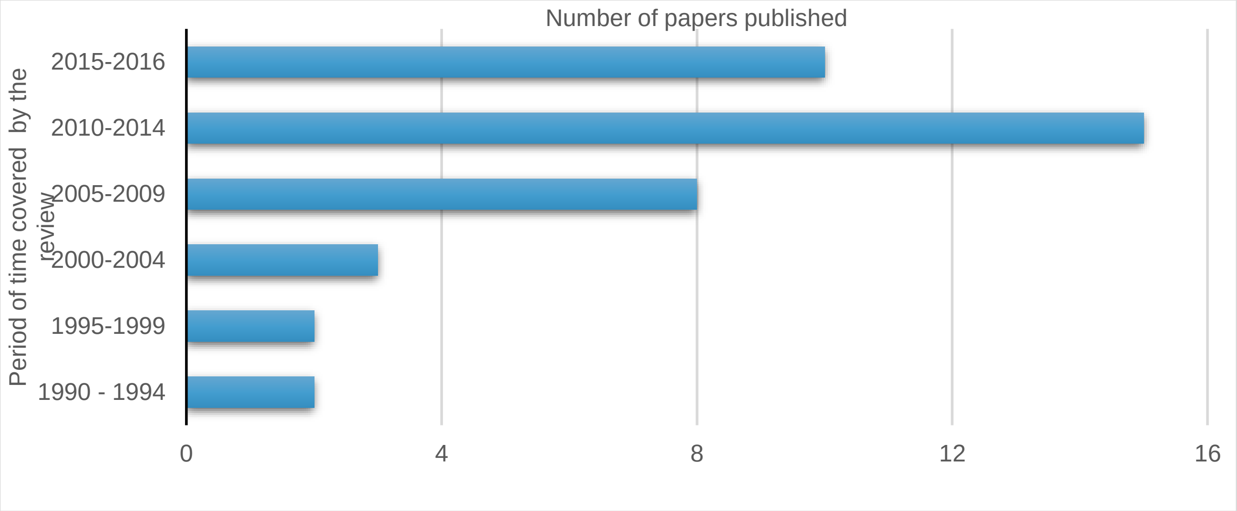

Figure 4: The range of publication dates for the included studies (1990–2016).

For peer review only

Figure 5: Immigrant women's experiences of maternity care in the UK

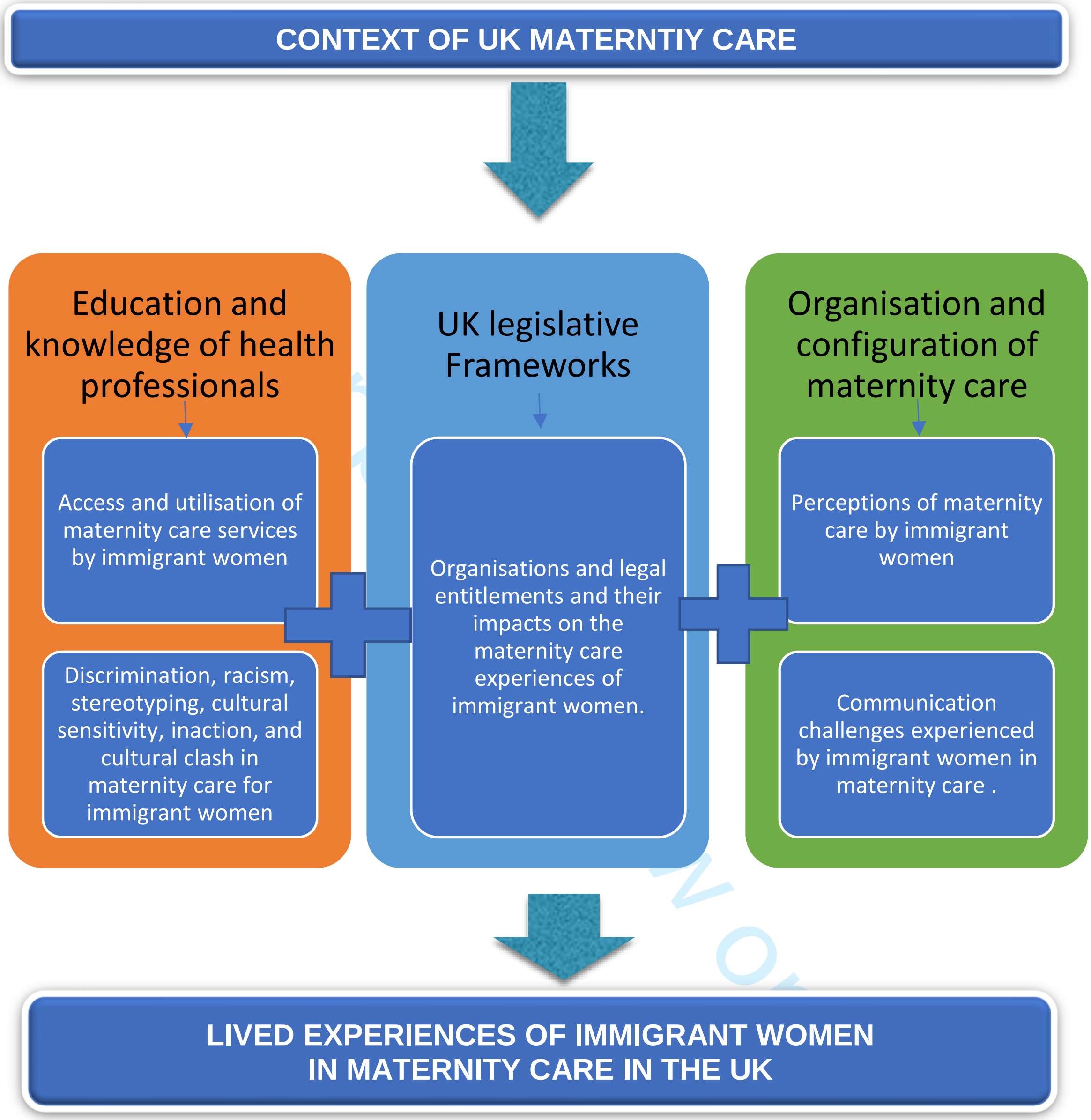

SUPPLEMENTARY FILES
File 1: Search Strategy
File 2: Bibliographic databases searched
File 3: Inclusion/Exclusion critiera
File 4: Excluded papers and rationale
File 5: Master table of included studies
File 6: Characteristics of study participants

For peer review only

File 1: Search Strategy

:Review search strategy - Medline

1	Maternal Health Services/ or Postnatal Care/ or Preconception Care/ or Prenatal Care/ or Perinatal Care/ or Infant Care/ or Midwifery/ or Obstetrics/ or General Practitioners/ or Primary Health Care/ or Family Health/	162335	
2	((maternal or child* or baby or babies or fetus* or fetal* or	119288	Field modified from .mp. to .ti,ab.
3	((birth* or matern* or mother* or pregnan* or childbearing or child-bearing or prenatal or pre-natal or postnatal or post-natal or perinatal or peri-natal or **preconception** or pre-conception or antenatal or ante-natal or postpartum or puerperium) adj3 (health* or nurs* or care or service*)).ti,ab.	65240	Field modified from .mp. to .ti,ab.
4	exp Midwifery/ or exp Obstetric Nursing/ or exp	47851	Field modified from .mp. to .ti,ab.
5	exp Health Services Accessibility/ or exp	1829149	
6	5 and (matern* or child* or baby or babies or fetus* or fetal* or embryo* or obstetric* or birth* or mother* or pregnan* or childbearing or child-bearing or prenatal or pre-natal or postnatal or post-natal or perinatal or peri-natal or preconception or pre-conception or antenatal or ante-natal or postpartum or puerperium).ti,ab.	253992	Limit 5 to female did not sufficiently focus previous search strategy so text terms used instead; Field modified from .mp. to .ti,ab.
7	1 or 2 or 3 or 4 or 6	490776	
8	("use" or access* or utili* or consum* or block* or hurdl* or barrier* or hindr* or hinder* or obstacle* or exclu* or discrimin* or disparit* or disproportion* or inequal* or unequal* or inadequat* or insuffic* or stratif* or limit* or lack* or unreliab* or poor* or poverty* or depriv* or disadvantag* or insecur* or insensit* or status* or entitl* or uninform* or ill-inform* or benefit* or interven* or deliver* or effective* or cost effective*).ti,ab.	8190143	

9	5 and 8	761677	use of/access to health services
10	"Emigrants and Immigrants"/ or Refugees/ or "Transients and Migrants"/ or "Emigration and Immigration"/	42486	
11	(((established or "first generation*" or new* or recent* or current*) adj3 (migrant* or migrat* or immigrant* or immigrat* or emigrant* or emigrat* or emigre* or expat* or (ex adj pat*) or transient* or alien*)) or newcomer* or (new adj comer*) or incomer*	14965	Revised to focus on established or new immigrant groups; Field modified from .mp. to .ti,ab.
12	(refugee* or (asylum adj seek*) or asylee* or (refused adj3 (asylum* or refugee*)) or (displaced adj person*) or exile* or (new adj arrival) or (country adj2 (birth or origin)) or **transnational*).ti,ab.**	13603	Field modified from .mp. to .ti,ab.
13	(foreigner* or (foreign adj (born or citizen* or national* or origin*)) or (non adj (citizen* or native*)) or ((adoptive or naturali#ed) adj (citizen* or resident*)) or overstay* or trafficked or "spousal migrant*").ti,ab.	10542	Additional migrants terminology; Field modified from .mp. to .ti,ab.
14	("non-UK-born" or "born outside the UK" or "length of residence in the UK" or (("not lawful*" or "not legal*" or unlawful* or illegal* or unauthori#ed* or "not authori#ed" or uncertain or insecure or illegal or legal or irregular* or refused or **undocumented)** adj3 (residen* or immigrant* or imigrat* or migrant* or migrat*))).ti,ab.	1375	Additional migrants terminology; Field modified from .mp. to .ti,ab.
15	exp Ethnic Groups/ or (ethnic* or ethno* or race or racial*).ti,ab.	282908	Expanded ethnic terminology; Field modified
16	exp african continental ancestry group/ or exp asian continental ancestry group/ or exp Caribbean Region/	152457	Additional ethnic terminology to specify South Asian and African Caribbean groups;
17	exp Vulnerable Populations/ or ((vulnerab* or disadvantag* or minorit*) adj3 (individ* or	35822	Expanded vulnerable populations terminology; Field

For peer review only

18	("Black and Minority Ethnic" or "Black & Minority ethnic" or BME or african caribbean* or afro caribbean* or black african* or (west adj (indies or indian*))).ti,ab.	7587	Expanded ethnic terminology; Field modified from .mp. to .ti,ab.
19	(south asia* or afghan* or bangladesh*	163384	
20	10 or 11 or 12 or 13 or 14 or 15 or 16 or 17 or 18 or 19	589408	All ethnic/migrant groups
21	7 and 9 and 20	20754	Maternity health services AND [illegible]
22	limit 21 to yr="1990 -Current"	18783	Time range expanded
23	higginbottom*.au.	214	
24	22 and 23	9	Check of strategy retrieval of known relevant records

For peer review only

File 2: Bibliographic databases searched

Databases searched.

- Ovid MEDLINE 1948– and MEDLINE In-Process and Other Non-Indexed Citations to daily update
- Ovid EMBASE 1980–2017 Week 11
- Ovid PsycINFO 1972–March Week 3 2017
- CINAHL Plus with Full Text/EBSCOHost to 2017
- MIDIRS on Ovid 1971 to April 2017
- Thomson Reuters Web of Science* 1900–2017
- ASSIA on ProQuest 1987–current
- HMIC on Ovid 1979–January 2017
- POPline (via http:// www.popline.org/) 1970 to the present

* Thomson Reuters Web of Science 1900-2017 includes the following:

- Science Citation Index Expanded (SCI-EXPANDED) 1900–2017
- Social Sciences Citation Index (SSCI) 1956–2017
- Conference Proceedings Citation Index - Science (CPCI-S) 1990–2017
- Conference Proceedings Citation Index - Social Science and Humanities (CPCI-SSH) 1990–2017
- Book Citation Index - Science (BKCI-S) 2008–2017
- Book Citation Index - Social Science and Humanities (BKCI-SSH) 2008–2017
- Emerging Sources Citation Index (ESCI) - 2015–2017

For peer review only

List of databases for searching grey literature

- **Cochrane Database of Systematic Reviews**
  http://www.thecochranelibrary.com/

Theses

- **Nottingham eDissertations**
  http://edissertations.nottingham.ac.uk/
- selected dissertations from UoN

- **Nottingham eTheses**
  http://etheses.nottingham.ac.uk/
- research degree theses awarded by UoN
- pilot project so not compulsory to submit, therefore not all these included

- **Index to Theses**
  http://www.theses.com/
  theses (incl. abstracts) accepted for higher degrees by universities in GB and Ireland
  now part of ProQuest Dissertations & Theses – UK & Ireland

- **Networked Digital Library of Theses & Dissertations**
  http://www.ndltd.org/
- includes theses and dissertations submitted to over 200 universities worldwide

- **EThOS – British Library Electronic Theses Online**
  http://ethos.bl.uk/Home.do

- **DEEP – DART Europe**
  http://www.dart-europe.eu

- **ProQuest Dissertations & Theses A&I** (worldwide coverage)
  http://search.proquest.com/pqdt/index?accountid=8018

Research Funders

- **Wellcome Trust**
  http://www.wellcome.ac.uk/
- Global charitable foundation supporting biomedical research and the medical humanities
- Provides support with funding, managing grants, education resources, application of research

- **Research Councils UK**
  http://www.rcuk.ac.uk/
- Support research across all academic disciplines
- Offer funding opportunities, international collaborations and training

For peer review only

- **Medical Research Council**
  http://www.mrc.ac.uk/index.htm
- Publicly funded organisation dedicated to improving human health
- Supports research across medical sciences in universities, hospitals and MRC Councils

- **Science and Technology Facilities Council**
  http://www.stfc.ac.uk/
- Independent public body of the Department of Business, Innovations and Skills
- Supports researchers across the sciences with the academic and industrial communities

- **National Institute for Health and Care Excellence**
  http://www.nice.org.uk/

- **Institute for Public Policy and Research**
  http://www.ippr.org/

- **ESRC**
  http://www.esrc.ac.uk/
  REGARD database

- **Clinical Research Network** (part of the NHS National Institute for Health Research)
  http://www.crncc.nihr.ac.uk
  From the NIHR portal
  (https://portal.nihr.ac.uk/Pages/NIHRResearchInfoStatement.aspx):
  - The repository for this information is the Portfolio Database, which currently contains approximately 2,000 studies, and can be accessed for public searching. Detailed instructions on how to search the Portfolio Database are available at http://www.ukcrn.org.uk/index/clinical/portfolio_new/P_search.html, and the Portfolio database is available at http://public.ukcrn.org.uk/search.
  - The National Research Register has been archived as a public resource and to support historical analysis. The archive is available via the National Institute for Health Research Portal http://portal.nihr.ac.uk.

Statistics

- **Department of Health**
  https://www.gov.uk/government/publications?publication_filter_option=statistics

- **UK Data Archive**
  http://www.data-archive.ac.uk

- **UK National Statistics**
  http://www.statistics.gov.uk

- **OECD Statistics Portal**
  http://www.oecd.org/statistics

- **World Health Organisation**

For peer review only

http://www.euro.who.int/en

- **NICE Evidence Services** (formerly NHS Evidence)
- Evidence search
- Clinical Knowledge Summaries
- NICE guidelines
- Journals and databases
- A-Z of topics – e.g. Diabetes
- Medicines information
- Public health information

https://www.evidence.nhs.uk

- **HMIC** (Health Management Information Consortium) – on Ovid
- combined database of the Department of Health, plus the King's Fund Information & Library Service
- official publications, journal articles, grey literature
- health service policy, management & admin, quality of hospitals, nursing, primary care and public health; occupational health; control/regulation of medicines

- **PAIS International**

  http://search.proquest.com/pais?accountid=8018 (via ProQuest)
- includes e.g.: gov docs, statistical directories, grey lit, research reports – mostly in social sciences

- **Open Grey**

  http://www.opengrey.eu/
- open access to grey literature published in Europe, including reports, dissertations, conference proceedings, official publications

- **Mednar**

  http://mednar.com/mednar/desktop/en/green/search.html

  one-stop federated search engine designed for professional medical researchers

- **WorldwideScience**

  http://worldwidescience.org/

- **OAIster**

  http://www.oclc.org/oaister.en.html

  catalog of millions of records from open access collections worldwide using the Open Archives Initiative Protocol for Metadata Harvesting (OAI-PMH)

- **Internet Archive Wayback Machine**

  http://archive.org/web/

  aims to provide permanent access to historical collections that exist in digital format

For peer review only

For peer review only

File 3: Inclusion/Exclusion criteria

Inclusion Criteria

Population	Immigrant women from any country other than England, Scotland, Northern Ireland or Wales
Phenomena of Interest	Maternity care
Context Setting	United Kingdom
Study designs	Qualitative, quantitative and mixed methods studies
Language	English
Date limitations	Jan 1990 - Jan 2018
**Exclusion Criteria**	
Context	Studies located in any country other than England, Scotland, Northern Ireland or Wales
Participants	Black and minority ethnic women born in the United Kingdom
Study Design	Non-empirical research, opinion pieces or editorial

For peer review only

File 4: Excluded papers and rationale

Excluded studies with reasons for exclusion.

Exclusion number	Reference	Reasons for exclusion
**1**	Bowler I. 'They're not the same as us': midwives' stereotypes of South Asian descent maternity patients. *Sociol Health Illn*. 1993 Mar 1;15(2):157-78.	Presented professionals' perspectives: focused on midwife interviews and observational data on midwives.
**2**	Straus L, McEwen A, Hussein FM. Somali women's experience of childbirth in the UK: perspectives from Somali health workers. *Midwifery*. 2 09 Apr 1;25(2):181-6.	Presented professionals' perspectives: interviewed Somali health workers and not the immigrant women.
**3**	Bowler IM. Stereotypes of women of Asian descent in midwifery: some evidence. *Midwifery*. 1993 Mar 1;9(1):7-16.	Presented professionals' perspectives: interviewed midwives.
**4**	Haith-Cooper M, Bradshaw G. Meeting the health and social needs of pregnant asylum seekers: midwifery students' perspectives. Part 2: Dominant discourses and approaches to care. *Nurse Educ Today*. 2013 Aug 1;33(8):772-7.	Presented professionals' perspectives: focused on midwifery students' perceptions.
**5**	Haith-Cooper M, Bradshaw G. Meeting the health and social care needs of pregnant asylum seekers; midwifery students' perspectives: Part 3; The pregnant woman within the global context; an inclusive model for midwifery education to address the needs of asylum-seeking women in the UK. *Nurse Educ Today*. 2013 Sep 1;33(9):1045-50.	Presented professionals' perspectives: interviewed midwives.
**6**	Balaam MC, Kingdon C, Thomson G, Finlayson K, Downe S. 'We make them feel special': the experiences of voluntary sector workers supporting asylum-seeking and refugee women during pregnancy and early motherhood. *Midwifery*. 2016 Mar 1;34:133-40.	Presented professionals' perspectives.
**7**	Richards J, Kliner M, Brierley S, Stroud L. Maternal and infant health of Eastern Europeans in Bradford, UK: a qualitative study. *Community Practitioner*. 2014 Sep 1;87(9):33.	Presented professionals' perspectives.
**8**	Redshaw M, Heikkilä K. Ethnic differences in women's worries about labour and birth. *Ethn Health*. 2011 Jun 1;16(3):213-23.	Mixed sample of UK-born BME and immigrant women with no separate findings reported for immigrant women.

**9**	Darwin Z, Green J, McLeish J, Willmot H, Spiby H. Evaluation of trained volunteer doula services for disadvantaged women in five areas in England: women's experiences. *Health Social Care Community*. 2017 Mar 1;25(2):466-77.	Mixed sample of UK-born BME and immigrant women with no separate findings reported for immigrant women.
**10**	Dunne FP, Brydon PA, Proffitt M, Smith T, Gee H, Holder RL. Fetal and maternal outcomes in Indo-Asian compared to Caucasian women with diabetes in pregnancy. *QJM*. 2000 Dec 1;93(12):813-8.	Mixed sample of Indo-Asian women born inside and outside the UK with no separate findings for immigrant women.
**11**	Ball HL, Moya E, Fairley L, Westman J, Oddie S, Wright J. Infant care practices related to sudden infant death syndrome in South Asian and White British families in the UK. *Paediatr Perinat Epidemiol*. 2012 Jan 1;26(1):3-12.	Focused on care of infants aged 2-4 months, but our chosen limit of maternity care was only up to 6 weeks after birth.<br>Mixed sample of UK-born and non-UK-born women with no separate findings for immigrant women.
**12**	McCarthy R, Haith-Cooper M. Evaluating the impact of befriending for pregnant asylum-seeking and refugee women. *Br J Midwifery*. 2013 Jun;21(6):404-9.	Not an empirical: the study does not report its methodology, sampling, or data analysis.
**13**	Streetly A, Grant C, Bickler G, Eldridge P, Bird S, Griffiths W. Variation in coverage by ethnic group of neonatal (Guthrie) screening programme in south London. *BMJ*. 1994 Aug 6;309(6951):372-4.	Mixed sample of ethnic groups with no separate findings for immigrant women.
**14**	Burchill J, Pevalin DJ. Demonstrating cultural competence within health-visiting practice: working with refugee and asylum-seeking families. *Divers Equal Health Care*. 2014;11(2).	Weak focus on maternity care: just two quotes on the influence of cultural sensitivity training and on addressing female genital mutilation (FGM) in maternity.
**15**	Dormandy E, Michie S, Hooper R, Marteau TM. Low uptake of prenatal screening for Down syndrome in minority ethnic groups and socially deprived groups: a reflection of women's attitudes or a failure to facilitate informed choices? *Int J Epidemiol*. 2005 Feb 28;34(2):346-52.	Not clear if sample was composed of immigrant women: no separate findings for immigrant women.
**16**	Henderson J, Gao H, Redshaw M. Experiencing maternity care: the care received and perceptions of women from different ethnic groups. *BMC Pregnancy Childbirth*. 2013 Dec;13(1):196.	Not clear if sample was composed of immigrant women: no separate findings for immigrant women.
**17**	Ingram J, Cann K, Peacock J, Potter B. Exploring the barriers to exclusive breastfeeding in Black and minority ethnic groups and young mothers in the UK. *Matern Child Nutr*. 2008 Jul 1;4(3):171-80.	Mixed sample of UK-born and immigrant women with no separate findings for immigrant women.

**18**	Parsons L, Day S. Improving obstetric outcomes in ethnic minorities: an evaluation of health advocacy in Hackney. *J Public Health*. 1992 Jun 1;14(2):183-91.	Not clear if sample was composed of immigrant women.
**19**	Knight M, Kurinczuk JJ, Spark P, Brocklehurst P. Inequalities in maternal health: national cohort study of ethnic variation in severe maternal morbidities. *BMJ*. 2009 Mar 4;338:b542.	Mixed sample of both UK- and foreign-born BME with no separate findings for immigrant women.
**20**	Almond P, Lathlean J. Inequity in provision of and access to health-visiting postnatal depression services. *J Adv Nurs*. 2011 Nov 1;67(11):2350-62.	Focused on professionals' perspective. Eight of the nine participants were immigrant women, but just three brief quotes were reported from immigrant Bangladeshi women. Authors did not reply to our request for clarification of the immigrant status of the sample.
**21**	Row MA, Nevill AM, Young DB, Adamson-Macedo EN. (2013) Promoting positive postpartum mental health through exercise in ethnically diverse priority groups. *Divers Equal Health Care*. 2013;10(3)185-195.	Mixed sample of minority ethnicity women born in and outside the UK with no separate findings for immigrant women.
**22**	Hemingway H, Saunders D, Parsons L. Social class, spoken language and pattern of care as determinants of continuity of carer in maternity services in east London. *J Public Health*. 1997 Jun 1;19(2):156-61.	Mixed sample of women with and without English as a first language. We used lack of English as a proxy for immigrant, but only one finding was reported for a non-English sample (i.e., the presence of an advocate who could translate for women visiting midwives or doctors). Did not receive a reply from the authors regarding the immigrant status of the sample.
**23**	Ingram J, Johnson D, Hamid N. South Asian grandmothers' influence on breast feeding in Bristol. *Midwifery*. 2003 Dec 1;19(4):318-27.	No clarity on the immigrant status of the sample and no separate findings for immigrant women. Did not receive a reply from the authors on the immigrant status of the sample.
**24**	Gardner PL, Bunton P, Edge D, Wittkowski A. The experience of postnatal depression in West African mothers living in the United Kingdom: A qualitative study. *Midwifery*. 2014 Jun 1;30(6):756-63.	No clarity on the immigrant status of the sample and no separate findings for immigrant women.
**25**	Kelly Y, Panico L, Bartley M, Marmot M, Nazroo J, Sacker A. Why does birthweight vary among ethnic groups in the UK? Findings from the Millennium Cohort Study. *J Public Health*. 2008 Jul 21;31(1):131-7.	Not clear if participants included immigrant women. Did not receive a reply from the authors on the immigrant status of the sample.

**26**	Beake S, McCourt C, Bick D. Women's views of hospital and community-based postnatal care: the good, the bad and the indifferent. *Evid Based Midwifery*. 2005 Dec 1;3(2):80-7.	Not clear if participants included immigrant women. Did not receive a reply from the authors on the immigrant status of the sample.
**27**	Ahmed S, Green J, Hewison J. Antenatal thalassaemia carrier testing: women's perceptions of information and consent. *J Med Screen*. 2005 Jun 1;12(2):69-77.	Weak focus on maternity care: main focus was on an ancestry issue.
**28**	Dyson SM, Cochran F, Culley L, Dyson SE, Kennefick A, Kirkham M, Morris P, Sutton F, Squire P. Ethnicity questions and antenatal screening for sickle cell/thalassaemia (EQUANS) in England: observation and interview study. *Crit Public Health*. 2007 Mar 1;17(1):31-43.	Not clear if participants included immigrant women.
**29**	Baker D, Garrow A, Shiels C. Inequalities in immunisation and breast feeding in an ethnically diverse urban area: cross-sectional study in Manchester, UK. *J Epidemiol Community Health*. 2011 Apr 1;65(4):346-52.	Not clear if participants included immigrant women and not focused on maternity care. Did not receive a reply from the authors on the immigrant status of the sample.
**30**	Dyson SM, Chambers K, Gawler S, Hubbard S, Jivanji V, Sutton F, Squire P. Lessons for intermediate- and low-prevalence areas in England from the Ethnicity Questions and Antenatal Screening for sickle cell/thalassaemia (EQUANS) study. *Divers Health Social Care*. 2007 Jun 1;4(2).	Not clear if participants included immigrant women and not focused on maternity care.
**31**	Sim JA, Ulanika AA, Katikireddi SV, Gorman D. 'Out of two bad choices, I took the slightly better one': Vaccination dilemmas for Scottish and Polish migrant women during the H1N1 influenza pandemic. *Public Health*. 2011 Aug 1;125(8):505-11.	Not focused on maternity care.
**32**	Wittkowski A, Zumla A, Glendenning S, Fox JR. The experience of postnatal depression in South Asian mothers living in Great Britain: a qualitative study. *J Reprod Infant Psychol*. 2011 Nov 1;29(5):480-92.	Mixed sample with only two quotes related to immigrant women.
**33**	McFadden A, Atkin K, Renfrew MJ. The impact of transnational migration on intergenerational transmission of knowledge and practice related to breast feeding. *Midwifery*. 2014 Apr 1;30(4):439-46.	Not focused on maternity care.
**34**	Datta S, Alfaham M, Davies DP, Dunstan F, Woodhead S, Evans J, Richards B. Vitamin D deficiency in pregnant women from a non-European ethnic minority population – an interventional study. *BJOG*. 2002 Aug 1;109(8):905-8.	Not clear if participants included immigrant women. Did not receive a reply from the authors on the immigrant status of the sample.

**35**	McLeish J, Redshaw M. 'I didn't think we'd be dealing with stuff like this': a qualitative study of volunteer support for very disadvantaged pregnant women and new mothers. *Midwifery*. 2017 Feb 1;45:36-43.	Mixed sample with no separate findings for immigrant women.

For peer review only

File 5: Master table of included studies

Summary of included studies

Reference	Study aim	Region	Methodology	Theory or Framework	Setting	Data analysis	Sample and mode of recruitment
[109]	To establish efficacy of linkworker services (an intervention) introduced for non-English-speaking Asian women in multi-racial health districts	Not specified	Quantitative survey: 21-item questionnaire	Not specified.		Qualitative: content analysis	Questionnaire to the Heads of Midwifery Services in 30 multi-racial district health authorities. 20 responded. Sample is not immigrant women, however this is an evaluation of an intervention
[115]	To develop a reliable and valid questionnaire to evaluate satisfaction with maternity care in Sylheti-speaking Bangladeshi women.	London.	Mixed methods: two-stage psychometric study. Firstly, a Sylheti-language questionnaire regarding Bangladeshi women's experiences of maternity services was translated and culturally adapted from an English-language questionnaire using focus groups, in-depth interviews, and iterative methods. Secondly, quantitative psychometric methods were used to field test and evaluate the acceptability, reliability, and validity of this questionnaire.	Not specified.	Not specified.	Qualitative: thematical analysis. Quantitative: validity of an instrument.	Located at four hospitals providing maternity services in London, UK. Study participants included 242 women from the London Bangladeshi communities who were in the antenatal (at least 4 months pregnant) or postnatal phase (up to 6 months after delivery). The women spoke Sylheti, a language with no accepted written form. In stage one purposive samples of 40 women in the antenatal or postnatal phase participated, along with one convenience sample of six women in the antenatal phase and three consecutive samples of 60 women in the postnatal phase. In stage two, 135 women (main sample) completed the questionnaire 2 months after delivery (82% response rate), and 50 women (retest sample) from the main sample completed a second questionnaire 2 weeks later (96% response rate).

For peer review only - http://bmjopen.bmj.com/site/about/guidelines.xhtml

88	To study the maternity care experiences of Somali refugee women in an area of West London. This article focused particularly on findings relating to the language barrier, which to a large degree underpinned or at least aggravated other problems the women experienced.	West London.	Qualitative: case study. Six semi-structured interviews and two focus groups (with six participants each).	Not specified.	Not specified.	Qualitative: thematical analysis.	Snowball sampling: 12 Somali women were selected from a larger survey involving 1400 women.
89	To undertake a qualitative study of the maternity experiences of 33 asylum seekers.	London, Plymouth, Hastings, Brighton, Oxford, Manchester, and King's Lynn.	Qualitative.	Not specified.	Home or a neutral location.	Qualitative: content analysis.	Convenience and snowball sampling of recent asylum seekers. Based on semi-structured interviews carried out in seven English cities.
116	A Sure Start local programme had funded a Bangladeshi support worker to provide bilingual breastfeeding support to childbearing Bangladeshi women, many of whom were not fluent in English. This study aimed to conduct a short evaluation of the impact of this work on the uptake and duration of breastfeeding among these women.	Tower Hamlets.	Mixed methods: the survey questionnaire included some open and closed questions about the women's intention to feed; their current feeding methods; the breastfeeding support and information they received antenatally, during the hospital stay, and postnatally; overall views on the information and support received; and some demographic details. Eleven interviews were conducted by telephone in Sylheti (a dialect that has no written format), three in English and one in Urdu (using a female family member to translate). Interviews took between 15 and 30	Not specified.	Not specified (survey conducted by telephone).	Qualitative: content analysis of a questionnaire (open and closed questions).	The two midwives and the support worker had provided breastfeeding support to 194 women during a one-year period (September 2001 to August 2002). Of these, 80 women received help from the support worker alone. The majority of these 80 women were Bangladeshi. For the evaluation, 15 women were randomly selected from these 80 women.

			minutes to complete.				
**{Rowe, 2008 #183}**	To identify any social or ethnic differences in access to antenatal care and to quantify the effects of any such differences using data collected in a survey of women's experiences of antenatal screening.	England.	Quantitative: a cross-sectional survey using a postal questionnaire.	Not specified.	Not specified.	Quantitative: cross-sectional analysis.	A stratified clustered random sampling strategy was used. Hospitals in England were stratified according to ethnic mix. To ensure inclusion of an adequate number of women from black and minority ethnicity (BME) backgrounds, hospitals with ≥ 15% of women of BME origin were oversampled. Pregnant women aged ≥16 years and receiving care in 15 participating hospitals were sent a postal questionnaire at 27–31 weeks of gestation.

110	To compare the health behaviours both antenatally (smoking and alcohol consumption) and postnatally (initiation and duration of breast feeding) of mothers who have white British or Irish heritage with those of mothers from ethnic minority groups and to examine in mothers from ethnic minority groups whether indicators of acculturation (generational status, language spoken at home, and length of residency in the UK) were associated with these health behaviours.	England.	Quantitative: a prospective nationally representative cohort study.	Not specified.	Not specified.	Quantitative: cohort study.	Stratified clustered sampling framework to over-represent mothers from ethnic minority groups and disadvantaged areas produced 6478 white British or Irish mothers and 2110 mothers from ethnic minority groups. Of those from ethnic minority groups, 681 (33%) were first generation and 55 (4%) second generation.
90	To explore and synthesise the maternity care experiences of female asylum seekers and refugees.	UK.	Qualitative: multiple exploratory longitudinal case studies that used a series of interviews, photographs taken by the women, field notes, and observational methods to contextualise data obtained during 2002 and 2003.	Theory of interactions and transformational educational theory.	Hospital settings or women's homes.	Qualitative: thematical analysis.	Women were approached if the status of 'asylum seeker' or 'refugee' was written in the hospital notes taken at their booking appointment. Fourteen women were approached, but nine women declined to participate. Five women consented, but one woman was dispersed before 20 weeks gestation and therefore was not included in the study. Of the remaining four participating women, three were asylum seekers and one was a refugee. The sampling technique was not clearly reported.

For peer review only - http://bmjopen.bmj.com/site/about/guidelines.xhtml

91	To identify key features of communication across antenatal care and whether they are evaluated positively or negatively by service users.	Central London.	Qualitative: used six focus groups of 15 participants each and conducted 15 semi-structured interviews. Non-English-speaking focus groups and interviews were conducted in standard Bengali, Sylheti, or Somali.	Not specified.	Focus groups: hospitals and university meeting rooms. Semi-structured interviews: various locations to suit the needs of the women.	Qualitative: thematical analysis.	The sampling technique was not clearly reported, but they recruited 30 pregnant women from diverse social and ethnic backgrounds affiliated with one NHS Trust (i.e., hospital) in central London. Participants were recruited within this hospital, in eight community antenatal clinics situated in socially and ethnically diverse areas, via a community parenting group for Somali women, and via a Bengali Women's Health Project. Within the hospital, participants were recruited from the antenatal waiting room (which services low- and high-risk pregnancies), the ultrasound clinic, and the glucose tolerance testing clinic.
111	To determine the pregnancy outcomes of women of similar parity and ethnic background who received antenatal care ('booked') compared those who did not ('unbooked') over a period of 18 months.	North Middlesex University Hospital (NMUH), London.	Quantitative: a retrospective cohort study from September 2006 to March 2008 comparing the socio-demographics and the foetal and maternal outcomes of pregnancies of unbooked versus booked women.	Not specified.	Not specified.	Quantitative: a retrospective cohort study.	Women who received no antenatal care or who delivered within 3 days of their initial booking visit were categorised as 'unbooked'. In each case, the woman who had delivered next on the labour ward register (matched for ethnicity and parity) and who had received antenatal care prior to the second trimester served as a comparison.
117	To explore the perspectives of first- and second-generation women of Pakistani origin on maternity care and to make recommendations for culturally appropriate support and care from maternity services.	West Midlands.	Mixed methods: a retrospective Q methodology study of Pakistani women following childbirth.	Retrospective Q method study.	Not specified.	Qualitative: Q methodology.	A purposive sampling strategy was used. Postnatal first- and second-generation Pakistani women were self-identified by their responses to information leaflets disseminated at local Children's Centres across an inner city in the West Midlands.

92	To study the relationships between Somali women and their Western obstetric care providers. The attitudes, perceptions, beliefs, and experiences of both groups were explored in relation to caesarean sections, particularly to identify factors that might lead to adverse obstetric outcomes.	Greater London.	Qualitative: in-depth individual and focus group interviews.	Framework of naturalistic enquiry, emic/etic model	Not specified.	Qualitative: emic/etic analysis.	Selected 39 Somali women by snowball sampling, 36 from the community and three purposively from a hospital.
93	To address the postulates that immigrant women experience sensitive care through the use of an ethnically congruent interpreter and that such women prefer to meet health providers of the same ethnic and gender profile when in a multi-ethnic obstetrics care setting.	Greater London.	Qualitative: in-depth individual and focus group interviews. Open-ended questions were presented by an obstetrician and an anthropologist.	Framework of naturalistic enquiry.	Not specified.	Qualitative: naturalistic inquiry.	Participants were recruited throughout Greater London between 2005 and 2006. Snowball sampling was used to recruit 36 immigrant Somali women, and another three were selected by a by purposive technique for a total of 39. A purposive technique was used to select further 11 Ghanaian women who had delivered at least one child within the British healthcare system and who were living within the study area at the time of data collection.

For peer review only

118	To evaluate a pilot mental health service for asylum-seeking mothers and babies.	UK (not clear).	Mixed methods: evaluation within a participatory action research framework.	Participatory action research framework.	Not specified.	Qualitative: thematical analysis. Quantitative: the CARE-Index.	An active outreach recruitment strategy was adopted by psychologists, who embedded themselves in a drop-in community group, the Merseyside Refugee & Asylum Seekers & Asylum Seekers Pre & Postnatal Support Group. Participants were West African women who were asylum seekers or refugee and who were either pregnant or had a young baby. They originated from The Gambia, Sierra Leone, Ivory Coast, and Nigeria. All spoke English. Their ages ranged from 17 to 32 years, and all babies were under 6 months of age at the point of initial contact, with three babies not yet born. Attendance at the 21 therapeutic group sessions ranged between 4 and 12 mothers (with their babies). Seven mothers attended a significant proportion or all group sessions. An additional six mothers attended 1-4 group sessions.
94	To apply the 'three delays' framework (developed for low-income African contexts) to a high-income Western scenario to identify delay-causing influences in the pathway to optimal facility treatment.	Greater London.	Qualitative: individual and focus group interviews.	'Three delays' framework.	Not specified.	Constructivist hermeneutic naturalistic study.	Purposive and snowball sampling was used to recruit 54 immigrant women originally from sub-Saharan regions in Africa (Somalia, Ghana, Nigeria, Senegal, and Eritrea) living in London and to recruit 32 maternal providers.

112	To identify predictors of late initiation of antenatal care within an ethnically diverse cohort.	Newham, East London.	Quantitative: a cross-sectional analysis of routinely collected electronic patient records from Newham University Hospital NHS Trust (NUHT).	Not specified.	Not specified.	Quantitative: cross-sectional analysis.	All women who attended their antenatal booking appointment within NUHT between 1st January 2008 and 24th January 2011 were included in this study. The main outcome measure was late antenatal booking, defined as attendance at the antenatal booking appointment after 12 weeks (+6 days) gestation. The sample included women from Somalia, Eastern Europe, Africa, the Caribbean, and South Asia.
87	To explore BME women's experiences of contemporary maternity care in England.	All over England.	Qualitative data collected from a large cross-sectional survey using three open-ended questions that encouraged participants to articulate their experience of maternity care in their own words.	Not specified.	Not specified.	Qualitative: Thematic analysis.	A random sample of 4800 women was selected using Office for National Statistics (ONS) birth registration records. The overall response rate was 63% but was only 3% from BME groups. A total of 368 women self-identified as coming from BME groups. Of those, 219 (60%) responded with open text and 132 (60%) were born outside the UK.
95	To investigate women's experiences of dispersal in pregnancy and to explore the effects of dispersal on the health and maternity care of women asylum seekers who were dispersed during pregnancy in the light of NICE guidelines on antenatal, intrapartum, and postnatal care.	London, South of England, Midlands and East of England, North West, North East, and Wales.	Qualitative: interviews were conducted with 19 women who had been dispersed during their pregnancies and with one woman kept in an Initial Accommodation Centre under a new Home Office pregnancy and dispersal guidance issued in 2012.	Not specified.	Not specified.	Qualitative (not clear).	The sampling technique was not mentioned clearly. The women interviewed came from 14 different countries and had been dispersed or relocated to or within six regions of the UK. At the time of dispersal, 14 had been awaiting a decision on their asylum claim and six had been refused asylum.

For peer review only

113	To compare the maternal and birth outcomes of Polish and Scottish women having babies in Scotland and to describe any differences in clinical profiles and service use associated with migration from Poland.	All over Scotland.	Quantitative: a population-based epidemiological study of linked maternal country of birth, maternity, and birth outcomes. Scottish maternity and neonatal records linked to birth registrations were analysed for differences in modes of delivery and pregnancy outcomes between Polish migrants and Scots. These outcomes were also compared with Polish Health Fund and survey data.	Not specified.	Not specified.	Quantitative: statistical analysis.	The study analysed 119,698 Scottish and 3105 Polish births to primiparous women in Scotland in 2004-09 using routinely collected administrative data on maternal country of birth and birth outcome.
96	To understand the multiple influences on behaviour and hence the risks to metabolic health of South Asian mothers and their unborn children, to theorise how these influences interact and build over time, and to inform the design of culturally congruent, multi-level interventions.	London boroughs, Tower Hamlets, and Newham.	Qualitative: group story-sharing sessions and individual biographical life-narrative interviews.	Multi-level ecological models.	All but four interviews were in the participants' homes.	Qualitative: phenomenology.	The study recruited from diabetes and antenatal services in two deprived London boroughs 45 women of Bangladeshi, Indian, Sri Lankan, or Pakistani origin aged 21–45 years with histories of diabetes in pregnancy. Overall, 17 women shared their experiences of diabetes, pregnancy, and health services in group discussions, and 28 women gave individual narrative interviews (facilitated by multilingual researchers). All were audiotaped, translated, and transcribed.

For peer review only

9	To understand the nature of need in superdiverse areas and to examine the emergent challenges for effective maternity service delivery in an era of superdiversity.	West Midlands.	Mixed methods: the study used a semi-structured questionnaire and held narrative interviews of newcomer women. The findings were then triangulated with interviews of professionals who regularly worked with such women.	Not specified.	Not specified.	Qualitative: systematic thematic analysis. Quantitative: triangulation of findings.	Sampling was not described clearly. However, the study used a semi-structured questionnaire that was designed in collaboration with maternity professionals and community researchers to explore the views and maternity experiences of newcomer women. Experienced multilingual female community researchers completed 82 of these questionnaires with interviewees in a range of different languages. Narrative interviews were also held with 13 women to further explore issues. The findings were triangulated with 18 interviews of professionals who regularly worked with migrant women.
98	To explore how Somali women with FGM experienced and perceived antenatal and intrapartum care in England.	Birmingham.	Qualitative: a descriptive, exploratory study using face-to-face semi-structured interviews that were audio-recorded.	Not specified.	Private room.	Qualitative: thematical analysis.	The study used convenience and snowball sampling of ten Somali women in Birmingham who had received antenatal care in England in the past 5 years.
100	To explore differences in infant thermal care beliefs between mothers of South Asian and white British origin in Bradford, UK.	Bradford District, West Yorkshire.	Mixed methods: mothers were interviewed using a questionnaire with structured and unstructured questions.	Not specified.	The women chose the location of the interview.	Qualitative: thematical analysis.	A total of 102 mothers (51 South Asian and 51 white British) were recruited in Bradford District, West Yorkshire, UK. The inclusion criteria specified infants aged 13 months or less with a parent of South Asian or white British cultural origin who lived in the Bradford District. South Asia was defined as including the countries of Pakistan, India, Afghanistan, Sri Lanka and Nepal. Recruitment was aided by local community organisations, children's centres, and community contacts. Urdu- and Punjabi-speaking interpreters were requested and provided for 69 per cent of the first-generation South Asian mothers (n = 26) in the sample.

97	To gain an understanding of infant feeding practices among a group of UK-based refugee mothers.	Liverpool and Manchester.	Qualitative: two focus group discussions and 15 semi-structured interviews.	Not specified.	HCPs: private offices or clinics Refugee women: private rooms or discrete areas at the support venue (community centre or church hall).	Qualitative: thematical analysis.	The study purposively selected 30 refugee mothers from 19 countries who now resided in Liverpool or Manchester and were at least 6 months pregnant or had a child who had been born in the UK in the last 4 years. Of these 30, 19 were HIV-negative and 11 were HIV-positive.
119	To provide insights into possible causes of poor maternity outcomes for new migrants in the West Midlands region of the UK and to develop recommendations that could help improve maternity services for these migrants.	West Midlands.	Mixed methods: a semi-structured questionnaire and in-depth interviews.	Not specified.	Not specified.	Qualitative: systematic thematical approach. Quantitative: triangulation of the findings.	A non-probability purposive sample was generated by selecting 82 women who had moved to the UK within the past 5 years and had subsequently utilised maternity services. Of these, 13 underwent in-depth interviews as well.
99	To explore the maternity care experiences of pregnant asylum-seeking women in West Yorkshire to inform service development.	West Yorkshire.	Qualitative: interpretative approach within the tradition of hermeneutic phenomenology.	Not specified.	Not specified.	Qualitative: interpretive approach with hermeneutic phenomenology analysis.	Purposive sampling was performed through the voluntary sector and a children's centre. In addition, word-of mouth led to an element of snowball sampling. Six women were recruited.
120	To provide locally applicable data on the needs of Black and minority ethnic women in relation to their uptake of maternity and neonatal care provision by primary healthcare teams in Leeds.	Leeds.	Mixed methods: questionnaires and focus groups. Interpreters were used when necessary for data collection. A questionnaire was translated into Urdu for some women.	Not specified.	Local community centres and in the participants' homes.	Qualitative: content analysis. Quantitative: survey (not clear).	A total of 97 questionnaires were completed, of which 50 were completed through informal links at community centres, schools, and in women homes. The remaining 47 were completed whilst the researcher attended various antenatal clinics in the community.

101	To study the effectiveness of three linkworker and advocacy schemes that were designed to empower minority ethnic community users of maternity services.	Birmingham.	Qualitative: focus group discussions, semi-structured interviews, and non-directive interviews.	Not specified.	Antenatal clinics in hospitals and health centres, community group settings, and participants' homes.	Qualitative: not clear, thematical analysis?	Individual interviews were conducted with 66 Asian women who had received support from linkworker and advocacy services during their pregnancy and postnatally. Of these, 28 were from Birmingham, 13 from Leeds, and 25 from Wandsworth-London. A semi-structured interview guide was translated into five Asian languages: Hindi, Punjabi, Gujarati, Urdu and Tamil. The study also included ten focus groups made up of 60 women who had not used linkworker or advocacy services. All participants were recruited with the help of various minority ethnic women's groups and community organisations. Interpreters assisted 11 personal interviews with non-users from Vietnamese and Chinese backgrounds.
62	To determine the nature of the barriers confronting women when they used antenatal and postnatal services.	Pollokshields, Glasgow.	Qualitative: semi-structured questionnaire.	Not specified.	Not specified.	Qualitative: thematic analysis.	Twenty women were interviewed in depth by a Centre's Health Development Worker. Of these, 17 were born outside the UK.
102	To study the maternity services experiences of Muslim parents in England.	UK: not specified.	Qualitative: focus groups with Muslim mothers to explore their experiences of and views about maternity services; questionnaires with Muslim fathers; and interviews with health professionals	Not specified.	Not specified.	Qualitative: content analysis.	A mixed sample of 43 immigrants and non-immigrants were recruited via their project advisory groups. The focus groups were conducted in various locations around the UK, with two focus group discussions in a language other than English. A total of eight health professionals were interviewed: six midwives (two of whom worked for Sure Start programmes), a health visitor, and a consultant obstetrician.

For peer review only

114	To determine the current clinical practice of maternity care in England, including the service provision and organisations that underpin care, from the perspective of women needing the care; to identify the key areas of concern for women receiving maternity care in England; and to determine whether and in what ways women's experiences and perceptions of care have changed over the last 10 years.	England: not specified.	Quantitative: survey.	Not specified.	Survey: not specified.	Quantitative: cross-sectional design.	Random samples of women selected for the pilot and main studies were identified by staff at the ONS using live birth registrations for births within 2 specific weeks: 2–8 January (pilot) and 4–10 March 2006 (main study). The same method of sampling was used as had been employed in 1995 to enable direct comparisons. Random samples of 400 women for the pilot survey and 4800 women for the main survey who were aged 16 years and over and who had delivered their baby in a one week period in England were selected. The sampling was stratified on the basis of births in different geographical areas (Government Office Regions (GORs)). No subgroups were oversampled. The usable response rate was 60% for the pilot survey and 63% for the main survey. The samples included 229 women of BME born outside the UK.

For peer review only

103	To explore the perceptions of pregnant asylum seekers in relation to the provision of their maternity care while in emergency accommodation in the UK.	South East of England.	Qualitative: an exploratory approach using unstructured interviews with five healthcare professionals and semi-structured interviews with ten pregnant asylum seekers.	Not specified.	Participants' emergency accommodatio ns.	Qualitative: thematical analysis.	Purposive sampling of those providing maternity care for asylum seekers produced a sample comprising two midwives (M1 and M2), one GP (GP), one hospital consultant (C), and one nurse (N), all based in south coast health centres and hospitals. A total of 15 pregnant asylum seekers were approached to participate in the study. These women entered the UK through a south coast port over a three-month period. Their countries of origin were Algeria, Congo, Angola, Nigeria, Somalia, and Iraq, and they spoke French, Portuguese, Yoruba, Arabic, and Kurdish. Translated information letters and consent forms were distributed to pregnant asylum seekers via the Refugee Help Line, which also returned signed consent forms. This constitutes non-probability, purposive sampling.
104	To explore the meanings attributed by migrant Arab Muslim women to their experiences of childbirth in the UK. In particular, to explore migrant Arab Muslim women's experiences of maternity services in the UK; to examine the traditional childbearing beliefs and practices of Arab Muslim society; and to suggest ways to provide culturally sensitive care for this group of women.	UK: not specified.	Qualitative: an interpretive ontological-phenomenological perspective informed by the philosophical tenets of Heidegger (1927/1962).	Heideggerian hermeneutic phenomenology.	All interviews were in the participants' homes except for one, which took place in a restaurant after 10 pm.	Qualitative: thematical analysis.	Purposive sampling produced eight Arab Muslim women who had migrated to one multicultural city in the Midlands.

For peer review

105	To examine the health-seeking behaviours of Korean migrant women living in the UK.	London.	Qualitative: 21 semi-structured interviews.	Foucauldian approach.	Not clear.	Qualitative: not clear.	Women were recruited from New Malden via Korean community contacts.
121	To explore perinatal clinical indicators and experiences of postnatal care among European and Middle Eastern migrant women and to compare them with those of British women at a tertiary hospital in the North East of Scotland.	North East of Scotland.	Mixed methods. Phase 1 of the research was a secondary analysis of routine data for 15,030 consecutive deliveries at Aberdeen Maternity Hospital. Phase 2 was a retrospective study of 26 European, Middle Eastern, and British mothers in this hospital. After the women had given birth, verbal data was collected using face-to-face semi-structured interviews.	Not clear.	Phase Two: 24 interviews were conducted in the homes of participants and two interviews at the University department.	Qualitative: thematical analysis. Quantitative: Phase One was a secondary analysis of routine data for 15,030 consecutive deliveries at Aberdeen Maternity Hospital. Phase 2 was a retrospective study of women.	Phase 1: The 15,030 deliveries included all births at Aberdeen Maternity Hospital over the financial years 2004–2008 in which maternal nationalities were identified and gestation was ≥ 24 weeks. Both singleton and multiple births were included. The clinical data was harvested from the Patient Administration System and the PROTOS maternity information system. In the case of women with multiple order births during the study, all births were included. Phase 2 of the research was a retrospective study of a few of the mothers who had given birth at this hospital. Eight European and five Middle Eastern women were semi-matched with 13 British women.

view only

122	To assess the mechanisms of support available to EM (ethnic minority) communities from community and voluntary sector organisations in relation to maternal and infant nutrition (a mapping exercise); to explore the experiences of the targeted client groups in seeking and receiving such support; and to identify gaps and opportunities to enhance support mechanisms and engagement with diverse EM communities.	Glasgow, Edinburgh, Aberdeen, Stirling, Fife, Dundee, and Inverness.	Mixed methods: an online questionnaire survey of organisations working with EM communities, focus groups, and telephone interviews with EM women.	Not specified.	Not specified.	Qualitative: thematical analysis. Quantitative:	The study identified 65 community organisations that potentially provided food and health services across EM communities in Scotland. In total, 37 organisations replied to the survey. Of those organisations, 15 indicated that they are providing services in the area of maternal and infant nutrition. A further 12 indicated that despite working with EM communities, they do not provide services in maternal and infant nutrition or healthy eating in general. An additional ten organisations confirmed by telephone that they were or had been working with EM women, but were unable to undertake the survey. The majority of interviewees for the focus groups and interviews were selected in response to a request sent by Black and Ethnic Minorities Infrastructure in Scotland (BEMIS) to community organisations. Snowball sampling was used to provide further contacts. In total, four focus groups were conducted with Polish, Roma, Czech, and African mothers. In addition, six telephone interviews were conducted with Polish mothers. We focused on Polish mothers because they were the largest new ethnic group in Scotland since 2004.

For peer review only

106	To explore the experiences of obstetric care in Scotland among women who have undergone FGM.	Glasgow and Edinburgh.	Qualitative: personal experiences of FGM and interviews.	Interpretivism paradigm and feminist perspective.	The Dignity Alert & Research Forum (DARF) office or in the participant's home.	Qualitative: thematical analysis.	Convenience and purposive sampling resulted in a total number of seven women taking part in this study. All women were of African origin living in Scotland (three in Glasgow and four in Edinburgh). The inclusion criteria for the study were women who have undergone FGM and had experienced childbirth in Scotland. Three women were originally from Somalia, two from The Gambia, one from Ghana, and one from Sudan. Six of them were Muslims and one was Christian. All women had undergone FGM in their countries of origin. Four women had been infibulated and the remaining three could not tell if they have had FGM type 2 or 3.
107	To gain a rich understanding of migrant Pakistani Muslim women's experiences of postnatal depression within motherhood; to inform clinical practice; and to suggest ways of improving supportive services for this group.	East London.	Qualitative: interpretative phenomenology.	Interpretative phenomenological analysis (IPA) theory.	Not specified.	Qualitative: interpretative phenomenology.	Purposive sampling resulted in the recruitment of four migrant Pakistani Muslim women from London aged from 27 to 39.
41	To explore the healthcare experience of vulnerable pregnant migrant women.	London.	Mixed methods: participants were contacted by phone (using a three-way interpreter call if appropriate) and interviewed using a pro forma questionnaire designed to determine their access to antenatal care; barriers to that access; and their experiences during pregnancy, labour, and the immediate postnatal period. Further data was extracted from their records at the Doctors of the	Not specified.	Phone survey.	Qualitative: thematical analysis. Quantitative: not clear.	Pregnant women who presented to the drop-in clinic of the DOTW in London were approached between January 2013 and June 2014.

For peer review only - http://bmjopen.bmj.com/site/about/guidelines.xhtml

			World (DOTW) clinic to see how they had accessed the clinic.				
108	To explore relationships between first-generation migrant Pakistani women and midwives in the South Wales region, focusing on the factors that contribute to these relationships and the ways that these factors might affect the women's experiences of care.	South Wales.	Qualitative: a focused ethnography.	Symbolic interactionism.	Midwives: at lunch break or between clinics. Pakistani women: not clear.	Qualitative: thematical analysis.	Purposive sampling, through midwife gatekeepers, was selected for the initial recruitment of pregnant migrant Pakistani women: emails were sent to all midwives working with migrant women in South Wales. Snowballing was then used to recruit other midwives eligible for participation. Focused, non-participant observations of antenatal booking appointments took place in antenatal clinics across the local health board region over a period of 3-6 months. A total of seven midwives and 15 women were observed during these appointments, which lasted 20-60 minutes each.

For peer review only

For peer review only

File 6: Characteristics of study participants

Participants country of origins

Studies	Country of origins
Duff, L. A., Ahmed, L. B., & Lamping, D. L. (2002).	Bangladesh
Ahmed, S., Macfarlane, A., Naylor, J., & Hastings, J. (2006).	Bangladesh
Cross-Sudworth, F., Williams, A., & Herron-Marx, S. (2011).	Pakistan
Greenhalgh,T., Clinch, M., Afsar, N., Choudhury, Y., Sudra, R., Campbell-Richards, D., & Finer, S. (2015).	Bangladesh, Indian, Sri Lanka, Pakistan
de Chavez, A. C., Ball, H. L., & Ward-Platt, M. (2016).	South Asia including Pakistan, India, Afghanistan, Sri Lanka, Nepal
Hicks, C., & Hayes, L. (1991).	Asian Sub-Continent
Harper Bulman, K., & McCourt, C. (2002).	Somalia
Essen et al. (2011).	Somalia
Moxey, J. M. &Jones, L.L. (2016).	Somalia
Raine, R., Cartwright, M., Richens, Y., Mahamed, Z., & Smith, D. (2010).	Somalia and Bengal
Binder, P., Borne, Y., Johnsdotter, S., & Essen, B. (2012).	Somalia and Ghana
Cresswell, J. A., Yu, G., Hatherall, B., Morris, J., Jamal, F., Harden, A., & Renton, A. (2013).	Somalia, Eastern European, African, Caribeean, South Asia
Binder, P., Johnsdotter, S., & Essen, B. (2012).	Somalia, Ghana, Nigeria, Eritrea and Senegal
O'Shaughnessy, R., Nelki, J., Chiumento, A., Hassan, A., & Rahman, A. (2012).	Gambia, Sierre Leone, Ivory Coast and Nigeria
McLeish, J. (2005).	Black African origin, other not specified

For peer review only

Gorman, D. R., Katikireddi, S. V., Morris, C., Chalmers, J. W. T., Sim, J., Szamotulska, K., . . . Hughes, R. G. (2014).	Poland
Rowe, R. E., Magee, H., Quigley, M. A., Heron, P., Askham, J., & Brocklehurst, P. (2008).	Not specified
Hawkins, S. S., Lamb, K., Cole, T. J., & Law, C. (2008).	Not specified
Briscoe, L., & Lavender, T. (2009).	Not specified
Tucker, A., Ogutu, D., Yoong, W., Nauta, M., & Fakokunde, A. (2010).	Not specified
Jomeen, J., & Redshaw, M. (2013).	Not specified
Feldman, R. (2014).	Not specified
Phillimore, J. (2015).	Not specified
Hufton, E., & Raven, J. (2016).	Not specified
Phillimore, J. (2016).	Not specified
Lephard, E., & Haith-Cooper, M. (2016).	Not specified
**Grey literature**	
Goodwin, L. (2016).	Pakistan
Lamba, R. (2015).	Pakistan-muslim
Bawadi, H. (2009).	Arab muslims
Lee, Jeung Yeon (2010).	Korean
BEMIS SCOTLAND in partnership with Community Food and Health (Scotland). (2013).	Poland, Roma, Czech and African
Almalik, M. (2011).	Europe and Middle East
Baldeh, F. (2013).	Somalia, Gambia, Ghana, Sudan
Nabb, J. (2006).	Algeria, Congo, Angola, Nigeria, Somalia and Iraq
Leeds Family Health. (1992).	Not specified

For peer review only

Warrier, S. (1996).	Asian
Pershad, P., Tyrrell, H. (1995).	Not specified
Ali, N. (2014).	Not specified
Redshaw et al. (2006).	Not specified
Shortall, C., et al. (2015).	Not specified

Antenatal & postnatal

**PEER REVIEWED**	
**Antenatal**	
Rowe, R. E., Magee, H., Quigley, M. A., Heron, P., Askham, J., & Brocklehurst, P. (2008).	Antenatal
Raine, R., Cartwright, M., Richens, Y., Mahamed, Z., & Smith, D. (2010).	Antenatal
Binder, P., Johnsdotter, S., & Essen, B. (2012).	Antenatal
Cresswell, J. A., Yu, G., Hatherall, B., Morris, J., Jamal, F., Harden, A., & Renton, A. (2013).	Antenatal
Feldman, R. (2014).	Antenatal
Greenhalgh,T., Clinch, M., Afsar, N., Choudhury,Y., Sudra, R., Campbell-Richards, D. & Finer, S. (2015).	Antenatal
Moxey, J. M. & Jones, L.L. (2016).	Antenatal
**Ante, intrapartum & postnatal**	
Cross-Sudworth, F., Williams, A., & Herron-Marx, S. (2011).	Ante, intrapartum & postnatal
Duff, L. A., Ahmed, L. B., & Lamping, D. L. (2002).	Ante, intrapartum & postnatal
McLeish, J. (2005).	Ante, intrapartum & postnatal
Lephard, E., & Haith-Cooper, M. (2016).	Ante, intrapartum & postnatal

For peer review only

Harper Bulman, K., & McCourt, C. (2002).	Ante, intrapartum & postnatal
Briscoe, L., & Lavender, T. (2009).	Ante, intrapartum & postnatal
Phillimore, J. (2015).	Ante, intrapartum & postnatal
Phillimore, J. (2016).	Ante, intrapartum & postnatal
Essen & al. (2011).	Ante, intrapartum & postnatal
**Postnatal**	
O'Shaughnessy, R., Nelki, J., Chiumento, A., Hassan, A., & Rahman, A. (2012).	Postnatal
Gorman, D. R., Katikireddi, S. V., Morris, C., Chalmers, J. W. T., Sim, J., Szamotulska, K., . . . Hughes, R. G. (2014).	Postnatal
de Chavez, A. C., Ball, H. L., & Ward-Platt, M. (2016).	Postnatal
Ahmed, S., Macfarlane, A., Naylor, J., & Hastings, J. (2006).	Postnatal
Hufton, E., & Raven, J. (2016).	postnatal
**Intrapartum & postnatal**	
Jomeen, J., & Redshaw, M. (2013).	Intrapartum & postnatal
**Antenatal & postnatal**	
Tucker, A., Ogutu, D., Yoong, W., Nauta, M., & Fakokunde, A. (2010).	Antenatal & postnatal
**Not clear**	
Hicks, C., & Hayes, L. (1991).	Not clear

For peer review only

**GREY LITERATURE**	
**Antenatal**	
Leeds Family Health. (1992).	Antenatal
Goodwin, L. (2016).	Antenatal
**Ante, intrapartum & postnatal**	
Ali, N. (2004).	Ante, intrapartum & postnatal
Bawadi, H. (2009)	Ante, intrapartum & postnatal
Lee, Jeung Yeon (2010).	Ante, intrapartum & postnatal
Baldeh, F. (2013).	Ante, intrapartum & postnatal
Shortall, C., et al (2015).	Ante, intrapartum & postnatal
BEMIS SCOTLAND in partnership with Community Food and Health (Scotland). (2013).	Ante, intrapartum & postnatal
Warrier, S. (1996)	Ante, intrapartum & postnatal
Redshaw et al. (2006).	Ante, intrapartum & postnatal
**Ante & postnatal**	
Almalik, M. (2011).	Ante & postnatal
**Postnatal**	
Lamba, R. (2015)	postnatal

Immigrant category

**PEER REVIEWED**	
**Refugees**	
Harper Bulman, K., & McCourt, C. (2002).	refugees
Binder, P., Borne, Y., Johnsdotter, S., & Essen, B. (2012).	refugees

For peer review only

Moxey, J. M. & Jones, L.L. (2016).	refugees
Hufton, E., & Raven, J. (2016).	refugees
**Asylum seekers**	
Feldman, R. (2014).	Asylum seekers
Lephard, E., & Haith-Cooper, M. (2016).	Asylum seekers
O'Shaughnessy, R., Nelki, J., Chiumento, A., Hassan, A., & Rahman, A. (2012).	Asylum seekers
McLeish, J. (2005).	Asylum seekers
**Immigrant category not clear**	
Duff, L. A., Ahmed, L. B., & Lamping, D. L. (2002).	Immigrant category not clear
Ahmed, S., Macfarlane, A., Naylor, J., & Hastings, J. (2006).	Immigrant category not clear
Rowe, R. E., Magee, H., Quigley, M. A., Heron, P., Askham, J., & Brocklehurst, P. (2008).	Immigrant category not clear
Hawkins, S. S., Lamb, K., Cole, T. J., & Law, C. (2008).	Immigrant category not clear
Tucker, A., Ogutu, D., Yoong, W., Nauta, M., & Fakokunde, A. (2010).	Immigrant category not clear
Cross-Sudworth, F., Williams, A., & Herron-Marx, S. (2011).	Immigrant category not clear
Raine, R., Cartwright, M., Richens, Y., Mahamed, Z., & Smith, D. (2010).	Immigrant category not clear
Jomeen, J., & Redshaw, M. (2013).	Immigrant category not clear
Gorman, D. R., Katikireddi, S. V., Morris, C., Chalmers, J. W. T., Sim, J., Szamotulska, K., . . . Hughes, R. G. (2014).	Immigrant category not clear
Greenhalgh,T.,Clinch, M., Afsar, N., Choudhury,Y., Sudra, R., Campbell-Richards, D., & Finer, S. (2015).	Immigrant category not clear
de Chavez, A. C., Ball, H. L., & Ward-Platt, M. (2016)	Immigrant category not clear

For peer review only

Hicks, C., & Hayes, L. (1991).	Immigrant category not clear
Binder, P., Johnsdotter, S., & Essen, B. (2012).	Immigrant category not clear
**Asylum seekers and refugees**	
Briscoe, L., & Lavender, T. (2009).	Asylum seekers and refugees
**Mixed migrant categories**	
Cresswell, J. A., Yu, G., Hatherall, B., Morris, J., Jamal, F., Harden, A., & Renton, A. (2013)	Mixed migrants categories
Phillimore, J. (2016).	Mixed migrant categories
Phillimore, J. (2015).	Mixed migrant categories
Essen, & al. (2011).	Mixed migrant categories

For peer review only

PRISMA 2009 Checklist

Section/topic	#	Checklist item	Reported on page #
**TITLE**			
Title	1	Identify the report as a systematic review, meta-analysis, or both.	1
**ABSTRACT**			
Structured summary	2	Provide a structured summary including, as applicable: background; objectives; data sources; study eligibility criteria, participants, and interventions; study appraisal and synthesis methods; results; limitations; conclusions and implications of key findings; systematic review registration number.	0
**INTRODUCTION**			
Rationale	3	Describe the rationale for the review in the context of what is already known.	2
Objectives	4	Provide an explicit statement of questions being addressed with reference to participants, interventions, comparisons, outcomes, and study design (PICOS).	4 & 6
**METHODS**			
Protocol and registration	5	Indicate if a review protocol exists, if and where it can be accessed (e.g., Web address), and, if available, provide registration information including registration number.	Yes https://bmjopen.bmj.com/content/bmjopen/7/7/e016988.full.pdf
Eligibility criteria	6	Specify study characteristics (e.g., PICOS, length of follow-up) and report characteristics (e.g., years considered, language, publication status) used as criteria for eligibility, giving rationale.	4 & 6
Information sources	7	Describe all information sources (e.g., databases with dates of coverage, contact with study authors to identify additional studies) in the search and date last searched.	Supplementary files
Search	8	Present full electronic search strategy for at least one database, including any limits used, such that it could be repeated.	Supplementary file
Study selection	9	State the process for selecting studies (i.e., screening, eligibility, included in systematic review, and, if applicable, included in the meta-analysis).	7 and Supplementary files

For peer review only

PRISMA 2009 Checklist

Data collection process	10	Describe method of data extraction from reports (e.g., piloted forms, independently, in duplicate) and any processes for obtaining and confirming data from investigators.	8
Data items	11	List and define all variables for which data were sought (e.g., PICOS, funding sources) and any assumptions and simplifications made.	6
Risk of bias in individual studies	12	Describe methods used for assessing risk of bias of individual studies (including specification of whether this was done at the study or outcome level), and how this information is to be used in any data synthesis.	9,10,11

n

Section/topic	#	Checklist item	Reported on page #
Risk of bias across studies	15	Specify any assessment of risk of bias that may affect the cumulative evidence (e.g., publication bias, selective reporting within studies).	9,10,11
Additional analyses	16	Describe methods of additional analyses (e.g., sensitivity or subgroup analyses, meta-regression), if done, indicating which were pre-specified.	11
**RESULTS**			
Study selection	17	Give numbers of studies screened, assessed for eligibility, and included in the review, with reasons for exclusions at each stage, ideally with a flow diagram.	Supplementary files and Prisma diagram
Study characteristics	18	For each study, present characteristics for which data were extracted (e.g., study size, PICOS, follow-up period) and provide the citations.	Supplementary file – Table of included studies
Risk of bias within studies	19	Present data on risk of bias of each study and, if available, any outcome level assessment (see item 12).	Supplementary files
Results of individual studies	20	For all outcomes considered (benefits or harms), present, for each study: (a) simple summary data for each intervention group (b) effect estimates and confidence intervals, ideally with a forest plot.	n/a

For peer review only

PRISMA 2009 Checklist

Synthesis of results	21	Present results of each meta-analysis done, including confidence intervals and measures of consistency.	n.a
Risk of bias across studies	22	Present results of any assessment of risk of bias across studies (see Item 15).	11
Additional analysis	23	Give results of additional analyses, if done (e.g., sensitivity or subgroup analyses, meta-regression [see Item 16]).	11
**DISCUSSION**			
Summary of evidence	24	Summarize the main findings including the strength of evidence for each main outcome; consider their relevance to key groups (e.g., healthcare providers, users, and policy makers).	14
Limitations	25	Discuss limitations at study and outcome level (e.g., risk of bias), and at review-level (e.g., incomplete retrieval of identified research, reporting bias).	19
Conclusions	26	Provide a general interpretation of the results in the context of other evidence, and implications for future research.	19
**FUNDING**			
Funding	27	Describe sources of funding for the systematic review and other support (e.g., supply of data); role of funders for the systematic review.	1

From: Moher D, Liberati A, Tetzlaff J, Altman DG, The PRISMA Group (2009). Preferred Reporting Items for Systematic Reviews and Meta-Analyses: The PRISMA Statement. PLoS Med 6(7): e1000097. doi:10.1371/journal.pmed1000097

For more information, visit: **www.prisma-statement.org**.

For peer review only

For peer review only - http://bmjopen.bmj.com/site/about/guidelines.xhtml

PRISMA 2009 Checklist

For peer review only

PRISMA 2009 Checklist

For peer review only

BMJ Open

Experience of and access to maternity care in the United Kingdom (UK) by immigrant women: a narrative synthesis systematic review

Journal:	*BMJ Open*
Manuscript ID	bmjopen-2019-029478.R2
Article Type:	Original research
Date Submitted by the Author:	18-Sep-2019
Complete List of Authors:	Higginbottom, Gina; University of Nottingham School of Health Sciences, School of Health Sciences<br>Evans, Catrin; University of Nottingham School of Health Sciences, School of Health Sciences<br>Morgan, Myfanwy; King's College London<br>Bharj , Kuldip; University of Leeds<br>Eldridge, Jeanette; University of Nottingham School of Health Sciences, Research and Learning Services,<br>Hussain, Basharat; University of Nottingham School of Health Sciences,
**Primary Subject Heading**:	Obstetrics and gynaecology
Secondary Subject Heading:	Health services research, Health policy, Communication
Keywords:	systematic review, narrative synthesis, immigrant women, maternity care, navigation and access

SCHOLARONE™
Manuscripts

Final

Experience of and access to maternity care in the United Kingdom (UK) by immigrant women: a narrative synthesis systematic review

Gina Marie Awoko Higginbottom,[1]* Catrin Evans,[1] Myfanwy Morgan,[2] Kuldip Kaur Bharj,[3] Jeanette Eldridge,[1] Basharat Hussain[1]

[1] Faculty of Medicine and Health Sciences, Queen's Medical Centre, University of Nottingham, Lenton Boulevard, Nottingham, NG7 2HA, UK

[2] Institute of Pharmaceutical Science, Faculty of Life Sciences and Medicine, King's College London, UK

[3] Division of Midwifery, University of Leeds, Leeds, UK

For peer review only

ABSTRACT [300 words]

One in four births in the UK is to foreign-born women. In 2016 the figure was 28.2%, the highest figure on record, with maternal and perinatal mortality also disproportionately higher for some immigrant women. Our objective was to examine issues of access and experience of maternity care by immigrant women based on a systematic review and narrative synthesis of empirical research.

Review methods

A research librarian designed the search strategies (retrieving literature published from 1990 to end June 2017). We retrieved 45,954 citations and used a screening tool to identify relevance. We searched for grey literature reported in databases/websites. We contacted stakeholders with expertise to identify additional research.

Results

We identified 40 studies for inclusion; 22 qualitative, 8 quantitative and 10 mixed methods. Immigrant women, particularly asylum seekers, often booked and accessed antenatal care later than the recommended first 10 weeks. Primary factors included limited English language proficiency, lack of awareness of availability of the services, lack of understanding of the purpose of antenatal appointments, immigration status, and income barriers. Maternity care experiences were both positive and negative. Women with positive perceptions described healthcare professionals as caring, confidential, and openly communicative in meeting their medical, emotional, psychological, and social needs. Those with negative views perceived that health professionals had as rude, discriminatory, and insensitive to their cultural and social needs. These women therefore avoided continuously utilising maternity care.

We found few interventions focused on improving maternity care and the effectiveness of existing interventions have not been scientifically evaluated

Conclusions

The experiences of immigrant women in accessing and using maternity care services were both positive and negative. Further education and training of health professionals in meeting the challenges of a super-diverse population may enhance quality of care and the perceptions and experiences of maternity care by immigrant women.

Word Count: 300

STRENGTHS AND LIMITATIONS OF THIS STUDY

For peer review only - http://bmjopen.bmj.com/site/about/guidelines.xhtml

Final

Immigration is an international phenomenon and this review increases understanding of how immigrant women navigate maternity services in the UK

The review systematically maps the positive and negative aspects of maternity care provision as experienced by immigrant women

The review provides strategic direction for enhancement of maternity care services

The review does not address the experiences of maternity care for second-generation women (e.g. women of black and minority origin born in the UK)

SUPPLEMENTARY FILES

- Original protocol
- Supplementary files 1-6

FUNDING STATEMENT

The NIHR HS&DR Programmed funded this project: The views expressed in this report are those of the authors and not necessarily those of the NHS, the NIHR or the Department of Health and Social Care (15/55/03).

PROSPERO 2015: CRD42015023605 Available from http://www.crd.york.ac.uk/PROSPERO/display_record.asp?ID=CRD420150

Professor Gina Higginbottom, Faculty of Medicine and Health Sciences, Queen's Medical Centre, Nottingham, NG7 2UH, UK
Tel: 0115 82 30991
Email: gawoko@hotmail.com

Declared competing interests of authors: none

Keywords: maternity, immigration, interventions, access to services, systematic review, narrative

For peer review only - http://bmjopen.bmj.com/site/about/guidelines.xhtml

Final

INTRODUCTION

The UK is in a period of superdiversity that is characterised by "an increased number of new, small and scattered, multiple-origin, transnationally connected, socio-economically differentiated and legally stratified immigrants".[1]p1024This presents challenges for the delivery and configuration of maternity services in achieving equality of provision which forms a key aim of the NHS in the UK.[2]One in four births in the UK is to foreign-born women.[3] Indeed some immigrant women (depending of country of origin) appear disproportionately in confidential inquiries into maternal and perinatal mortality,[4] perhaps indicating possible deficits in the delivery of care, access and utilisation. Our review contributes to amelioration of this situation by synthesising knowledge related to maternity care access and interventions so as to configure appropriate interventions as identified per the NHS Midwifery 2020 vision to guide professional development of healthcare professionals (HCPs).[5] Reshaping care to ensure culturally safe and congruent maternity care that will not only benefit immigrant women but also improve the health of future generations in the UK.[3,4,6]Without the delivery of culturally appropriate and culturally safe maternal care, negative event trajectories may occur that range from simple miscommunications to life-threatening incidents,[7-9] risking increased maternal and perinatal mortality. While recent reviews have focused on specific aspects of maternity care,[10,11] they have not considered a comprehensive conceptualisation of access or the current super diversity and redesign of NHS maternal services to meet the needs of immigrant women which requires integration of all these aspects.[2] We have addressed this deficit in our current review which utilises Gulliford et theory of access to care.[12]

Considering the global context, some commonality exists between high income nations in the maternity care experiences of immigrant women: studies in the United States,[13] Canada,[11] Australia,[14,15] Sweden,[16,17] and Germany,[8,18] all provided evidence of this in earlier international reviews led by Higginbottom[7,19], Gagnon et al.[11] However, the international comparative reviews by Gagnon focused on specific populations (South Asian and Somali) women in the UK[11] thus focusing on established immigrant groups are not the more recent super diverse patterns of migration. We have addressed this deficit in our current review.

CONCEPTUAL DEFINITIONS

There is no consensus definition in the UK regarding the definition of the term immigrant [20] with the terms immigrant and migrant which are frequently used interchangeably across different data sources and datasets whilst conveying the same meaning. **Country of birth** is used by The Annual Population Survey (APS) of workers and Labour Force Survey (LFS) as a precursor for defining a 'migrant'. This survey therefore declares a person born outside the UK is classified as a 'migrant'. Noteworthy is the fact that workers born outside the UK may become British citizens with increasing residence in the UK.

For peer review only - http://bmjopen.bmj.com/site/about/guidelines.xhtml

Final

A second source of data on migrants is applications made for obtaining a National Insurance Number. This differs from the former in that the term migrant is conferred on the basis of **nationality**. All applicants who hold nationality other than the UK are therefore considered migrants. However, the situation is dynamic in that the nationality of a person may also to change over time and in some cases individuals may acquire dual citizenship involving several nation states.

A third and significant source of data on migrants is the Office for National Statistics (ONS). ONS utilises a different strategy classification focusing on the notion of short-term international migrant and long-term international migrant. In this definition the term 'long-term' refers to holding, the intention of residing longer than a year, whereas short-term is intention of residing less than a year. The implication of this is that the ONS considers **length of stay** of a person in the UK as critical in determining migrant status which reflects the United Nations (UN) recommended classification of migrant into short and long term. Additionally, ONS utilises the UN definition of long-term international migrant. Accordingly, *"a migrant is someone who changes his or her country of usual residence for a period of at least a year, so that the country of destination effectively becomes the country of usual residence*.[20] In long-term international migration data, students and asylum seekers are also included which differs for example from the situation in the United States (US).

Immigrants and the UK National Health Service (NHS)

In respect of service provision, the NHS adheres to the mandates set by central government that determines immigrant's entitlement to free NHS care. These mandates are concerned with the immigrant status and the type of service provision.[21] Within these mandates, an asylum seeker woman may not be entitled to full maternity care because of immigration status.[22] Moreover, data collection by the NHS on this topic is not well established nor comprehensive. Currently, the NHS usually collects data on ethnicity and nationality and not on migration- related variables such as length of stay, country of origin, etc.

The National Institute of Clinical Excellence (NICE), which provides clinical guidelines for healthcare practice in the UK see NICE (2010)[23] identified recent migrant women as having complex social needs in its guidelines on *Pregnancy and complex social factors: a model for service provision for pregnant women with complex social factors* identified recent migrant women having complex social needs. Within the NICE definition, a recent migrant woman is a woman has who moved to the UK within the previous 12 months. This generic definition of the term migrant conflates migrant women of all classifications (e.g. economic migrants, asylum seekers, refugees, and those lacking English language proficiency). This suggests that there is implicit acceptance of the term migrant women in healthcare in respect of being born outside the UK, and being subject to immigration regulations, furthermore possible challenges in English language proficiency.

The operational definition of an immigrant women used in this review

For peer review only - http://bmjopen.bmj.com/site/about/guidelines.xhtml

Final

The preceding paragraphs suggests that the term 'immigrant' is defined in various ways in different countries and by different authors. However, two features are frequently referred to in these definitions, namely 'country of birth' and 'length of stay'. These factors are noted by the NICE guidelines[23] on the provision of maternity care as important in entitlement, access and ability to utilise healthcare in the UK. For example, if you are born outside the UK, it is unlikely that you are knowledgeable about the UK healthcare provision.

We adopted the following definition of an immigrant woman for the purposes of our review, and most importantly, to inform our inclusion and exclusion criteria. We defined a woman as an immigrant if she was:

- Born outside the UK, and;
- Was living in the UK for more than 12 months or had the intention to live in the UK for 12 (or more) months when first entered.

We therefore included studies on immigrant women where the population studied fulfils the these two characteristics and included population groups of foreign students, asylum seekers, recent legal refugees and immigrants, and illegal immigrants. In cases where the study populations/sample was not accurately or fully described we employed the criteria of linguistic ability, as demonstrated by the need for an interpreter as a proxy for immigrant status. Notwithstanding all of these perspectives, we acknowledge that the term 'immigrant women' is generic and refers to a highly heterogeneous group of individuals with a complex and vast array of ethnocultural groups.

Aim and rationale

We consider in this paper how accessibility and acceptability manifest, as important dimensions of access to maternity care services in terms of women's perception about availability of services and their experiences of accessing these services. We also consider whether evaluated interventions exist that challenge inequalities in maternity health care provision.

Our review employed two theoretical frameworks. These are Gulliford and colleagues theory of access and secondly the concept of cultural safety.
A theory of access to services developed by Gulliford *et al.*[12] map out four dimensions *(Figure 1)*:

1. Service availability
2. Utilisation of services and barriers to access (which includes personal, financial, and
   organisational barriers)
3. Relevance, effectiveness, and access
4. Equity and access

INSERT FIGURE 1

For peer review only - http://bmjopen.bmj.com/site/about/guidelines.xhtml

Final

We used this theoretical model in our systematic review, which was based on a synthesis project funded by the National Institute for Health Research (NIHR). Unlike most access models in the United States, this framework reflects the philosophy of the NHS in that its key principles are to provide horizontal access in terms of ensuring equality of access in the population and to achieve vertical access in terms of meeting the needs of particular groups in the population, such as minority ethnic groups. The application of these principles is influenced by availability, accessibility, and acceptability. The Gulliford model[12] has been widely used in empirical research, with the main paper cited over 730 times. This model with its emphasis on accessibility, acceptability, relevance, and effectiveness, is entirely appropriate for assessing the provision of maternity services to minority ethnic groups and was employed in this review to assist in initial theme development and to examine how this access model intersected with our evidence.

Secondly, concepts of cultural safety provided a theoretical lens for the production of recommendations. Cultural safety is a theory that aims to assist the understanding of deficits in care by considering the historical and social processes that impact power relationships within and beyond healthcare.[24]Cultural safety is achieved when programmes, instruments, procedures, methods, and actions are implemented in ways that do not harm any members of the culture or ethnocultural group who are the recipients of care. Those within the culture are best placed to know what is or is not safe for their culture, which suggests the need for increased dialogue about immigrant and partner approaches.[25-29]

Methods

We employed Popay's approach to NS[30] which consists of 4 elements (for a comprehensive explanation please see our published protocol (https://bmjopen.bmj.com/content/bmjopen/7/7/e016988.full.pdf). The unique feature of this approach is that it provides highly specified steps.

Team members have successfully employed NS previously and have vast expertise in its usage.[7]

Element 1: Developing a theory of why and for whom.

Element 2: Developing a preliminary synthesis of the findings of the included studies, following implementation of the search strategy.

Element 3: Exploring relationships in the data.

Element 4: Assessing the robustness of the synthesis.

The NS approach relies primarily on a narrative synthesis of the key findings of studies using text to summarise the findings of the synthesis that form a synthesis of the *narrative findings*

For peer review only - http://bmjopen.bmj.com/site/about/guidelines.xhtml

Final

of included papers. NS maybe used with all paradigms of research quantitative, qualitative studies, and mixed methods research studies, as the emphasis is on an interpretive synthesis of the narrative findings of research rather than on a metadata analysis.[30]

Search strategy refinement and implementation

The search strategy employed key terms used in consistently formulated text-based queries and search statements. These terms were based on subject headings, thesaurus terms, or related indexing and categorisation terms appropriate for each literature database. An example of a detailed final search strategy is given in *Supplementary File 1*. First, we searched 10 electronic databases using the aforementioned strategies see *Supplementary File 2*. Following this, we searched for appropriate grey literature in SI Web of Knowledge Conference Proceedings Citation Index (Science 1990–), ISI Web of Knowledge Conference Proceedings Citation Index (Social Science and Humanities 1990–), ProQuest Dissertations and Theses, and the Cochrane Methodology Register. We also searched using Google and Google Scholar and consulted with the study expert advisory group. In conclusion we hand searched the reference list of all included studies and relevant systematic reviews. Citations were downloaded into an ENDNOTE library and following this all duplicates removed. The bibliographic databases that we searched are listed in Table 1.

INSERT TABLE 1

We adopted the PICO approach to implement the search strategy as follows:-
P = immigrant women
I = maternity care
[C = non-immigrant women - implicit comparator emerging in the results]
O = experience of care

Our search strategy development was therefore based on:

Search concept 1 = pregnancy, childbirth [implicitly females requiring maternity care], explicit terms covering women/females requiring all types of maternity care [antenatal, perinatal, postnatal, etc.]
Search concept 2 = immigrant populations [which would not fully distinguish between “new” and “second-generation” immigrants – this would be done at the selection stage]
Search concept 3 = terms used to identify access to, use of, deficiencies in, etc., service provision [to help identify groups with poorer health outcomes or vulnerabilities]
This comprehensive search strategy generated high rates of retrieval of records, however many were not pertinent.

Screening for relevance

In many cases, the study populations/sample was not fully described. In this situation we contacted the authors for further clarification and in some cases used linguistic ability e.g. the need for an interpreter as a *proxy for immigrant status*. Our focus was on first generation immigrant women regardless of their phenotype which led to inclusion of women of *white ethnicities*, although we encountered few studies that focusing on these groups. Study

For peer review only - http://bmjopen.bmj.com/site/about/guidelines.xhtml

Final

screening was undertaken independently by two team members (GH & BH) who employed our screening tool to asses the relevance of titles and abstracts in respect of our screening tool. The entire team reviewed papers classified as ambiguous papers in order to achieve a consensus agreement and where necessary full text papers of potentially included studies were retrieved and appraised. The exclusion and inclusion criteria can be found in *Supplementary File 3*. When we retrieved full text papers which were later rejected we have documented these excluded papers and presented a rationale for exclusion. These can be found in *Supplementary File 4*

Results

Studies included in the review, findings, and evidence

Our systematic review identified 40 empirical research studies in the scientific and grey literature. The included studies embraced a broad range of ethnocultural groups and methodological genres (see table 2 for master table of included studies and *Supplementary File 5*). The search outcomes are comprehensively detailed in figure 2 the PRISMA flow chart.[31]The distribution of the studies across the themes are shown in figure 3 and publication dates in figure 4.

INSERT FIGURE 2 PRISMA FLOW CHART INSERT TABLE 2

INSERT FIGURE 3 AND 4

Data Extraction and assessment of relevance

We conducted the following foundational activities in order to extract data (discussed in detail later).

1) Textual description. A systematic textual narrative was written for each study. We used headings adapted from Popay *et al*

Setting, Participants, Aim, Sampling and Recruitment, Method, Analysis, Results.[31]

(2) Tabulation and summarisation of all studies to be included. These tables described the attributes of the studies and the results. Information was extracted from the textual description using the same headings as above and additional headings as necessary. Papers in the PDF format were imported into ATLAS.ti qualitative data analysis software (ATLAS.ti Scientific Software Development GmbH, Berlin) using the 'Attributes' option to allow the tabulation of relevant data.

Quality assessment

For peer review only - http://bmjopen.bmj.com/site/about/guidelines.xhtml

Final

In element 4, we conducted the quality appraisal (see tables 3 & 4).[32] All included studies were critically appraised by two reviewers using tools from the Center for Evidence-Based Management (CEBMa).[33]We used GRAMM[34] for the mixed-methods studies. Differences were resolved in our reflective team meetings. We also used high, medium, and low as appraisal categories (discussed in detail below) This is approach is congruent with recent publications from the Cochrane Qualitative Research Group's CERQUAL publications and was previously used by in published studies by Higginbottom and colleagues.[7,9] Studies were classified in three into domains, high, medium and low, to enable a 'macro' evaluation.

- *High* was assigned to studies that used a rigorous and robust scientific approach that largely met all CEBMa benchmarks, perhaps equal to or exceeding 7 out of 10 for qualitative studies, 9 out of 12 for cross-sectional surveys, or 5 out of 6 for mixed-methods research.

- *Medium* was assigned to studies that had some flaws but that did not seriously undermine the quality and scientific value of the research conducted, perhaps scoring 5 or 6 out of 10 for qualitative studies, 6 to 8 out of 12 for cross-sectional surveys, or 4 out of 6 for mixed-methods research.

- *Low* was assigned to studies that had serious or fatal flaws and poor scientific value and scored below the numbers of benchmarks listed above for medium-level appraisals in each type of research

INSERT TABLES 3 & 4

The past decade has witnessed a growth in approaches to assessing quality and Popay[30] recommends evaluating not only the scientific quality of studies but also the 'richness' of studies, defined as "*the extent to which study findings provide in-depth explanatory insights that are transferable to other settings*"p 230[30] 'Thick' papers create or draw upon theory to provide in-depth explanatory insights that can potentially be transferable to other contexts. By contrast, 'thin' papers provide a limited or superficial description and offer little opportunity for generalising. Each paper was assessed against the criteria as set out in Higginbottom et al. p.5[32,28] and categorised as either 'thick' or 'thin' see table 3.

Analysis and synthesis

Following construction of the preliminary themes, we produced code/narrative theme tables to demonstrate how the basic meaning units related to the theme. This involved utilising the

For peer review only - http://bmjopen.bmj.com/site/about/guidelines.xhtml

Final

codes produced in ATLAS.ti and aligning these to the manually extracted key findings (see Figure 5)We reviewed all these processes in our reflective team meetings to ensure the rigour and robustness of our analytical steps. This iterative process is similar to the process of qualitative research and involved grouping the narrative findings into meaning units and social processes as they manifested in the maternity care experiences of immigrant women. Individual team members engaged in independent theming of tabular and coded data. We subsequently merged these individual perspectives to form the final harmonised themes representing a '*meta-inference'* which is a term used in mixed methods research to describe merging of findings from the positivistic and the interpretative paradigms. Tashakorri and Teddlie p.101 [35] describe meta-inference as "*an overall conclusion, explanation of understanding developed from the integration of inferences obtained from the qualitative and quantitative strands"*.

Following construction of the preliminary themes, we produced code/narrative theme tables to demonstrate how the basic meaning units related to the theme. Utilising the codes produced in ATLAS.ti and aligning these to the manually extracted key findings **see figure 5**

During the analytical processes we interrogated the data identifying using the concept suggested by Roper and Shapira.[36] We have constructed the themes in a policy directive fashion in terms of containing implicit indications in order to provide tangible guidance for policy and practice that might be developed into relevant strategies that benefit immigrant women and the NHS.
INSERT FIGURE 5

Rigour, reflexivity and the quality of the synthesis

Reflexivity in the review process requires a self-conscious and explicit acknowledgement of the impact of the researcher on the research processes, interpretations and research products. Reflexivity therefore demands acknowledgement of inherent power dimensions, hierarchies and prevailing ideologies that might shape and determine interpretations and the consequent knowledge production and research products. Gender, sexuality, professional socialisation, ethnocultural orientation and political lenses as these impact upon social identities further coalescing to provide a specific perspective on any given phenomena. The review team members are imbued with a strong personal and professional commitment to the eradication of inequalities and allegiance to contemporary equality and diversity agendas. From a reflexive perspective, this is important given that immigration is global phenomena and the inherent vulnerability of some immigrant women.
Reflexive analysis alerts us as researchers to emergent themes and informs the formal and systematic process of analysis, with reflexivity defined as:

> *"sensitivity to the ways in which the researcher's presence in the research setting has contributed to the data collected and their own a priori assumptions have shaped the data analysis"* (Murphy *et al.* p.188)[37]

For peer review only - http://bmjopen.bmj.com/site/about/guidelines.xhtml

Final

Our collaborative decisions required constant review and reading and, in some cases, reviewing the theme allocation and evidence to reach consensus. Therefore, we believe we achieved a nuanced and comprehensive approach. Gina Higginbottom and Myfanwy Morgan have successfully employed this review genre previously and have vast expertise in its usage.[38]

Within the published NS reviews, we have not given great attention to the issue of publication bias. However we strived to eradicate any potential bias by undertaking a comprehensive and exhaustive literature review that included grey literature and follow up emails with authors seeking greater clarity and explanation of opaque issues. A number of the included research studies were identified via *ProQuest* and *E-theses* and do not appear as publications in peer reviewed scientific journals.

We also held a national stakeholder event during which we presented our preliminary findings to a wide range of health professions (obstetrician, general practitioner and midwives), academics, voluntary and community workers. Possibly this approach may be considered contentious in the respect of systematic review, as attendees had no previous knowledge of the original included papers although they held deep topic knowledge. Notwithstanding this, we found broad support for our findings and facilitated groups work activities in order to challenge our initial interpretations. These challenges resulted in the construction of *Theme 5: Discrimination, racism, stereotyping, cultural sensitivity, inaction, and cultural clash in maternity care for immigrant women.* These focused activities collectively contribute to the confidence in the review findings, providing verification and validation of the themes.

We identified 40 research studies that met our inclusion criteria, and we extracted and synthesised key findings into five themes see Table 5 for the publications informing each theme.

Methodological genres

Quantitative studies

We identified eight quantitative studies that all used a questionnaire for data collection.[39-46]These population-based studies and cohort surveys were all cross-sectional: none were longitudinal.

Mixed-methods studies

We identified ten mixed-methods studies that employed both qualitative and quantitative dimensions.[2,47-55]For example, Duff *et al.*[48] reported a two-stage psychometric study in which focus groups and interviews were used in the first stage to develop a questionnaire for

For peer review only - http://bmjopen.bmj.com/site/about/guidelines.xhtml

an ethnocultural group (Sylheti) In the second stage, quantitative methods were used to test and evaluate the acceptability, reliability, and validity of the questionnaire. Other mixed-methods designs included (a) interviewing a small sample of the participants after collecting data from a large-scale survey; (b) conducting semi-structured interviews with a small sample of participants based on quantitative data routinely collected from a large group of participants; and (c) using face-to-face, postal, and online questionnaires to collect data. One of the studies used Q methodology, which uses questionnaires with structured and unstructured questions.

Qualitative studies

Of the 40 studies included in this review, we identified 22 as qualitative research studies employing a range of qualitative methodologies and approaches. [17,56-76] However, many of these studies did not specify a qualitative methodological genre but instead employed a more generic qualitative approach and described only the data collection tools used. For example, some presented multiple longitudinal case studies of participants (asylum seekers and refugees) about their maternity care experiences that included photographs taken by the participants, field notes, and observations in addition to researcher interviews. Another example was a case study of an ethnocultural group, immigrant women of Somali origin, that used semi-structured interviews and focus groups. Some studies used focus groups and interviews conducted in the language of the population group; for example, Bengali, Sylheti, Urdu, and Arabic. Others used in-depth interviews, open-ended questions, group story-sharing sessions, and individual biographical life-narrative interviews. In contrast, a few studies specified a qualitative interpretive approach that used hermeneutic phenomenology and focused ethnography.

For peer review only

Studies focusing on specific ethnocultural groups

The chosen studies included participants from a wide range of ethnocultural groups that originated in diverse countries in different continents, including Asia (e.g., Bangladesh and Pakistan), Africa (e.g., Somalia and Ghana), and Europe (e.g., Poland). In some cases, the sample was drawn from a single ethnocultural group, such as Pakistani.[71] However, most of the studies were undertaken on mixed samples of immigrant women originating from different countries (e.g., Somalia, Bangladesh, and Eastern Europe) see *Supplementary File 6.*

Studies focusing on immigrant women without a clearly specified ethnocultural group

We identified 16 studies that used the term immigrant women generically and not clearly specify an ethnocultural group. In deciding to include these studies, we believed that legitimate proxies for immigrant status could be the specified use of an interpreter or the participants having countries of origin or birth outside the UK. Some studies reported immigrant women arriving from 14 different countries but did not specify the country of birth. These studies could still be included.

For peer review only - http://bmjopen.bmj.com/site/about/guidelines.xhtml

Final

INSERT TABLE 5

Theme 1: Access and utilisation of maternity care services by immigrant women

Late booking emerged as an important dimension in this theme with immigrant women study participants often booking and accessing antenatal care later than the recommended timeframe of during the first 10 weeks of pregnancy. This delayed utilisation was found to be multi-factorial in nature with influencing factors including the effects of limited English language proficiency, immigration status, lack of awareness of the services, lack of understanding of the purpose of the services, income barriers, the presence of female genital mutilation, factors associated with differences between the maternity care systems of their countries of origin and the UK, arrival in the UK late in the pregnancy, frequent relocations after arrival, the poor reputations of antenatal services in specific communities and perceptions of regarding antenatal care as a facet of medicalisation of childbirth. The range of factors affecting the access and utilisation of postnatal services were similar to those reported for antenatal services.

Theme 2: Maternity care relationships between immigrant women and healthcare professionals.

Our included studies identified the perception of service users in this group and their interactions and therapeutic encounters with healthcare professionals as significant in understanding access, utilisation, outcomes, and the quality of their maternity care experience.

Included studies identified both positive and negative perceptions of study participants regarding the ways healthcare professionals delivered maternity care services were both positive and negative. A number of studies illustrated positive relationships between healthcare professionals and immigrant women with the healthcare professionals described as caring, respecting confidentiality, and communicating openly in meeting their medical as well as emotional, psychological, and social needs. Conversely, some studies provided evidence of negative relationships between participants and healthcare professionals, with healthcare professionals described from the perspective of immigrant women as being rude, discriminatory, or insensitive to the cultural and social needs of the women. The end result of these negative encounters was that these women tended to avoid accessing utilising maternity care services consistently.

Theme 3: Communication challenges experienced by immigrant women in maternity care.

It is axiomatic that limited English language fluency presents verbal communication challenges between health care professional and their patients, families and carers. Moreover,

For peer review only - http://bmjopen.bmj.com/site/about/guidelines.xhtml
For peer review only

Final

this is compounded when healthcare professionals use complex medical or professional language that is difficult to comprehend. Nonverbal communication is culturally defined and challenges can occur through misunderstandings of facial expressions, gestures, or pictorial representations. Poor communications result as illustrated in our included studies in limited awareness of available services in addition to miscommunication with healthcare professionals. Study participants often expressed challenges in accessing services, failed to understand procedures and their outcomes and were constrained in their ability to articulate their health or maternity needs to health care providers and disempowered in respect of their involvement indecision making. They therefore sometimes gave consent for clinical procedures without fully comprehending the risks and benefits, and did not always understand advice on baby care. Studies also identified communication as not reciprocal with healthcare professionals often misunderstanding participants. These issues of communication were described as leading to feelings of isolation, fear and a perception of being ignored.

Theme 4: Organisation and legal entitlements and their impacts on the maternity care experiences of immigrant women.

The study participants in our included studies had mixed experiences with the maternity care services in the UK. Positive and commendable experiences included feeling safe in giving birth at hospital rather than at home, being able to register a complaint if poor healthcare was received, being close to a hospital facility, not being denied access to a maternity service, and having good experiences with postnatal care. Conversely, negative experiences included lack of continuity (e.g. not being able to see same maternity care providers each time)and being unaware of the configuration of maternity services work that limited appropriate use.Participants in our included studies found services bureaucratic and perceived within the UK maternity care model as having a propensity towards medical/obstetric intervention and lower segment caesarean section births.

The legal status of an immigrant women in the UK has a profound influence on their on their access to maternity care. Women without entitlement to free maternity care services in the UK were deterred from accessing timely antenatal care by the costs and by the confidentiality of their legal status. Moreover, some women arrived in the UK during the final phase of their pregnancies that resulted in interruptions in the care process, loss of their social networks, reduced control over their lives, increased mental stress, and increased vulnerability to domestic violence.

Positive experiences included receiving information from their midwives on the benefits of breastfeeding together with demonstrations on how to position the baby. Negative experiences included poor support from hospital staff on how to breastfeed their babies consequently these reported experiences are mixed.

Theme 5: Cultural sensitivity, inaction, and cultural clash in maternity care for immigrant women.

For peer review only - http://bmjopen.bmj.com/site/about/guidelines.xhtml

Final

Inequalities in access, navigation, utilisation and the subsequent maternity care outcomes are influenced by discrimination and cultural insensitivity in maternity care services according to the perspectives of women in several included studies. Although discrimination is often subtle and difficult to identify, direct and overt discrimination was reported in some studies.

Specifically, study participants of Muslim faiths challenged assumptions held by healthcare professionals, including those held regarding Muslim food practices and that their partners or husbands should help the women during labour. Moreover, healthcare professionals were reported in some studies to lack cultural sensitivity and cultural understanding. For example, these women did not optimally benefit from antenatal classes facilitated by a non-Muslim educator who had no knowledge of the relationships of Muslim culture to maternity. Furthermore, studies reported participant dissatisfaction of antenatal class with a gender mix, which contravened religious edicts. Studies illustrated that some women of Muslim faith also regarded their cultural and religious needs for were not met, and they felt that the staff lacked insight, knowledge and understanding of female genital mutilation (FGM).

Evidence from our included studies suggests some immigrant women perceived that the staff did not treat them with respect or attended fully to their health care needs, and they felt devalued, unsupported, and fearful while receiving maternity care. Our findings also identified instances of cultural clash and conflicting advice during pregnancy and maternity care, mostly resulting from differences between the cultural practices and medical systems of the home countries of the immigrant women and those in the UK In a few cases, however, midwives were happy to meet the cultural and religious needs of the study participants in our included studies in both antenatal and postnatal settings which is a positive finding.

We conceptualise the findings graphically in 5.

Patient and Public Involvement

The systematic review questions were developed in consultation with our project advisory group (PAG) including service users' priorities experience and preferences. This systematic review did not include empirical research therefore there were no human participants.

Discussion and conclusions

The UK is in a period of superdiversity, defined as being "distinguished by a dynamic interplay of variables among an increased number of new, small and scattered, multiple-origin, transnationally connected, socio-economically differentiated and legally stratified immigrants" p1024).[1]Responding to this level of diversity is challenging for UK maternity care health services and may require the development of new and innovative strategies.

The experiences of immigrant women in accessing, navigating and utilising maternity care services in the UK are both positive and negative. In order to enhance services it is essential that strategies are developed to overcome the negative experiences reported. The experience

For peer review only - http://bmjopen.bmj.com/site/about/guidelines.xhtml

Final

of maternity care services is multi-factorial in nature with a number of issues appearing to coalesce to determine the poorer experience reported by some immigrant women .Important factors identified by the review included a lack of language support, cultural insensitivity, discrimination, poor relationships between immigrant women and healthcare professionals, and a lack of legal entitlements and guidelines on the provision of welfare support and maternity care to immigrants.

Implications of findings and recommendations for maternity care policy, practice, and service delivery

Inequitable access appeared to be a consequence of the immigration and legal status of asylum-seeking women which has a profound impact on health care experiences and consequently health, and was also influenced by language fluency. We concluded that addressing language barriers and ensuring culturally sensitive care are essential elements of providing optimal maternal care for immigrant women. The issue of confidentiality may be compromised by having known interpreters in small communities. One solution may be the setting up a national-level website offering standard information on maternity care and the option of translation in a wide range of languages. Additionally, the identification of best language practices should be identified in order to improve the current language service model.

The knowledge, understandings and attitudes of maternity care health care providers is a critical determinant of care. Ethno-culturally based stereotypes, racism, judgmental views, and direct and indirect discrimination require eradication requires challenging discrimination and racism at all levels: individual, institutional, clinical, and societal Interventions to improve maternity care for immigrant women are scant, and formal evaluations of these interventions were largely absent. Increasing the social capital available to immigrant, health literacy, and advocacy resources may empower women to access and utilise maternity care services appropriately.

Maternity care staff require a greater level of mandated education to have better cultural awareness of needs of diverse client groups including newcomers to the UK. Our findings highlight the importance of demonstrating compassion, empathy, and warmth in their relationships with these women to reinforce positive attitudes among immigrant women.

It is contingent on maternity care providers to value diversity among service users and to offer individualised and culturally congruent care. One way to achieve this goal would be through birth plans that can be jointly agreed and discussed in advance by the maternity care staff and recently arrived newcomers and immigrant women. Maternity care staff should seek to empower immigrant women by providing comprehensible information and better education concerning the configuration of the maternity system in the UK, conveying accurate information about care delivery. Central to these suggestions may be to enable volunteer and third-sector organisations to work as links between the statutory maternity

For peer review only - http://bmjopen.bmj.com/site/about/guidelines.xhtml

Final

services and immigrant women. We found evidence (though not scientifically evaluated) of such links in our national networking event.

Representatives of immigration control agencies may feel obligated to adheres to immigrant rules and consider the maternity care needs of immigrant women's and baby's health as a secondary issue. The policy context regarding data protection and sharing information with the Home Office about the immigrant status of women was at issue as well, especially since variabilities have been seen in the policies for sharing this information. The results suggest that the legal and policy context is important in addressing the maternity care needs of immigrant women.

It would seem imperative, as reflected in current policy directives, to adopt a universal of aim of achieving optimal maternity care for all and not just for immigrant women .However, maternity care services should strive to give more information to immigrant women about their rights to care, the availability and configuration of maternity services, and how to navigate maternity care systems. The child in utero of an immigrant is a future UK citizen and optimising maternity care is a dimension of securing the future health of the nation. In a period of super diversity is incumbent upon health professional to have an awareness of immigrant women's legal rights and perhaps education on this topic should be mandated for maternity care professionals. Continuity in maternity caregivers and compulsory provision of interpreters would also help to improve the experiences of these women.

Decision makers and health care leaders should address the findings at a strategic level. A focus on diversity, equality and the needs of immigrant women could reasonably be embedded in the role and responsibility of '*Board level Maternity Champion'* and of '*Maternity Clinical Networks*'. Maternity service providers could consider the appointment of one obstetrician and one midwife jointly responsible for championing maternity care provision to immigrant women in their organisation. As these dimensions feature within the '*Bespoke Maternity Safety Improvement Plan.*[77] Key areas of action include: -

- **Focus on learning and best practice** – issues of equality and diversity should be featured in the Saving Babies' Lives care bundle for use by maternity commissioners and providers.

- **Focus on multi-professional team working** – continuous personal and professional training

- **Focus on data** – greater focus on ethnicity and immigration within the Maternity Services Dataset and other key data sets.

- **Focus on innovation** – create space for accelerated improvement and innovation at local level.

For peer review only - http://bmjopen.bmj.com/site/about/guidelines.xhtml

Final

Gaps in the evidence

Some locally developed and locally based interventions to address inequalities in access and quality in maternity care for immigrant women were described during the final feedback meeting. However there are very few interventions to address these issues in the published literature and their effectiveness has not been evaluated robustly. None of the interventions had also included economic evaluation of the intervention. Studies of the usual 6 weeks postnatal checks by a general practitioner were not identified nor studies that focused on the intrapartum period. As mentioned earlier we found few studies that focused on immigrant women with 'white ethnicities' in our review time period e.g. women of Eastern European origin.

Strengths and limitations

- We were challenged and constrained by the lack of consistency in describing immigrant population sin the published literature. There is exists a great deal of variation and no unified approach within the UK literature.

- Immigration is an international phenomenon and this review increases understanding of how immigrant women navigate maternity services in the UK

- The review systematically maps our positive and negative aspects of maternity care provision as experienced by immigrant

- The review provides strategic policy level direction for enhancement of maternity care services

- The review does not address the experiences of maternity care for second generation women (e.g. women of black and minority origin born in the UK) nor does it consider refugee and asylum seeking women as a separate group

Implications for future research

More research is required into how the term 'immigrant' is used, and the changes in its use over time that may affect immigrant women's care. At present, the term is used very broadly and simplistically, which masks its inherent heterogeneity. Furthermore, more research is required to understand how the intersections of particular characteristics – such as gender, education status, time in the UK, immigration status, wealth, and country of origin – may influence or alter the experiences of these women in their maternity. Research is also required that focuses on developing and evaluating specific interventions to improve maternity care for immigrant women.

COMPETING INTEREST
None

For peer review only - http://bmjopen.bmj.com/site/about/guidelines.xhtml

Final

AUTHOR CONTRIBUTIONS

Dr Gina Higginbottom (Professor, School of |Health Sciences) was principal investigator. Initiated the project and oversaw all stages. Led the interpretation/synthesis phases and drafted the manuscript.

Dr. Basharat Hussain (Senior Research Fellow) contributed to all stages of the review. Led the data extraction, coding, and quality appraisal and contributed to the manuscript.

Dr. Catrin Evans (Associate Professor of Nursing, Director of the Centre for Evidence Based Health Care) contributed to all stages of the review, provided expert methodological advice, acted as second reviewer for quality appraisal, and development of the synthesis. She contributed to the review of the final version of the manuscript.

Dr Myfanwy Morgan (Professor Emeritus King's College London) contributed to all stages of the review, provided expert methodological advice, acted as second reviewer for quality appraisal, and development of the synthesis. She contributed to the review of the manuscript.

Dr Kuldip Bharj (Retired Director of Midwifery, University of Leeds) contributed to all stages of the review, provided clinical and policy perspectives, contributed to formulation of the implications and recommendation in the manuscript.

Jeanette Eldridge (Information Specialist) designed the literature search strategy, advised the team on all aspects of information retrieval, undertook the main database searches, and contributed to the development of the manuscript.

Disclaimer

The views expressed in this report are those of the authors and not necessarily those of the NHS, NIHR or the Department of Health.

For peer review only

Acknowledgements

This project was funded by the UK NIHR Health Services and Delivery Research Programme (Grant No. HS&DR-15/55/03). Along with this funding, NIHR also contributed by peer reviewing the funding proposal.
We gratefully acknowledge valuable input from the members of our Project Advisory Group. Their input has been very helpful in making possible the successful completion and the high quality of this review. The following people kindly consented to be members:

- Jim Thornton, Professor of Obstetrics and Gynaecology, Faculty of Medicine and Health Sciences, University of Nottingham

For peer review only - http://bmjopen.bmj.com/site/about/guidelines.xhtml

Final

- Dr Caroline Mitchell, General Practitioner/Senior Clinical Lecturer, Clinical Academic Training Programme Lead, Academic Unit of Primary Medical Care (AUPMC), University of Sheffield
- Dr Jane Mischenko, Commissioning Lead: Children and Maternity Services, NHS Leeds
- Carol McCormack, Specialist Midwife, NUH Trust

We also thank following immigrant women for their input in the conceptualisation of this review:

- Ms Valentine Nkoyo, Director of Mojatu, Nottingham
- Kinsi Clarke, Nottingham Refugee Forum

For peer review only

Data Sharing

No additional data available

For peer review only - http://bmjopen.bmj.com/site/about/guidelines.xhtml

Final

IN TEXT TABLES

Ovid MEDLINE 1948– and MEDLINE In-Process and Other Non-Indexed Citations to daily update

- Ovid EMBASE 1980–2017 Week 11
- Ovid PsycINFO 1972–March Week 3 2017
- CINAHL Plus with Full Text/EBSCOHost to 2017
- MIDIRS on Ovid 1971 to April 2017
- Thomson Reuters Web of Science* 1900–2017
- ASSIA on ProQuest 1987–current
- HMIC on Ovid 1979–January 2017
- POPline (via http:// www.popline.org/) 1970 to the present

Thomson Reuters Web of Science 1900-2017 includes the following:

- Science Citation Index Expanded (SCI-EXPANDED) 1900–2017
- Social Sciences Citation Index (SSCI) 1956–2017
- Conference Proceedings Citation Index - Science (CPCI-S) 1990–2017
- Conference Proceedings Citation Index - Social Science and Humanities (CPCI-SSH) 1990–2017
- Book Citation Index - Science (BKCI-S) 2008–2017
- Book Citation Index - Social Science and Humanities (BKCI-SSH) 2008–2017
- Emerging Sources Citation Index (ESCI) - 2015–2017

Table 1: Data bases searched

For peer review only

For peer review only - http://bmjopen.bmj.com/site/about/guidelines.xhtml

Final

Table 2: Master table of included studies

Reference	Study aim	Region	Methodology	Theory or Framework	Setting	Data analysis	Sample and mode of recruitment
2	To provide insights into possible causes of poor maternity outcomes for new migrants in the West Midlands region of the UK and to develop recommendations that could help improve maternity services for these migrants.	West Midlands.	Mixed methods: a semi-structured questionnaire and in-depth interviews.	Not specified.	Not specified.	Qualitative: systematic thematic approach. Quantitative: triangulation of the findings.	A non-probability purposive sample was generated by selecting 82 women who had moved to the UK within the past 5 years and had subsequently utilised maternity services. Of these, 13 underwent in-depth interviews as well.
17	To apply the 'three delays' framework (developed for low-income African contexts) to a high-income Western scenario to identify delay-causing influences in the pathway to optimal facility treatment.	Greater London.	Qualitative: individual and focus group interviews.	'Three delays' framework.	Not specified.	Constructivist hermeneutic naturalistic study.	Purposive and snowball sampling was used to recruit 54 immigrant women originally from sub-Saharan regions in Africa (Somalia, Ghana, Nigeria, Senegal, and Eritrea) living in London and to recruit 32 maternal providers.
39	To identify any social or ethnic differences in access to antenatal care and to quantify the effects of any such differences using data collected in a survey of women's experiences of antenatal screening.	England.	Quantitative: a cross-sectional survey using a postal questionnaire.	Not specified.	Not specified.	Quantitative: cross-sectional analysis.	A stratified clustered random sampling strategy was used. Hospitals in England were stratified according to ethnic mix. To ensure inclusion of an adequate number of women from black and minority ethnicity (BME) backgrounds, hospitals with ≥ 15% of women of BME origin were oversampled. Pregnant women aged ≥16 years and receiving care in 15 participating hospitals were sent a postal questionnaire at 27–31 weeks of gestation.

For peer review only - http://bmjopen.bmj.com/site/about/guidelines.xhtml

Final

40	To establish efficacy of link-worker services (an intervention) introduced for non-English-speaking Asian women in multi-racial health districts	Not specified	Quantitative survey: 21-item questionnaire	Not specified.		Qualitative: content analysis	Questionnaire to the Heads of Midwifery Services in 30 multi-racial district health authorities. 20 responded. Sample is not immigrant women, however this is an evaluation of an intervention
41	To compare the health behaviours both antenatally (smoking and alcohol consumption) and postnatally (initiation and duration of breast feeding) of mothers who have white British or Irish heritage with those of mothers from ethnic minority groups and to examine in mothers from ethnic minority groups whether indicators of acculturation (generational status, language spoken at home, and length of residency in the UK) were associated with these health behaviours.	England.	Quantitative: a prospective nationally representative cohort study.	Not specified.	Not specified.	Quantitative: cohort study.	Stratified clustered sampling framework to over-represent mothers from ethnic minority groups and disadvantaged areas produced 6478 white British or Irish mothers and 2110 mothers from ethnic minority groups. Of those from ethnic minority groups, 681 (33%) were first generation and 55 (4%) second generation.

Final

42	To determine the pregnancy outcomes of women of similar parity and ethnic background who received antenatal care ('booked') compared those who did not ('unbooked') over a period of 18 months.	North Middlesex University Hospital (NMUH), London.	Quantitative: a retrospective cohort study from September 2006 to March 2008 comparing the socio-demographics and the foetal and maternal outcomes of pregnancies of unbooked versus booked women.	Not specified.	Not specified.	Quantitative: a retrospective cohort study.	Women who received no antenatal care or who delivered within 3 days of their initial booking visit were categorised as 'unbooked'. In each case, the woman who had delivered next on the labour ward register (matched for ethnicity and parity) and who had received antenatal care prior to the second trimester served as a comparison.
43	To identify predictors of late initiation of antenatal care within an ethnically diverse cohort.	Newham, East London.	Quantitative: a cross-sectional analysis of routinely collected electronic patient records from Newham University Hospital NHS Trust (NUHT).	Not specified.	Not specified.	Quantitative: cross-sectional analysis.	All women who attended their antenatal booking appointment within NUHT between 1st January 2008 and 24th January 2011 were included in this study. The main outcome measure was late antenatal booking, defined as attendance at the antenatal booking appointment after 12 weeks (+6 days) gestation. The sample included women from Somalia, Eastern Europe, Africa, the Caribbean, and South Asia.

For peer review only - http://bmjopen.bmj.com/site/about/guidelines.xhtml

Final

44	To compare the maternal and birth outcomes of Polish and Scottish women having babies in Scotland and to describe any differences in clinical profiles and service use associated with migration from Poland.	All over Scotland.	Quantitative: a population-based epidemiological study of linked maternal country of birth, maternity, and birth outcomes. Scottish maternity and neonatal records linked to birth registrations were analysed for differences in modes of delivery and pregnancy outcomes between Polish migrants and Scots. These outcomes were also compared with Polish Health Fund and survey data.	Not specified.	Not specified.	Quantitative: statistical analysis.	The study analysed 119,698 Scottish and 3105 Polish births to primiparous women in Scotland in 2004-09 using routinely collected administrative data on maternal country of birth and birth outcome.
45	To determine the nature of the barriers confronting women when they used antenatal and postnatal services.	Pollokshields , Glasgow.	Qualitative: semi-structured questionnaire.	Not specified.	Not specified.	Qualitative: thematic analysis.	Twenty women were interviewed in depth by a Centre's Health Development Worker. Of these, 17 were born outside the UK.

For peer review only - http://bmjopen.bmj.com/site/about/guidelines.xhtml

Final

46	To determine the current clinical practice of maternity care in England, including the service provision and organisations that underpin care, from the perspective of women needing the care; to identify the key areas of concern for women receiving maternity care in England; and to determine whether and in what ways women's experiences and perceptions of care have changed over the last 10 years.	England: not specified.	Quantitative: survey.	Not specified.	Survey: not specified.	Quantitative: cross-sectional design.	Random samples of women selected for the pilot and main studies were identified by staff at the ONS using live birth registrations for births within 2 specific weeks: 2–8 January (pilot) and 4–10 March 2006 (main study). The same method of sampling was used as had been employed in 1995 to enable direct comparisons. Random samples of 400 women for the pilot survey and 4800 women for the main survey who were aged 16 years and over and who had delivered their baby in a one-week period in England were selected. The sampling was stratified on the basis of births in different geographical areas (Government Office Regions (GORs)). No subgroups were oversampled. The usable response rate was 60% for the pilot survey and 63% for the main survey. The samples included 229 women of BME born outside the UK.

For peer review only - http://bmjopen.bmj.com/site/about/guidelines.xhtml

Final

47	To explore the healthcare experience of vulnerable pregnant migrant women.	London.	Mixed methods: participants were contacted by phone (using a three-way interpreter call if appropriate) and interviewed using a pro forma questionnaire designed to determine their access to antenatal care; barriers to that access; and their experiences during pregnancy, labour, and the immediate postnatal period. Further data was extracted from their records at the Doctors of the World (DOTW) clinic to see how they had accessed the clinic.	Not specified.	Phone survey.	Qualitative: thematic analysis. Quantitative: not clear.	Pregnant women who presented to the drop-in clinic of the DOTW in London were approached between January 2013 and June 2014.

For peer review only - http://bmjopen.bmj.com/site/about/guidelines.xhtml

Final

48	To develop a reliable and valid questionnaire to evaluate satisfaction with maternity care in Sylheti-speaking Bangladeshi women.	London.	Mixed methods: two-stage psychometric study. Firstly, a Sylheti-language questionnaire regarding Bangladeshi women's experiences of maternity services was translated and culturally adapted from an English-language questionnaire using focus groups, in-depth interviews, and iterative methods. Secondly, quantitative psychometric methods were used to field test and evaluate the acceptability, reliability, and validity of this questionnaire.	Not specified.	Not specified.	Qualitative: thematic analysis. Quantitative: validity of an instrument.	Located at four hospitals providing maternity services in London, UK. Study participants included 242 women from the London Bangladeshi communities who were in the antenatal (at least 4 months pregnant) or postnatal phase (up to 6 months after delivery). The women spoke Sylheti, a language with no accepted written form. In stage one purposive samples of 40 women in the antenatal or postnatal phase participated, along with one convenience sample of six women in the antenatal phase and three consecutive samples of 60 women in the postnatal phase. In stage two, 135 women (main sample) completed the questionnaire 2 months after delivery (82% response rate), and 50 women (retest sample) from the main sample completed a second questionnaire 2 weeks later (96% response rate).

For peer review only

Final

49	A Sure Start local programme had funded a Bangladeshi support worker to provide bilingual breastfeeding support to childbearing Bangladeshi women, many of whom were not fluent in English. This study aimed to conduct a short evaluation of the impact of this work on the uptake and duration of breastfeeding among these women.	Tower Hamlets.	Mixed methods: the survey questionnaire included some open and closed questions about the women's intention to feed; their current feeding methods; the breastfeeding support and information they received antenatally, during the hospital stay, and postnatally; overall views on the information and support received; and some demographic details. Eleven interviews were conducted by telephone in Sylheti (a dialect that has no written format), three in English and one in Urdu (using a female family member to translate). Interviews took between 15 and 30 minutes to complete.	Not specified.	Not specified (survey conducted by telephone).	Qualitative: content analysis of a questionnaire (open and closed questions).	The two midwives and the support worker had provided breastfeeding support to 194 women during a one-year period (September 2001 to August 2002). Of these, 80 women received help from the support worker alone. The majority of these 80 women were Bangladeshi. For the evaluation, 15 women were randomly selected from these 80 women.

Final

50	To explore the perspectives of first- and second-generation women of Pakistani origin on maternity care and to make recommendations for culturally appropriate support and care from maternity services.	West Midlands.	Mixed methods: a retrospective Q methodology study of Pakistani women following childbirth.	Retrospective Q method study.	Not specified.	Qualitative: Q methodology.	A purposive sampling strategy was used. Postnatal first- and second-generation Pakistani women were self-identified by their responses to information leaflets disseminated at local Children's Centres across an inner city in the West Midlands.

For peer review only - http://bmjopen.bmj.com/site/about/guidelines.xhtml

Final

51	To evaluate a pilot mental health service for asylum-seeking mothers and babies.	UK (not clear).	Mixed methods: evaluation within a participatory action research framework.	Participatory action research framework.	Not specified.	Qualitative: thematic analysis. Quantitative: the CARE-Index.	An active outreach recruitment strategy was adopted by psychologists, who embedded themselves in a drop-in community group, the Merseyside Refugee & Asylum Seekers & Asylum Seekers Pre-& Postnatal Support Group. Participants were West African women who were asylum seekers or refugee and who were either pregnant or had a young baby. They originated from The Gambia, Sierra Leone, Ivory Coast, and Nigeria. All spoke English. Their ages ranged from 17 to 32 years, and all babies were under 6 months of age at the point of initial contact, with three babies not yet born. Attendance at the 21 therapeutic group sessions ranged between 4 and 12 mothers (with their babies). Seven mothers attended a significant proportion or all group sessions. An additional six mothers attended 1-4 group sessions.

For peer review only - http://bmjopen.bmj.com/site/about/guidelines.xhtml

Final

52	To provide locally applicable data on the needs of Black and minority ethnic women in relation to their uptake of maternity and neonatal care provision by primary healthcare teams in Leeds.	Leeds.	Mixed methods: questionnaires and focus groups. Interpreters were used when necessary for data collection. A questionnaire was translated into Urdu for some women.	Not specified.	Local community centres and in the participants' homes.	Qualitative: content analysis. Quantitative: survey (not clear).	A total of 97 questionnaires were completed, of which 50 were completed through informal links at community centres, schools, and in women homes. The remaining 47 were completed whilst the researcher attended various antenatal clinics in the community.

Final

53	To explore perinatal clinical indicators and experiences of postnatal care among European and Middle Eastern migrant women and to compare them with those of British women at a tertiary hospital in the North East of Scotland.	North East of Scotland.	Mixed methods. Phase 1 of the research was a secondary analysis of routine data for 15,030 consecutive deliveries at Aberdeen Maternity Hospital. Phase 2 was a retrospective study of 26 European, Middle Eastern, and British mothers in this hospital. After the women had given birth, verbal data was collected using face-to-face semi-structured interviews.	Not clear.	Phase Two: 24 interviews were conducted in the homes of participants and two interviews at the University department.	Qualitative: thematic analysis. Quantitative: Phase One was a secondary analysis of routine data for 15,030 consecutive deliveries at Aberdeen Maternity Hospital. Phase 2 was a retrospective study of women.	Phase 1: The 15,030 deliveries included all births at Aberdeen Maternity Hospital over the financial years 2004–2008 in which maternal nationalities were identified and gestation was ≥ 24 weeks. Both singleton and multiple births were included. The clinical data was harvested from the Patient Administration System and the PROTOS maternity information system. In the case of women with multiple order births during the study, all births were included. Phase 2 of the research was a retrospective study of a few of the mothers who had given birth at this hospital. Eight European and five Middle Eastern women were semi-matched with 13 British women.

For peer review only - http://bmjopen.bmj.com/site/about/guidelines.xhtml

Final

54	To assess the mechanisms of support available to EM (ethnic minority) communities from community and voluntary sector organisations in relation to maternal and infant nutrition (a mapping exercise); to explore the experiences of the targeted client groups in seeking and receiving such support; and to identify gaps and opportunities to enhance support mechanisms and engagement with diverse EM communities.	Glasgow, Edinburgh, Aberdeen, Stirling, Fife, Dundee, and Inverness.	Mixed methods: an online questionnaire survey of organisations working with EM communities, focus groups, and telephone interviews with EM women.	Not specified.	Not specified.	Qualitative: thematic analysis. Quantitative:	The study identified 65 community organisations that potentially provided food and health services across EM communities in Scotland. In total, 37 organisations replied to the survey. Of those organisations, 15 indicated that they are providing services in the area of maternal and infant nutrition. A further 12 indicated that despite working with EM communities, they do not provide services in maternal and infant nutrition or healthy eating in general. An additional ten organisations confirmed by telephone that they were or had been working with EM women, but were unable to undertake the survey. The majority of interviewees for the focus groups and interviews were selected in response to a request sent by Black and Ethnic Minorities Infrastructure in Scotland (BEMIS) to community organisations. Snowball sampling was used to provide further contacts. In total, four focus groups were conducted with Polish, Roma, Czech, and African mothers. In addition, six telephone interviews were conducted with Polish mothers. We focused on Polish mothers because they were the largest new ethnic group in Scotland since 2004.

Final

55	To understand the nature of need in super-diverse areas and to examine the emergent challenges for effective maternity service delivery in an era of superdiversity.	West Midlands.	Mixed methods: the study used a semi-structured questionnaire and held narrative interviews of newcomer women. The findings were then triangulated with interviews of professionals who regularly worked with such women.	Not specified.	Not specified.	Qualitative: systematic thematic analysis. Quantitative: triangulation of findings.	Sampling was not described clearly. However, the study used a semi-structured questionnaire that was designed in collaboration with maternity professionals and community researchers to explore the views and maternity experiences of newcomer women. Experienced multilingual female community researchers completed 82 of these questionnaires with interviewees in a range of different languages. Narrative interviews were also held with 13 women to further explore issues. The findings were triangulated with 18 interviews of professionals who regularly worked with migrant women.

For peer review only - http://bmjopen.bmj.com/site/about/guidelines.xhtml

Final

56	To study the maternity care experiences of Somali refugee women in an area of West London. This article focused particularly on findings relating to the language barrier, which to a large degree underpinned or at least aggravated other problems the women experienced.	West London.	Qualitative: case study. Six semi-structured interviews and two focus groups (with six participants each).	Not specified.	Not specified.	Qualitative: themati analysis.	Snowball sampling: 12 Somali women were selected from a larger survey involving 1400 women.
57	To undertake a qualitative study of the maternity experiences of 33 asylum seekers.	London, Plymouth, Hastings, Brighton, Oxford, Manchester, and King's Lynn.	Qualitative.	Not specified.	Home or a neutral location.	Qualitative: content analysis.	Convenience and snowball sampling of recent asylum seekers. Based on semi-structured interviews carried out in seven English cities.

For peer review only - http://bmjopen.bmj.com/site/about/guidelines.xhtml

Final

58	To explore and synthesise the maternity care experiences of female asylum seekers and refugees.	UK.	Qualitative: multiple exploratory longitudinal case studies that used a series of interviews, photographs taken by the women, field notes, and observational methods to contextualise data obtained during 2002 and 2003.	Theory of interactions and transformational educational theory.	Hospital settings or women's homes.	Qualitative: thematic analysis.	Women were approached if the status of 'asylum seeker' or 'refugee' was written in the hospital notes taken at their booking appointment. Fourteen women were approached, but nine women declined to participate. Five women consented, but one woman was dispersed before 20 weeks gestation and therefore was not included in the study. Of the remaining four participating women, three were asylum seekers and one was a refugee. The sampling technique was not clearly reported.

For peer review only

Final

59	To identify key features of communication across antenatal care and whether they are evaluated positively or negatively by service users.	Central London.	Qualitative: used six focus groups of 15 participants each and conducted 15 semi-structured interviews. Non-English-speaking focus groups and interviews were conducted in standard Bengali, Sylheti, or Somali.	Not specified.	Focus groups: hospitals and university meeting rooms. Semi-structured interviews: various locations to suit the needs of the women.	Qualitative: thematic analysis.	The sampling technique was not clearly reported, but they recruited 30 pregnant women from diverse social and ethnic backgrounds affiliated with one NHS Trust (i.e., hospital) in central London. Participants were recruited within this hospital, in eight community antenatal clinics situated in socially and ethnically diverse areas, via a community parenting group for Somali women, and via a Bengali Women's Health Project. Within the hospital, participants were recruited from the antenatal waiting room (which services low- and high-risk pregnancies), the ultrasound clinic, and the glucose tolerance testing clinic.
60	To address the postulates that immigrant women experience sensitive care through the use of an ethnically congruent interpreter and that such women prefer to meet health providers of the same ethnic and gender profile when in a multi-ethnic obstetrics care setting.	Greater London.	Qualitative: in-depth individual and focus group interviews. Open-ended questions were presented by an obstetrician and an anthropologist.	Framework of naturalistic enquiry.	Not specified.	Qualitative: naturalistic inquiry.	Participants were recruited throughout Greater London between 2005 and 2006. Snowball sampling was used to recruit 36 immigrant Somali women, and another three were selected by a by purposive technique for a total of 39. A purposive technique was used to select further 11 Ghanaian women who had delivered at least one child within the British healthcare system and who were living within the study area at the time of data collection.

For peer review only - http://bmjopen.bmj.com/site/about/guidelines.xhtml

Final

61	To study the relationships between Somali women and their Western obstetric care providers. The attitudes, perceptions, beliefs, and experiences of both groups were explored in relation to caesarean sections, particularly to identify factors that might lead to adverse obstetric outcomes.	Greater London.	Qualitative: in-depth individual and focus group interviews.	Framework of naturalistic enquiry, emic/etic model	Not specified.	Qualitative: emic/etic analysis.	Selected 39 Somali women by snowball sampling, 36 from the community and three purposively from a hospital.
62	To investigate women's experiences of dispersal in pregnancy and to explore the effects of dispersal on the health and maternity care of women asylum seekers who were dispersed during pregnancy in the light of NICE guidelines on antenatal, intrapartum, and postnatal care.	London, South of England, Midlands and East of England, North West, North East, and Wales.	Qualitative: interviews were conducted with 19 women who had been dispersed during their pregnancies and with one woman kept in an Initial Accommodation Centre under a new Home Office pregnancy and dispersal guidance issued in 2012.	Not specified.	Not specified.	Qualitative (not clear).	The sampling technique was not mentioned clearly. The women interviewed came from 14 different countries and had been dispersed or relocated to or within six regions of the UK. At the time of dispersal, 14 had been awaiting a decision on their asylum claim and six had been refused asylum.

For peer review only - http://bmjopen.bmj.com/site/about/guidelines.xhtml

Final

63	To understand the multiple influences on behaviour and hence the risks to metabolic health of South Asian mothers and their unborn children, to theorise how these influences interact and build over time, and to inform the design of culturally congruent, multi-level interventions.	London boroughs, Tower Hamlets, and Newham.	Qualitative: group story-sharing sessions and individual biographical life-narrative interviews.	Multi-level ecological models.	All but four interviews were in the participants' homes.	Qualitative: phenomenology.	The study recruited from diabetes and antenatal services in two deprived London boroughs 45 women of Bangladeshi, Indian, Sri Lankan, or Pakistani origin aged 21–45 years with histories of diabetes in pregnancy. Overall, 17 women shared their experiences of diabetes, pregnancy, and health services in group discussions, and 28 women gave individual narrative interviews (facilitated by multilingual researchers). All were audiotaped, translated, and transcribed.

For peer review only

Final

64	To gain an understanding of infant feeding practices among a group of UK-based refugee mothers.	Liverpool and Manchester.	Qualitative: two focus group discussions and 15 semi-structured interviews.	Not specified.	HCPs: private offices or clinics Refugee women: private rooms or discrete areas at the support venue (community centre or church hall).	Qualitative: thematic analysis.	The study purposively selected 30 refugee mothers from 19 countries who now resided in Liverpool or Manchester and were at least 6 months pregnant or had a child who had been born in the UK in the last 4 years. Of these 30, 19 were HIV-negative and 11 were HIV-positive.

For peer review only

Final

65	To explore how Somali women with FGM experienced and perceived antenatal and intrapartum care in England.	Birmingham.	Qualitative: a descriptive, exploratory study using face-to-face semi-structured interviews that were audio-recorded.	Not specified.	Private room.	Qualitative: thematic analysis.	The study used convenience and snowball sampling of ten Somali women in Birmingham who had received antenatal care in England in the past 5 years.
66	To explore the maternity care experiences of pregnant asylum-seeking women in West Yorkshire to inform service development.	West Yorkshire.	Qualitative: interpretative approach within the tradition of hermeneutic phenomenology.	Not specified.	Not specified.	Qualitative: interpretive approach with hermeneutic phenomenology analysis.	Purposive sampling was performed through the voluntary sector and a children's centre. In addition, word-of mouth led to an element of snowball sampling. Six women were recruited.

For peer review only - http://bmjopen.bmj.com/site/about/guidelines.xhtml

Final

67	To explore differences in infant thermal care beliefs between mothers of South Asian and white British origin in Bradford, UK.	Bradford District, West Yorkshire.	Mixed methods: mothers were interviewed using a questionnaire with structured and unstructured questions.	Not specified.	The women chose the location of the interview.	Qualitative: thematic analysis.	A total of 102 mothers (51 South Asian and 51 white British) were recruited in Bradford District, West Yorkshire, UK. The inclusion criteria specified infants aged 13 months or less with a parent of South Asian or white British cultural origin who lived in the Bradford District. South Asia was defined as including the countries of Pakistan, India, Afghanistan, Sri Lanka and Nepal. Recruitment was aided by local community organisations, children's centres, and community contacts. Urdu- and Punjabi-speaking interpreters were requested and provided for 69 per cent of the first-generation South Asian mothers (n = 26) in the sample.
68	To study the effectiveness of three linkworker and advocacy schemes that were designed to empower minority ethnic community users of maternity services.	Birmingham.	Qualitative: focus group discussions, semi-structured interviews, and non-directive interviews.	Not specified.	Antenatal clinics in hospitals and health centres, community group settings, and participants' homes.	Qualitative: not clear, thematic analysis?	Individual interviews were conducted with 66 Asian women who had received support from link-worker and advocacy services during their pregnancy and postnatally. Of these, 28 were from Birmingham, 13 from Leeds, and 25 from Wandsworth-London. A semi-structured interview guide was translated into five Asian languages: Hindi, Punjabi, Gujarati, Urdu and Tamil. The study also included ten focus groups made up of 60 women who had not used linkworker or advocacy services. All participants were recruited with the help of various minority ethnic women's groups and community organisations. Interpreters assisted 11 personal interviews with non-users from Vietnamese and Chinese backgrounds.

For peer review only - http://bmjopen.bmj.com/site/about/guidelines.xhtml

Final

69	To study the maternity services experiences of Muslim parents in England.	UK: not specified.	Qualitative: focus groups with Muslim mothers to explore their experiences of and views about maternity services; questionnaires with Muslim fathers; and interviews with health professionals	Not specified.	Not specified.	Qualitative: content analysis.	A mixed sample of 43 immigrants and non-immigrants were recruited via their project advisory groups. The focus groups were conducted in various locations around the UK, with two focus group discussions in a language other than English. A total of eight health professionals were interviewed: six midwives (two of whom worked for Sure Start programmes), a health visitor, and a consultant obstetrician.
70	To explore the perceptions of pregnant asylum seekers in relation to the provision of their maternity care while in emergency accommodation in the UK.	South East of England.	Qualitative: an exploratory approach using unstructured interviews with five healthcare professionals and semi-structured interviews with ten pregnant asylum seekers.	Not specified.	Participants' emergency accommodat ions.	Qualitative: thematic analysis.	Purposive sampling of those providing maternity care for asylum seekers produced a sample comprising two midwives (M1 and M2), one GP (GP), one hospital consultant (C), and one nurse (N), all based in south coast health centres and hospitals. A total of 15 pregnant asylum seekers were approached to participate in the study. These women entered the UK through a south coast port over a three-month period. Their countries of origin were Algeria, Congo, Angola, Nigeria, Somalia, and Iraq, and they spoke French, Portuguese, Yoruba, Arabic, and Kurdish. Translated information letters and consent forms were distributed to pregnant asylum seekers via the Refugee Help Line, which also returned signed consent forms. This constitutes non-probability, purposive sampling.

For peer review only

For peer review only - http://bmjopen.bmj.com/site/about/guidelines.xhtml

Final

71	To explore the meanings attributed by migrant Arab Muslim women to their experiences of childbirth in the UK. In particular, to explore migrant Arab Muslim women's experiences of maternity services in the UK; to examine the traditional childbearing beliefs and practices of Arab Muslim society; and to suggest ways to provide culturally sensitive care for this group of women.	UK: not specified.	Qualitative: an interpretive ontological-phenomenological perspective informed by the philosophical tenets of Heidegger (1927/1962).	Heideggerian hermeneutic phenomenology.	All interviews were in the participants' homes except for one, which took place in a restaurant after 10 pm.	Qualitative: thematic analysis.	Purposive sampling produced eight Arab Muslim women who had migrated to one multicultural city in the Midlands.
72	To examine the health-seeking behaviours of Korean migrant women living in the UK.	London.	Qualitative: 21 semi-structured interviews.	Foucauldian approach.	Not clear.	Qualitative: not clear.	Women were recruited from New Malden via Korean community contacts.

For peer review only

For peer review only - http://bmjopen.bmj.com/site/about/guidelines.xhtml

Final

73	To explore the experiences of obstetric care in Scotland among women who have undergone FGM.	Glasgow and Edinburgh.	Qualitative: personal experiences of FGM and interviews.	Interpretivism paradigm and feminist perspective.	The Dignity Alert & Research Forum (DARF) office or in the participant's home.	Qualitative: thematic analysis.	Convenience and purposive sampling resulted in a total number of seven women taking part in this study. All women were of African origin living in Scotland (three in Glasgow and four in Edinburgh). The inclusion criteria for the study were women who have undergone FGM and had experienced childbirth in Scotland. Three women were originally from Somalia, two from The Gambia, one from Ghana, and one from Sudan. Six of them were Muslims and one was Christian. All women had undergone FGM in their countries of origin. Four women had been infibulated and the remaining three could not tell if they have had FGM type 2 or 3.
74	To gain a rich understanding of migrant Pakistani Muslim women's experiences of postnatal depression within motherhood; to inform clinical practice; and to suggest ways of improving supportive services for this group.	East London.	Qualitative: interpretative phenomenology.	Interpretative phenomenologic al analysis (IPA) theory.	Not specified.	Qualitative: interpretative phenomenolog y.	Purposive sampling resulted in the recruitment of four migrant Pakistani Muslim women from London aged from 27 to 39.

For peer review only

Final

75	To explore relationships between first-generation migrant Pakistani women and midwives in the South Wales region, focusing on the factors that contribute to these relationships and the ways that these factors might affect the women's experiences of care.	South Wales.	Qualitative: a focused ethnography.	Symbolic interactionism.	Midwives: at lunch break or between clinics. Pakistani women: not clear.	Qualitative: thematic analysis.	Purposive sampling, through midwife gatekeepers, was selected for the initial recruitment of pregnant migrant Pakistani women: emails were sent to all midwives working with migrant women in South Wales. Snowballing was then used to recruit other midwives eligible for participation. Focused, non-participant observations of antenatal booking appointments took place in antenatal clinics across the local health board region over a period of 3-6 months. A total of seven midwives and 15 women were observed during these appointments, which lasted 20-60 minutes each.
76	To explore BME women's experiences of contemporary maternity care in England.	All over England.	Qualitative data collected from a large cross-sectional survey using three open-ended questions that encouraged participants to articulate their experience of maternity care in their own words.	Not specified.	Not specified.	Qualitative: Thematic analysis.	A random sample of 4800 women was selected using Office for National Statistics (ONS) birth registration records. The overall response rate was 63% but was only 3% from BME groups. A total of 368 women self-identified as coming from BME groups. Of those, 219 (60%) responded with open text and 132 (60%) were born outside the UK.

For peer review only - http://bmjopen.bmj.com/site/about/guidelines.xhtml

Final

Table 3: Thick and Thin Criteria *Higginbottom et al [32]*

Richness	Operational Definition
Thick papers	• Offer greater explanatory insights into the outcome of interest<br>• Provide a clear account of the process by which the findings were produced—including the sample, its selection and its size, with any limitations or bias noted—along with clear methods of analysis<br>• Present a developed and plausible interpretation of the analysis based on the data presented.
Thin papers	• Offer only limited insights<br>• Lack a clear account of the process by which the findings were produced<br>• Present an underdeveloped and weak interpretation of the analysis based on the data presented

For peer review only - http://bmjopen.bmj.com/site/about/guidelines.xhtml

Final

Table 4: Quality appraisal of the included studies

Manual reference no.	Quality as per the CEBMa tool	Relevance	Thick/Thin
**1**	low	high	thin
**2**	low	high	thin
**3**	low	high	thin
**4**	low	high	thick
**5**	high	high	thick
**6**	med	high	thick
**7**	low	high	thin
**8**	low	high	thin
**9**	low	high	thin
**10**	med	high	thin
**11**	med	high	thin
**12**	med	high	thin
**13**	med	low	thin

For peer review only - http://bmjopen.bmj.com/site/about/guidelines.xhtml

Final

**14**	med	med	thin
**15**	high	high	thin
**16**	high	high	thin
**17**	med	high	thin
**18**	med	med	thick
**19**	high	high	thin
**20**	med	high	thick
**21**	high/medium	high	thin
**22**	high	high	thick
**23**	med	med	thin
**24**	high	high	thick
**25**	med	high	thin
**26**	high	high	thick
**27**	low	med	thin
**28**	med	high	thin
**29**	low	high	thin
**30**	med	med	thin
**31**	high	high	thick
**32**	low	high	thick

For peer review only - http://bmjopen.bmj.com/site/about/guidelines.xhtml

Final

**33**	high	high	thick
**34**	low	high	thin
**35**	high	high	thick
**36**	low	low	thin
**37**	med	med	thin
**38**	med	high	thick
**39**	high	high	thin
**40**	high	high	thick

For peer review only

For peer review only - http://bmjopen.bmj.com/site/about/guidelines.xhtml

Final

Table 5: Publications informing the themes	1	2	3	4	5
	23	23	23	11	12
Bedew, H (2009). *Migrant Arab Muslim women's experiences of childbirth in the UK.*		X	X	X	X
Bazley Goodwin, LK (2016). *The midwife-woman relationship in a South Wales community: a focused ethnography of the experiences of midwives and migrant Pakistani women in early pregnancy.*	X	X	X		
Hicks, C., & Hayes, L. (1991). *Link-workers in antenatal care: facilitators of equal opportunities in health provision or salves for the management conscience?*	X		X	X	
Leeds Family Health (1992). *Research into the uptake of maternity services as provided by primary health care teams to women from black and minorities.*	X	X	X		
Pershad, P., & Tyrrell, H. (1995). *Access to antenatal and postnatal services for Asian women living in East Pollokshields, Glasgow.*	X	X	X	X	

For peer review only - http://bmjopen.bmj.com/site/about/guidelines.xhtml

Final

Warrier, S. (1996). *Consumer empowerment: a qualitative study of link-worker and advocacy services for non-English speaking users of maternity services.*	X	X	X	X	
Duff, L. A., Ahmed, L. B., & Lamping, D. L. (2002). *Evaluating satisfaction with maternity care in women from minority ethnic communities: development and validation of a Sylheti questionnaire.*			X		X
Harper Busman, K., & McCourt, C. (2002). *Somali refugee women's experiences of maternity care in west London: a case study.*	X	X	X	X	X
Ali, N. (2004). *Experiences of maternity services: Muslim women's perspectives.*	X	X	X	X	X
MacLeish, J. (2005). *Maternity experiences of asylum seekers in England.*	X	X	X	X	X
Ahmed, S., Macfarlane, A., Naylor, J., & Hastings, J. (2006). *Evaluating bilingual peer support for breastfeeding in a local sure start.*	X	X	X	X	
Redshaw, *et al.* (2007). *Recorded delivery: a national survey of women's experience of maternity care 2006.*		X	X	X	

For peer review only - http://bmjopen.bmj.com/site/about/guidelines.xhtml

Final

Nabb, J. (2006). *Pregnant asylum-seekers: Perceptions of maternity service provision.*	X	X		X	
Rowe, R. E., Magee, H., Quigley, M. A., Heron, P., Askham, J., & Brocklehurst, P. (2008). *Social and ethnic differences in attendance for antenatal care in England.*	X				
Hawkins, S. S., Lamb, K., Cole, T. J., & Law, C. (2008). *Influence of moving to the UK on maternal health behaviours: Prospective cohort study.*	X		X		
Briscoe, L., & Lavender, T. (2009). *Exploring maternity care for asylum seekers and refugees.*			X	X	
Raine, R., Cartwright, M., Richens, Y., Mahamed, Z., & Smith, D. 2010). *A qualitative study of women's experiences of communication in antenatal care: identifying areas for action.*	X	X	X		
Tucker, A., Ogutu, D., Yoong, W., Nauta, M., & Fakokunde, A. (2010). *The unbooked mother: a cohort study of maternal and foetal outcomes in a North London Hospital.*	X		X		
Lee, J-Y (2010). *'My body is Korean, but not my child's...': a Foucauldian approach to Korean migrant women's health-seeking behaviours in the UK.*	X	X			

For peer review only - http://bmjopen.bmj.com/site/about/guidelines.xhtml

Final

Cross-Sudworth, F., Williams, A., & Herron-Marx, S. (2011). *Maternity services in multi-cultural Britain: using Q methodology to explore the views of first- and second-generation women of Pakistani origin.*		X		X	X
Essen, *et al.* (2011). *An anthropological analysis of the perspectives of Somali women in the West and their obstetric care providers on caesarean birth.*		X			
Almalik, M. (2011). *A comparative evaulation of postnatal care for migrant and UK-born women.*	X	X	X	X	
Binder, P., Borne, Y., Johnsdotter, S., & Essen, B. (2012a). *Conceptualising the prevention of adverse obstetric outcomes among immigrants using the 'three delays' framework in a high-income context.*	X	X	X	X	
O'Shaughnessy, R., Nelki, J., Chiumento, A., Hassan, A., & Rahman, A. (2012). *Sweet Mother: evaluation of a pilot mental health service for asylum-seeking mothers and babies.*				X	
Binder, P., Johnsdotter, S., & Essen, B. (2012b). *Shared language is essential: communication in a multiethnic obstetric care setting.*		X	X		
Cresswell, J. A., Yu, G., Hatherall, B., Morris, J., Jamal, F., Harden, A., & Renton, A. (2013). *Predictors of the timing of initiation of antenatal care in an ethnically diverse urban cohort in the UK.*	X		X		

For peer review only - http://bmjopen.bmj.com/site/about/guidelines.xhtml

Final

Jomeen, J., & Redshaw, M. (2013). *Ethnic minority women's experience of maternity services in England.*		X	X		X
BEMIS Scotland (2013). *A comparative evaluation of postnatal care for migrant and UK-born women.*	X	X	X		
Baldeh, F. (2013). *Obstetric Care in Scotland: the experience of women who have undergone Female Genital Mutilation (FGM).*		X	X	X	
Feldman, R. (2014). *When maternity doesn't matter: Dispersing pregnant women seeking asylum.*	X			X	
Gorman, D. R., Katikireddi, S. V., Morris, C., Chalmers, J. W. T., Sim, J., Szamotulska, K., . . . & Hughes, R. G. (2014). *Ethnic variation in maternity care: a comparison of Polish and Scottish women delivering in Scotland 2004-2009.*	X				
Greenhalgh, T., Clinch, M., Afsar, N., Choudhury, Y., Sudra, R., Campbell-Richards, D., & Finer, S. (2015). *Socio-cultural influences on the behaviour of South Asian women with diabetes in pregnancy: Qualitative study using a multi-level theoretical approach.*	X				
Phillimore, J. (2015). *Delivering maternity services in an era of superdiversity: The challenges of novelty and newness.*	X	X	X	X	X

For peer review only - http://bmjopen.bmj.com/site/about/guidelines.xhtml

Final

Lamba, R. (2015). *A Qualitative Study Exploring Migrant Pakistani-Muslim Women's Lived Experiences and Understanding of Postnatal Depression.*		X	X		
Shortall, C., *et al.* (2015). *Experiences of Pregnant Migrant Women receiving Ante/Peri and Postnatal Care in the UK: A Doctors of the World Report on the Experiences of attendees at their London Drop-In Clinic.*	X		X	X	
Moxey, J. M. & L. L. Jones (2016). *A qualitative study exploring how Somali women exposed to female genital mutilation experience and perceive antenatal and intrapartum care in England.*	X	X	X	X	
de Chavez, A. C., Ball, H. L., & Ward-Platt, M. (2016). *Bi-ethnic infant thermal care beliefs in Bradford, UK.*			X		
Hufton, E., & Raven, J. (2016). *Exploring the infant feeding practices of immigrant women in the North West of England: A case study of asylum seekers and refugees in Liverpool and Manchester.*		X		X	
Phillimore, J. (2016). *Migrant maternity in an era of superdiversity: New migrants' access to, and experience of, antenatal care in the West Midlands, UK.*	X			X	
Lephard, E., & Hait.h-Cooper, M. (2016). *Pregnant and seeking asylum: Exploring women's experiences from booking to baby'.*	X	X	X	X	X

For peer review only - http://bmjopen.bmj.com/site/about/guidelines.xhtml

Final

References

1. Vertovec, S. (2007). Super-diversity and its implications. *Ethnic and racial studies*, 30(6), 1024-1054.
2.Phillimore, J. (2016). Migrant maternity in an era of superdiversity: New migrants' access to, and experience of, antenatal care in the West Midlands, UK. Soc Sci Med. 2016 Jan;148:152-9. doi: 10.1016/j.socscimed.2015.11.030.
3. Office of National Statistics. *Birth in England and Wales by Parents Country of Birth (2013)*. 2014. Available from https://www.ons.gov.uk/peoplepopulationandcommunity/birthsdeathsandmarriages/livebirths/bulletins/parentscountryofbirthenglandandwales/2014-08-28).
4. Manktelow B, Smith L, Seaton S, Hyman-Taylor P, Kurinczuk J, Field D, et al. *MBRRACE-UK Perinatal Mortality Surveillance Report, UK Perinatal Deaths for Births from January to December 2014*. 2016. Leicester: The Infant Mortality and Morbidity Studies, Department of Health Sciences, University of Leicester. Available from https://www.npeu.ox.ac.uk/downloads/files/mbrrace-uk/reports/MBRRACE-UK-PMS-Report-2014.pdf).
5. Department of Health. *Midwifery 2020: Delivering expectations*. 2010. Available from https://assets.publishing.service.gov.uk/government/uploads/system/uploads/attachment_data/file/216029/dh_119470.pdf).
6. Garcia R, Ali N, Papadopoulos C and Randhawa G. Specific antenatal interventions for Black, Asian and Minority Ethnic (BAME) pregnant women at high risk of poor birth outcomes in the United Kingdom: a scoping review. *BMC Pregnancy and Childbirth* 2015; 15: 226. DOI: 10.1186/s12884-015-0657-2.
7. Higginbottom G, Morgan M, Alexandre M, Chiu Y, Forgeron J, Kocay D, et al. Immigrant women's experiences of maternity-care services in Canada: a systematic review using a narrative synthesis. *Systematic Reviews* 2015; 4: 13. DOI: 10.1186/2046-4053-4-13.
8. Salway SM, Higginbottom G, Reime B, Bharj KK, Chowbey P, Foster C, et al. Contributions and challenges of cross-national comparative research in migration, ethnicity and health: insights from a preliminary study of maternal health in Germany, Canada and the UK. *BMC Public Health* 2011; 11: 514. DOI: 10.1186/1471-2458-11-514.

For peer review only - http://bmjopen.bmj.com/site/about/guidelines.xhtml

Final

9. Higginbottom GM, Safipour J, Yohani S, O'Brien B, Mumtaz Z and Paton P. An ethnographic study of communication challenges in maternity care for immigrant women in rural Alberta. *Midwifery* 2015; 31: 297-304. DOI: 10.1016/j.midw.2014.09.009.
10. Tobin CL, Di Napoli P and Beck CT. Refugee and Immigrant Women's Experience of Postpartum Depression: A Meta-Synthesis. *Journal of Transcultural Nursing* 2017: 84-100. DOI: 10.1177/1043659616686167.
11. Gagnon A ZM, Zeitlin J, Roam Collaboration. Migration to western industrialised countries and perinatal health: a systematic review. *Social Science and Medicine* 2009; 69: 934-946. DOI: 10.1016/j.socscimed.2009.06.027.
12. Gulliford M, Figueroa-Munoz J, Morgan M, Hughes D, Gibson B, Beech R, et al. What does 'access to health care' mean? *Journal of Health Services Research and Policy* 2002; 7: 186-188. DOI: 10.1258/135581902760082517.
13. Guendelman S, Buekens P, Blondel B, Kaminski M, Notzon FC and Masuy-Stroobant G. Birth outcomes of immigrant women in the United States, France, and Belgium. *Maternal and Child Health Journal* 1999; 3: 177-187. DOI: 10.1023/A:102232802.
14. Carolan M and Cassar L. Antenatal care perceptions of pregnant African women attending maternity services in Melbourne, Australia. *Midwifery* 2010; 26: 189-201. DOI: 10.1016/j.midw.2008.03.005.
15. Small R, Yelland J, Lumley J, Brown S and Liamputtong P. Immigrant women's views about care during labor and birth: an Australian study of Vietnamese, Turkish, and Filipino women. *Birth* 2002; 29: 266-277. DOI: 10.1046/j.1523-536X.2002.00201.x
16. Essén B, Johnsdotter S, Hovelius B, Gudmundsson S, Sjöberg NO, Friedman J, et al. Qualitative study of pregnancy and childbirth experiences in Somalian women resident in Sweden. *BJOG: An International Journal of Obstetrics and Gynaecology* 2000; 107: 1507-1512. DOI: 10.1111/j.1471-0528.2000.tb11676.x.
17. Binder P, Johnsdotter S and Essen B. Conceptualising the prevention of adverse obstetric outcomes among immigrants using the 'three delays' framework in a high-income context. *Social Science and Medicine* 2012; 75: 2028-2036. DOI: 10.1016/j.socscimed.2012.08.010. http://ovidsp.ovid.com/ovidweb.cgi?T=JS&PAGE=reference&D=med7&NEWS=N&AN=22940012.
18. Drewniak D, Krones T and Wild V. Do attitudes and behavior of health care professionals exacerbate health care disparities among immigrant and ethnic minority groups? An integrative literature review. *International Journal of Nursing Studies* 2017; 70: 89-98. DOI: 10.1016/j.ijnurstu.2017.02.015.
19. Mumtaz Z, O'Brien B and Higginbottom G. Navigating maternity health care: a survey of the Canadian prairie newcomer experience. *BMC Pregnancy and Childbirth* 2014; 14: 4. DOI: 10.1186/1471-2393-14-4.
20. Mullins T. Definition of 'immigrant'. In: Hussain B (ed). London: Migration Statistics Unit, Office for National Statistics, 2017.
21. The King's Fund. What do we know about the impact of immigration on the NHS? Available from https://www.kingsfund.org.uk/projects/verdict/what-do-we-know-about-impact-immigration-nhs (2015, accessed 08/08/2018).
22. Jayaweera H. *Health of migrants in the UK: What do we know?* 2011. The Migration Observatory, University of Oxford. Available from https://migrationobservatory.ox.ac.uk/resources/briefings/health-of-migrants-in-the-uk-what-do-we-know/ (accessed 08/08/2018).
23. National Institute for Health and Clinical Excellence (NICE). *Pregnancy and complex social factors: A model for service provision for pregnant women with complex social factors*. 2010. Available from https://www.nice.org.uk/guidance/cg110).

For peer review only

Final

24. Ramsden I. From facts to practice: cultural safety and nursing education in Aotearoa and Te Waipounamu. In: *Binan Goonj: bridging cultures in Aboriginal health*. 2nd ed. Sydney: Elsevier, 2009. https://www.elsevier.com/books/binan-goonj/eckermann/978-0-7295-3936-4
25. Ramsden I. *Cultural safety and nursing education in Aotearoa and Te Waipounamu*. PhD, Victoria University of Wellington New Zealand, 2002.
26. Papps E and Ramsden I. Cultural safety in nursing: The New Zealand experience. *International Journal for Quality in Health Care* 1996; 8: 491-497. DOI: 10.1093/intqhc/8.5.491.
27. Nursing Council of New Zealand. Guidelines for Cultural Safety, the Treaty of Waitangi, and Maori Health in Nursing and Midwifery Education and Practice. Available from http://www.nursingcouncil.org.nz/culturalsafety.pdf. (2002, accessed 26 February).
28. Brascoupé S and Catherine Waters BA M. Cultural safety: Exploring the applicability of the concept of cultural safety to Aboriginal health and community wellness. *International Journal of Indigenous Health* 2009; 5: 6. DOI: 10.18357/ijih52200912332.
29. Aboriginal Nurses Association of Canada. Cultural Competence and Cultural Safety in Nursing Education: A Framework for First Nations, Inuit and Métis Nursing. Available from https://www.cna-aiic.ca/~/media/cna/page-content/pdf-en/first_nations_framework_e.pdf (2009, accessed 26 February).
30. Popay J, Roberts H, Sowden A, Petticrew M, Arai L, Rodgers M, et al. *Guidance on the conduct of narrative synthesis in systematic reviews*. 2006. Available from http://citeseerx.ist.psu.edu/viewdoc/download?doi=10.1.1.178.3100&rep=rep1&type=pdf).

31. Moher D, Liberati A, Tetzlaff J, Altman DG and Group P. Preferred reporting items for systematic reviews and meta-analyses: the PRISMA statement. *PLoS Medicine* 2009; 6: e1000097. DOI: 10.1371/journal.pmed.1000097 A. *Supplementary Guidance for Inclusion of Qualitative Research in Cochrane Systematic Reviews of Interventions*. Version 1 (updated August 2011) ed.: Cochrane Collaboration Qualitative Methods Group, 2016.
32. Higginbottom GM, Morgan M, O'Mahony J, Chiu Y, Kocay D, Alexandre M, et al. Immigrant women's experiences of postpartum depression in Canada: a protocol for systematic review using a narrative synthesis. *Systematic Reviews* 2013; 2: 65. DOI: 10.1186/2046-4053-2-65.
33. Center for Evidence-Based Management. Critical Appraisal Tools & Questionnaires. Available from https://www.cebma.org/ (2017, accessed 15 February).
34. O'Cathain A. Reporting Mixed Methods Projects. In: *Mixed Methods Research for Nursing and the Health Sciences*. Blackwell, 2009, pp.135-158.

35. Tashakorri C TA. *Foundations in mixed methods research; Integrating Quantitative and Qualitative Approaches in the Social and Behavioral Sciences*. Thousand Oaks, Ca: Sage Publications, 2008.

36. Roper JM, Shapira J. Ethnography in Nursing Research. Thousand Oaks, CA: Sage Publications;
2000. https://doi.org/10.4135/9781483328294

37. Murphy E DR, Greatbatch D, Parker S, Watson P Qualitative research methods in health technology assessment: a review of the literature Health Technology Assessment. *Health Technology Assessment Reports* 1998; 2.
38.Higginbottom GM, Morgan M, O'Mahony J, Chiu Y, Kocay D, Alexandre M, et al. Immigrant women's experiences of postpartum depression in Canada: a protocol for

For peer review only - http://bmjopen.bmj.com/site/about/guidelines.xhtml

Final

systematic review using a narrative synthesis. *Systematic Reviews* 2013; 2: 65. DOI: 10.1186/2046-4053-2-65

39. Rowe RE, Magee H, Quigley MA, Heron P, Askham J and Brocklehurst P. Social and ethnic differences in attendance for antenatal care in England. *Public Health* 2008; 122: 1363-1372. Health & Mental Health Services 3370. DOI: 10.1016/j.puhe.2008.05.011. http://ovidsp.ovid.com/ovidweb.cgi?T=JS&PAGE=reference&D=psyc6&NEWS=N&AN=2009-02349-007; http://dx.doi.org/10.1016/j.puhe.2008.05.011.

40. Hicks C and Hayes L. Linkworkers in antenatal care: facilitators of equal opportunities in health provision or salves for the management conscience? *Health Services Management Research* 1991; 4: 89-93. DOI: 10.1177/095148489100400202.

41. Hawkins SS, Lamb K, Cole TJ and Law C. Influence of moving to the UK on maternal health behaviours: Prospective cohort study. *BMJ* 2008; 336: 1052-1055. DOI: 10.1136/bmj.39532.688877.25. http://ovidsp.ovid.com/ovidweb.cgi?T=JS&PAGE=reference&D=emed11&NEWS=N&AN=351692247.

42. Tucker A, Ogutu D, Yoong W, Nauta M and Fakokunde A. The unbooked mother: a cohort study of maternal and foetal outcomes in a North London Hospital. *Archives of Gynecology and Obstetrics* 2010; 281: 613-616. DOI: 10.1007/s00404-009-1152-7. http://ovidsp.ovid.com/ovidweb.cgi?T=JS&PAGE=reference&D=med6&NEWS=N&AN=19551396.

43. Cresswell JA, Yu G, Hatherall B, Morris J, Jamal F, Harden A, et al. Predictors of the timing of initiation of antenatal care in an ethnically diverse urban cohort in the UK. *BMC Pregnancy and Childbirth* 2013; 13: 103. DOI: 10.1186/1471-2393-13-103. http://ovidsp.ovid.com/ovidweb.cgi?T=JS&PAGE=reference&D=medl&NEWS=N&AN=23642084.

44. Gorman DR, Katikireddi SV, Morris C, Chalmers JWT, Sim J, Szamotulska K, et al. Ethnic variation in maternity care: a comparison of Polish and Scottish women delivering in Scotland 2004-2009. *Public Health* 2014; 128: 262-267. DOI: 10.1016/j.puhe.2013.11.008. http://ovidsp.ovid.com/ovidweb.cgi?T=JS&PAGE=reference&D=medl&NEWS=N&AN=24613449.

45. Pershad P and Tyrrell H. *Access to antenatal and postnatal services for Asian women living in East Pollokshields, Glasgow*. 1995. Save the Children. Available from).

46. Redshaw M, Rowe R, Hockley C and Brocklehurst P. *Recorded delivery: a national survey of women's experience of maternity care 2006*. 2007. Oxford: National Perinatal Epidemiology Unit (NPEU). Available from https://www.npeu.ox.ac.uk/recorded-delivery).

47. Shortall C, McMorran J, Taylor K, Traianou A, Garcia de Frutos M, Jones L, et al. *Experiences of Pregnant Migrant Women receiving Ante/Peri and Postnatal Care in the UK: A Doctors of the World Report on the Experiences of attendees at their London Drop-In Clinic*. 2015. Available from https://b.3cdn.net/droftheworld/08303864eb97b2d304_lam6brw4c.pdf).

48. Duff LA, Ahmed LB and Lamping DL. Evaluating satisfaction with maternity care in women from minority ethnic communities : development and validation of a Sylheti questionnaire. *International Journal for Quality in Health Care* 2002; 13: 215-230. DOI: 10.1093/intqhc/13.3.215.

49. Ahmed S, Macfarlane A, Naylor J and Hastings J. Evaluating bilingual peer support for breastfeeding in a local sure start. *British Journal of Midwifery* 2006; 14: 467-470. DOI: 10.12968/bjom.2006.14.8.21640. http://search.ebscohost.com/login.aspx?direct=true&db=rzh&AN=106354580&site=ehost-live.

For peer review only - http://bmjopen.bmj.com/site/about/guidelines.xhtml

Final

50. Cross-Sudworth F, Williams A and Herron-Marx S. Maternity services in multi-cultural Britain: Using Q methodology to explore the views of first- and second-generation women of Pakistani origin. *Midwifery* 2011; 27: 458-468. DOI: 10.1016/j.midw.2010.03.001. http://search.ebscohost.com/login.aspx?direct=true&db=rzh&AN=104661096&site=ehost-live.

51. O'Shaughnessy R, Nelki J, Chiumento A, Hassan A and Rahman A. Sweet Mother: evaluation of a pilot mental health service for asylum-seeking mothers and babies. *Journal of Public Mental Health* 2012; 11: 214-228. DOI: 10.1108/17465721211289392.

52. Leeds Family Health. *Research into the uptake of maternity services as provided by primary health care teams to women from black and minorities*. 1992. London: Leeds FHSA. Available from).

53. Almalik M. *A comparative evaluation of postnatal care for migrant and UK-born women*. PhD, University of Aberdeen, 2011.

54 . BEMIS Scotland and Community Food and Health (Scotland). *Strengthening food work across ethnic minority communities: A focus on maternal and infant nutrition*. 2013. Available from https://www.communityfoodandhealth.org.uk/publications/strengthening-food-work-ethnic-minority-communities-focus-maternal-infant-nutrition/).

55. Phillimore J. Delivering maternity services in an era of superdiversity: The challenges of novelty and newness. *Ethnic and Racial Studies* 2015; 38: 568-582. Health & Mental Health Services 3370. DOI: 10.1080/01419870.2015.980288 http://ovidsp.ovid.com/ovidweb.cgi?T=JS&PAGE=reference&D=psyc12&NEWS=N&AN=2015-02651-003; http://dx.doi.org/10.1080/01419870.2015.980288.

56. Harper Bulman K and McCourt C. Somali refugee women's experiences of maternity care in west London: a case study. *Critical Public Health* 2002; 12: 365-380. DOI: 10.1080/0958159021000029568. http://ovidsp.ovid.com/ovidweb.cgi?T=JS&PAGE=reference&D=mwic&NEWS=N&AN=2003020613.

57. McLeish J. Maternity experiences of asylum seekers in England. *British Journal of Midwifery* 2005; 13: 782-785. DOI: 10.12968/bjom.2005.13.12.20125. http://search.ebscohost.com/login.aspx?direct=true&db=rzh&AN=106416342&site=ehost-live.

58. Briscoe L and Lavender T. Exploring maternity care for asylum seekers and refugees. *British Journal of Midwifery* 2009; 17: 17-24. DOI: 10.12968/bjom.2009.17.1.37649. http://search.ebscohost.com/login.aspx?direct=true&db=rzh&AN=105631311&site=ehost-live.

59. Raine R, Cartwright M, Richens Y, Mahamed Z and Smith D. A qualitative study of women's experiences of communication in antenatal care: identifying areas for action. *Maternal and Child Health Journal* 2010; 14: 590-599. DOI: 10.1007/s10995-009-0489-7. http://ovidsp.ovid.com/ovidweb.cgi?T=JS&PAGE=reference&D=med6&NEWS=N&AN=19554436.

60. Essén B, Binder P and Johnsdotter S. An anthropological analysis of the perspectives of Somali women in the West and their obstetric care providers on caesarean birth. *Journal of Psychosomatic Obstetrics and Gynaecology* 2011; 32: 10-18. DOI: 10.3109/0167482X.2010.547966.

61. Binder P, Borne Y, Johnsdotter S and Essen B. Shared language is essential: communication in a multiethnic obstetric care setting. *Journal of Health Communication* 2012; 17: 1171-1186. DOI: 10.1080/10810730.2012.665421.

For peer review only - http://bmjopen.bmj.com/site/about/guidelines.xhtml

Final

http://ovidsp.ovid.com/ovidweb.cgi?T=JS&PAGE=reference&D=emed14&NEWS=N&AN=366382910.

62. Feldman R. When maternity doesn't matter: Dispersing pregnant women seeking asylum. *British Journal of Midwifery* 2014; 22: 23-28. DOI: 10.12968/bjom.2014.22.1.23. http://search.ebscohost.com/login.aspx?direct=true&db=rzh&AN=104006997&site=ehost-live.

63. Greenhalgh T, Clinch M, Afsar N, Choudhury Y, Sudra R, Campbell-Richards D, et al. Socio-cultural influences on the behaviour of South Asian women with diabetes in pregnancy: Qualitative study using a multi-level theoretical approach. *BMC Medicine* 2015; 13: 120. DOI: 10.1186/s12916-015-0360-1. http://ovidsp.ovid.com/ovidweb.cgi?T=JS&PAGE=reference&D=emed17&NEWS=N&AN=604640620.

64. Hufton E and Raven J. Exploring the infant feeding practices of immigrant women in the North West of England: A case study of asylum seekers and refugees in Liverpool and Manchester. *Maternal and Child Nutrition* 2016; 12: 299-313. DOI: 10.1111/mcn.12145. http://onlinelibrary.wiley.com/journal/10.1111/(ISSN)1740-8709 http://ovidsp.ovid.com/ovidweb.cgi?T=JS&PAGE=reference&D=emed18a&NEWS=N&AN=609162428.

65. Moxey JM and Jones LL. A qualitative study exploring how Somali women exposed to female genital mutilation experience and perceive antenatal and intrapartum care in England. *BMJ Open* 2016; 6: e009846. DOI: 10.1136/bmjopen-2015-009846.

66. Lephard E and Haith-Cooper M. Pregnant and seeking asylum: Exploring women's experiences' from booking to baby'. *British Journal of Midwifery* 2016; 24: 130-136. DOI: 10.12968/bjom.2016.24.2.130. https://www.magonlinelibrary.com/doi/10.12968/bjom.2016.24.2.130.

67. de Chavez AC, Ball HL and Ward-Platt M. Bi-ethnic infant thermal care beliefs in Bradford, UK. *International Journal of Human Rights in Healthcare* 2016; 9: 120-134. DOI: 10.1108/IJHRH-08-2015-0024. http://ovidsp.ovid.com/ovidweb.cgi?T=JS&PAGE=reference&D=psyc13&NEWS=N&AN=2016-25577-005.

68. Warrier S. *Consumer empowerment: a qualitative study of linkworker and advocacy services for non-English speaking users of maternity services*. Report no. 9780946741205, 1996. London: The Maternity Alliance. Available from).

69. Ali N. *Experiences of maternity services: Muslim women's perspectives*. 2004. London: The Maternity Alliance. Available from https://www.maternityaction.org.uk/wp-content/uploads/2013/09/muslimwomensexperiencesofmaternityservices.pdf (2017, acccessed 10 April).

70. Nabb J. Pregnant asylum-seekers: Perceptions of maternity service provision. *Evidence-Based Midwifery* 2006; 4: 89-96. http://create.canterbury.ac.uk/5890/.

71. Bawadi HA. *Migrant Arab Muslim women's experiences of childbirth in the UK*. PhD, De Montfort University, 2009.

72. Lee JY. *'My body is Korean, but not my child's...': a Foucauldian approach to Korean migrant women's health-seeking behaviours in the UK*. PhD, University of Nottingham, 2010.

73. Baldeh F. *Obstetric Care in Scotland: the experience of women who have undergone Female Genital Mutilation (FGM)*. MSc (Masters), Queen Margaret University, 2013.

74. Lamba R. *A Qualitative Study Exploring Migrant Pakistani-Muslim Women's Lived Experiences and Understanding of Postnatal Depression*. PhD, University of East London, 2015.

For peer review only - http://bmjopen.bmj.com/site/about/guidelines.xhtml

Final

75. Goodwin L. *The midwife-woman relationship in a South Wales community: a focused ethnography of the experiences of midwives and migrant Pakistani women in early pregnancy*. PhD, Cardiff University, 2016.
76. Jomeen and Redshaw M. Ethnic minority women's experience of maternity services in England. Ethnicity and Health 2013; 18: 280-296. DOI: 10.1080/13557858.2012.730608. http://ovidsp.ovid.com/ovidweb.cgi?T=JS&PAGE=reference&D=medl&NEWS=N&AN=23039872.
77 Department of Health. *Bespoke Maternity Improvement Plan* (2016) accessed 14th September 2019 https://www.england.nhs.uk/signuptosafety/wp-content/uploads/sites/16/2015/11/spotlight-on-maternity-guide.pdf

For peer review only

Figure Legend/Caption

Figure 1: Gulliford et al. Theory of access (2002)

Figure 2: Prisma Flow Chart (From 1990)

Figure 3: The total numbers of studies involved in each theme

Figure 4: The range of publication dates for the included studies (1990–2016).

Figure 5: Immigrant women’s experiences of maternity care in the UK

For peer review only - http://bmjopen.bmj.com/site/about/guidelines.xhtml

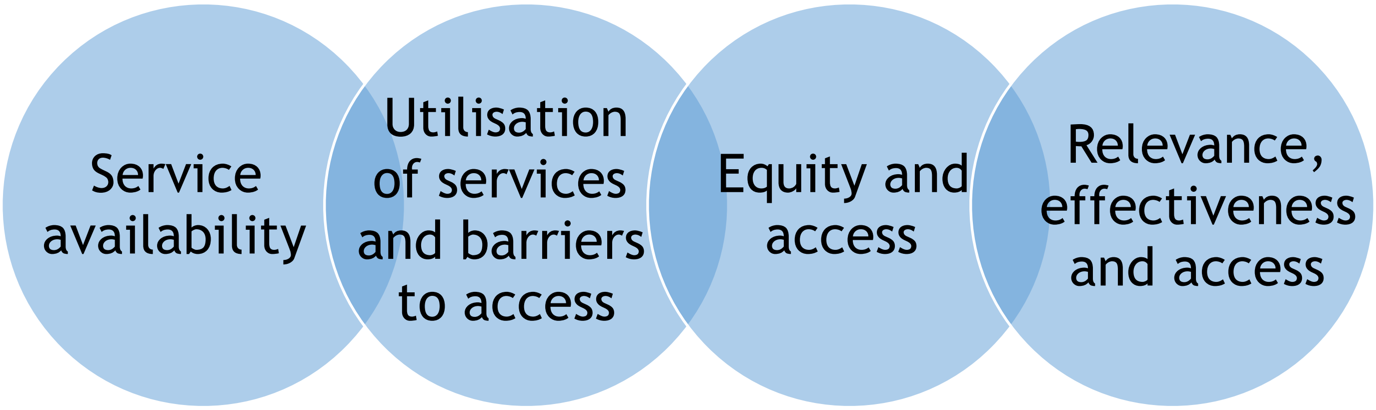

Type to enter text

Type to enter text

Figure1: Gulliford *et al.* Theory of access

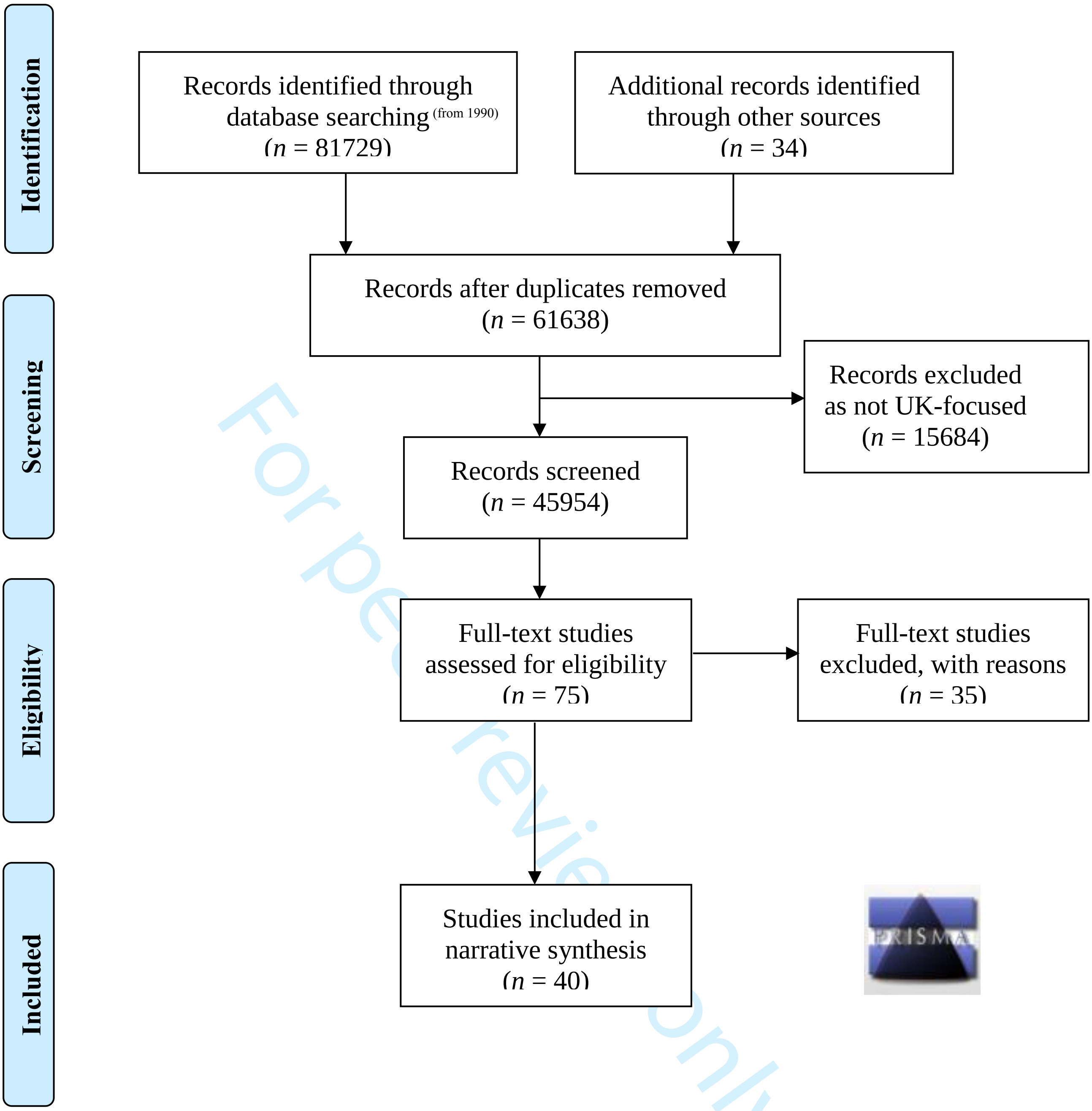

Figure 2: PRISMA flow diagram of the final selection process.

For peer review only

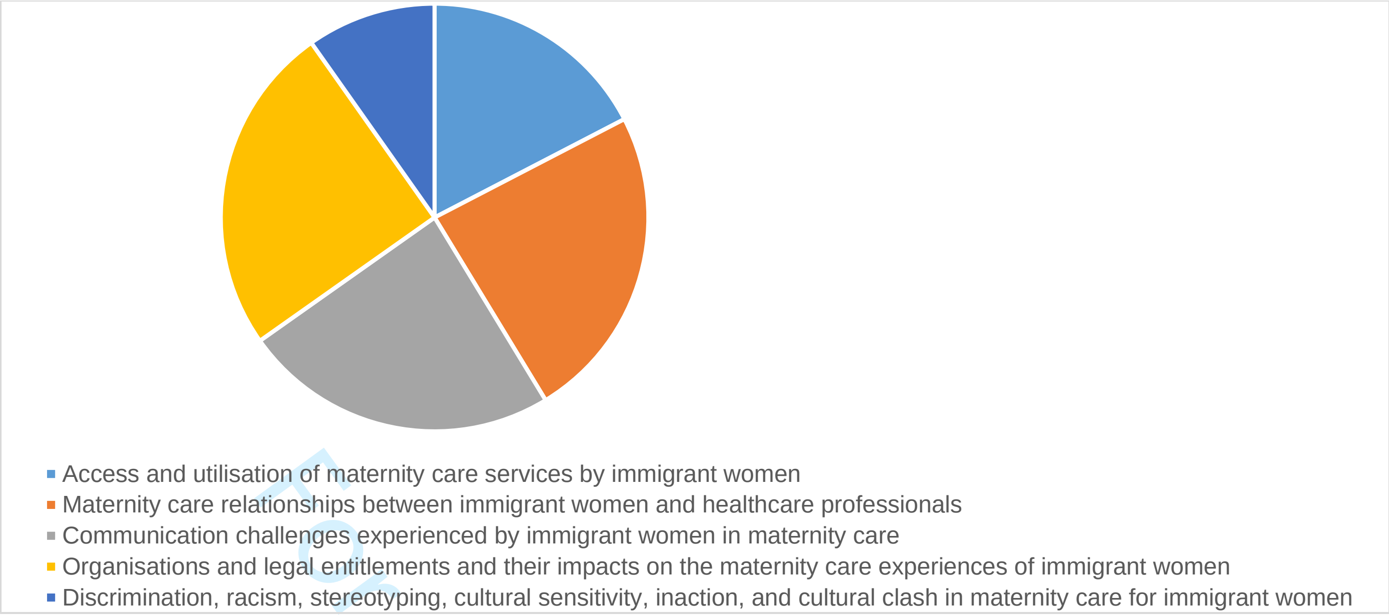

.***Figure 3:*** *The* total numbers of studies involved in each theme

For peer review only

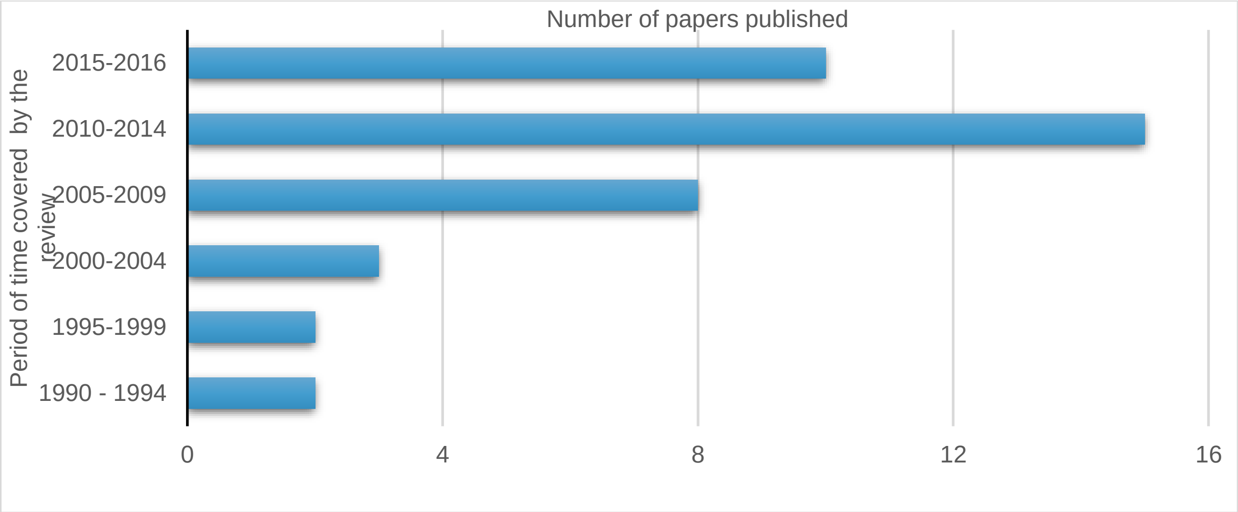

Figure 4: The range of publication dates for the included studies (1990–2016).

For peer review only

Figure 5: Immigrant women's experiences of maternity care in the UK

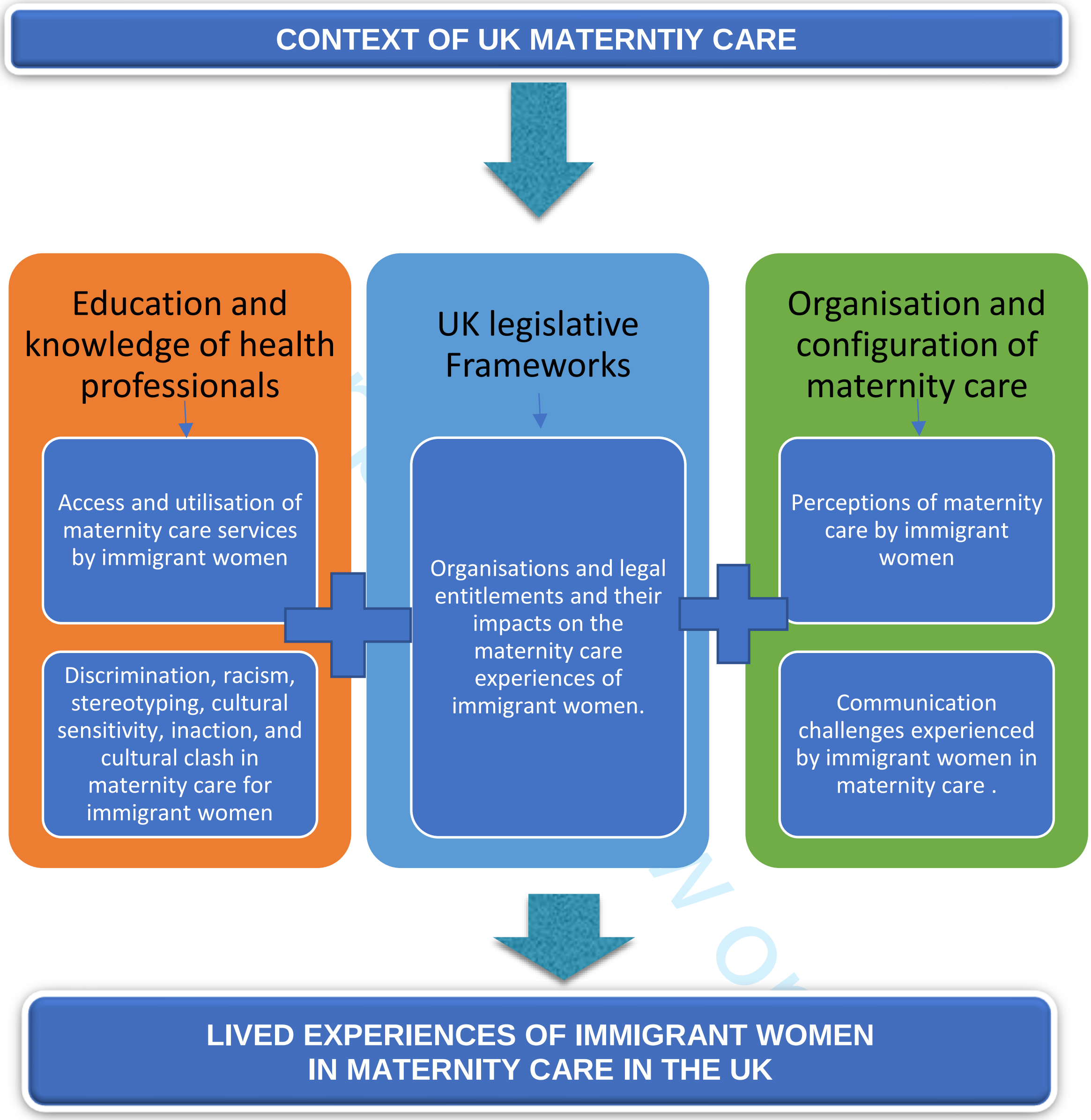

SUPPLEMENTARY FILES

File 1: Search Strategy
File 2: Bibliographic databases searched
File 3: Inclusion/Exclusion critiera
File 4: Excluded papers and rationale
File 5: Master table of included studies
File 6: Characteristics of study participants

For peer review only

File 1: Search Strategy

:Review search strategy - Medline

1	Maternal Health Services/ or Postnatal Care/ or Preconception Care/ or Prenatal Care/ or Perinatal Care/ or Infant Care/ or Midwifery/ or Obstetrics/ or General Practitioners/ or Primary Health Care/ or Family Health/	162335	
2	((maternal or child* or baby or babies or fetus* or fetal* or	119288	Field modified from .mp. to .ti,ab.
3	((birth* or matern* or mother* or pregnan* or childbearing or child-bearing or prenatal or pre-natal or postnatal or post-natal or perinatal or peri-natal or **preconception** or pre-conception or antenatal or ante-natal or postpartum or puerperium) adj3 (health* or nurs* or care or service*)).ti,ab.	65240	Field modified from .mp. to .ti,ab.
4	exp Midwifery/ or exp Obstetric Nursing/ or exp	47851	Field modified from .mp. to .ti,ab.
5	exp Health Services Accessibility/ or exp	1829149	
6	5 and (matern* or child* or baby or babies or fetus* or fetal* or embryo* or obstetric* or birth* or mother* or pregnan* or childbearing or child-bearing or prenatal or pre-natal or postnatal or post-natal or perinatal or peri-natal or preconception or pre-conception or antenatal or ante-natal or postpartum or puerperium).ti,ab.	253992	Limit 5 to female did not sufficiently focus previous search strategy so text terms used instead; Field modified from .mp. to .ti,ab.
7	1 or 2 or 3 or 4 or 6	490776	
8	("use" or access* or utili* or consum* or block* or hurdl* or barrier* or hindr* or hinder* or obstacle* or exclu* or discrimin* or disparit* or disproportion* or inequal* or unequal* or inadequat* or insuffic* or stratif* or limit* or lack* or unreliab* or poor* or poverty* or depriv* or disadvantag* or insecur* or insensit* or status* or entitl* or uninform* or ill-inform* or benefit* or interven* or deliver* or effective* or cost effective*).ti,ab.	8190143	

For peer review only

9	5 and 8	761677	use of/access to health services
10	"Emigrants and Immigrants"/ or Refugees/ or "Transients and Migrants"/ or "Emigration and Immigration"/	42486	
11	(((established or "first generation*" or new* or recent* or current*) adj3 (migrant* or migrat* or immigrant* or immigrat* or emigrant* or emigrat* or emigre* or expat* or (ex adj pat*) or transient* or alien*)) or newcomer* or (new adj comer*) or incomer*	14965	Revised to focus on established or new immigrant groups; Field modified from .mp. to .ti,ab.
12	(refugee* or (asylum adj seek*) or asylee* or (refused adj3 (asylum* or refugee*)) or (displaced adj person*) or exile* or (new adj arrival) or (country adj2 (birth or origin)) or **transnational*).ti,ab.**	13603	Field modified from .mp. to .ti,ab.
13	(foreigner* or (foreign adj (born or citizen* or national* or origin*)) or (non adj (citizen* or native*)) or ((adoptive or naturali#ed) adj (citizen* or resident*)) or overstay* or trafficked or "spousal migrant*").ti,ab.	10542	Additional migrants terminology; Field modified from .mp. to .ti,ab.
14	("non-UK-born" or "born outside the UK" or "length of residence in the UK" or (("not lawful*" or "not legal*" or unlawful* or illegal* or unauthori#ed* or "not authori#ed" or uncertain or insecure or illegal or legal or irregular* or refused or **undocumented)** adj3 (residen* or immigrant* or imigrat* or migrant* or migrat*))).ti,ab.	1375	Additional migrants terminology; Field modified from .mp. to .ti,ab.
15	exp Ethnic Groups/ or (ethnic* or ethno* or race or racial*).ti,ab.	282908	Expanded ethnic terminology; Field modified
16	exp african continental ancestry group/ or exp asian continental ancestry group/ or exp Caribbean Region/	152457	Additional ethnic terminology to specify South Asian and African Caribbean groups;
17	exp Vulnerable Populations/ or ((vulnerab* or disadvantag* or minorit*) adj3 (individ* or	35822	Expanded vulnerable populations terminology; Field

For peer review only

18	("Black and Minority Ethnic" or "Black & Minority ethnic" or BME or african caribbean* or afro caribbean* or black african* or (west adj (indies or indian*))).ti,ab.	7587	Expanded ethnic terminology; Field modified from .mp. to .ti,ab.
19	(south asia* or afghan* or bangladesh*	163384	
20	10 or 11 or 12 or 13 or 14 or 15 or 16 or 17 or 18 or 19	589408	All ethnic/migrant groups
21	7 and 9 and 20	20754	Maternity health services AND [illegible]
22	limit 21 to yr="1990 -Current"	18783	Time range expanded
23	higginbottom*.au.	214	
24	22 and 23	9	Check of strategy retrieval of known relevant records

For peer review only

File 2: Bibliographic databases searched

Databases searched.

- Ovid MEDLINE 1948– and MEDLINE In-Process and Other Non-Indexed Citations to daily update
- Ovid EMBASE 1980–2017 Week 11
- Ovid PsycINFO 1972–March Week 3 2017
- CINAHL Plus with Full Text/EBSCOHost to 2017
- MIDIRS on Ovid 1971 to April 2017
- Thomson Reuters Web of Science* 1900–2017
- ASSIA on ProQuest 1987–current
- HMIC on Ovid 1979–January 2017
- POPline (via http:// www.popline.org/) 1970 to the present

* Thomson Reuters Web of Science 1900-2017 includes the following:

- Science Citation Index Expanded (SCI-EXPANDED) 1900–2017
- Social Sciences Citation Index (SSCI) 1956–2017
- Conference Proceedings Citation Index - Science (CPCI-S) 1990–2017
- Conference Proceedings Citation Index - Social Science and Humanities (CPCI-SSH) 1990–2017
- Book Citation Index - Science (BKCI-S) 2008–2017
- Book Citation Index - Social Science and Humanities (BKCI-SSH) 2008–2017
- Emerging Sources Citation Index (ESCI) - 2015–2017

For peer review only

List of databases for searching grey literature

- **Cochrane Database of Systematic Reviews**
  http://www.thecochranelibrary.com/

Theses

- **Nottingham eDissertations**
  http://edissertations.nottingham.ac.uk/
- selected dissertations from UoN

- **Nottingham eTheses**
  http://etheses.nottingham.ac.uk/
- research degree theses awarded by UoN
- pilot project so not compulsory to submit, therefore not all these included

- **Index to Theses**
  http://www.theses.com/
  theses (incl. abstracts) accepted for higher degrees by universities in GB and Ireland
  now part of ProQuest Dissertations & Theses – UK & Ireland

- **Networked Digital Library of Theses & Dissertations**
  http://www.ndltd.org/
- includes theses and dissertations submitted to over 200 universities worldwide

- **EThOS – British Library Electronic Theses Online**
  http://ethos.bl.uk/Home.do

- **DEEP – DART Europe**
  http://www.dart-europe.eu

- **ProQuest Dissertations & Theses A&I** (worldwide coverage)
  http://search.proquest.com/pqdt/index?accountid=8018

Research Funders

- **Wellcome Trust**
  http://www.wellcome.ac.uk/
- Global charitable foundation supporting biomedical research and the medical humanities
- Provides support with funding, managing grants, education resources, application of research

- **Research Councils UK**
  http://www.rcuk.ac.uk/
- Support research across all academic disciplines
- Offer funding opportunities, international collaborations and training

For peer review only

- **Medical Research Council**
  http://www.mrc.ac.uk/index.htm
- Publicly funded organisation dedicated to improving human health
- Supports research across medical sciences in universities, hospitals and MRC Councils

- **Science and Technology Facilities Council**
  http://www.stfc.ac.uk/
- Independent public body of the Department of Business, Innovations and Skills
- Supports researchers across the sciences with the academic and industrial communities

- **National Institute for Health and Care Excellence**
  http://www.nice.org.uk/

- **Institute for Public Policy and Research**
  http://www.ippr.org/

- **ESRC**
  http://www.esrc.ac.uk/
  REGARD database

- **Clinical Research Network** (part of the NHS National Institute for Health Research)
  http://www.crncc.nihr.ac.uk
  From the NIHR portal
  (https://portal.nihr.ac.uk/Pages/NIHRResearchInfoStatement.aspx):
  - The repository for this information is the Portfolio Database, which currently contains approximately 2,000 studies, and can be accessed for public searching. Detailed instructions on how to search the Portfolio Database are available at http://www.ukcrn.org.uk/index/clinical/portfolio_new/P_search.html, and the Portfolio database is available at http://public.ukcrn.org.uk/search.
  - The National Research Register has been archived as a public resource and to support historical analysis. The archive is available via the National Institute for Health Research Portal http://portal.nihr.ac.uk.

Statistics

- **Department of Health**
  https://www.gov.uk/government/publications?publication_filter_option=statistics

- **UK Data Archive**
  http://www.data-archive.ac.uk

- **UK National Statistics**
  http://www.statistics.gov.uk

- **OECD Statistics Portal**
  http://www.oecd.org/statistics

- **World Health Organisation**

http://www.euro.who.int/en

- **NICE Evidence Services** (formerly NHS Evidence)
- Evidence search
- Clinical Knowledge Summaries
- NICE guidelines
- Journals and databases
- A-Z of topics – e.g. Diabetes
- Medicines information
- Public health information

https://www.evidence.nhs.uk

- **HMIC** (Health Management Information Consortium) – on Ovid
- combined database of the Department of Health, plus the King's Fund Information & Library Service
- official publications, journal articles, grey literature
- health service policy, management & admin, quality of hospitals, nursing, primary care and public health; occupational health; control/regulation of medicines

- **PAIS International**

  http://search.proquest.com/pais?accountid=8018 (via ProQuest)
- includes e.g.: gov docs, statistical directories, grey lit, research reports – mostly in social sciences

- **Open Grey**

  http://www.opengrey.eu/
- open access to grey literature published in Europe, including reports, dissertations, conference proceedings, official publications

- **Mednar**

  http://mednar.com/mednar/desktop/en/green/search.html

  one-stop federated search engine designed for professional medical researchers

- **WorldwideScience**

  http://worldwidescience.org/

- **OAIster**

  http://www.oclc.org/oaister.en.html

  catalog of millions of records from open access collections worldwide using the Open Archives Initiative Protocol for Metadata Harvesting (OAI-PMH)

- **Internet Archive Wayback Machine**

  http://archive.org/web/

  aims to provide permanent access to historical collections that exist in digital format

For peer review only

For peer review only

File 3: Inclusion/Exclusion criteria

Inclusion Criteria

Population	Immigrant women from any country other than England, Scotland, Northern Ireland or Wales
Phenomena of Interest	Maternity care
Context Setting	United Kingdom
Study designs	Qualitative, quantitative and mixed methods studies
Language	English
Date limitations	Jan 1990 - Jan 2018
**Exclusion Criteria**	
Context	Studies located in any country other than England, Scotland, Northern Ireland or Wales
Participants	Black and minority ethnic women born in the United Kingdom
Study Design	Non-empirical research, opinion pieces or editorial

For peer review only

File 4: Excluded papers and rationale

Excluded studies with reasons for exclusion.

Exclusion number	Reference	Reasons for exclusion
**1**	Bowler I. 'They're not the same as us': midwives' stereotypes of South Asian descent maternity patients. *Sociol Health Illn*. 1993 Mar 1;15(2):157-78.	Presented professionals' perspectives: focused on midwife interviews and observational data on midwives.
**2**	Straus L, McEwen A, Hussein FM. Somali women's experience of childbirth in the UK: perspectives from Somali health workers. *Midwifery*. 2 09 Apr 1;25(2):181-6.	Presented professionals' perspectives: interviewed Somali health workers and not the immigrant women.
**3**	Bowler IM. Stereotypes of women of Asian descent in midwifery: some evidence. *Midwifery*. 1993 Mar 1;9(1):7-16.	Presented professionals' perspectives: interviewed midwives.
**4**	Haith-Cooper M, Bradshaw G. Meeting the health and social needs of pregnant asylum seekers: midwifery students' perspectives. Part 2: Dominant discourses and approaches to care. *Nurse Educ Today*. 2013 Aug 1;33(8):772-7.	Presented professionals' perspectives: focused on midwifery students' perceptions.
**5**	Haith-Cooper M, Bradshaw G. Meeting the health and social care needs of pregnant asylum seekers; midwifery students' perspectives: Part 3; The pregnant woman within the global context; an inclusive model for midwifery education to address the needs of asylum-seeking women in the UK. *Nurse Educ Today*. 2013 Sep 1;33(9):1045-50.	Presented professionals' perspectives: interviewed midwives.
**6**	Balaam MC, Kingdon C, Thomson G, Finlayson K, Downe S. 'We make them feel special': the experiences of voluntary sector workers supporting asylum-seeking and refugee women during pregnancy and early motherhood. *Midwifery*. 2016 Mar 1;34:133-40.	Presented professionals' perspectives.
**7**	Richards J, Kliner M, Brierley S, Stroud L. Maternal and infant health of Eastern Europeans in Bradford, UK: a qualitative study. *Community Practitioner*. 2014 Sep 1;87(9):33.	Presented professionals' perspectives.
**8**	Redshaw M, Heikkilä K. Ethnic differences in women's worries about labour and birth. *Ethn Health*. 2011 Jun 1;16(3):213-23.	Mixed sample of UK-born BME and immigrant women with no separate findings reported for immigrant women.

**9**	Darwin Z, Green J, McLeish J, Willmot H, Spiby H. Evaluation of trained volunteer doula services for disadvantaged women in five areas in England: women's experiences. *Health Social Care Community*. 2017 Mar 1;25(2):466-77.	Mixed sample of UK-born BME and immigrant women with no separate findings reported for immigrant women.
**10**	Dunne FP, Brydon PA, Proffitt M, Smith T, Gee H, Holder RL. Fetal and maternal outcomes in Indo-Asian compared to Caucasian women with diabetes in pregnancy. *QJM*. 2000 Dec 1;93(12):813-8.	Mixed sample of Indo-Asian women born inside and outside the UK with no separate findings for immigrant women.
**11**	Ball HL, Moya E, Fairley L, Westman J, Oddie S, Wright J. Infant care practices related to sudden infant death syndrome in South Asian and White British families in the UK. *Paediatr Perinat Epidemiol*. 2012 Jan 1;26(1):3-12.	Focused on care of infants aged 2-4 months, but our chosen limit of maternity care was only up to 6 weeks after birth.<br>Mixed sample of UK-born and non-UK-born women with no separate findings for immigrant women.
**12**	McCarthy R, Haith-Cooper M. Evaluating the impact of befriending for pregnant asylum-seeking and refugee women. *Br J Midwifery*. 2013 Jun;21(6):404-9.	Not an empirical: the study does not report its methodology, sampling, or data analysis.
**13**	Streetly A, Grant C, Bickler G, Eldridge P, Bird S, Griffiths W. Variation in coverage by ethnic group of neonatal (Guthrie) screening programme in south London. *BMJ*. 1994 Aug 6;309(6951):372-4.	Mixed sample of ethnic groups with no separate findings for immigrant women.
**14**	Burchill J, Pevalin DJ. Demonstrating cultural competence within health-visiting practice: working with refugee and asylum-seeking families. *Divers Equal Health Care*. 2014;11(2).	Weak focus on maternity care: just two quotes on the influence of cultural sensitivity training and on addressing female genital mutilation (FGM) in maternity.
**15**	Dormandy E, Michie S, Hooper R, Marteau TM. Low uptake of prenatal screening for Down syndrome in minority ethnic groups and socially deprived groups: a reflection of women's attitudes or a failure to facilitate informed choices? *Int J Epidemiol*. 2005 Feb 28;34(2):346-52.	Not clear if sample was composed of immigrant women: no separate findings for immigrant women.
**16**	Henderson J, Gao H, Redshaw M. Experiencing maternity care: the care received and perceptions of women from different ethnic groups. *BMC Pregnancy Childbirth*. 2013 Dec;13(1):196.	Not clear if sample was composed of immigrant women: no separate findings for immigrant women.
**17**	Ingram J, Cann K, Peacock J, Potter B. Exploring the barriers to exclusive breastfeeding in Black and minority ethnic groups and young mothers in the UK. *Matern Child Nutr*. 2008 Jul 1;4(3):171-80.	Mixed sample of UK-born and immigrant women with no separate findings for immigrant women.

**18**	Parsons L, Day S. Improving obstetric outcomes in ethnic minorities: an evaluation of health advocacy in Hackney. *J Public Health*. 1992 Jun 1;14(2):183-91.	Not clear if sample was composed of immigrant women.
**19**	Knight M, Kurinczuk JJ, Spark P, Brocklehurst P. Inequalities in maternal health: national cohort study of ethnic variation in severe maternal morbidities. *BMJ*. 2009 Mar 4;338:b542.	Mixed sample of both UK- and foreign-born BME with no separate findings for immigrant women.
**20**	Almond P, Lathlean J. Inequity in provision of and access to health-visiting postnatal depression services. *J Adv Nurs*. 2011 Nov 1;67(11):2350-62.	Focused on professionals' perspective. Eight of the nine participants were immigrant women, but just three brief quotes were reported from immigrant Bangladeshi women. Authors did not reply to our request for clarification of the immigrant status of the sample.
**21**	Row MA, Nevill AM, Young DB, Adamson-Macedo EN. (2013) Promoting positive postpartum mental health through exercise in ethnically diverse priority groups. *Divers Equal Health Care*. 2013;10(3)185-195.	Mixed sample of minority ethnicity women born in and outside the UK with no separate findings for immigrant women.
**22**	Hemingway H, Saunders D, Parsons L. Social class, spoken language and pattern of care as determinants of continuity of carer in maternity services in east London. *J Public Health*. 1997 Jun 1;19(2):156-61.	Mixed sample of women with and without English as a first language. We used lack of English as a proxy for immigrant, but only one finding was reported for a non-English sample (i.e., the presence of an advocate who could translate for women visiting midwives or doctors). Did not receive a reply from the authors regarding the immigrant status of the sample.
**23**	Ingram J, Johnson D, Hamid N. South Asian grandmothers' influence on breast feeding in Bristol. *Midwifery*. 2003 Dec 1;19(4):318-27.	No clarity on the immigrant status of the sample and no separate findings for immigrant women. Did not receive a reply from the authors on the immigrant status of the sample.
**24**	Gardner PL, Bunton P, Edge D, Wittkowski A. The experience of postnatal depression in West African mothers living in the United Kingdom: A qualitative study. *Midwifery*. 2014 Jun 1;30(6):756-63.	No clarity on the immigrant status of the sample and no separate findings for immigrant women.
**25**	Kelly Y, Panico L, Bartley M, Marmot M, Nazroo J, Sacker A. Why does birthweight vary among ethnic groups in the UK? Findings from the Millennium Cohort Study. *J Public Health*. 2008 Jul 21;31(1):131-7.	Not clear if participants included immigrant women. Did not receive a reply from the authors on the immigrant status of the sample.

**26**	Beake S, McCourt C, Bick D. Women's views of hospital and community-based postnatal care: the good, the bad and the indifferent. *Evid Based Midwifery*. 2005 Dec 1;3(2):80-7.	Not clear if participants included immigrant women. Did not receive a reply from the authors on the immigrant status of the sample.
**27**	Ahmed S, Green J, Hewison J. Antenatal thalassaemia carrier testing: women's perceptions of information and consent. *J Med Screen*. 2005 Jun 1;12(2):69-77.	Weak focus on maternity care: main focus was on an ancestry issue.
**28**	Dyson SM, Cochran F, Culley L, Dyson SE, Kennefick A, Kirkham M, Morris P, Sutton F, Squire P. Ethnicity questions and antenatal screening for sickle cell/thalassaemia (EQUANS) in England: observation and interview study. *Crit Public Health*. 2007 Mar 1;17(1):31-43.	Not clear if participants included immigrant women.
**29**	Baker D, Garrow A, Shiels C. Inequalities in immunisation and breast feeding in an ethnically diverse urban area: cross-sectional study in Manchester, UK. *J Epidemiol Community Health*. 2011 Apr 1;65(4):346-52.	Not clear if participants included immigrant women and not focused on maternity care. Did not receive a reply from the authors on the immigrant status of the sample.
**30**	Dyson SM, Chambers K, Gawler S, Hubbard S, Jivanji V, Sutton F, Squire P. Lessons for intermediate- and low-prevalence areas in England from the Ethnicity Questions and Antenatal Screening for sickle cell/thalassaemia (EQUANS) study. *Divers Health Social Care*. 2007 Jun 1;4(2).	Not clear if participants included immigrant women and not focused on maternity care.
**31**	Sim JA, Ulanika AA, Katikireddi SV, Gorman D. 'Out of two bad choices, I took the slightly better one': Vaccination dilemmas for Scottish and Polish migrant women during the H1N1 influenza pandemic. *Public Health*. 2011 Aug 1;125(8):505-11.	Not focused on maternity care.
**32**	Wittkowski A, Zumla A, Glendenning S, Fox JR. The experience of postnatal depression in South Asian mothers living in Great Britain: a qualitative study. *J Reprod Infant Psychol*. 2011 Nov 1;29(5):480-92.	Mixed sample with only two quotes related to immigrant women.
**33**	McFadden A, Atkin K, Renfrew MJ. The impact of transnational migration on intergenerational transmission of knowledge and practice related to breast feeding. *Midwifery*. 2014 Apr 1;30(4):439-46.	Not focused on maternity care.
**34**	Datta S, Alfaham M, Davies DP, Dunstan F, Woodhead S, Evans J, Richards B. Vitamin D deficiency in pregnant women from a non-European ethnic minority population – an interventional study. *BJOG*. 2002 Aug 1;109(8):905-8.	Not clear if participants included immigrant women. Did not receive a reply from the authors on the immigrant status of the sample.

**35**	McLeish J, Redshaw M. 'I didn't think we'd be dealing with stuff like this': a qualitative study of volunteer support for very disadvantaged pregnant women and new mothers. *Midwifery*. 2017 Feb 1;45:36-43.	Mixed sample with no separate findings for immigrant women.

For peer review only

File 5: Master table of included studies

Summary of included studies

Reference	Study aim	Region	Methodology	Theory or Framework	Setting	Data analysis	Sample and mode of recruitment
109	To establish efficacy of linkworker services (an intervention) introduced for non-English-speaking Asian women in multi-racial health districts	Not specified	Quantitative survey: 21-item questionnaire	Not specified.		Qualitative: content analysis	Questionnaire to the Heads of Midwifery Services in 30 multi-racial district health authorities. 20 responded. Sample is not immigrant women, however this is an evaluation of an intervention
115	To develop a reliable and valid questionnaire to evaluate satisfaction with maternity care in Sylheti-speaking Bangladeshi women.	London.	Mixed methods: two-stage psychometric study. Firstly, a Sylheti-language questionnaire regarding Bangladeshi women's experiences of maternity services was translated and culturally adapted from an English-language questionnaire using focus groups, in-depth interviews, and iterative methods. Secondly, quantitative psychometric methods were used to field test and evaluate the acceptability, reliability, and validity of this questionnaire.	Not specified.	Not specified.	Qualitative: thematical analysis. Quantitative: validity of an instrument.	Located at four hospitals providing maternity services in London, UK. Study participants included 242 women from the London Bangladeshi communities who were in the antenatal (at least 4 months pregnant) or postnatal phase (up to 6 months after delivery). The women spoke Sylheti, a language with no accepted written form. In stage one purposive samples of 40 women in the antenatal or postnatal phase participated, along with one convenience sample of six women in the antenatal phase and three consecutive samples of 60 women in the postnatal phase. In stage two, 135 women (main sample) completed the questionnaire 2 months after delivery (82% response rate), and 50 women (retest sample) from the main sample completed a second questionnaire 2 weeks later (96% response rate).

For peer review only

88	To study the maternity care experiences of Somali refugee women in an area of West London. This article focused particularly on findings relating to the language barrier, which to a large degree underpinned or at least aggravated other problems the women experienced.	West London.	Qualitative: case study. Six semi-structured interviews and two focus groups (with six participants each).	Not specified.	Not specified.	Qualitative: thematical analysis.	Snowball sampling: 12 Somali women were selected from a larger survey involving 1400 women.
89	To undertake a qualitative study of the maternity experiences of 33 asylum seekers.	London, Plymouth, Hastings, Brighton, Oxford, Manchester, and King's Lynn.	Qualitative.	Not specified.	Home or a neutral location.	Qualitative: content analysis.	Convenience and snowball sampling of recent asylum seekers. Based on semi-structured interviews carried out in seven English cities.
116	A Sure Start local programme had funded a Bangladeshi support worker to provide bilingual breastfeeding support to childbearing Bangladeshi women, many of whom were not fluent in English. This study aimed to conduct a short evaluation of the impact of this work on the uptake and duration of breastfeeding among these women.	Tower Hamlets.	Mixed methods: the survey questionnaire included some open and closed questions about the women's intention to feed; their current feeding methods; the breastfeeding support and information they received antenatally, during the hospital stay, and postnatally; overall views on the information and support received; and some demographic details. Eleven interviews were conducted by telephone in Sylheti (a dialect that has no written format), three in English and one in Urdu (using a female family member to translate). Interviews took between 15 and 30	Not specified.	Not specified (survey conducted by telephone).	Qualitative: content analysis of a questionnaire (open and closed questions).	The two midwives and the support worker had provided breastfeeding support to 194 women during a one-year period (September 2001 to August 2002). Of these, 80 women received help from the support worker alone. The majority of these 80 women were Bangladeshi. For the evaluation, 15 women were randomly selected from these 80 women.

			minutes to complete.				
**{Rowe, 2008 #18 3}**	To identify any social or ethnic differences in access to antenatal care and to quantify the effects of any such differences using data collected in a survey of women's experiences of antenatal screening.	England.	Quantitative: a cross-sectional survey using a postal questionnaire.	Not specified.	Not specified.	Quantitative: cross-sectional analysis.	A stratified clustered random sampling strategy was used. Hospitals in England were stratified according to ethnic mix. To ensure inclusion of an adequate number of women from black and minority ethnicity (BME) backgrounds, hospitals with ≥ 15% of women of BME origin were oversampled. Pregnant women aged ≥16 years and receiving care in 15 participating hospitals were sent a postal questionnaire at 27–31 weeks of gestation.

iew only

110	To compare the health behaviours both antenatally (smoking and alcohol consumption) and postnatally (initiation and duration of breast feeding) of mothers who have white British or Irish heritage with those of mothers from ethnic minority groups and to examine in mothers from ethnic minority groups whether indicators of acculturation (generational status, language spoken at home, and length of residency in the UK) were associated with these health behaviours.	England.	Quantitative: a prospective nationally representative cohort study.	Not specified.	Not specified.	Quantitative: cohort study.	Stratified clustered sampling framework to over-represent mothers from ethnic minority groups and disadvantaged areas produced 6478 white British or Irish mothers and 2110 mothers from ethnic minority groups. Of those from ethnic minority groups, 681 (33%) were first generation and 55 (4%) second generation.
90	To explore and synthesise the maternity care experiences of female asylum seekers and refugees.	UK.	Qualitative: multiple exploratory longitudinal case studies that used a series of interviews, photographs taken by the women, field notes, and observational methods to contextualise data obtained during 2002 and 2003.	Theory of interactions and transformational educational theory.	Hospital settings or women's homes.	Qualitative: thematical analysis.	Women were approached if the status of 'asylum seeker' or 'refugee' was written in the hospital notes taken at their booking appointment. Fourteen women were approached, but nine women declined to participate. Five women consented, but one woman was dispersed before 20 weeks gestation and therefore was not included in the study. Of the remaining four participating women, three were asylum seekers and one was a refugee. The sampling technique was not clearly reported.

For peer review only

91	To identify key features of communication across antenatal care and whether they are evaluated positively or negatively by service users.	Central London.	Qualitative: used six focus groups of 15 participants each and conducted 15 semi-structured interviews. Non-English-speaking focus groups and interviews were conducted in standard Bengali, Sylheti, or Somali.	Not specified.	Focus groups: hospitals and university meeting rooms. Semi-structured interviews: various locations to suit the needs of the women.	Qualitative: thematical analysis.	The sampling technique was not clearly reported, but they recruited 30 pregnant women from diverse social and ethnic backgrounds affiliated with one NHS Trust (i.e., hospital) in central London. Participants were recruited within this hospital, in eight community antenatal clinics situated in socially and ethnically diverse areas, via a community parenting group for Somali women, and via a Bengali Women's Health Project. Within the hospital, participants were recruited from the antenatal waiting room (which services low- and high-risk pregnancies), the ultrasound clinic, and the glucose tolerance testing clinic.
111	To determine the pregnancy outcomes of women of similar parity and ethnic background who received antenatal care ('booked') compared those who did not ('unbooked') over a period of 18 months.	North Middlesex University Hospital (NMUH), London.	Quantitative: a retrospective cohort study from September 2006 to March 2008 comparing the socio-demographics and the foetal and maternal outcomes of pregnancies of unbooked versus booked women.	Not specified.	Not specified.	Quantitative: a retrospective cohort study.	Women who received no antenatal care or who delivered within 3 days of their initial booking visit were categorised as 'unbooked'. In each case, the woman who had delivered next on the labour ward register (matched for ethnicity and parity) and who had received antenatal care prior to the second trimester served as a comparison.
117	To explore the perspectives of first- and second-generation women of Pakistani origin on maternity care and to make recommendations for culturally appropriate support and care from maternity services.	West Midlands.	Mixed methods: a retrospective Q methodology study of Pakistani women following childbirth.	Retrospective Q method study.	Not specified.	Qualitative: Q methodology.	A purposive sampling strategy was used. Postnatal first- and second-generation Pakistani women were self-identified by their responses to information leaflets disseminated at local Children's Centres across an inner city in the West Midlands.

92	To study the relationships between Somali women and their Western obstetric care providers. The attitudes, perceptions, beliefs, and experiences of both groups were explored in relation to caesarean sections, particularly to identify factors that might lead to adverse obstetric outcomes.	Greater London.	Qualitative: in-depth individual and focus group interviews.	Framework of naturalistic enquiry, emic/etic model	Not specified.	Qualitative: emic/etic analysis.	Selected 39 Somali women by snowball sampling, 36 from the community and three purposively from a hospital.
93	To address the postulates that immigrant women experience sensitive care through the use of an ethnically congruent interpreter and that such women prefer to meet health providers of the same ethnic and gender profile when in a multi-ethnic obstetrics care setting.	Greater London.	Qualitative: in-depth individual and focus group interviews. Open-ended questions were presented by an obstetrician and an anthropologist.	Framework of naturalistic enquiry.	Not specified.	Qualitative: naturalistic inquiry.	Participants were recruited throughout Greater London between 2005 and 2006. Snowball sampling was used to recruit 36 immigrant Somali women, and another three were selected by a by purposive technique for a total of 39. A purposive technique was used to select further 11 Ghanaian women who had delivered at least one child within the British healthcare system and who were living within the study area at the time of data collection.

For peer review only

118	To evaluate a pilot mental health service for asylum-seeking mothers and babies.	UK (not clear).	Mixed methods: evaluation within a participatory action research framework.	Participatory action research framework.	Not specified.	Qualitative: thematical analysis. Quantitative: the CARE-Index.	An active outreach recruitment strategy was adopted by psychologists, who embedded themselves in a drop-in community group, the Merseyside Refugee & Asylum Seekers & Asylum Seekers Pre & Postnatal Support Group. Participants were West African women who were asylum seekers or refugee and who were either pregnant or had a young baby. They originated from The Gambia, Sierra Leone, Ivory Coast, and Nigeria. All spoke English. Their ages ranged from 17 to 32 years, and all babies were under 6 months of age at the point of initial contact, with three babies not yet born. Attendance at the 21 therapeutic group sessions ranged between 4 and 12 mothers (with their babies). Seven mothers attended a significant proportion or all group sessions. An additional six mothers attended 1-4 group sessions.
94	To apply the 'three delays' framework (developed for low-income African contexts) to a high-income Western scenario to identify delay-causing influences in the pathway to optimal facility treatment.	Greater London.	Qualitative: individual and focus group interviews.	'Three delays' framework.	Not specified.	Constructivist hermeneutic naturalistic study.	Purposive and snowball sampling was used to recruit 54 immigrant women originally from sub-Saharan regions in Africa (Somalia, Ghana, Nigeria, Senegal, and Eritrea) living in London and to recruit 32 maternal providers.

For peer review only

112	To identify predictors of late initiation of antenatal care within an ethnically diverse cohort.	Newham, East London.	Quantitative: a cross-sectional analysis of routinely collected electronic patient records from Newham University Hospital NHS Trust (NUHT).	Not specified.	Not specified.	Quantitative: cross-sectional analysis.	All women who attended their antenatal booking appointment within NUHT between 1st January 2008 and 24th January 2011 were included in this study. The main outcome measure was late antenatal booking, defined as attendance at the antenatal booking appointment after 12 weeks (+6 days) gestation. The sample included women from Somalia, Eastern Europe, Africa, the Caribbean, and South Asia.
87	To explore BME women's experiences of contemporary maternity care in England.	All over England.	Qualitative data collected from a large cross-sectional survey using three open-ended questions that encouraged participants to articulate their experience of maternity care in their own words.	Not specified.	Not specified.	Qualitative: Thematic analysis.	A random sample of 4800 women was selected using Office for National Statistics (ONS) birth registration records. The overall response rate was 63% but was only 3% from BME groups. A total of 368 women self-identified as coming from BME groups. Of those, 219 (60%) responded with open text and 132 (60%) were born outside the UK.
95	To investigate women's experiences of dispersal in pregnancy and to explore the effects of dispersal on the health and maternity care of women asylum seekers who were dispersed during pregnancy in the light of NICE guidelines on antenatal, intrapartum, and postnatal care.	London, South of England, Midlands and East of England, North West, North East, and Wales.	Qualitative: interviews were conducted with 19 women who had been dispersed during their pregnancies and with one woman kept in an Initial Accommodation Centre under a new Home Office pregnancy and dispersal guidance issued in 2012.	Not specified.	Not specified.	Qualitative (not clear).	The sampling technique was not mentioned clearly. The women interviewed came from 14 different countries and had been dispersed or relocated to or within six regions of the UK. At the time of dispersal, 14 had been awaiting a decision on their asylum claim and six had been refused asylum.

For peer review only

113	To compare the maternal and birth outcomes of Polish and Scottish women having babies in Scotland and to describe any differences in clinical profiles and service use associated with migration from Poland.	All over Scotland.	Quantitative: a population-based epidemiological study of linked maternal country of birth, maternity, and birth outcomes. Scottish maternity and neonatal records linked to birth registrations were analysed for differences in modes of delivery and pregnancy outcomes between Polish migrants and Scots. These outcomes were also compared with Polish Health Fund and survey data.	Not specified.	Not specified.	Quantitative: statistical analysis.	The study analysed 119,698 Scottish and 3105 Polish births to primiparous women in Scotland in 2004-09 using routinely collected administrative data on maternal country of birth and birth outcome.
96	To understand the multiple influences on behaviour and hence the risks to metabolic health of South Asian mothers and their unborn children, to theorise how these influences interact and build over time, and to inform the design of culturally congruent, multi-level interventions.	London boroughs, Tower Hamlets, and Newham.	Qualitative: group story-sharing sessions and individual biographical life-narrative interviews.	Multi-level ecological models.	All but four interviews were in the participants' homes.	Qualitative: phenomenology.	The study recruited from diabetes and antenatal services in two deprived London boroughs 45 women of Bangladeshi, Indian, Sri Lankan, or Pakistani origin aged 21–45 years with histories of diabetes in pregnancy. Overall, 17 women shared their experiences of diabetes, pregnancy, and health services in group discussions, and 28 women gave individual narrative interviews (facilitated by multilingual researchers). All were audiotaped, translated, and transcribed.

For peer review only

9	To understand the nature of need in superdiverse areas and to examine the emergent challenges for effective maternity service delivery in an era of superdiversity.	West Midlands.	Mixed methods: the study used a semi-structured questionnaire and held narrative interviews of newcomer women. The findings were then triangulated with interviews of professionals who regularly worked with such women.	Not specified.	Not specified.	Qualitative: systematic thematic analysis. Quantitative: triangulation of findings.	Sampling was not described clearly. However, the study used a semi-structured questionnaire that was designed in collaboration with maternity professionals and community researchers to explore the views and maternity experiences of newcomer women. Experienced multilingual female community researchers completed 82 of these questionnaires with interviewees in a range of different languages. Narrative interviews were also held with 13 women to further explore issues. The findings were triangulated with 18 interviews of professionals who regularly worked with migrant women.
98	To explore how Somali women with FGM experienced and perceived antenatal and intrapartum care in England.	Birmingham.	Qualitative: a descriptive, exploratory study using face-to-face semi-structured interviews that were audio-recorded.	Not specified.	Private room.	Qualitative: thematical analysis.	The study used convenience and snowball sampling of ten Somali women in Birmingham who had received antenatal care in England in the past 5 years.
100	To explore differences in infant thermal care beliefs between mothers of South Asian and white British origin in Bradford, UK.	Bradford District, West Yorkshire.	Mixed methods: mothers were interviewed using a questionnaire with structured and unstructured questions.	Not specified.	The women chose the location of the interview.	Qualitative: thematical analysis.	A total of 102 mothers (51 South Asian and 51 white British) were recruited in Bradford District, West Yorkshire, UK. The inclusion criteria specified infants aged 13 months or less with a parent of South Asian or white British cultural origin who lived in the Bradford District. South Asia was defined as including the countries of Pakistan, India, Afghanistan, Sri Lanka and Nepal. Recruitment was aided by local community organisations, children's centres, and community contacts. Urdu- and Punjabi-speaking interpreters were requested and provided for 69 per cent of the first-generation South Asian mothers (n = 26) in the sample.

97	To gain an understanding of infant feeding practices among a group of UK-based refugee mothers.	Liverpool and Manchester.	Qualitative: two focus group discussions and 15 semi-structured interviews.	Not specified.	HCPs: private offices or clinics Refugee women: private rooms or discrete areas at the support venue (community centre or church hall).	Qualitative: thematical analysis.	The study purposively selected 30 refugee mothers from 19 countries who now resided in Liverpool or Manchester and were at least 6 months pregnant or had a child who had been born in the UK in the last 4 years. Of these 30, 19 were HIV-negative and 11 were HIV-positive.
119	To provide insights into possible causes of poor maternity outcomes for new migrants in the West Midlands region of the UK and to develop recommendations that could help improve maternity services for these migrants.	West Midlands.	Mixed methods: a semi-structured questionnaire and in-depth interviews.	Not specified.	Not specified.	Qualitative: systematic thematical approach. Quantitative: triangulation of the findings.	A non-probability purposive sample was generated by selecting 82 women who had moved to the UK within the past 5 years and had subsequently utilised maternity services. Of these, 13 underwent in-depth interviews as well.
99	To explore the maternity care experiences of pregnant asylum-seeking women in West Yorkshire to inform service development.	West Yorkshire.	Qualitative: interpretative approach within the tradition of hermeneutic phenomenology.	Not specified.	Not specified.	Qualitative: interpretive approach with hermeneutic phenomenology analysis.	Purposive sampling was performed through the voluntary sector and a children's centre. In addition, word-of mouth led to an element of snowball sampling. Six women were recruited.
120	To provide locally applicable data on the needs of Black and minority ethnic women in relation to their uptake of maternity and neonatal care provision by primary healthcare teams in Leeds.	Leeds.	Mixed methods: questionnaires and focus groups. Interpreters were used when necessary for data collection. A questionnaire was translated into Urdu for some women.	Not specified.	Local community centres and in the participants' homes.	Qualitative: content analysis. Quantitative: survey (not clear).	A total of 97 questionnaires were completed, of which 50 were completed through informal links at community centres, schools, and in women homes. The remaining 47 were completed whilst the researcher attended various antenatal clinics in the community.

101	To study the effectiveness of three linkworker and advocacy schemes that were designed to empower minority ethnic community users of maternity services.	Birmingham.	Qualitative: focus group discussions, semi-structured interviews, and non-directive interviews.	Not specified.	Antenatal clinics in hospitals and health centres, community group settings, and participants' homes.	Qualitative: not clear, thematical analysis?	Individual interviews were conducted with 66 Asian women who had received support from linkworker and advocacy services during their pregnancy and postnatally. Of these, 28 were from Birmingham, 13 from Leeds, and 25 from Wandsworth-London. A semi-structured interview guide was translated into five Asian languages: Hindi, Punjabi, Gujarati, Urdu and Tamil. The study also included ten focus groups made up of 60 women who had not used linkworker or advocacy services. All participants were recruited with the help of various minority ethnic women's groups and community organisations. Interpreters assisted 11 personal interviews with non-users from Vietnamese and Chinese backgrounds.
62	To determine the nature of the barriers confronting women when they used antenatal and postnatal services.	Pollokshields, Glasgow.	Qualitative: semi-structured questionnaire.	Not specified.	Not specified.	Qualitative: thematic analysis.	Twenty women were interviewed in depth by a Centre's Health Development Worker. Of these, 17 were born outside the UK.
102	To study the maternity services experiences of Muslim parents in England.	UK: not specified.	Qualitative: focus groups with Muslim mothers to explore their experiences of and views about maternity services; questionnaires with Muslim fathers; and interviews with health professionals	Not specified.	Not specified.	Qualitative: content analysis.	A mixed sample of 43 immigrants and non-immigrants were recruited via their project advisory groups. The focus groups were conducted in various locations around the UK, with two focus group discussions in a language other than English. A total of eight health professionals were interviewed: six midwives (two of whom worked for Sure Start programmes), a health visitor, and a consultant obstetrician.

For peer review only

114	To determine the current clinical practice of maternity care in England, including the service provision and organisations that underpin care, from the perspective of women needing the care; to identify the key areas of concern for women receiving maternity care in England; and to determine whether and in what ways women's experiences and perceptions of care have changed over the last 10 years.	England: not specified.	Quantitative: survey.	Not specified.	Survey: not specified.	Quantitative: cross-sectional design.	Random samples of women selected for the pilot and main studies were identified by staff at the ONS using live birth registrations for births within 2 specific weeks: 2–8 January (pilot) and 4–10 March 2006 (main study). The same method of sampling was used as had been employed in 1995 to enable direct comparisons. Random samples of 400 women for the pilot survey and 4800 women for the main survey who were aged 16 years and over and who had delivered their baby in a one week period in England were selected. The sampling was stratified on the basis of births in different geographical areas (Government Office Regions (GORs)). No subgroups were oversampled. The usable response rate was 60% for the pilot survey and 63% for the main survey. The samples included 229 women of BME born outside the UK.

For peer review only

103	To explore the perceptions of pregnant asylum seekers in relation to the provision of their maternity care while in emergency accommodation in the UK.	South East of England.	Qualitative: an exploratory approach using unstructured interviews with five healthcare professionals and semi-structured interviews with ten pregnant asylum seekers.	Not specified.	Participants' emergency accommodations.	Qualitative: thematical analysis.	Purposive sampling of those providing maternity care for asylum seekers produced a sample comprising two midwives (M1 and M2), one GP (GP), one hospital consultant (C), and one nurse (N), all based in south coast health centres and hospitals. A total of 15 pregnant asylum seekers were approached to participate in the study. These women entered the UK through a south coast port over a three-month period. Their countries of origin were Algeria, Congo, Angola, Nigeria, Somalia, and Iraq, and they spoke French, Portuguese, Yoruba, Arabic, and Kurdish. Translated information letters and consent forms were distributed to pregnant asylum seekers via the Refugee Help Line, which also returned signed consent forms. This constitutes non-probability, purposive sampling.
104	To explore the meanings attributed by migrant Arab Muslim women to their experiences of childbirth in the UK. In particular, to explore migrant Arab Muslim women's experiences of maternity services in the UK; to examine the traditional childbearing beliefs and practices of Arab Muslim society; and to suggest ways to provide culturally sensitive care for this group of women.	UK: not specified.	Qualitative: an interpretive ontological-phenomenological perspective informed by the philosophical tenets of Heidegger (1927/1962).	Heideggerian hermeneutic phenomenology.	All interviews were in the participants' homes except for one, which took place in a restaurant after 10 pm.	Qualitative: thematical analysis.	Purposive sampling produced eight Arab Muslim women who had migrated to one multicultural city in the Midlands.

For peer review only

105	To examine the health-seeking behaviours of Korean migrant women living in the UK.	London.	Qualitative: 21 semi-structured interviews.	Foucauldian approach.	Not clear.	Qualitative: not clear.	Women were recruited from New Malden via Korean community contacts.
121	To explore perinatal clinical indicators and experiences of postnatal care among European and Middle Eastern migrant women and to compare them with those of British women at a tertiary hospital in the North East of Scotland.	North East of Scotland.	Mixed methods. Phase 1 of the research was a secondary analysis of routine data for 15,030 consecutive deliveries at Aberdeen Maternity Hospital. Phase 2 was a retrospective study of 26 European, Middle Eastern, and British mothers in this hospital. After the women had given birth, verbal data was collected using face-to-face semi-structured interviews.	Not clear.	Phase Two: 24 interviews were conducted in the homes of participants and two interviews at the University department.	Qualitative: thematical analysis. Quantitative: Phase One was a secondary analysis of routine data for 15,030 consecutive deliveries at Aberdeen Maternity Hospital. Phase 2 was a retrospective study of women.	Phase 1: The 15,030 deliveries included all births at Aberdeen Maternity Hospital over the financial years 2004–2008 in which maternal nationalities were identified and gestation was ≥ 24 weeks. Both singleton and multiple births were included. The clinical data was harvested from the Patient Administration System and the PROTOS maternity information system. In the case of women with multiple order births during the study, all births were included. Phase 2 of the research was a retrospective study of a few of the mothers who had given birth at this hospital. Eight European and five Middle Eastern women were semi-matched with 13 British women.

122	To assess the mechanisms of support available to EM (ethnic minority) communities from community and voluntary sector organisations in relation to maternal and infant nutrition (a mapping exercise); to explore the experiences of the targeted client groups in seeking and receiving such support; and to identify gaps and opportunities to enhance support mechanisms and engagement with diverse EM communities.	Glasgow, Edinburgh, Aberdeen, Stirling, Fife, Dundee, and Inverness.	Mixed methods: an online questionnaire survey of organisations working with EM communities, focus groups, and telephone interviews with EM women.	Not specified.	Not specified.	Qualitative: thematical analysis. Quantitative:	The study identified 65 community organisations that potentially provided food and health services across EM communities in Scotland. In total, 37 organisations replied to the survey. Of those organisations, 15 indicated that they are providing services in the area of maternal and infant nutrition. A further 12 indicated that despite working with EM communities, they do not provide services in maternal and infant nutrition or healthy eating in general. An additional ten organisations confirmed by telephone that they were or had been working with EM women, but were unable to undertake the survey. The majority of interviewees for the focus groups and interviews were selected in response to a request sent by Black and Ethnic Minorities Infrastructure in Scotland (BEMIS) to community organisations. Snowball sampling was used to provide further contacts. In total, four focus groups were conducted with Polish, Roma, Czech, and African mothers. In addition, six telephone interviews were conducted with Polish mothers. We focused on Polish mothers because they were the largest new ethnic group in Scotland since 2004.

For peer review only

106	To explore the experiences of obstetric care in Scotland among women who have undergone FGM.	Glasgow and Edinburgh.	Qualitative: personal experiences of FGM and interviews.	Interpretivism paradigm and feminist perspective.	The Dignity Alert & Research Forum (DARF) office or in the participant's home.	Qualitative: thematical analysis.	Convenience and purposive sampling resulted in a total number of seven women taking part in this study. All women were of African origin living in Scotland (three in Glasgow and four in Edinburgh). The inclusion criteria for the study were women who have undergone FGM and had experienced childbirth in Scotland. Three women were originally from Somalia, two from The Gambia, one from Ghana, and one from Sudan. Six of them were Muslims and one was Christian. All women had undergone FGM in their countries of origin. Four women had been infibulated and the remaining three could not tell if they have had FGM type 2 or 3.
107	To gain a rich understanding of migrant Pakistani Muslim women's experiences of postnatal depression within motherhood; to inform clinical practice; and to suggest ways of improving supportive services for this group.	East London.	Qualitative: interpretative phenomenology.	Interpretative phenomenological analysis (IPA) theory.	Not specified.	Qualitative: interpretative phenomenology.	Purposive sampling resulted in the recruitment of four migrant Pakistani Muslim women from London aged from 27 to 39.
41	To explore the healthcare experience of vulnerable pregnant migrant women.	London.	Mixed methods: participants were contacted by phone (using a three-way interpreter call if appropriate) and interviewed using a pro forma questionnaire designed to determine their access to antenatal care; barriers to that access; and their experiences during pregnancy, labour, and the immediate postnatal period. Further data was extracted from their records at the Doctors of the	Not specified.	Phone survey.	Qualitative: thematical analysis. Quantitative: not clear.	Pregnant women who presented to the drop-in clinic of the DOTW in London were approached between January 2013 and June 2014.

For peer review only - http://bmjopen.bmj.com/site/about/guidelines.xhtml

			World (DOTW) clinic to see how they had accessed the clinic.				
108	To explore relationships between first-generation migrant Pakistani women and midwives in the South Wales region, focusing on the factors that contribute to these relationships and the ways that these factors might affect the women's experiences of care.	South Wales.	Qualitative: a focused ethnography.	Symbolic interactionism.	Midwives: at lunch break or between clinics. Pakistani women: not clear.	Qualitative: thematical analysis.	Purposive sampling, through midwife gatekeepers, was selected for the initial recruitment of pregnant migrant Pakistani women: emails were sent to all midwives working with migrant women in South Wales. Snowballing was then used to recruit other midwives eligible for participation. Focused, non-participant observations of antenatal booking appointments took place in antenatal clinics across the local health board region over a period of 3-6 months. A total of seven midwives and 15 women were observed during these appointments, which lasted 20-60 minutes each.

For peer review only

For peer review only

File 6: Characteristics of study participants

Participants country of origins

Studies	Country of origins
Duff, L. A., Ahmed, L. B., & Lamping, D. L. (2002).	Bangladesh
Ahmed, S., Macfarlane, A., Naylor, J., & Hastings, J. (2006).	Bangladesh
Cross-Sudworth, F., Williams, A., & Herron-Marx, S. (2011).	Pakistan
Greenhalgh,T., Clinch, M., Afsar, N., Choudhury, Y., Sudra, R., Campbell-Richards, D., & Finer, S. (2015).	Bangladesh, Indian, Sri Lanka, Pakistan
de Chavez, A. C., Ball, H. L., & Ward-Platt, M. (2016).	South Asia including Pakistan, India, Afghanistan, Sri Lanka, Nepal
Hicks, C., & Hayes, L. (1991).	Asian Sub-Continent
Harper Bulman, K., & McCourt, C. (2002).	Somalia
Essen et al. (2011).	Somalia
Moxey, J. M. &Jones, L.L. (2016).	Somalia
Raine, R., Cartwright, M., Richens, Y., Mahamed, Z., & Smith, D. (2010).	Somalia and Bengal
Binder, P., Borne, Y., Johnsdotter, S., & Essen, B. (2012).	Somalia and Ghana
Cresswell, J. A., Yu, G., Hatherall, B., Morris, J., Jamal, F., Harden, A., & Renton, A. (2013).	Somalia, Eastern European, African, Caribeean, South Asia
Binder, P., Johnsdotter, S., & Essen, B. (2012).	Somalia, Ghana, Nigeria, Eritrea and Senegal
O'Shaughnessy, R., Nelki, J., Chiumento, A., Hassan, A., & Rahman, A. (2012).	Gambia, Sierre Leone, Ivory Coast and Nigeria
McLeish, J. (2005).	Black African origin, other not specified

For peer review only

Gorman, D. R., Katikireddi, S. V., Morris, C., Chalmers, J. W. T., Sim, J., Szamotulska, K., . . . Hughes, R. G. (2014).	Poland
Rowe, R. E., Magee, H., Quigley, M. A., Heron, P., Askham, J., & Brocklehurst, P. (2008).	Not specified
Hawkins, S. S., Lamb, K., Cole, T. J., & Law, C. (2008).	Not specified
Briscoe, L., & Lavender, T. (2009).	Not specified
Tucker, A., Ogutu, D., Yoong, W., Nauta, M., & Fakokunde, A. (2010).	Not specified
Jomeen, J., & Redshaw, M. (2013).	Not specified
Feldman, R. (2014).	Not specified
Phillimore, J. (2015).	Not specified
Hufton, E., & Raven, J. (2016).	Not specified
Phillimore, J. (2016).	Not specified
Lephard, E., & Haith-Cooper, M. (2016).	Not specified
**Grey literature**	
Goodwin, L. (2016).	Pakistan
Lamba, R. (2015).	Pakistan-muslim
Bawadi, H. (2009).	Arab muslims
Lee, Jeung Yeon (2010).	Korean
BEMIS SCOTLAND in partnership with Community Food and Health (Scotland). (2013).	Poland, Roma, Czech and African
Almalik, M. (2011).	Europe and Middle East
Baldeh, F. (2013).	Somalia, Gambia, Ghana, Sudan
Nabb, J. (2006).	Algeria, Congo, Angola, Nigeria, Somalia and Iraq
Leeds Family Health. (1992).	Not specified

For peer review only

Warrier, S. (1996).	Asian
Pershad, P., Tyrrell, H. (1995).	Not specified
Ali, N. (2014).	Not specified
Redshaw et al. (2006).	Not specified
Shortall, C., et al. (2015).	Not specified

Antenatal & postnatal

**PEER REVIEWED**	
**Antenatal**	
Rowe, R. E., Magee, H., Quigley, M. A., Heron, P., Askham, J., & Brocklehurst, P. (2008).	Antenatal
Raine, R., Cartwright, M., Richens, Y., Mahamed, Z., & Smith, D. (2010).	Antenatal
Binder, P., Johnsdotter, S., & Essen, B. (2012).	Antenatal
Cresswell, J. A., Yu, G., Hatherall, B., Morris, J., Jamal, F., Harden, A., & Renton, A. (2013).	Antenatal
Feldman, R. (2014).	Antenatal
Greenhalgh,T., Clinch, M., Afsar, N., Choudhury,Y., Sudra, R., Campbell-Richards, D. & Finer, S. (2015).	Antenatal
Moxey, J. M. & Jones, L.L. (2016).	Antenatal
**Ante, intrapartum & postnatal**	
Cross-Sudworth, F., Williams, A., & Herron-Marx, S. (2011).	Ante, intrapartum & postnatal
Duff, L. A., Ahmed, L. B., & Lamping, D. L. (2002).	Ante, intrapartum & postnatal
McLeish, J. (2005).	Ante, intrapartum & postnatal
Lephard, E., & Haith-Cooper, M. (2016).	Ante, intrapartum & postnatal

For peer review only

Harper Bulman, K., & McCourt, C. (2002).	Ante, intrapartum & postnatal
Briscoe, L., & Lavender, T. (2009).	Ante, intrapartum & postnatal
Phillimore, J. (2015).	Ante, intrapartum & postnatal
Phillimore, J. (2016).	Ante, intrapartum & postnatal
Essen & al. (2011).	Ante, intrapartum & postnatal
**Postnatal**	
O'Shaughnessy, R., Nelki, J., Chiumento, A., Hassan, A., & Rahman, A. (2012).	Postnatal
Gorman, D. R., Katikireddi, S. V., Morris, C., Chalmers, J. W. T., Sim, J., Szamotulska, K., . . . Hughes, R. G. (2014).	Postnatal
de Chavez, A. C., Ball, H. L., & Ward-Platt, M. (2016).	Postnatal
Ahmed, S., Macfarlane, A., Naylor, J., & Hastings, J. (2006).	Postnatal
Hufton, E., & Raven, J. (2016).	postnatal
**Intrapartum & postnatal**	
Jomeen, J., & Redshaw, M. (2013).	Intrapartum & postnatal
**Antenatal & postnatal**	
Tucker, A., Ogutu, D., Yoong, W., Nauta, M., & Fakokunde, A. (2010).	Antenatal & postnatal
**Not clear**	
Hicks, C., & Hayes, L. (1991).	Not clear

For peer review only

GREY LITERATURE	
**Antenatal**	
Leeds Family Health. (1992).	Antenatal
Goodwin, L. (2016).	Antenatal
**Ante, intrapartum & postnatal**	
Ali, N. (2004).	Ante, intrapartum & postnatal
Bawadi, H. (2009)	Ante, intrapartum & postnatal
Lee, Jeung Yeon (2010).	Ante, intrapartum & postnatal
Baldeh, F. (2013).	Ante, intrapartum & postnatal
Shortall, C., et al (2015).	Ante, intrapartum & postnatal
BEMIS SCOTLAND in partnership with Community Food and Health (Scotland). (2013).	Ante, intrapartum & postnatal
Warrier, S. (1996)	Ante, intrapartum & postnatal
Redshaw et al. (2006).	Ante, intrapartum & postnatal
**Ante & postnatal**	
Almalik, M. (2011).	Ante & postnatal
**Postnatal**	
Lamba, R. (2015)	postnatal

Immigrant category

PEER REVIEWED	
**Refugees**	
Harper Bulman, K., & McCourt, C. (2002).	refugees
Binder, P., Borne, Y., Johnsdotter, S., & Essen, B. (2012).	refugees

For peer review only

Moxey, J. M. & Jones, L.L. (2016).	refugees
Hufton, E., & Raven, J. (2016).	refugees
**Asylum seekers**	
Feldman, R. (2014).	Asylum seekers
Lephard, E., & Haith-Cooper, M. (2016).	Asylum seekers
O'Shaughnessy, R., Nelki, J., Chiumento, A., Hassan, A., & Rahman, A. (2012).	Asylum seekers
McLeish, J. (2005).	Asylum seekers
**Immigrant category not clear**	
Duff, L. A., Ahmed, L. B., & Lamping, D. L. (2002).	Immigrant category not clear
Ahmed, S., Macfarlane, A., Naylor, J., & Hastings, J. (2006).	Immigrant category not clear
Rowe, R. E., Magee, H., Quigley, M. A., Heron, P., Askham, J., & Brocklehurst, P. (2008).	Immigrant category not clear
Hawkins, S. S., Lamb, K., Cole, T. J., & Law, C. (2008).	Immigrant category not clear
Tucker, A., Ogutu, D., Yoong, W., Nauta, M., & Fakokunde, A. (2010).	Immigrant category not clear
Cross-Sudworth, F., Williams, A., & Herron-Marx, S. (2011).	Immigrant category not clear
Raine, R., Cartwright, M., Richens, Y., Mahamed, Z., & Smith, D. (2010).	Immigrant category not clear
Jomeen, J., & Redshaw, M. (2013).	Immigrant category not clear
Gorman, D. R., Katikireddi, S. V., Morris, C., Chalmers, J. W. T., Sim, J., Szamotulska, K., . . . Hughes, R. G. (2014).	Immigrant category not clear
Greenhalgh,T.,Clinch, M., Afsar, N., Choudhury,Y., Sudra, R., Campbell-Richards, D., & Finer, S. (2015).	Immigrant category not clear
de Chavez, A. C., Ball, H. L., & Ward-Platt, M. (2016)	Immigrant category not clear

For peer review only

Hicks, C., & Hayes, L. (1991).	Immigrant category not clear
Binder, P., Johnsdotter, S., & Essen, B. (2012).	Immigrant category not clear
**Asylum seekers and refugees**	
Briscoe, L., & Lavender, T. (2009).	Asylum seekers and refugees
**Mixed migrant categories**	
Cresswell, J. A., Yu, G., Hatherall, B., Morris, J., Jamal, F., Harden, A., & Renton, A. (2013)	Mixed migrants categories
Phillimore, J. (2016).	Mixed migrant categories
Phillimore, J. (2015).	Mixed migrant categories
Essen, & al. (2011).	Mixed migrant categories

For peer review only

PRISMA 2009 Checklist

Section/topic	#	Checklist item	Reported on page #
**TITLE**			
Title	1	Identify the report as a systematic review, meta-analysis, or both.	1
**ABSTRACT**			
Structured summary	2	Provide a structured summary including, as applicable: background; objectives; data sources; study eligibility criteria, participants, and interventions; study appraisal and synthesis methods; results; limitations; conclusions and implications of key findings; systematic review registration number.	0
**INTRODUCTION**			
Rationale	3	Describe the rationale for the review in the context of what is already known.	2
Objectives	4	Provide an explicit statement of questions being addressed with reference to participants, interventions, comparisons, outcomes, and study design (PICOS).	4 & 6
**METHODS**			
Protocol and registration	5	Indicate if a review protocol exists, if and where it can be accessed (e.g., Web address), and, if available, provide registration information including registration number.	Yes https://bmjopen.bmj.com/content/bmjopen/7/7/e016988.full.pdf
Eligibility criteria	6	Specify study characteristics (e.g., PICOS, length of follow-up) and report characteristics (e.g., years considered, language, publication status) used as criteria for eligibility, giving rationale.	4 & 6
Information sources	7	Describe all information sources (e.g., databases with dates of coverage, contact with study authors to identify additional studies) in the search and date last searched.	Supplementary files
Search	8	Present full electronic search strategy for at least one database, including any limits used, such that it could be repeated.	Supplementary file
Study selection	9	State the process for selecting studies (i.e., screening, eligibility, included in systematic review, and, if applicable, included in the meta-analysis).	7 and Supplementary files

For peer review only

PRISMA 2009 Checklist

Data collection process	10	Describe method of data extraction from reports (e.g., piloted forms, independently, in duplicate) and any processes for obtaining and confirming data from investigators.	8
Data items	11	List and define all variables for which data were sought (e.g., PICOS, funding sources) and any assumptions and simplifications made.	6
Risk of bias in individual studies	12	Describe methods used for assessing risk of bias of individual studies (including specification of whether this was done at the study or outcome level), and how this information is to be used in any data synthesis.	9,10,11

n

Section/topic	#	Checklist item	Reported on page #
Risk of bias across studies	15	Specify any assessment of risk of bias that may affect the cumulative evidence (e.g., publication bias, selective reporting within studies).	9,10,11
Additional analyses	16	Describe methods of additional analyses (e.g., sensitivity or subgroup analyses, meta-regression), if done, indicating which were pre-specified.	11
**RESULTS**			
Study selection	17	Give numbers of studies screened, assessed for eligibility, and included in the review, with reasons for exclusions at each stage, ideally with a flow diagram.	Supplementary files and Prisma diagram
Study characteristics	18	For each study, present characteristics for which data were extracted (e.g., study size, PICOS, follow-up period) and provide the citations.	Supplementary file – Table of included studies
Risk of bias within studies	19	Present data on risk of bias of each study and, if available, any outcome level assessment (see item 12).	Supplementary files
Results of individual studies	20	For all outcomes considered (benefits or harms), present, for each study: (a) simple summary data for each intervention group (b) effect estimates and confidence intervals, ideally with a forest plot.	n/a

For peer review only

PRISMA 2009 Checklist

Synthesis of results	21	Present results of each meta-analysis done, including confidence intervals and measures of consistency.	n.a
Risk of bias across studies	22	Present results of any assessment of risk of bias across studies (see Item 15).	11
Additional analysis	23	Give results of additional analyses, if done (e.g., sensitivity or subgroup analyses, meta-regression [see Item 16]).	11
**DISCUSSION**			
Summary of evidence	24	Summarize the main findings including the strength of evidence for each main outcome; consider their relevance to key groups (e.g., healthcare providers, users, and policy makers).	14
Limitations	25	Discuss limitations at study and outcome level (e.g., risk of bias), and at review-level (e.g., incomplete retrieval of identified research, reporting bias).	19
Conclusions	26	Provide a general interpretation of the results in the context of other evidence, and implications for future research.	19
**FUNDING**			
Funding	27	Describe sources of funding for the systematic review and other support (e.g., supply of data); role of funders for the systematic review.	1

From: Moher D, Liberati A, Tetzlaff J, Altman DG, The PRISMA Group (2009). Preferred Reporting Items for Systematic Reviews and Meta-Analyses: The PRISMA Statement. PLoS Med 6(7): e1000097. doi:10.1371/journal.pmed1000097

For more information, visit: **www.prisma-statement.org**.

For peer review only

For peer review only - http://bmjopen.bmj.com/site/about/guidelines.xhtml

PRISMA 2009 Checklist

For peer review only

PRISMA 2009 Checklist

For peer review only
